# Liver Antioxidant, Transcriptomic and Metabolomic Responses to Heatwaves in an Aquatic Turtle Species, *Pelodiscus sinensis*

**DOI:** 10.3390/ani16121870

**Published:** 2026-06-17

**Authors:** Han-Bing Zhang, Wan-Ying Lin, Zhi-Hao Cao, Jian-Fang Gao, Hong-Liang Lu

**Affiliations:** Zhejiang Provincial Key Laboratory of Wetland Intelligent Monitoring and Ecological Restoration, Hangzhou Normal University, Hangzhou 311121, China; 2023210304025@stu.hznu.edu.cn (H.-B.Z.); 2025111010070@stu.hznu.edu.cn (W.-Y.L.); 2024011010014@stu.hznu.edu.cn (Z.-H.C.)

**Keywords:** heatwave frequency, oxidative stress, energy metabolism, immune defense

## Abstract

The physiological and metabolic responses of juvenile Chinese softshell turtles, *Pelodiscus sinensis*, exposed to single or double heatwaves (33 °C for 4 days) were investigated based on liver antioxidant assay and transcriptomic and metabolomic analyses. Heatwave exposure increased superoxide dismutase and catalase activities, but did not significantly alter malondialdehyde and reactive oxygen species levels, indicating enhanced antioxidant defense. Transcriptomic analysis revealed the differential expression of genes involved in carbohydrate metabolism, immune function, cardiovascular regulation, and signal transduction, with more pronounced alterations in single-heatwave-exposed turtles. Additionally, metabolic dysregulation in amino acids was revealed by significantly altered levels of some key amino acids and their derivatives. Compared with single-heatwave-exposed turtles, fewer differentially expressed genes and less metabolic disruptions in double-heatwave-exposed turtles probably indicated less physiological disorders under recurrent heat stress. Although *P. sinensis* can adopt physiological and metabolic adjustments to mitigate the adverse effects of short-term heatwaves, repeated heatwave exposure might still cause severe physiological consequences in aquatic turtles. These findings may have important implications for the conservation of freshwater turtle species under future climate change scenarios.

## Simple Summary

Heatwaves are becoming more frequent and intense due to global climate change, posing a growing threat to aquatic animals. However, most studies have focused on fish and marine invertebrates, while much less is known about how aquatic reptiles respond to these extreme temperature events. The physiological alterations of aquatic turtle juveniles after simulated heatwave exposure were evaluated here. Turtle juveniles were shown to activate antioxidant defenses, but their energy metabolism, immune function, and cardiovascular health might be disrupted under heat stress. Interestingly, turtles that experienced repeated heatwaves showed fewer transcriptomic alterations than those that experienced a single heatwave. This study would contribute to a better understanding of how aquatic turtles cope with extreme heat and highlight the importance of considering heatwave frequency in future conservation efforts for turtle species.

## 1. Introduction

Heatwaves, defined as short-term extreme high-temperature events lasting several days, are becoming increasingly intense and frequent due to climate change, and pose severe threats to living organisms, including humans [1,2]. Heatwaves may affect various physiological and biochemical processes (such as energy expenditure, immune function and infection resistance) of organisms, thereby harming their health and survival [3,4,5]. For example, marine heatwaves significantly reduce the body size and increase the mortality rate of aquatic invertebrates and fish [6,7,8,9]. The vulnerability of organisms to heatwaves varies across different species, as well as across different developmental stages [10,11]. Behavioral (such as avoidance of high-temperature conditions) or physiological adjustments (such as shift to anaerobic metabolism) can be employed as short-term adaptive coping mechanisms for mitigating the adverse effects of heatwaves [12,13,14]. However, these adjustments may increase energy consumption or even lead to energy reserve depletion [15,16,17]. When heatwaves occur repeatedly within a relatively short period of time, the energy depletion of organisms becomes increasingly severe, further exacerbating species vulnerability [18].

In aquatic ecosystems, besides direct adverse effects, heatwaves may also promote rapid growth and reproduction of algae, which possibly causes the depletion of dissolved oxygen in water and the release of large amounts of algal toxins into the water, thereby leading to mass mortality of aquatic organisms [19,20]. The understanding of their impacts on non-fish vertebrates (such as amphibian and reptile species) still remains very limited. A few existing studies have evaluated the heatwave-induced changes in locomotor, immune performance, and oxidative physiology in several anuran and turtle species [21,22,23]. To fill this gap, the heatwave effects on liver physiological performance were evaluated in an aquatic turtle species, *Pelodiscus sinensis,* in this study. The Chinese softshell turtle, *P. sinensis*, is the most commonly farmed freshwater turtle in China for food or used in traditional medicine. This species is susceptible to environmental stresses and is suitable as a model species for exploring potential heatwave effects in aquatic reptiles [24]. Transcriptomic and metabolomic analysis can provide a comprehensive and detailed assessment of the physiological and biochemical changes induced by environmental stresses, including heatwaves [25,26]. Transcriptomic profiles revealed genes related to energy metabolism, body defense and cell proliferation were differentially expressed after exposure to heatwaves, but the number of differentially expressed genes decreased in the subsequent heatwaves in the pearl oyster, *Pinctada maxima* [18]. Under heatwave stress, the Pacific clams (*Ruditapes philippinarum*) would upregulate the expression of genes involved in unfolded protein response to reduce oxidative damage and increase saturated fatty acid content to lower cell membrane fluidity [16]. Here, we investigated physiological and biochemical changes in the livers of juvenile *P. sinensis* after exposure to single or double heatwaves primarily based on transcriptomic and metabolomic profiling, and hypothesized that heatwave exposure would cause oxidative damage, disrupt energy metabolism and reduce immune ability in *P. sinensis,* but that these adverse effects might be mitigated in subsequent heatwaves. Our study might provide novel insights into the adaptive physiological responses of freshwater turtle species under heatwave stress.

## 2. Materials and Methods

### 2.1. Animals and Experimental Design

**Figure 1:**
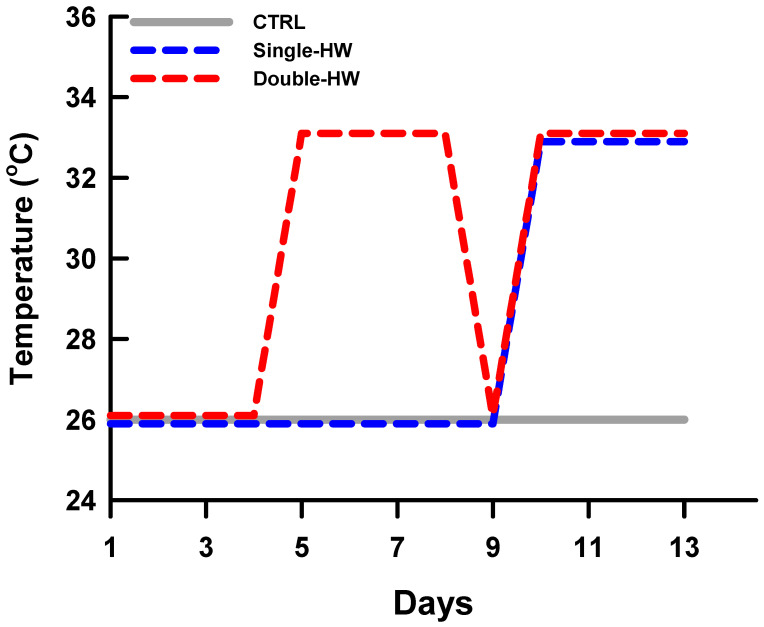
Schematic diagram showing different heatwave treatments.

Fertilized eggs of *P. sinensis* were purchased from a private hatchery (Huzhou city, Zhejiang Province, China) in May 2025, and incubated in moist vermiculite substrate at an incubation temperature of 28 °C. After hatching, juvenile turtles were individually reared in aquaria with a water depth of 2 cm and placed in an artificial climate chamber set at 26 °C with a 12 h light/12 h dark cycle. Turtles were fed commercial pellets ad libitum daily. Heatwave events may occur between July and August. The recorded daily mean water temperature of some waterbodies in Huzhou normally fluctuates between 22 and 28 °C in July, but it can reach about 33 °C during days when a heatwave occurs. Accordingly, three temperature treatments (control, single- and double-heatwave) were established in this study. About two weeks later, 12 healthy juvenile turtles (body mass: mean ± standard error, 7.31 ± 0.19 g) were randomly selected and assigned to 3 different groups (4 individuals per group). Turtles were maintained in the artificial climate chamber at 26 °C as the control treatment (hereafter as CTRL); and maintained at 26 °C for 9 days, then experienced a 4-day heatwave (rising to 33 °C within 3 h, hereafter as single-HW); or maintained at 26 °C for 4 days, then experienced two-time 4-day heatwaves with a 1-day recovery at 26 °C between them (hereafter as double-HW), respectively (Figure 1). The general quality parameters of water in aquaria were: pH, 7.02 ± 0.04; dissolved oxygen, 7.40 ± 0.08 mg/L and NH_3_-N, 0.12 ± 0.01 mg/L. During the experimental period, turtles were fed daily with sufficient commercial food pellets.

Thirteen days later, turtles were anesthetized and dissected on ice, and their livers were harvested immediately, rapidly frozen in liquid nitrogen and stored in an ultra-low temperature freezer at −80 °C before analysis.

### 2.2. Liver Antioxidant Response

A portion of liver tissue (approximately 5 mg) was crushed, homogenized in normal saline, and centrifuged at 4 °C for 10 min. The supernatant was transferred into a new centrifuge tube, diluted at 1:10 with normal saline and used for determining the total protein, superoxide dismutase (SOD) and catalase (CAT) activities, and malondialdehyde (MDA) and reactive oxygen species (ROS) contents. The kits for these detections were purchased from Nanjing Jiancheng Bioengineering Institute (Nanjing, China). Total protein content was determined using the Bradford method at 595 nm, SOD activity was determined using the xanthine oxidase assay at 550 nm, CAT activity was determined using the ammonium molybdate colorimetric assay at 405 nm, MDA content was determined using the thiobarbituric acid assay at 532 nm, and ROS content was determined using the dichlorofluorescin assay at 488 nm excitation and 525 nm emission.

### 2.3. Liver Transcriptomic Analysis

A portion of liver tissue (approximately 50 mg) was used for transcriptomic analysis. Total RNA was extracted using Trizol reagent (Invitrogen, Carlsbad, CA, USA), and the integrity and purity was assessed via agarose gel electrophoresis and Nanodrop analysis. mRNA was enriched using Oligo(dT) magnetic beads (ThermoFisher Scientific: Waltham, MA, USA), fragmented, and then reverse-transcribed into double-stranded cDNA. cDNA underwent end repair, adapter ligation, and PCR amplification to construct sequencing libraries. After being quality-checked, libraries were 2 × 150 bp paired-end sequenced on the Illumina NovaSeq platform (Illumina, San Diego, CA, USA).

### 2.4. Liver Metabolomic Analysis

Another portion of liver tissue (approximately 50 mg) was used for non-targeted metabolomic analysis based on liquid chromatography-mass spectrometry (LC-MS). Liver tissue samples were homogenized, incubated and centrifuged at 4 °C, and their supernatants were collected and freeze-dried. The residue was redissolved and filtered with a 0.22 μm filtering membrane before LC-MS analysis. Detailed procedures can be referenced in previous work [27]. Liver transcriptomic and metabolomic analysis was done by Hangzhou Kaitai Biotech. Co., Ltd. (Hangzhou, China).

### 2.5. Data Processing and Analysis

One-way analysis of variance (ANOVA) followed by Tukey’s test was performed to determine the difference in body size and liver antioxidant activity among groups, and partial eta-square (*η*_p_^2^) was presented as the measure of effect size. For liver transcriptomic data, they were processed as described in previous work [28]. Briefly, after being filtered and checked for quality, generated clean reads were mapped to the reference genome of *P. sinensis* (https://www.ncbi.nlm.nih.gov/assembly/GCF_000230535.1/, (accessed on 15 December 2025)) with HISAT2 v2.2.1 [29]. Expression levels of genes were calculated using StringTie v2.2.1 [30] and differentially expressed genes (DEGs) were identified using edgeR v3.36.0 with a significance threshold of false discovery rate (FDR) < 0.05 and absolute log_2_fold change (log_2_FC) > 1. The Gene Ontology (GO) and Kyoto Encyclopedia of Genes and Genomes (KEGG) pathway functional enrichment analysis of DEGs was performed using clusterProfiler v4.18.3 [31].

Liver LC-MS data were processed as described in previous works [27,32]. Briefly, after being converted and processed, data were analyzed using principal component analysis (PCA) to determine among-group variations using Ropls v1.22.0 [33]. Liver metabolites were identified by searching in the Human Metabolome Database (HMDB), MassBank, and the mzcloud database. One-way ANOVA was used to determine differences in liver metabolites among groups. Differential metabolites were identified with a *p*-value < 0.05. Prior to parametric statistics, the Shapiro–Wilk test was used to determine data normality, and Levene’s test was used to determine variance homogeneity, respectively.

## 3. Results

### 3.1. Antioxidant Activity

**Figure 2:**
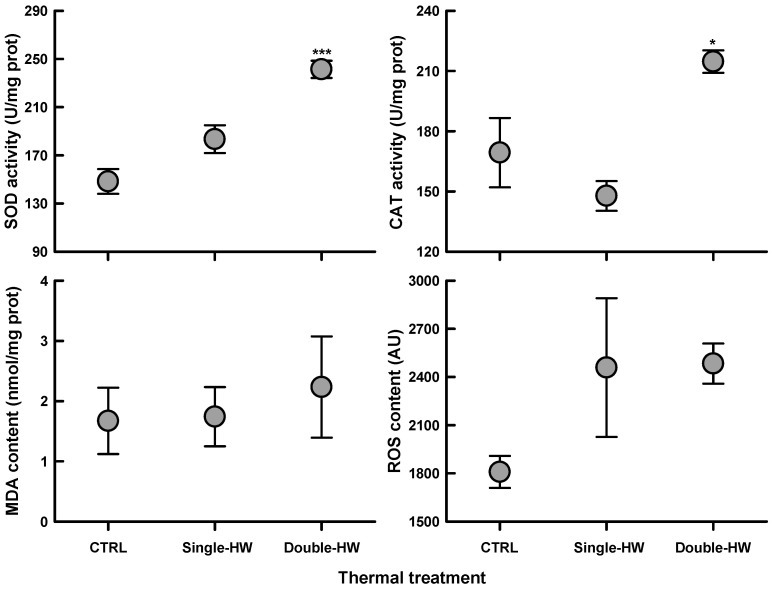
Mean values (±standard errors) for liver SOD and CAT activities, MDA and ROS levels in CTRL, single- and double-HW groups of *Pelodiscus sinensis* juveniles, respectively. Asterisks indicated significant differences compared with the CTRL group (* *p* < 0.05, *** *p* < 0.001).

There was no significant among-group difference in the body size (mass) of turtles (*F*_2,9_ = 0.58, *p* = 0.580, *η*_p_^2^ = 0.11), as well as in the total protein content in their liver tissue (*F*_2,9_ = 3.14, *p* = 0.092, *η*_p_^2^ = 0.41). Heatwave exposure significantly affected the liver SOD (*F*_2,9_ = 22.84, *p* < 0.001, *η*_p_^2^ = 0.84) and CAT (*F*_2,9_ = 9.12, *p* < 0.01, *η*_p_^2^ = 0.67) activities of turtles. The SOD and CAT activities of double-HW-exposed turtles were higher than those of CTRL turtles (Figure 2). Liver MDA (*F*_2,9_ = 0.22, *p* = 0.803, *η*_p_^2^ = 0.05) and ROS levels (*F*_2,9_ = 2.07, *p* = 0.182, *η*_p_^2^ = 0.31) showed an increasing trend after heatwave exposure, but the differences were not statistically significant (Figure 2).

### 3.2. Transcriptomic Profile

**Figure 3:**
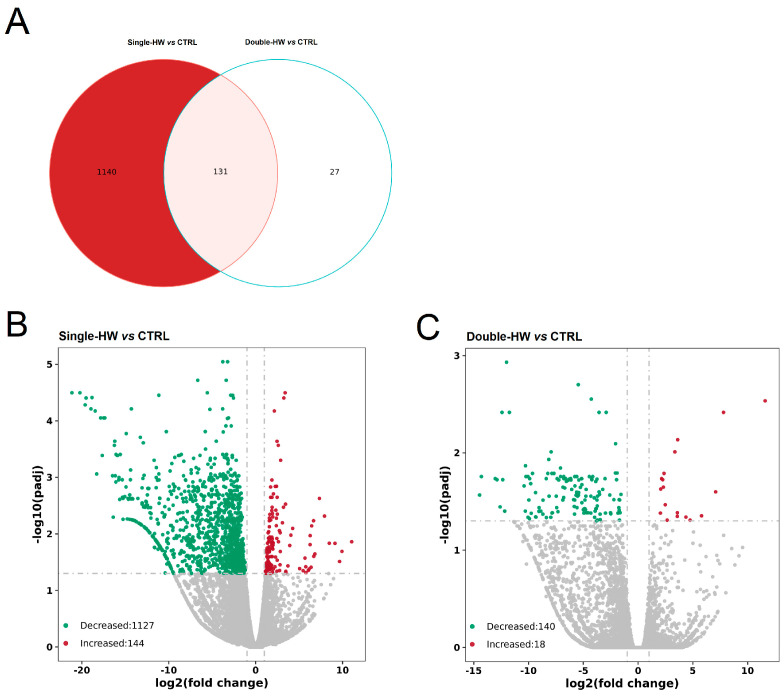
Venn (**A**) and volcano diagrams (between single-HW and CTRL, (**B**); between double-HW and CTRL (**C**)) of DEGs in livers of *Pelodiscus sinensis* juveniles under heatwave stress.

After being filtered, a total of 602,675,264 qualified clean reads were generated from our liver samples (Supplementary Table S1) and mapped against the reference genome with a total mapping rate of 83.8% (Table 1). After being annotated and analyzed for differential expression, compared with the CTRL group, 1271 and 158 DEGs were respectively identified in single- and double-HW groups, with 131 DEGs being shared by the heatwave-treated groups (Figure 3A). Of them, 144 and 18 DEGs were upregulated, 1127 and 140 DEGs were downregulated in single- and double-HW groups, respectively (Figure 3B,C, Supplementary Table S2).

**Figure 4:**
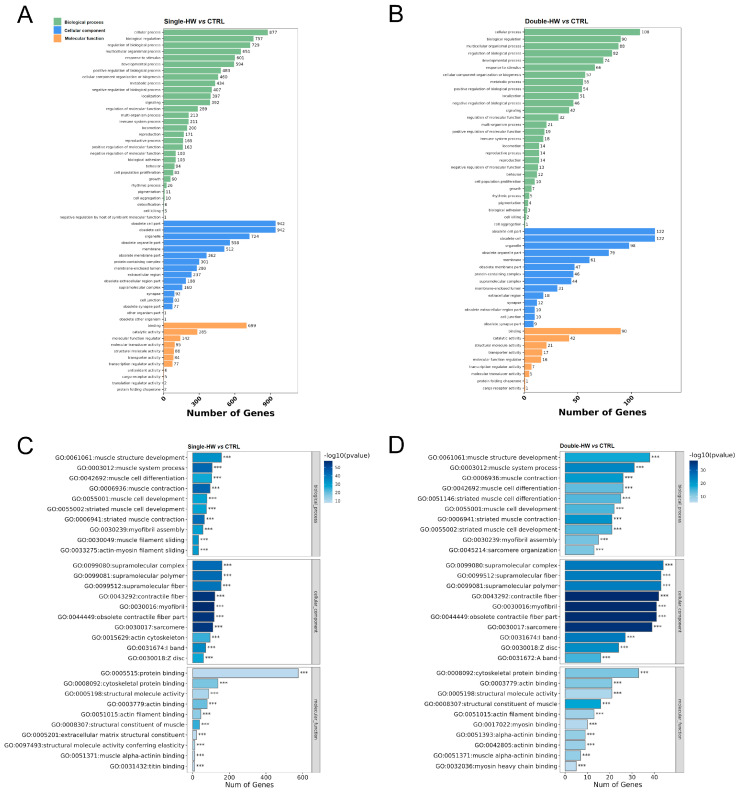
GO enrichment analysis on DEGs in livers of *Pelodiscus sinensis* juveniles under heatwave stress. Top 10 most significantly enriched GO pathways in each category between single-HW and CTRL (**A**,**C**) and between double-HW and CTRL (**B**,**D**) groups (*** *p* < 0.001).

GO enrichment analysis showed that DEGs were mainly enriched in cellular processes, biological regulation, regulation of biological and multicellular organismal processes in the biological process category, obsolete cell and organelle parts in the cellular component category, and binding and catalytic activity in the molecular function category (Figure 4A,C). Compared with the CTRL group, differential biological processes primarily included muscle structure development, muscle cell differentiation and development; differential cellular components primarily included supramolecular complex, fiber and polymer; and differential molecular functions primarily included protein and actin binding, and structural molecule activity in single- and double-HW groups (Figure 4B,D).

**Figure 5:**
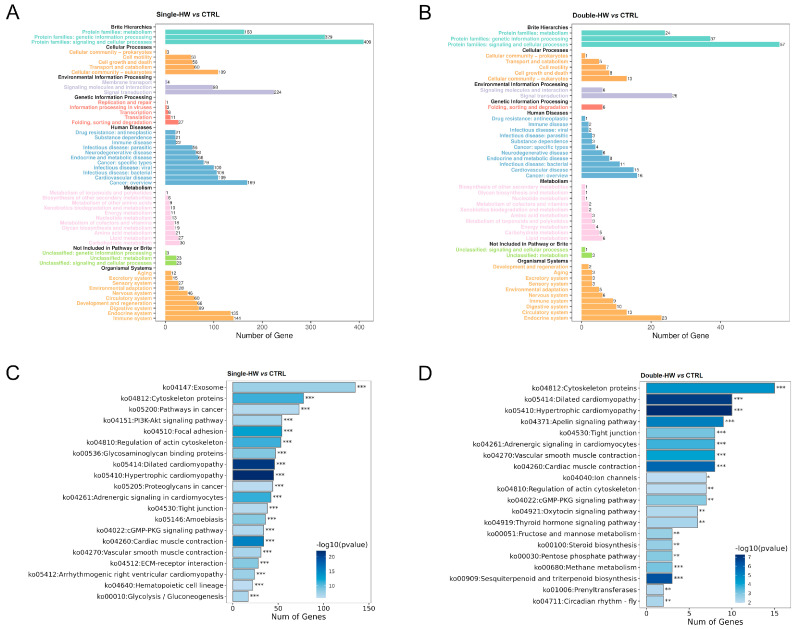
KEGG enrichment analysis on DEGs in livers of *Pelodiscus sinensis* juveniles under heatwave stress. Top 20 most significantly enriched KEGG pathways between single-HW and CTRL (**A**,**C**) and between double-HW and CTRL (**B**,**D**) groups (* *p* < 0.05, ** *p* < 0.01, *** *p* < 0.001).

KEGG enrichment analysis showed that, between the single-HW and CTRL group, and between the double-HW and CTRL group, DEGs were enriched in 51 and 45 KEGG pathways, respectively (Figure 5A,C). Metabolic pathways related to carbohydrate metabolism (such as glycolysis/gluconeogenesis and pyruvate metabolism in single-HW group, pentose phosphate pathway in double-HW group, fructose and mannose metabolism in both groups), cardiovascular disease (such as arrhythmogenic right ventricular cardiomyopathy and viral myocarditis in single-HW group, hypertrophic cardiomyopathy and dilated cardiomyopathy in both groups), circulatory system (such as cardiac muscle contraction, vascular smooth muscle contraction and adrenergic signaling in cardiomyocytes in both groups), and signal transduction (such as PI3K-Akt signaling pathway, calcium signaling pathway in single-HW group, and apelin signaling pathway, cGMP-PKG signaling pathway in both groups) were altered significantly after heatwave exposure (Supplementary Table S3, Figure 5B,D). There were more significantly altered metabolic pathways in the single-HW group than in the double-HW group. For example, some pathways related to the immune system (such as hematopoietic cell lineage, leukocyte transendothelial migration and platelet activation) and endocrine system (such as thyroid hormone signaling pathway, relaxin signaling pathway and insulin secretion) were altered significantly in the single-HW group but not in the double-HW group (Supplementary Table S3).

### 3.3. Metabolomic Profile

**Figure 6:**
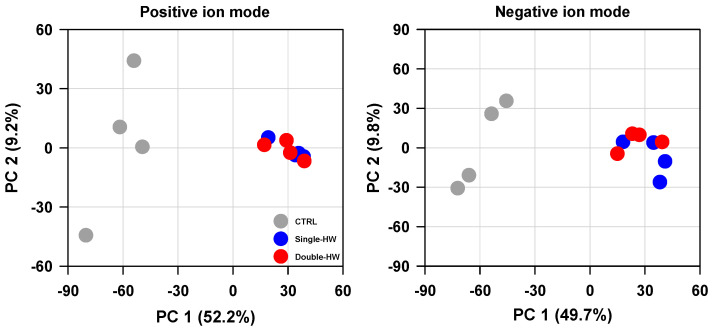
Score plots for principal component analysis (PCA) of liver metabolomic profiles in *Pelodiscus sinensis* juveniles under heatwave stress in the positive and negative ion mode, respectively.

PCA analysis on liver metabolomic data could exhibit clear separations between CTRL and heatwave-treated groups, with the first two principal components explaining 61.4% and 59.5% of the total variation in the positive and negative ion modes, respectively (Figure 6). After being identified and analyzed for statistical differences, compared with the CTRL group, 377 (262 upregulated and 115 downregulated) and 445 (339 upregulated and 106 downregulated) differentially significant metabolites were shown in single- and double-HW groups, respectively (Supplementary Table S4). These identified metabolites with significant differences were primarily involved in the biosynthesis and metabolism of amino acids. For example, the levels of some amino acids (such as isoleucine, aspartic acid, histidine and glutamine) decreased somewhat, and those of amino acid derivatives (argininosuccinic acid disodium, α-ketoisovaleric acid) increased in the single- or double-HW groups (Table 2, Supplementary Table S4). The levels of glutathione decreased in the double-HW group, and those of some metabolites associated with substrate transport, thermosensation and neural activity (such as taurine and anandamide) increased in both heatwave-treated groups (Table 2).

## 4. Discussion

In this study, physiological and metabolic changes under heatwave stress were preliminarily investigated in an aquatic turtle, *P. sinensis*. Although a relatively small sample size (*n* = 4 per group) was used in this study, these variables with significant differences, such as SOD and CAT activities, and some identified metabolite levels, showed large effect sizes with acceptably high statistical powers (>0.67). Therefore, these experimental results should be reliable. Obvious histological, physiological, and biochemical changes after heatwave exposure are observed in some aquatic species, including invertebrates, fish and amphibians [3,22,26,34,35,36]. Antioxidant responses under heatwave stress have been frequently evaluated, and it is expected that heatwaves may enhance the production of ROS and induce oxidative stress in aquatic organisms [3,22,29]. However, these responses often vary significantly across different aquatic species. Altered antioxidant enzyme (SOD and CAT) activities, and elevated MDA and ROS levels could indicate increased oxidative damage [37,38,39]. Despite an increasing trend in MDA and ROS levels, it was not statistically significant. Inconsistent with the expectation, increased SOD and CAT activities, but no altered MDA and ROS levels, probably indicated the activation of antioxidant defenses in the livers of *P. sinensis* under heatwave stress. *P. sinensis* individuals might have the ability to effectively cope with heatwave stress through improving antioxidant defense. In fact, the oxidative damage caused by less intense and less frequent heatwaves would be limited in some species. For example, increased antioxidant enzyme activities and decreased MDA level after short-term heatwave exposure were also shown in mollusc and amphibian species, suggesting that they could adapt to such temperature changes [3,21,40].

More detailed physiological and biochemical changes under environmental stresses can be revealed by transcriptomic and metabolomic analysis. For example, transcriptomic profiles revealed that heatwaves would disrupt metabolic pathways related to carbohydrate and energy metabolism, immune function, signal transduction and defense response in various aquatic organisms [18,26,36]. Several carbohydrate metabolic pathways, including glycolysis/gluconeogenesis, pentose phosphate pathway, and fructose and mannose metabolism, were shown to be downregulated, probably reflecting heatwave-induced energy metabolic dysregulation in *P. sinensis*. Glycolysis/gluconeogenesis and the pentose phosphate pathway were the central pathways of glucose metabolism and can provide the energy required for life activities [41]. Despite not being validated through quantitative real-time reverse transcription polymerase chain reaction (RT-qPCR), the downregulation of some genes involved in glycolysis/gluconeogenesis and pentose phosphate pathway (such as *HK1*, *PFKM*, *PGM2L1* and *PKM*), as well as in pyruvate metabolism (such as *LDH-A*, *ME1* and *ME3*), implied a reduction in energy supply, thereby potentially influencing the functional performances (such as immune ability) [25,41]. Contrarily, energy expenditure was expected to increase because additional energetic costs (e.g., for protein synthesis) would be incurred to withstand thermal stress, although it might decrease in subsequent heatwave exposure [18,42]. Long-term heat stress may inhibit the secretion of insulin, thereby affecting glucose metabolism [43]. Downregulated expressions of some genes involved in insulin signaling pathway(such as *SLC2A4*) could be observed in short-term heatwave-exposed turtles here. Other endocrine-related metabolic pathways, such as the thyroid hormone signaling pathway, might be disrupted by heatwave exposure, potentially influencing the growth of aquatic organisms [44,45].

Immune defense serves as a crucial protective mechanism for organisms responding to environmental stress [46]. Hematopoietic cell lineage, leukocyte transendothelial migration and platelet activation are critical processes in immune responses to injury or infection [47]. Although further validation was required by RT-qPCR, downregulated expressions of some genes related to these pathways (such as *IL5RA*, *ITGA4*, *PECAM1* and *COL11A1*) might suggest a decrease in immune function after heatwave exposure. The adverse effect of heatwaves on immune responses was observed in some aquatic organisms, including molluscs and fish species [3,18,48], but it could also be minor in some molluscs, such as *Saccostrea glomerata* [49]. Despite the reduced ability of the immune system, the synthesis of some proteins (e.g., heat shock proteins as well as other molecular chaperones) might be important to counter abrupt thermal stress [37,38]. Upregulated expressions of related genes (such as *HSP90AA1*, *HSPA5*, *HSPH1*, *DNAJA4* and *SERPINH1*) might promote the ability for proper protein folding and to efficiently clear misfolded proteins, thereby potentially contributing to reducing or eliminating the adverse effects of heatwave stress [38].

Based on transcriptomic data, heatwaves seem to influence the cardiovascular performance and the circulatory system of turtles. The frequency and amplitude of cardiac contractility might be increased in order to ensure oxygen supply and maintain sufficient circulation under short-term heatwave exposure (but might decrease under prolonged heatwaves) [50]. Sustained enhancement of cardiac contractility would increase myocardial burden and lead to cardiomyocyte hyperplasia, potentially triggering cardiovascular diseases [51]. Although the cardiac performances (such as heart rate and amplitude) were not evaluated directly in this study, the expression dysregulation of genes related to pathways of cardiovascular disease and the circulatory system might indicate an impaired cardiovascular function in heatwave-exposed turtles. Some predicted signaling pathways related to the regulation of the cardiovascular system (e.g., apelin and cGMP-PKG signaling pathway) were also altered significantly in heatwave-exposed turtles. Certainly, whether heatwave stress would directly induce immune impairment and cardiovascular dysfunction in turtles should be further investigated in future studies.

Heatwave-induced alterations in the amino acid metabolism of juvenile *P. sinensis* were revealed by metabolomic analysis. A number of amino acids (such as L-isoleucine, L-aspartic acid, L-glutamine and L-histidine) were shown to decrease in heatwave-exposed turtles, indicating amino acid metabolic disorders. The change trend of amino acids under heatwave stress could be different in other aquatic species [52,53]. Amino acids play important roles in various biological processes, such as protein synthesis and antioxidant and immune responses [52]. For example, reduced glutamate and glutamine levels might cause a deficiency of energy supply, suppression of immune function and neural activity [52]. Some amino acids (such as isoleucine, aspartic acid and histidine) can be converted into intermediates (such as pyruvate, oxaloacetic acid and α-ketoglutaric acid) of the tricarboxylic acid (TCA) cycle, thereby entering it and influencing energy metabolism [25,52]. Heatwave-induced alterations in other metabolic pathways, such as nucleotide metabolism, could be reflected by significantly altered liver metabolites in exposed turtles (such as increased deoxyuridine and xanthosine levels, which were often associated with the disruption of nucleotide metabolism). Integrated transcriptomic and metabolomic analysis might provide deeper biological insight into how DEGs and differential metabolites converged on shared metabolic pathways. However, enrichment analysis of differential liver metabolites could not identify any differential metabolic pathways between groups. Enriched metabolic pathways seemed to be inconsistent between transcriptomic and metabolomic profiles. Therefore, integration analyses (such as gene–metabolite correlation, joint enrichment and network analysis) were not performed here.

The impact of double-HW exposure might be weaker than that of single-HW exposure, probably reflecting significantly fewer DEGs, as well as less differentially enriched metabolic pathways, in the double-HW group. Although it was speculated to originate from enriched metabolic pathways, reduced immune defense, and the resultant lower resistance to pathogenic bacteria, and altered metabolic pathways related to signal transduction, protein synthesis and folding were revealed in single-HW turtles, but did not necessarily occur in double-HW ones. These findings might indicate that *P. sinensis* exhibited a certain degree of adaptation to environmental thermal variations and might cope with less intense subsequent heatwaves. Contrarily, enhanced negative responses under subsequent or prolonged heatwaves were observed in some aquatic invertebrates [3,18,38]. For example, more DEGs related to energy metabolism and body defense were shown to be upregulated or downregulated during the second heatwave than during the first heatwave in a mollusc species, *Pinctada maxima* [18]. We inferred that differential outcomes might result from the difference in resistance to thermal extremes across species, with relatively higher resistance potential for aquatic vertebrates, such as *P. sinensis*.

## 5. Conclusions

This study preliminarily investigated physiological and biochemical responses under heatwave stress in an aquatic turtle, *P. sinensis*, based on transcriptomic and metabolomic analysis. Heatwave exposure would activate antioxidant defense responses, reflected in increased SOD and CAT activities, but might induce disruptions in energy metabolism and affect the functional performances of turtles. Metabolic disruptions in double-HW turtles seemed to be reduced, which might suggest that *P. sinensis* partially adapted to recurrent thermal stress through enhanced defense mechanisms and metabolic adjustments. Despite having higher resistance to thermal extremes, alterations in metabolic pathways related to energy supply, immune defense, and cardiovascular health indicated that heatwaves still posed substantial physiological challenges in aquatic turtles. Considering more frequent and intense extreme heat events, these findings would have important implications for the conservation of freshwater turtle species under future scenarios.

## Figures and Tables

**Table 1:** Summary of RNA-seq transcriptomic data of *Pelodiscus sinensis* juveniles under heatwave stress.

Group	Raw Reads	Clean Reads	Clean Bases (GB)	Q20%	Q30%	GC%	Total Mapped Rate (%)
CTRL	50,866,718 ± 3,470,385	49,498,330 ± 3,363,196	7.44 ± 0.50 G	98.7 ± 0.01	95.8 ± 0.01	49.2 ± 0.01	83.0 ± 0.01
Single-HW	57,182,109 ± 3,022,298	54,094,903 ± 3,360,734	8.13 ± 0.50 G	98.7 ± 0.01	95.9 ± 0.01	48.3 ± 0.01	84.6 ± 0.01
Double-HW	48,307,155 ± 2,648,505	47,075,582 ± 2,453,497	7.08 ± 0.37 G	98.7 ± 0.01	95.9 ± 0.01	49.1 ± 0.01	83.6 ± 0.01
In total	52,118,661 ± 1,956,425	50,222,939 ± 1,836,489	7.55 ± 0.28 G	98.7 ± 0.01	95.9 ± 0.01	48.9 ± 0.01	83.7 ± 0.01

**Table 2:** Liver metabolites differed between the single-HW and CTRL groups, or between the double-HW and CTRL groups. The asterisk indicated significant differences (* *p* < 0.05, ** *p* < 0.01, *** *p* < 0.001).

	Single-HW vs. CTRL		Double-HW vs. CTRL	
Metabolite	Log_2_FC	*p*	Log_2_FC	*p*
L-Isoleucine	−1.64	*	−2.24	**
L-Aspartic acid	−0.66		−0.72	*
L-Histidine	−1.56	***	−1.80	***
L-Glutamic acid	−2.03	**	−1.79	**
L-Glutamine	−1.98	**	−1.68	**
Glutathione	0.75		−0.82	*
L-Ornithine	−2.27	***	−2.10	***
Pyroglutamic acid	1.62		2.59	*
Aminopropylcadaverine	1.68	*	1.92	*
Argininosuccinic acid disodium	4.85	***	4.18	***
α-Ketoisovaleric acid	2.14		2.23	*
(S)-Methylmalonic acid semialdehyde	0.29		0.51	*
Taurine	2.66	***	2.80	***
Deoxyuridine	1.20	*	1.29	*
Xanthosine	1.90	***	1.11	**
Allantoic acid	1.77	**	2.19	***
Anandamide	4.80	***	3.26	*

## Data Availability

All data generated by this study are available in this manuscript and the accompanying Supplementary Materials.

## Supplementary Materials

The following supporting information can be downloaded at: https://www.mdpi.com/article/10.3390/ani16121870/s1. Table S1: Raw reads and clean reads from each library; Table S2: Differentially expressed genes in single- and double-HW groups compared with the CTRL group; Table S3: Results of KEGG pathway functional enrichment analysis of DEGs. Table S4: Differential metabolites in single- and double-HW groups compared with the CTRL group.
