# Supplementary material for: Liver Antioxidant, Transcriptomic and Metabolomic Responses to Heatwaves in an Aquatic Turtle Species, Pelodiscus sinensis
Source: Animals (Basel). 2026 Jun 17;16(12):1870. doi: 10.3390/ani16121870 (PMC13295678; doi:10.3390/ani16121870)
Supplement: Supplementary file 1 [file animals-16-01870-s001.zip › Supplementary Table S1.pdf]

| Sample | Raw data reac | Raw data base | Raw data q2 | Raw data q3 | Raw data q2 | Raw data q3 |
|---|---|---|---|---|---|---|
| CTRL | 55131646 | 8.32G | 8175797161 | 7909623759 | 0.982092 | 0.950119 |
| CTRL | 55062078 | 8.31G | 8162691339 | 7896684036 | 0.981757 | 0.949763 |
| CTRL | 52675780 | 7.95G | 7804805958 | 7540668087 | 0.981238 | 0.94803 |
| CTRL | 40597370 | 6.13G | 6014753285 | 5806269100 | 0.981167 | 0.947158 |
| single-HW | 61595146 | 9.30G | 9124829359 | 8821942088 | 0.981073 | 0.948507 |
| single-HW | 54216000 | 8.19G | 8028603207 | 7755393741 | 0.980699 | 0.947326 |
| single-HW | 62794730 | 9.48G | 9297031238 | 8974088404 | 0.980492 | 0.946434 |
| single-HW | 50122560 | 7.57G | 7395558427 | 7099007520 | 0.977149 | 0.937967 |
| double-HW | 53121568 | 8.02G | 7866019132 | 7595466366 | 0.980634 | 0.946905 |
| double-HW | 46533642 | 7.03G | 6898052587 | 6669896982 | 0.981708 | 0.949238 |
| double-HW | 41627856 | 6.29G | 6173490192 | 5973274034 | 0.982132 | 0.95028 |
| double-HW | 51945556 | 7.84G | 7698463781 | 7442744964 | 0.981474 | 0.948872 |

| Clean data | Clean data bas | Clean data q20 b | Clean data q30 b | Clean data q20 r |
|---|---|---|---|---|
| 53241096 | 7.99G | 7895753142 | 7680015264 | 0.987587 |
| 53615996 | 8.05G | 7948667118 | 7725396707 | 0.987091 |
| 51645796 | 7.76G | 7653948043 | 7425773451 | 0.986333 |
| 39490432 | 5.94G | 5858333236 | 5681212384 | 0.986393 |
| 59193844 | 8.89G | 8780005437 | 8539081467 | 0.987345 |
| 52272484 | 7.85G | 7749483402 | 7529649773 | 0.98701 |
| 59557566 | 8.94G | 8829150599 | 8579335335 | 0.987071 |
| 45355720 | 6.82G | 6727971887 | 6521502679 | 0.986161 |
| 51145366 | 7.69G | 7584107251 | 7364783518 | 0.986707 |
| 45226222 | 6.80G | 6718179950 | 6530821697 | 0.987416 |
| 40976014 | 6.15G | 6074199360 | 5899693562 | 0.987008 |
| 50954728 | 7.66G | 7553406206 | 7331868575 | 0.986613 |

| Clean data q30 ra | Clean data GC content |
| --- | --- |
| 0.960602 | 48.53% |
| 0.959365 | 49.67% |
| 0.956929 | 49.62% |
| 0.95657 | 49.16% |
| 0.960252 | 48.24% |
| 0.959011 | 48.24% |
| 0.959142 | 48.67% |
| 0.955897 | 47.90% |
| 0.958173 | 49.68% |
| 0.959879 | 48.86% |
| 0.958652 | 48.35% |
| 0.957677 | 49.45% |
