# Supplementary material for: Liver Antioxidant, Transcriptomic and Metabolomic Responses to Heatwaves in an Aquatic Turtle Species, Pelodiscus sinensis
Source: Animals (Basel). 2026 Jun 17;16(12):1870. doi: 10.3390/ani16121870 (PMC13295678; doi:10.3390/ani16121870)
Supplement: Supplementary file 1 [file animals-16-01870-s001.zip › Supplementary Table S2.pdf]

Single HW *vs* CTRL

Gene ID	Gene name	Log_2FC	*P* value	*P* adj	level
gene-COL14A1	COL14A1	-3.79	6.23E-10	8.99E-06	Decreased
gene-FNDC1	FNDC1	-3.23	9.22E-10	8.99E-06	Decreased
gene-LOC112546	LOC112546115	-6.67	3.83E-09	1.91E-05	Decreased
gene-FCER1G	FCER1G	-3.39	3.93E-09	1.91E-05	Decreased
gene-OGN	OGN	-5.56	8.23E-09	3.18E-05	Decreased
gene-LOC102454	LOC102454405	-20.22	1.11E-08	3.18E-05	Decreased
gene-MYL1	MYL1	-21.14	1.19E-08	3.18E-05	Decreased
gene-HSPH1	HSPH1	3.39	1.31E-08	3.18E-05	Increased
gene-NID2	NID2	-2.86	1.63E-08	3.51E-05	Decreased
gene-HTRA3	HTRA3	-11.14	1.80E-08	3.51E-05	Decreased
gene-ANXA1	ANXA1	-2.64	1.99E-08	3.53E-05	Decreased
gene-MYOZ1	MYOZ1	-18.83	2.38E-08	3.87E-05	Decreased
gene-CASQ1	CASQ1	-19.52	2.66E-08	3.94E-05	Decreased
gene-CDH11	CDH11	-2.55	2.87E-08	3.94E-05	Decreased
gene-DNAJA4	DNAJA4	3.24	3.03E-08	3.94E-05	Increased
gene-MYBPC1	MYBPC1	-19.62	4.28E-08	5.21E-05	Decreased
gene-LDB3	LDB3	-18.95	5.79E-08	6.14E-05	Decreased
gene-ACTA1	ACTA1	-14.30	5.95E-08	6.14E-05	Decreased
gene-FGL2	FGL2	-3.80	5.99E-08	6.14E-05	Decreased
gene-SEMA3C	SEMA3C	-5.27	6.41E-08	6.25E-05	Decreased
gene-MYOM2	MYOM2	-18.47	7.41E-08	6.67E-05	Decreased
gene-NHP2	NHP2	2.15	7.53E-08	6.67E-05	Increased
gene-PVALB	PVALB	-17.49	1.14E-07	8.86E-05	Decreased
gene-LOC102446	LOC102446383	-17.35	1.15E-07	8.86E-05	Decreased
gene-MID1IP1	MID1IP1	-3.13	1.16E-07	8.86E-05	Decreased
gene-MYL2	MYL2	-17.84	1.18E-07	8.86E-05	Decreased
gene-COL5A1	COL5A1	-3.24	1.26E-07	9.08E-05	Decreased
gene-COL1A2	COL1A2	-3.42	1.81E-07	1.23E-04	Decreased
gene-LOC102462	LOC102462333	-2.81	1.83E-07	1.23E-04	Decreased
gene-SLC43A2	SLC43A2	-5.80	2.38E-07	1.55E-04	Decreased
gene-FHL1	FHL1	-10.28	2.46E-07	1.55E-04	Decreased
gene-LOC102447	LOC102447978	-3.73	2.58E-07	1.57E-04	Decreased
gene-ENO3	ENO3	-14.89	2.84E-07	1.68E-04	Decreased
gene-ACTN3	ACTN3	-13.27	3.41E-07	1.95E-04	Decreased
gene-LOC102445	LOC102445983	-16.21	4.19E-07	2.29E-04	Decreased
gene-CSF3R	CSF3R	-4.53	4.23E-07	2.29E-04	Decreased
gene-SERPINH1	SERPINH1	2.46	4.36E-07	2.30E-04	Increased
gene-AQP4	AQP4	-12.93	4.76E-07	2.44E-04	Decreased
gene-RSRP1	RSRP1	2.60	5.56E-07	2.71E-04	Increased
gene-LOC102450	LOC102450121	-16.26	5.56E-07	2.71E-04	Decreased
gene-LOC102462	LOC102462847	-5.77	6.62E-07	3.15E-04	Decreased
gene-FSTL1	FSTL1	-3.37	8.73E-07	3.93E-04	Decreased
gene-LOC102445	LOC102445123	-8.56	8.79E-07	3.93E-04	Decreased
gene-TNNI2	TNNI2	-16.07	9.32E-07	3.93E-04	Decreased
gene-PPP1R3A	PPP1R3A	-15.58	9.41E-07	3.93E-04	Decreased
gene-LCP1	LCP1	-3.34	9.44E-07	3.93E-04	Decreased
gene-ZNF423	ZNF423	-3.79	9.47E-07	3.93E-04	Decreased
gene-CAV1	CAV1	-2.69	1.01E-06	4.08E-04	Decreased
gene-LOC102463	LOC102463217	-6.41	1.03E-06	4.08E-04	Decreased
gene-FHOD3	FHOD3	-8.78	1.07E-06	4.09E-04	Decreased
gene-LOC102451	LOC102451731	-15.91	1.07E-06	4.09E-04	Decreased
gene-XIRP2	XIRP2	-8.88	1.10E-06	4.09E-04	Decreased

gene-TRDN	TRDN	-17.65	1.11E-06	4.09E-04	Decreased
gene-SYPL2	SYPL2	-9.31	1.23E-06	4.43E-04	Decreased
gene-COL5A2	COL5A2	-2.69	1.26E-06	4.43E-04	Decreased
gene-MFAP2	MFAP2	-3.60	1.27E-06	4.43E-04	Decreased
gene-TIMP3	TIMP3	-3.24	1.37E-06	4.60E-04	Decreased
gene-LOC102463	LOC102463089	-5.21	1.40E-06	4.60E-04	Decreased
gene-COL4A2	COL4A2	-4.67	1.40E-06	4.60E-04	Decreased
gene-CDH13	CDH13	-7.14	1.44E-06	4.60E-04	Decreased
gene-THBS2	THBS2	-3.33	1.44E-06	4.60E-04	Decreased
gene-FBN1	FBN1	-3.16	1.63E-06	4.98E-04	Decreased
gene-LOC102449	LOC102449655	-5.23	1.63E-06	4.98E-04	Decreased
gene-TNNT3	TNNT3	-11.69	1.65E-06	4.98E-04	Decreased
gene-LOC112545	LOC112545390	-2.24	1.67E-06	4.98E-04	Decreased
gene-HSP90AA1	HSP90AA1	2.88	1.69E-06	4.98E-04	Increased
gene-CAPN3	CAPN3	-8.27	1.78E-06	5.19E-04	Decreased
gene-COL1A1	COL1A1	-3.18	1.97E-06	5.65E-04	Decreased
gene-ISM1	ISM1	-9.65	2.01E-06	5.69E-04	Decreased
gene-F13A1	F13A1	-3.19	2.06E-06	5.73E-04	Decreased
gene-OXCT1	OXCT1	-5.50	2.14E-06	5.84E-04	Decreased
gene-DST	DST	-4.78	2.16E-06	5.84E-04	Decreased
gene-PGM5	PGM5	-5.11	2.49E-06	6.64E-04	Decreased
gene-LOC102457	LOC102457840	-11.43	2.58E-06	6.70E-04	Decreased
gene-PDLIM3	PDLIM3	-6.86	2.58E-06	6.70E-04	Decreased
gene-FHL3	FHL3	-6.55	2.79E-06	7.14E-04	Decreased
gene-CPE	CPE	-2.87	2.84E-06	7.20E-04	Decreased
gene-LOC102443	LOC102443642	-7.42	2.95E-06	7.36E-04	Decreased
gene-EEF1A2	EEF1A2	-14.39	3.00E-06	7.40E-04	Decreased
gene-ASB2	ASB2	-9.85	3.16E-06	7.70E-04	Decreased
gene-PDLIM4	PDLIM4	-3.17	3.37E-06	8.11E-04	Decreased
gene-PLXNA2	PLXNA2	-6.70	3.49E-06	8.30E-04	Decreased
gene-LOC102453	LOC102453880	-7.42	3.62E-06	8.44E-04	Decreased
gene-TGFBR3	TGFBR3	-2.37	3.64E-06	8.44E-04	Decreased
gene-HK1	HK1	-5.17	3.82E-06	8.69E-04	Decreased
gene-MEGF10	MEGF10	-11.14	3.87E-06	8.69E-04	Decreased
gene-FZD4	FZD4	-2.47	3.92E-06	8.69E-04	Decreased
gene-LOC102463	LOC102463153	-3.73	3.94E-06	8.69E-04	Decreased
gene-TNNC1	TNNC1	-18.29	3.97E-06	8.69E-04	Decreased
gene-FSCN1	FSCN1	-4.10	4.05E-06	8.78E-04	Decreased
gene-LOC102453	LOC102453787	-12.84	4.29E-06	9.17E-04	Decreased
gene-CELF2	CELF2	-4.67	4.36E-06	9.17E-04	Decreased
gene-CD99	CD99	-2.90	4.38E-06	9.17E-04	Decreased
gene-LOC102461	LOC102461260	-1.94	4.67E-06	9.36E-04	Decreased
gene-OBSCN	OBSCN	-16.16	4.69E-06	9.36E-04	Decreased
gene-FRZB	FRZB	-2.69	4.70E-06	9.36E-04	Decreased
gene-TUBB6	TUBB6	-3.46	4.71E-06	9.36E-04	Decreased
gene-CKMT2	CKMT2	-16.47	4.72E-06	9.36E-04	Decreased
gene-HSPB1	HSPB1	-8.30	4.75E-06	9.36E-04	Decreased
gene-LUM	LUM	-4.05	4.87E-06	9.48E-04	Decreased
gene-SLIT3	SLIT3	-2.21	4.91E-06	9.48E-04	Decreased
gene-PPP3CA	PPP3CA	-3.41	5.24E-06	9.99E-04	Decreased
gene-OLFML2B	OLFML2B	-3.71	5.29E-06	9.99E-04	Decreased
gene-ADAMTSL1	ADAMTSL1	-5.06	5.34E-06	9.99E-04	Decreased
gene-SHISA4	SHISA4	-5.32	5.38E-06	9.99E-04	Decreased
gene-MYL3	MYL3	-11.44	5.49E-06	0.001004	Decreased

gene-NEB	NEB	-15.73	5.51E-06	0.001004	Decreased
gene-RIPOR2	RIPOR2	-4.40	5.60E-06	0.00101	Decreased
gene-CAVIN1	CAVIN1	-3.29	5.88E-06	0.001044	Decreased
gene-KCNJ11	KCNJ11	-12.98	5.89E-06	0.001044	Decreased
gene-CLEC3B	CLEC3B	-5.36	6.14E-06	0.001078	Decreased
gene-MATN2	MATN2	-3.67	6.30E-06	0.001096	Decreased
gene-KLHL31	KLHL31	-15.35	6.42E-06	0.0011	Decreased
gene-LRRC20	LRRC20	-4.82	6.45E-06	0.0011	Decreased
gene-AMPD1	AMPD1	-14.41	6.49E-06	0.0011	Decreased
gene-ENG	ENG	-2.11	6.54E-06	0.0011	Decreased
gene-CAV3	CAV3	-15.75	6.64E-06	0.001106	Decreased
gene-LOC106732	LOC106732479	-8.07	6.73E-06	0.001107	Decreased
gene-LOC102458	LOC102458182	-4.83	6.80E-06	0.001107	Decreased
gene-TTN	TTN	-11.12	6.83E-06	0.001107	Decreased
gene-LOC102450	LOC102450310	-5.38	6.87E-06	0.001107	Decreased
gene-ALG12	ALG12	1.87	6.96E-06	0.001112	Increased
gene-TPBG	TPBG	-11.30	7.08E-06	0.001123	Decreased
gene-LOC112544	LOC112544376	-8.05	7.15E-06	0.001124	Decreased
gene-AK1	AK1	-6.38	7.29E-06	0.001137	Decreased
gene-TENM3	TENM3	-2.84	7.37E-06	0.001138	Decreased
gene-PXDN	PXDN	-2.15	7.41E-06	0.001138	Decreased
gene-LIMCH1	LIMCH1	-5.07	7.63E-06	0.001162	Decreased
gene-JPH2	JPH2	-5.92	7.96E-06	0.001203	Decreased
gene-NRAP	NRAP	-7.23	8.04E-06	0.001205	Decreased
gene-LOC102454	LOC102454894	-2.49	8.16E-06	0.001207	Decreased
gene-SRL	SRL	-16.09	8.17E-06	0.001207	Decreased
gene-MYOT	MYOT	-9.48	8.96E-06	0.001313	Decreased
gene-SYNPO2	SYNPO2	-6.07	9.07E-06	0.001319	Decreased
gene-LIMD2	LIMD2	-2.77	9.27E-06	0.001339	Decreased
gene-ZYX	ZYX	-2.50	1.01E-05	0.001436	Decreased
gene-CAVIN2	CAVIN2	-2.16	1.02E-05	0.001436	Decreased
gene-LOC102455	LOC102455168	2.40	1.02E-05	0.001436	Increased
gene-MXRA5	MXRA5	-7.73	1.02E-05	0.001436	Decreased
gene-LOC102462	LOC102462085	-7.21	1.04E-05	0.001446	Decreased
gene-CAMK2A	CAMK2A	-9.95	1.05E-05	0.001446	Decreased
gene-GSN	GSN	-3.67	1.06E-05	0.001446	Decreased
gene-ANO6	ANO6	-4.62	1.07E-05	0.001446	Decreased
gene-MFAP5	MFAP5	-6.10	1.08E-05	0.001446	Decreased
gene-DNAJC10	DNAJC10	2.26	1.08E-05	0.001446	Increased
gene-SGCA	SGCA	-9.29	1.09E-05	0.001458	Decreased
gene-ALPK3	ALPK3	-6.70	1.12E-05	0.001475	Decreased
gene-CEP85L	CEP85L	-5.78	1.13E-05	0.001475	Decreased
gene-LOC102462	LOC102462791	1.72	1.13E-05	0.001475	Increased
gene-KIAA1143	KIAA1143	-1.84	1.14E-05	0.001475	Decreased
gene-MB	MB	-14.92	1.14E-05	0.001475	Decreased
gene-LOC102448	LOC102448341	-9.33	1.15E-05	0.001475	Decreased
gene-CUNH1orf1	CUNH1orf198	-2.07	1.17E-05	0.001488	Decreased
gene-NTNG2	NTNG2	-3.46	1.18E-05	0.001499	Decreased
gene-SLN	SLN	-11.45	1.21E-05	0.001523	Decreased
gene-MMP2	MMP2	-2.13	1.22E-05	0.001523	Decreased
gene-NEXN	NEXN	-6.99	1.24E-05	0.001533	Decreased
gene-LOC102458	LOC102458660	-13.07	1.24E-05	0.001533	Decreased
gene-LOC102445	LOC102445544	-6.60	1.26E-05	0.001549	Decreased
gene-RBM20	RBM20	-12.41	1.29E-05	0.001567	Decreased

gene-ASB14	ASB14	-12.30	1.30E-05	0.001571	Decreased
gene-LOC102456	LOC102456597	-6.61	1.31E-05	0.001571	Decreased
gene-LOC102444	LOC102444471	-8.85	1.31E-05	0.001571	Decreased
gene-HSPB8	HSPB8	-5.08	1.37E-05	0.001624	Decreased
gene-EPDR1	EPDR1	-4.99	1.37E-05	0.001624	Decreased
gene-CACNA2D1	CACNA2D1	-6.56	1.47E-05	0.001704	Decreased
gene-SGCD	SGCD	-7.06	1.48E-05	0.001704	Decreased
gene-EMP3	EMP3	-2.42	1.48E-05	0.001704	Decreased
gene-SVIL	SVIL	-5.07	1.48E-05	0.001704	Decreased
gene-LOC102448	LOC102448706	-4.48	1.49E-05	0.001704	Decreased
gene-LOC106732	LOC106732620	-2.10	1.50E-05	0.001704	Decreased
gene-USP13	USP13	-9.43	1.51E-05	0.001704	Decreased
gene-CSF1R	CSF1R	-3.00	1.51E-05	0.001704	Decreased
gene-ITGA8	ITGA8	-2.80	1.54E-05	0.001725	Decreased
gene-GATM	GATM	-8.17	1.59E-05	0.001769	Decreased
gene-LOC102460	LOC102460301	-9.82	1.61E-05	0.001773	Decreased
gene-ZMIZ1	ZMIZ1	-2.77	1.62E-05	0.001773	Decreased
gene-LOC102463	LOC102463012	-7.22	1.63E-05	0.001773	Decreased
gene-CAMK4	CAMK4	-7.69	1.63E-05	0.001773	Decreased
gene-MYLK2	MYLK2	-6.70	1.65E-05	0.001785	Decreased
gene-COLEC12	COLEC12	-4.37	1.74E-05	0.001876	Decreased
gene-ID2	ID2	-1.67	1.77E-05	0.001901	Decreased
gene-MMRN2	MMRN2	-2.92	1.81E-05	0.001926	Decreased
gene-ANGPTL2	ANGPTL2	-3.34	1.85E-05	0.001957	Decreased
gene-ECE1	ECE1	-1.84	1.86E-05	0.001957	Decreased
gene-JARID2	JARID2	2.04	1.87E-05	0.001957	Increased
gene-COL11A1	COL11A1	-5.40	1.88E-05	0.00196	Decreased
gene-PRKCQ	PRKCQ	-8.57	1.91E-05	0.00198	Decreased
gene-PAQR6	PAQR6	-2.86	1.92E-05	0.00198	Decreased
gene-ADIPOQ	ADIPOQ	-9.69	1.94E-05	0.001992	Decreased
gene-LOC102446	LOC102446585	-4.61	1.98E-05	0.002018	Decreased
gene-LOC102462	LOC102462198	-2.37	2.00E-05	0.002018	Decreased
gene-LOC102459	LOC102459215	-4.19	2.02E-05	0.002018	Decreased
gene-RGMA	RGMA	-4.97	2.02E-05	0.002018	Decreased
gene-PLP1	PLP1	-6.28	2.03E-05	0.002018	Decreased
gene-OAT	OAT	-2.74	2.03E-05	0.002018	Decreased
gene-LOC102450	LOC102450688	-12.46	2.06E-05	0.002041	Decreased
gene-LOC102462	LOC102462326	-14.49	2.08E-05	0.002041	Decreased
gene-BCHE	BCHE	-5.38	2.09E-05	0.002041	Decreased
gene-LOC102443	LOC102443906	-7.54	2.09E-05	0.002041	Decreased
gene-VLDLR	VLDLR	-4.45	2.12E-05	0.002045	Decreased
gene-LOC102460	LOC102460859	-7.03	2.12E-05	0.002045	Decreased
gene-EDNRA	EDNRA	-1.89	2.17E-05	0.002078	Decreased
gene-LOC102461	LOC102461556	-4.42	2.18E-05	0.002078	Decreased
gene-LAPTM5	LAPTM5	-2.20	2.19E-05	0.002078	Decreased
gene-ITIH5	ITIH5	-6.19	2.20E-05	0.002083	Decreased
gene-COL4A1	COL4A1	-3.78	2.21E-05	0.002084	Decreased
gene-IGSF3	IGSF3	-2.29	2.24E-05	0.002096	Decreased
gene-PTPRM	PTPRM	-2.61	2.25E-05	0.002096	Decreased
gene-LOXL2	LOXL2	-3.17	2.26E-05	0.002096	Decreased
gene-LOC106732	LOC106732455	-5.87	2.33E-05	0.002153	Decreased
gene-NT5C1A	NT5C1A	-15.07	2.38E-05	0.002184	Decreased
gene-MYLK4	MYLK4	-7.45	2.40E-05	0.002184	Decreased
gene-TMOD1	TMOD1	-6.94	2.40E-05	0.002184	Decreased

gene-LOC102456	LOC102456381	-2.10	2.41E-05	0.002186	Decreased
gene-FBN2	FBN2	-5.11	2.45E-05	0.002202	Decreased
gene-CXCL12	CXCL12	-1.98	2.45E-05	0.002202	Decreased
gene-KCNJ2	KCNJ2	-9.08	2.52E-05	0.00225	Decreased
gene-HYOU1	HYOU1	2.47	2.53E-05	0.00225	Increased
gene-LMOD2	LMOD2	-15.33	2.55E-05	0.002251	Decreased
gene-TPMT	TPMT	1.88	2.56E-05	0.002251	Increased
gene-FAM78A	FAM78A	-5.99	2.56E-05	0.002251	Decreased
gene-MAK16	MAK16	1.72	2.58E-05	0.002254	Increased
gene-DOCK2	DOCK2	-2.63	2.61E-05	0.002274	Decreased
gene-LOC102462	LOC102462213	-2.99	2.62E-05	0.002274	Decreased
gene-EN2	EN2	-8.02	2.68E-05	0.002312	Decreased
gene-HOXA7	HOXA7	-11.05	2.71E-05	0.002329	Decreased
gene-LOC102459	LOC102459781	-13.56	2.76E-05	0.002358	Decreased
gene-MGP	MGP	-3.08	2.79E-05	0.002358	Decreased
gene-LOC102456	LOC102456107	-4.25	2.80E-05	0.002358	Decreased
gene-SGCG	SGCG	-14.49	2.82E-05	0.002358	Decreased
gene-TNFAIP8L2	TNFAIP8L2	-2.98	2.84E-05	0.002358	Decreased
gene-LOC102455	LOC102455200	-7.48	2.84E-05	0.002358	Decreased
gene-LOC102459	LOC102459228	7.34	2.84E-05	0.002358	Increased
gene-WIPF3	WIPF3	-8.40	2.87E-05	0.002358	Decreased
gene-GSE1	GSE1	-1.61	2.87E-05	0.002358	Decreased
gene-LOC102443	LOC102443664	-15.26	2.89E-05	0.002358	Decreased
gene-FLT1	FLT1	-2.62	2.90E-05	0.002358	Decreased
gene-RELT	RELT	-11.03	2.90E-05	0.002358	Decreased
gene-LOC102461	LOC102461635	-2.06	2.92E-05	0.002358	Decreased
gene-ADPRHL1	ADPRHL1	-14.70	2.93E-05	0.002358	Decreased
gene-DLK1	DLK1	-14.22	2.93E-05	0.002358	Decreased
gene-LOC102455	LOC102455817	-14.59	2.95E-05	0.002364	Decreased
gene-LOC102452	LOC102452319	-14.25	2.98E-05	0.00238	Decreased
gene-SEMA6C	SEMA6C	-2.74	3.02E-05	0.002403	Decreased
gene-HACD1	HACD1	-7.33	3.05E-05	0.002403	Decreased
gene-CRIM1	CRIM1	-2.68	3.06E-05	0.002403	Decreased
gene-CUNH4orf5	CUNH4orf54	-14.33	3.06E-05	0.002403	Decreased
gene-LOC102447	LOC102447219	-14.14	3.07E-05	0.002403	Decreased
gene-EMILIN2	EMILIN2	-2.44	3.10E-05	0.002414	Decreased
gene-LOC102450	LOC102450075	-2.52	3.11E-05	0.002414	Decreased
gene-TUBA8	TUBA8	-14.64	3.16E-05	0.002439	Decreased
gene-SMPX	SMPX	-10.06	3.17E-05	0.002439	Decreased
gene-LOC102456	LOC102456587	-7.53	3.18E-05	0.002439	Decreased
gene-LOC102459	LOC102459610	-15.65	3.21E-05	0.002456	Decreased
gene-HCK	HCK	-1.89	3.23E-05	0.002456	Decreased
gene-ARL15	ARL15	-1.80	3.24E-05	0.002456	Decreased
gene-FAM129A	FAM129A	-3.26	3.28E-05	0.00247	Decreased
gene-PLEKHG4B	PLEKHG4B	-3.58	3.28E-05	0.00247	Decreased
gene-ANXA2	ANXA2	-2.21	3.33E-05	0.002499	Decreased
gene-FNDC5	FNDC5	-6.89	3.39E-05	0.002529	Decreased
gene-PTPRB	PTPRB	-1.77	3.41E-05	0.002538	Decreased
gene-LOC106732	LOC106732749	-5.98	3.43E-05	0.002545	Decreased
gene-CSDC2	CSDC2	-2.67	3.47E-05	0.002562	Decreased
gene-MYL10	MYL10	-12.63	3.60E-05	0.002651	Decreased
gene-LOC102446	LOC102446694	-7.22	3.62E-05	0.002655	Decreased
gene-MPZ	MPZ	-7.95	3.69E-05	0.002694	Decreased
gene-PRLR	PRLR	-3.54	3.70E-05	0.002694	Decreased

gene-HSPB7	HSPB7	-8.31	3.72E-05	0.002697	Decreased
gene-ANO5	ANO5	-3.15	3.76E-05	0.002716	Decreased
gene-ART1	ART1	-12.58	3.78E-05	0.002719	Decreased
gene-TRIM36	TRIM36	-4.69	3.85E-05	0.002756	Decreased
gene-PDGFD	PDGFD	-5.07	3.90E-05	0.002782	Decreased
gene-LOC102457	LOC102457799	-4.98	3.92E-05	0.002788	Decreased
gene-MSRB3	MSRB3	-2.70	3.98E-05	0.00282	Decreased
gene-FILIP1L	FILIP1L	-3.52	3.99E-05	0.00282	Decreased
gene-LOC102447	LOC102447192	-3.15	4.02E-05	0.002826	Decreased
gene-LOC102448	LOC102448738	-13.06	4.03E-05	0.002826	Decreased
gene-LOC102454	LOC102454345	-3.39	4.08E-05	0.00285	Decreased
gene-CUNH10orf	CUNH10orf71	-11.12	4.13E-05	0.002874	Decreased
gene-RGS16	RGS16	-3.00	4.17E-05	0.002877	Decreased
gene-PKIA	PKIA	-3.94	4.18E-05	0.002877	Decreased
gene-LFNG	LFNG	-1.79	4.18E-05	0.002877	Decreased
gene-LPIN1	LPIN1	-1.99	4.20E-05	0.002885	Decreased
gene-PDZRN3	PDZRN3	-5.08	4.29E-05	0.002929	Decreased
gene-LOC102462	LOC102462153	-3.15	4.30E-05	0.002929	Decreased
gene-EYA4	EYA4	-13.06	4.39E-05	0.002976	Decreased
gene-LEXM	LEXM	3.44	4.40E-05	0.002976	Increased
gene-LOC102446	LOC102446023	-3.39	4.46E-05	0.003009	Decreased
gene-RNF207	RNF207	-6.75	4.51E-05	0.003028	Decreased
gene-LOC102446	LOC102446703	-4.19	4.61E-05	0.00309	Decreased
gene-LOC102460	LOC102460847	-12.46	4.63E-05	0.003094	Decreased
gene-GADL1	GADL1	-9.50	4.69E-05	0.003109	Decreased
gene-CAV2	CAV2	-2.52	4.70E-05	0.003109	Decreased
gene-LOC102452	LOC102452779	-1.93	4.71E-05	0.003109	Decreased
gene-LOC102446	LOC102446622	-12.64	4.75E-05	0.003126	Decreased
gene-LOC102461	LOC102461925	-2.75	4.86E-05	0.003188	Decreased
gene-CLMP	CLMP	-3.15	4.91E-05	0.003212	Decreased
gene-FLRT2	FLRT2	-3.57	4.94E-05	0.003218	Decreased
gene-GLDN	GLDN	-10.46	5.08E-05	0.003298	Decreased
gene-A4GALT	A4GALT	-5.53	5.17E-05	0.003324	Decreased
gene-PFKM	PFKM	-5.10	5.19E-05	0.003324	Decreased
gene-MME	MME	-6.51	5.20E-05	0.003324	Decreased
gene-PLEKHO2	PLEKHO2	-3.10	5.20E-05	0.003324	Decreased
gene-PITX1	PITX1	-12.78	5.20E-05	0.003324	Decreased
gene-LRRN1	LRRN1	-6.62	5.28E-05	0.00335	Decreased
gene-LOC102447	LOC102447514	-12.90	5.28E-05	0.00335	Decreased
gene-RAB7B	RAB7B	-2.65	5.32E-05	0.00337	Decreased
gene-PYGM	PYGM	-6.09	5.37E-05	0.003379	Decreased
gene-PECAM1	PECAM1	-1.84	5.38E-05	0.003379	Decreased
gene-BHLHA15	BHLHA15	3.20	5.40E-05	0.003379	Increased
gene-TECRL	TECRL	-12.72	5.41E-05	0.003379	Decreased
gene-TM6SF1	TM6SF1	-5.23	5.44E-05	0.003381	Decreased
gene-HSPA4L	HSPA4L	1.90	5.45E-05	0.003381	Increased
gene-MARCKS	MARCKS	-2.07	5.48E-05	0.003393	Decreased
gene-KY	KY	-12.38	5.55E-05	0.00342	Decreased
gene-PITX3	PITX3	-12.09	5.57E-05	0.00342	Decreased
gene-DKK3	DKK3	-2.64	5.58E-05	0.00342	Decreased
gene-CIT	CIT	-2.44	5.80E-05	0.003542	Decreased
gene-KLF5	KLF5	-4.28	5.81E-05	0.003542	Decreased
gene-FRY	FRY	-3.37	5.97E-05	0.003628	Decreased
gene-LOC106731	LOC106731549	-4.17	6.06E-05	0.00366	Decreased

gene-LOC102455	LOC102455319	-9.96	6.08E-05	0.00366	Decreased
gene-PER1	PER1	-1.97	6.08E-05	0.00366	Decreased
gene-SLC25A12	SLC25A12	-5.70	6.34E-05	0.003791	Decreased
gene-ANKRD23	ANKRD23	-6.37	6.34E-05	0.003791	Decreased
gene-MANF	MANF	2.33	6.40E-05	0.003815	Increased
gene-CHRNE	CHRNE	-5.63	6.46E-05	0.003842	Decreased
gene-B4GALNT4	B4GALNT4	-3.93	6.51E-05	0.003858	Decreased
gene-PCP4L1	PCP4L1	1.86	6.54E-05	0.003861	Increased
gene-KLHDC8A	KLHDC8A	-6.61	6.59E-05	0.003876	Decreased
gene-ME3	ME3	-3.54	6.60E-05	0.003876	Decreased
gene-LOC112546	LOC112546643	-7.87	6.84E-05	0.004004	Decreased
gene-LOC102451	LOC102451133	-2.94	6.92E-05	0.004038	Decreased
gene-SYNM	SYNM	-4.33	7.01E-05	0.004076	Decreased
gene-MAP3K15	MAP3K15	-4.21	7.04E-05	0.004084	Decreased
gene-DPYSL4	DPYSL4	-5.42	7.11E-05	0.004113	Decreased
gene-NEU3	NEU3	-4.65	7.22E-05	0.004164	Decreased
gene-LOC102446	LOC102446550	-8.38	7.30E-05	0.004196	Decreased
gene-LOC102458	LOC102458926	-10.74	7.38E-05	0.004208	Decreased
gene-MAFB	MAFB	-2.34	7.41E-05	0.004208	Decreased
gene-ST3GAL1	ST3GAL1	-2.00	7.41E-05	0.004208	Decreased
gene-LOC102459	LOC102459454	-3.62	7.42E-05	0.004208	Decreased
gene-DTNA	DTNA	-6.49	7.45E-05	0.004208	Decreased
gene-LOC102458	LOC102458809	-1.74	7.47E-05	0.004208	Decreased
gene-CSPG4	CSPG4	-4.71	7.48E-05	0.004208	Decreased
gene-CD109	CD109	-3.10	7.49E-05	0.004208	Decreased
gene-FBXL22	FBXL22	-6.88	7.56E-05	0.004226	Decreased
gene-MYADML2	MYADML2	-12.16	7.58E-05	0.004226	Decreased
gene-LOC112543	LOC112543701	-8.73	7.59E-05	0.004226	Decreased
gene-RBM24	RBM24	-5.19	7.79E-05	0.004325	Decreased
gene-SEMA5A	SEMA5A	-4.23	7.82E-05	0.00433	Decreased
gene-SPON2	SPON2	-6.66	7.91E-05	0.004369	Decreased
gene-SIX2	SIX2	-12.04	7.94E-05	0.004369	Decreased
gene-SUMF2	SUMF2	1.93	7.97E-05	0.004375	Increased
gene-PDGFRA	PDGFRA	-2.11	8.00E-05	0.00438	Decreased
gene-DUSP27	DUSP27	-8.96	8.06E-05	0.004399	Decreased
gene-LOC102459	LOC102459272	2.49	8.22E-05	0.004476	Increased
gene-TRIM63	TRIM63	-6.29	8.32E-05	0.004516	Decreased
gene-EARS2	EARS2	1.63	8.34E-05	0.004516	Increased
gene-LOC106732	LOC106732484	-10.43	8.51E-05	0.004591	Decreased
gene-BPGM	BPGM	-2.77	8.53E-05	0.004591	Decreased
gene-ARHGDIB	ARHGDIB	-1.68	8.55E-05	0.004591	Decreased
gene-MMP23B	MMP23B	-4.36	8.61E-05	0.004609	Decreased
gene-PLXDC2	PLXDC2	-5.50	8.78E-05	0.004691	Decreased
gene-PARVB	PARVB	-2.96	8.92E-05	0.004749	Decreased
gene-LOC112544	LOC112544805	7.92	9.11E-05	0.004837	Increased
gene-LOC102456	LOC102456632	-10.52	9.28E-05	0.004908	Decreased
gene-ART5	ART5	-1.77	9.29E-05	0.004908	Decreased
gene-LOC106731	LOC106731550	-6.61	9.34E-05	0.004919	Decreased
gene-ADAMTS19	ADAMTS19	-4.60	9.40E-05	0.004939	Decreased
gene-HPCA	HPCA	-7.25	9.47E-05	0.004949	Decreased
gene-PKNOX2	PKNOX2	-3.86	9.47E-05	0.004949	Decreased
gene-LOC102452	LOC102452756	-4.59	9.50E-05	0.004952	Decreased
gene-KBTBD11	KBTBD11	-4.48	9.54E-05	0.004958	Decreased
gene-JMJD8	JMJD8	1.97	9.66E-05	0.005009	Increased

gene-LOC102447	LOC102447789	-10.54	9.70E-05	0.005013	Decreased
gene-BASP1	BASP1	-1.89	9.74E-05	0.00502	Decreased
gene-TGFBR2	TGFBR2	-1.54	9.80E-05	0.00504	Decreased
gene-TNNI1	TNNI1	-16.39	9.87E-05	0.00506	Decreased
gene-RPL3L	RPL3L	-8.33	1.02E-04	0.005214	Decreased
gene-HSPB6	HSPB6	-6.68	1.02E-04	0.005224	Decreased
gene-POLR2H	POLR2H	1.58	1.03E-04	0.005234	Increased
gene-GCA	GCA	-1.81	1.03E-04	0.005246	Decreased
gene-LOC102459	LOC102459178	-12.06	1.04E-04	0.005247	Decreased
gene-ME1	ME1	-2.85	1.04E-04	0.005248	Decreased
gene-POSTN	POSTN	-2.56	1.04E-04	0.005248	Decreased
gene-LOC102452	LOC102452072	-3.24	1.05E-04	0.005276	Decreased
gene-BIN1	BIN1	-5.18	1.05E-04	0.005276	Decreased
gene-MCU	MCU	-3.79	1.06E-04	0.005276	Decreased
gene-HSPA5	HSPA5	2.39	1.06E-04	0.005276	Increased
gene-LOC102454	LOC102454394	-2.12	1.06E-04	0.005276	Decreased
gene-ITGAV	ITGAV	-1.82	1.06E-04	0.005276	Decreased
gene-LOC102458	LOC102458609	-2.15	1.07E-04	0.005276	Decreased
gene-MEF2C	MEF2C	-4.22	1.07E-04	0.005278	Decreased
gene-TAAR1	TAAR1	-6.68	1.08E-04	0.0053	Decreased
gene-JAM3	JAM3	-2.65	1.08E-04	0.005314	Decreased
gene-TCAP	TCAP	-6.67	1.10E-04	0.005372	Decreased
gene-LOC102464	LOC102464074	-1.77	1.10E-04	0.005385	Decreased
gene-TXLNB	TXLNB	-14.77	1.12E-04	0.005434	Decreased
gene-LOC102463	LOC102463311	-4.85	1.13E-04	0.005476	Decreased
gene-GPRC5B	GPRC5B	-1.89	1.13E-04	0.005502	Decreased
gene-CACNG1	CACNG1	-15.26	1.14E-04	0.005515	Decreased
gene-LOC102447	LOC102447587	-7.18	1.16E-04	0.005545	Decreased
gene-LOC102455	LOC102455412	-14.49	1.16E-04	0.005545	Decreased
gene-SYNPO	SYNPO	-3.13	1.17E-04	0.005545	Decreased
gene-CRYAB	CRYAB	-11.34	1.17E-04	0.005545	Decreased
gene-LOC106733	LOC106733009	-3.21	1.17E-04	0.005545	Decreased
gene-TACC3	TACC3	-1.63	1.18E-04	0.005545	Decreased
gene-BCAT1	BCAT1	-4.29	1.18E-04	0.005545	Decreased
gene-RAB11FIP5	RAB11FIP5	-2.38	1.18E-04	0.005545	Decreased
gene-CAVIN4	CAVIN4	-6.15	1.18E-04	0.005545	Decreased
gene-P2RY12	P2RY12	-2.43	1.18E-04	0.005545	Decreased
gene-MYOM1	MYOM1	-5.04	1.18E-04	0.005545	Decreased
gene-LOC102452	LOC102452168	-8.63	1.18E-04	0.005545	Decreased
gene-DHRS7C	DHRS7C	-14.27	1.20E-04	0.0056	Decreased
gene-COL6A1	COL6A1	-2.01	1.20E-04	0.0056	Decreased
gene-CDH5	CDH5	-1.65	1.21E-04	0.005637	Decreased
gene-LOC102452	LOC102452661	-14.22	1.21E-04	0.005637	Decreased
gene-DAPP1	DAPP1	-3.38	1.22E-04	0.005643	Decreased
gene-HHATL	HHATL	-14.16	1.22E-04	0.00566	Decreased
gene-NMRK2	NMRK2	-14.15	1.23E-04	0.005671	Decreased
gene-VIT	VIT	-8.09	1.24E-04	0.005682	Decreased
gene-TBCCD1	TBCCD1	1.73	1.24E-04	0.005682	Increased
gene-LOC102449	LOC102449893	-3.12	1.24E-04	0.005682	Decreased
gene-ITGA7	ITGA7	-5.64	1.24E-04	0.005682	Decreased
gene-SLC2A4	SLC2A4	-5.73	1.26E-04	0.005747	Decreased
gene-CSRP3	CSRP3	-13.96	1.27E-04	0.005792	Decreased
gene-MIGA1	MIGA1	-2.70	1.29E-04	0.005845	Decreased
gene-PTGIS	PTGIS	-3.56	1.29E-04	0.005846	Decreased

gene-LOC102453	LOC102453489	6.66	1.30E-04	0.005851	Increased
gene-LOC106732	LOC106732469	-6.83	1.30E-04	0.005851	Decreased
gene-ADGRL4	ADGRL4	-1.75	1.30E-04	0.005854	Decreased
gene-LOC102446	LOC102446471	-3.83	1.30E-04	0.005854	Decreased
gene-KLHL40	KLHL40	-13.90	1.31E-04	0.005854	Decreased
gene-RPS19BP1	RPS19BP1	1.57	1.31E-04	0.005854	Increased
gene-CHRNB1	CHRNB1	-5.36	1.31E-04	0.005854	Decreased
gene-ADAMTS15	ADAMTS15	-3.74	1.32E-04	0.005857	Decreased
gene-SOCS3	SOCS3	-2.81	1.32E-04	0.005857	Decreased
gene-SAMD11	SAMD11	-2.17	1.33E-04	0.005873	Decreased
gene-PLCD4	PLCD4	-6.38	1.33E-04	0.005895	Decreased
gene-LOC112547	LOC112547447	-9.08	1.35E-04	0.005947	Decreased
gene-AQP1	AQP1	-3.10	1.36E-04	0.005988	Decreased
gene-SLC9A9	SLC9A9	-2.43	1.38E-04	0.006052	Decreased
gene-LAMA2	LAMA2	-5.45	1.38E-04	0.006052	Decreased
gene-FSD2	FSD2	-13.72	1.39E-04	0.006079	Decreased
gene-PRELP	PRELP	-3.98	1.40E-04	0.006092	Decreased
gene-AHNAK	AHNAK	-2.63	1.40E-04	0.006108	Decreased
gene-PKM	PKM	-4.42	1.41E-04	0.006123	Decreased
gene-TMEM109	TMEM109	-2.03	1.41E-04	0.006123	Decreased
gene-SUSD4	SUSD4	3.08	1.47E-04	0.006359	Increased
gene-LOC102448	LOC102448657	-2.76	1.48E-04	0.006359	Decreased
gene-LAPTM4B	LAPTM4B	-7.88	1.48E-04	0.006359	Decreased
gene-SHISA2	SHISA2	-3.25	1.48E-04	0.006359	Decreased
gene-BTBD6	BTBD6	-3.54	1.49E-04	0.006374	Decreased
gene-GAS2L3	GAS2L3	-2.49	1.50E-04	0.006402	Decreased
gene-SCN1B	SCN1B	-11.21	1.51E-04	0.006453	Decreased
gene-TBX15	TBX15	-13.43	1.52E-04	0.006477	Decreased
gene-PROB1	PROB1	-13.42	1.53E-04	0.006482	Decreased
gene-STC2	STC2	-4.52	1.56E-04	0.006602	Decreased
gene-LOC106731	LOC106731301	-6.45	1.56E-04	0.006612	Decreased
gene-LOC102451	LOC102451438	-11.21	1.59E-04	0.006675	Decreased
gene-TCP11L2	TCP11L2	-2.01	1.59E-04	0.006675	Decreased
gene-TPM1	TPM1	-5.15	1.59E-04	0.006675	Decreased
gene-ABRA	ABRA	-13.25	1.60E-04	0.006702	Decreased
gene-LOC102451	LOC102451408	-1.94	1.63E-04	0.00683	Decreased
gene-LOC102462	LOC102462846	-13.20	1.64E-04	0.006837	Decreased
gene-LOC106732	LOC106732423	-5.23	1.67E-04	0.006946	Decreased
gene-MYF6	MYF6	-13.13	1.67E-04	0.006946	Decreased
gene-PTPRS	PTPRS	-2.30	1.68E-04	0.006963	Decreased
gene-LOC102448	LOC102448272	-6.77	1.69E-04	0.007013	Decreased
gene-GPM6B	GPM6B	-1.70	1.70E-04	0.007027	Decreased
gene-CTHRC1	CTHRC1	-4.24	1.71E-04	0.007037	Decreased
gene-CFAP58	CFAP58	-7.30	1.72E-04	0.007089	Decreased
gene-KCNJ3	KCNJ3	-9.79	1.73E-04	0.007089	Decreased
gene-SYNC	SYNC	-5.10	1.73E-04	0.007103	Decreased
gene-LOC102447	LOC102447528	-2.57	1.74E-04	0.007104	Decreased
gene-UNC45B	UNC45B	-9.68	1.74E-04	0.007104	Decreased
gene-ALDH1A3	ALDH1A3	-3.85	1.75E-04	0.007128	Decreased
gene-LOC102462	LOC102462423	2.38	1.76E-04	0.007163	Increased
gene-LOC112546	LOC112546888	6.45	1.78E-04	0.007218	Increased
gene-CFL2	CFL2	-3.26	1.79E-04	0.00725	Decreased
gene-MBP	MBP	-3.44	1.80E-04	0.007267	Decreased
gene-CDC42EP2	CDC42EP2	-3.07	1.81E-04	0.007279	Decreased

gene-TNFAIP8L1	TNFAIP8L1	-2.07	1.81E-04	0.00729	Decreased
gene-LOC102460	LOC102460470	-6.70	1.82E-04	0.0073	Decreased
gene-CDR2	CDR2	1.58	1.87E-04	0.007473	Increased
gene-TMEM38A	TMEM38A	-5.41	1.87E-04	0.007481	Decreased
gene-LOC102447	LOC102447848	-3.35	1.88E-04	0.007491	Decreased
gene-COL8A1	COL8A1	-7.95	1.89E-04	0.00753	Decreased
gene-SIX1	SIX1	-12.82	1.90E-04	0.00753	Decreased
gene-TPPP3	TPPP3	-4.65	1.92E-04	0.007599	Decreased
gene-KCNS3	KCNS3	-8.05	1.94E-04	0.007686	Decreased
gene-IL17D	IL17D	-9.51	1.96E-04	0.00772	Decreased
gene-LOC102460	LOC102460240	-4.90	1.96E-04	0.00772	Decreased
gene-MYOD1	MYOD1	-12.75	1.96E-04	0.00772	Decreased
gene-TBC1D4	TBC1D4	-2.33	1.98E-04	0.007757	Decreased
gene-RCSD1	RCSD1	-3.08	1.99E-04	0.007779	Decreased
gene-PDE2A	PDE2A	-3.35	2.00E-04	0.007814	Decreased
gene-TRIM55	TRIM55	-12.69	2.02E-04	0.007858	Decreased
gene-LOC102453	LOC102453431	-3.45	2.03E-04	0.007882	Decreased
gene-TBC1D1	TBC1D1	-2.90	2.03E-04	0.007892	Decreased
gene-GREM1	GREM1	-5.99	2.05E-04	0.00794	Decreased
gene-PRICKLE1	PRICKLE1	-3.78	2.05E-04	0.007947	Decreased
gene-NFAM1	NFAM1	-2.75	2.06E-04	0.007957	Decreased
gene-MEF2A	MEF2A	-2.97	2.07E-04	0.007957	Decreased
gene-MYL4	MYL4	-12.62	2.08E-04	0.007995	Decreased
gene-TXNRD1	TXNRD1	-2.24	2.09E-04	0.007995	Decreased
gene-FLRT3	FLRT3	-1.84	2.09E-04	0.007995	Decreased
gene-AMY2A	AMY2A	4.26	2.09E-04	0.007995	Increased
gene-CDC45	CDC45	-2.15	2.10E-04	0.008011	Decreased
gene-CNR2	CNR2	-6.56	2.11E-04	0.008046	Decreased
gene-AKAP6	AKAP6	-12.58	2.13E-04	0.008108	Decreased
gene-ENTPD2	ENTPD2	-2.54	2.15E-04	0.008163	Decreased
gene-MRC2	MRC2	-2.07	2.16E-04	0.008194	Decreased
gene-SPARC	SPARC	-2.29	2.20E-04	0.008297	Decreased
gene-ITGB1BP2	ITGB1BP2	-4.46	2.20E-04	0.008297	Decreased
gene-ITGB2	ITGB2	-2.73	2.21E-04	0.008325	Decreased
gene-APOBEC2	APOBEC2	-8.67	2.28E-04	0.008561	Decreased
gene-SELPLG	SELPLG	-2.41	2.29E-04	0.008583	Decreased
gene-LOC102452	LOC102452069	-2.01	2.29E-04	0.008583	Decreased
gene-TCF4	TCF4	-2.44	2.30E-04	0.008604	Decreased
gene-CDH23	CDH23	-2.63	2.31E-04	0.008609	Decreased
gene-LOC102444	LOC102444244	-8.31	2.32E-04	0.008618	Decreased
gene-LOC102457	LOC102457574	-9.54	2.33E-04	0.008657	Decreased
gene-HSPB3	HSPB3	-12.40	2.35E-04	0.008704	Decreased
gene-NLRC3	NLRC3	2.73	2.36E-04	0.008736	Increased
gene-PEA15	PEA15	-2.92	2.37E-04	0.00876	Decreased
gene-MANSC1	MANSC1	-2.91	2.41E-04	0.008888	Decreased
gene-PTP4A3	PTP4A3	-4.73	2.42E-04	0.008888	Decreased
gene-RHOJ	RHOJ	-2.93	2.43E-04	0.008928	Decreased
gene-FAM180B	FAM180B	-5.10	2.46E-04	0.009014	Decreased
gene-SLC43A3	SLC43A3	-2.40	2.48E-04	0.009067	Decreased
gene-PLA2G5	PLA2G5	-5.38	2.49E-04	0.009078	Decreased
gene-MMRN1	MMRN1	-3.48	2.50E-04	0.009108	Decreased
gene-PLAT	PLAT	-2.59	2.51E-04	0.009117	Decreased
gene-HIVEP1	HIVEP1	1.50	2.52E-04	0.009117	Increased
gene-FGF16	FGF16	-6.47	2.52E-04	0.009117	Decreased

gene-ST8SIA5	ST8SIA5	-4.38	2.54E-04	0.009174	Decreased
gene-KDR	KDR	-1.76	2.56E-04	0.009255	Decreased
gene-CHRND	CHRND	-12.25	2.58E-04	0.009299	Decreased
gene-CSTA	CSTA	-2.44	2.60E-04	0.009355	Decreased
gene-AMIGO1	AMIGO1	-4.37	2.61E-04	0.009355	Decreased
gene-TMEM47	TMEM47	-1.97	2.65E-04	0.009484	Decreased
gene-SLC9A2	SLC9A2	-12.20	2.66E-04	0.009503	Decreased
gene-VGLL2	VGLL2	-12.19	2.67E-04	0.009521	Decreased
gene-LOC102461	LOC102461212	-12.19	2.67E-04	0.009521	Decreased
gene-STAR	STAR	2.82	2.68E-04	0.009526	Increased
gene-RNF152	RNF152	-2.41	2.69E-04	0.009534	Decreased
gene-TMEM204	TMEM204	-2.12	2.70E-04	0.009535	Decreased
gene-PRPH	PRPH	-6.33	2.70E-04	0.009535	Decreased
gene-WNK2	WNK2	-6.11	2.70E-04	0.009535	Decreased
gene-ARHGAP31	ARHGAP31	-2.57	2.71E-04	0.009535	Decreased
gene-ABCC9	ABCC9	-2.76	2.71E-04	0.009536	Decreased
gene-SMYD1	SMYD1	-10.10	2.72E-04	0.009555	Decreased
gene-MDN1	MDN1	1.43	2.73E-04	0.009555	Increased
gene-WFDC1	WFDC1	-5.63	2.75E-04	0.009628	Decreased
gene-EFEMP2	EFEMP2	-2.84	2.79E-04	0.009757	Decreased
gene-SEMA7A	SEMA7A	-2.57	2.80E-04	0.009761	Decreased
gene-BMP5	BMP5	-2.21	2.82E-04	0.009829	Decreased
gene-SYNPO2L	SYNPO2L	-11.64	2.84E-04	0.009856	Decreased
gene-ALDH1L2	ALDH1L2	-3.50	2.86E-04	0.009928	Decreased
gene-ECM2	ECM2	-2.55	2.89E-04	0.009997	Decreased
gene-LOC102454	LOC102454753	-2.54	2.89E-04	0.009999	Decreased
gene-OLFM1	OLFM1	-4.68	2.94E-04	0.010144	Decreased
gene-SLC6A4	SLC6A4	-2.32	2.96E-04	0.010203	Decreased
gene-TSPAN5	TSPAN5	-2.56	2.99E-04	0.010273	Decreased
gene-ARHGAP18	ARHGAP18	-2.47	2.99E-04	0.010273	Decreased
gene-HCLS1	HCLS1	-1.97	3.01E-04	0.01031	Decreased
gene-MYOZ2	MYOZ2	-10.70	3.02E-04	0.010313	Decreased
gene-IGFBP5	IGFBP5	-5.57	3.03E-04	0.010313	Decreased
gene-COL25A1	COL25A1	-3.07	3.03E-04	0.010313	Decreased
gene-ACTN2	ACTN2	-7.62	3.04E-04	0.010313	Decreased
gene-KLHL30	KLHL30	-6.61	3.04E-04	0.010313	Decreased
gene-LOC102455	LOC102455202	-2.20	3.04E-04	0.010313	Decreased
gene-ZNF469	ZNF469	-2.77	3.05E-04	0.010313	Decreased
gene-ANOS1	ANOS1	-5.72	3.08E-04	0.010402	Decreased
gene-LOC112546	LOC112546320	4.05	3.12E-04	0.010524	Increased
gene-SGO1	SGO1	-1.95	3.18E-04	0.01072	Decreased
gene-CACNG5	CACNG5	6.27	3.19E-04	0.010732	Increased
gene-VIM	VIM	-2.25	3.21E-04	0.010756	Decreased
gene-LOC102460	LOC102460124	-9.12	3.22E-04	0.010778	Decreased
gene-MAP2K6	MAP2K6	-2.60	3.24E-04	0.01082	Decreased
gene-NRROS	NRROS	-2.27	3.25E-04	0.010843	Decreased
gene-LOC102461	LOC102461777	-11.89	3.26E-04	0.010848	Decreased
gene-LIFR	LIFR	-2.00	3.27E-04	0.010867	Decreased
gene-COL20A1	COL20A1	-8.78	3.28E-04	0.010877	Decreased
gene-BLVRA	BLVRA	-4.41	3.30E-04	0.01093	Decreased
gene-LOC102443	LOC102443928	-4.85	3.34E-04	0.011042	Decreased
gene-LOC102457	LOC102457174	-2.88	3.39E-04	0.011156	Decreased
gene-TNFSF11	TNFSF11	-6.61	3.39E-04	0.011156	Decreased
gene-MCC	MCC	-2.56	3.40E-04	0.011156	Decreased

gene-SCN2B	SCN2B	-11.83	3.40E-04	0.011156	Decreased
gene-HDAC9	HDAC9	-3.57	3.41E-04	0.011156	Decreased
gene-BAIAP2	BAIAP2	-3.11	3.41E-04	0.011156	Decreased
gene-PQLC3	PQLC3	-2.55	3.41E-04	0.011156	Decreased
gene-LOC102449	LOC102449400	-4.57	3.42E-04	0.011156	Decreased
gene-MYF5	MYF5	-11.82	3.45E-04	0.011233	Decreased
gene-TCP11L1	TCP11L1	-2.72	3.46E-04	0.011254	Decreased
gene-LOC102445	LOC102445800	-3.12	3.47E-04	0.011274	Decreased
gene-TENT5B	TENT5B	-3.12	3.49E-04	0.01131	Decreased
gene-LOC102448	LOC102448412	-7.91	3.50E-04	0.01131	Decreased
gene-RTN4RL1	RTN4RL1	-7.22	3.50E-04	0.01131	Decreased
gene-NLE1	NLE1	1.62	3.51E-04	0.011327	Increased
gene-GLI3	GLI3	-3.81	3.53E-04	0.011376	Decreased
gene-LOC102453	LOC102453427	-5.36	3.54E-04	0.011376	Decreased
gene-LOC112543	LOC112543494	-11.77	3.54E-04	0.011376	Decreased
gene-TEAD3	TEAD3	-2.78	3.55E-04	0.011391	Decreased
gene-TSPAN7	TSPAN7	-3.38	3.57E-04	0.011417	Decreased
gene-LRFN4	LRFN4	-5.41	3.58E-04	0.01143	Decreased
gene-LOC102460	LOC102460693	1.87	3.59E-04	0.011437	Increased
gene-MYOM3	MYOM3	-3.64	3.59E-04	0.011437	Decreased
gene-KLHL41	KLHL41	-4.69	3.60E-04	0.011442	Decreased
gene-MOGAT1	MOGAT1	-3.39	3.65E-04	0.01159	Decreased
gene-USP43	USP43	-3.32	3.66E-04	0.01159	Decreased
gene-PDPN	PDPN	-6.46	3.68E-04	0.011658	Decreased
gene-NAV3	NAV3	-5.52	3.70E-04	0.011698	Decreased
gene-LOC102450	LOC102450447	-8.30	3.71E-04	0.011699	Decreased
gene-FUS	FUS	1.74	3.73E-04	0.011741	Increased
gene-HSPA2	HSPA2	1.72	3.73E-04	0.011741	Increased
gene-CPZ	CPZ	-5.90	3.74E-04	0.011746	Decreased
gene-LOC102460	LOC102460983	-5.43	3.76E-04	0.011797	Decreased
gene-CMYA5	CMYA5	-9.10	3.79E-04	0.011858	Decreased
gene-LOC102458	LOC102458716	1.49	3.81E-04	0.011886	Increased
gene-LOC102453	LOC102453468	1.47	3.82E-04	0.011903	Increased
gene-LOC102455	LOC102455315	-7.27	3.84E-04	0.011965	Decreased
gene-LOC102445	LOC102445741	-10.10	3.86E-04	0.012012	Decreased
gene-GRAMD2A	GRAMD2A	-2.53	3.89E-04	0.012046	Decreased
gene-LOC102452	LOC102452796	-9.38	3.90E-04	0.012046	Decreased
gene-LOC102446	LOC102446018	2.44	3.90E-04	0.012046	Increased
gene-LMCD1	LMCD1	-2.57	3.90E-04	0.012046	Decreased
gene-CLIP4	CLIP4	-2.22	3.94E-04	0.012136	Decreased
gene-VGLL3	VGLL3	-7.72	3.94E-04	0.012136	Decreased
gene-CCNB1	CCNB1	-2.07	3.95E-04	0.012142	Decreased
gene-PMEPA1	PMEPA1	-1.47	3.96E-04	0.012142	Decreased
gene-UACA	UACA	-2.86	4.00E-04	0.012259	Decreased
gene-LOC102443	LOC102443551	1.91	4.03E-04	0.01231	Increased
gene-LOC106732	LOC106732518	-7.37	4.03E-04	0.01231	Decreased
gene-LOC102447	LOC102447223	-3.88	4.08E-04	0.012432	Decreased
gene-CCDC141	CCDC141	-2.57	4.08E-04	0.012432	Decreased
gene-LOC106731	LOC106731405	-8.02	4.10E-04	0.012462	Decreased
gene-SCN3B	SCN3B	-11.56	4.14E-04	0.012546	Decreased
gene-LOC102461	LOC102461694	-2.91	4.14E-04	0.012546	Decreased
gene-HTR2A	HTR2A	-5.17	4.17E-04	0.012623	Decreased
gene-LRTM1	LRTM1	-10.22	4.18E-04	0.012641	Decreased
gene-GPR141	GPR141	-3.22	4.22E-04	0.012745	Decreased

gene-LOC102456	LOC102456925	-3.72	4.25E-04	0.012777	Decreased
gene-IL5RA	IL5RA	-7.31	4.25E-04	0.012777	Decreased
gene-WLS	WLS	-2.09	4.27E-04	0.012818	Decreased
gene-TULP4	TULP4	-2.87	4.28E-04	0.012825	Decreased
gene-LOC102458	LOC102458936	-1.68	4.30E-04	0.012864	Decreased
gene-PREX1	PREX1	-2.31	4.30E-04	0.012864	Decreased
gene-ANKRD1	ANKRD1	-11.50	4.34E-04	0.012966	Decreased
gene-YDJC	YDJC	-4.75	4.38E-04	0.013048	Decreased
gene-BARX2	BARX2	-11.49	4.39E-04	0.013053	Decreased
gene-AIF1L	AIF1L	-4.82	4.41E-04	0.013113	Decreased
gene-LOC102445	LOC102445828	1.58	4.42E-04	0.013123	Increased
gene-LOC106731	LOC106731442	-2.15	4.44E-04	0.013144	Decreased
gene-EMP1	EMP1	-1.57	4.44E-04	0.013144	Decreased
gene-CFAP157	CFAP157	-7.93	4.45E-04	0.013151	Decreased
gene-LOC112546	LOC112546070	-7.09	4.47E-04	0.013179	Decreased
gene-MSS51	MSS51	-7.59	4.48E-04	0.013185	Decreased
gene-MFSD2A	MFSD2A	-1.58	4.48E-04	0.013185	Decreased
gene-LOC102456	LOC102456260	-8.51	4.54E-04	0.013316	Decreased
gene-LOC102444	LOC102444204	-4.81	4.63E-04	0.013576	Decreased
gene-LOC106732	LOC106732070	-7.45	4.66E-04	0.013625	Decreased
gene-EML1	EML1	-3.68	4.67E-04	0.013645	Decreased
gene-LOC102459	LOC102459093	-7.17	4.68E-04	0.013645	Decreased
gene-SEMA3A	SEMA3A	-2.84	4.69E-04	0.01367	Decreased
gene-LOC112544	LOC112544307	2.62	4.73E-04	0.013769	Increased
gene-LOC102455	LOC102455684	-10.20	4.74E-04	0.013769	Decreased
gene-LOC102449	LOC102449499	-3.27	4.75E-04	0.013769	Decreased
gene-GYPC	GYPC	-2.11	4.76E-04	0.013769	Decreased
gene-LOC102462	LOC102462442	11.05	4.76E-04	0.013769	Increased
gene-LOC102457	LOC102457152	-11.36	4.78E-04	0.013793	Decreased
gene-CHRNA1	CHRNA1	-11.36	4.79E-04	0.0138	Decreased
gene-NTNG1	NTNG1	1.47	4.80E-04	0.013811	Increased
gene-FGF6	FGF6	-11.35	4.81E-04	0.013822	Decreased
gene-LOC102459	LOC102459532	-5.36	4.87E-04	0.013954	Decreased
gene-DNAJB9	DNAJB9	1.94	4.87E-04	0.013954	Increased
gene-EMCN	EMCN	-2.35	4.88E-04	0.013954	Decreased
gene-STRIP2	STRIP2	-2.80	4.90E-04	0.014005	Decreased
gene-TSPAN2	TSPAN2	-5.41	4.92E-04	0.014037	Decreased
gene-LOC112545	LOC112545853	-11.30	5.01E-04	0.01429	Decreased
gene-FUT8	FUT8	-2.49	5.05E-04	0.014324	Decreased
gene-LOC102443	LOC102443392	-7.57	5.05E-04	0.014324	Decreased
gene-NCF1	NCF1	-2.94	5.05E-04	0.014324	Decreased
gene-LOC102443	LOC102443716	2.01	5.09E-04	0.014412	Increased
gene-SIM2	SIM2	-11.28	5.12E-04	0.01449	Decreased
gene-LOC102447	LOC102447370	1.83	5.14E-04	0.014523	Increased
gene-TNFAIP6	TNFAIP6	-5.72	5.17E-04	0.014565	Decreased
gene-ZNF106	ZNF106	-3.89	5.17E-04	0.014565	Decreased
gene-ARX	ARX	-11.26	5.18E-04	0.014565	Decreased
gene-LOC112545	LOC112545559	8.47	5.21E-04	0.014622	Increased
gene-FTSJ3	FTSJ3	1.36	5.22E-04	0.014634	Increased
gene-EGF	EGF	-7.75	5.25E-04	0.014686	Decreased
gene-TPX2	TPX2	-1.84	5.27E-04	0.014686	Decreased
gene-MBNL2	MBNL2	-1.61	5.27E-04	0.014686	Decreased
gene-SLC16A7	SLC16A7	-5.41	5.27E-04	0.014686	Decreased
gene-PENK	PENK	9.13	5.27E-04	0.014686	Increased

gene-LOC102460	LOC102460285	-1.95	5.30E-04	0.014739	Decreased
gene-PPP1R26	PPP1R26	-1.45	5.32E-04	0.014765	Decreased
gene-CACHD1	CACHD1	-2.15	5.34E-04	0.014797	Decreased
gene-LOC102446	LOC102446845	-10.05	5.35E-04	0.014803	Decreased
gene-LOC102446	LOC102446945	-5.23	5.36E-04	0.014803	Decreased
gene-NOL3	NOL3	-5.03	5.36E-04	0.014803	Decreased
gene-RASSF2	RASSF2	-2.33	5.38E-04	0.01483	Decreased
gene-LOC102445	LOC102445789	-4.54	5.41E-04	0.014903	Decreased
gene-EMX2	EMX2	-5.81	5.43E-04	0.014924	Decreased
gene-GAS6	GAS6	-2.74	5.44E-04	0.014924	Decreased
gene-LOC102463	LOC102463142	-7.51	5.46E-04	0.014961	Decreased
gene-VSTM4	VSTM4	-4.72	5.58E-04	0.015265	Decreased
gene-SSPN	SSPN	-3.61	5.60E-04	0.015302	Decreased
gene-TMEM200A	TMEM200A	-2.81	5.65E-04	0.015421	Decreased
gene-CCDC80	CCDC80	-1.50	5.66E-04	0.015421	Decreased
gene-LOC102455	LOC102455777	6.26	5.69E-04	0.015485	Increased
gene-LOC102447	LOC102447615	-2.88	5.73E-04	0.015571	Decreased
gene-FBXO40	FBXO40	-11.14	5.76E-04	0.015649	Decreased
gene-PRKCD	PRKCD	-2.16	5.77E-04	0.015655	Decreased
gene-VEGFD	VEGFD	-2.88	5.79E-04	0.015663	Decreased
gene-LOC102448	LOC102448406	-2.37	5.79E-04	0.015663	Decreased
gene-SPATA24	SPATA24	-1.88	5.82E-04	0.0157	Decreased
gene-LOC102457	LOC102457340	-2.13	5.83E-04	0.0157	Decreased
gene-GNG7	GNG7	-1.53	5.84E-04	0.0157	Decreased
gene-RNF150	RNF150	-2.76	5.84E-04	0.0157	Decreased
gene-PLXNA1	PLXNA1	-1.41	5.90E-04	0.015826	Decreased
gene-HSP90B1	HSP90B1	1.99	5.90E-04	0.015826	Increased
gene-CERS4	CERS4	-7.86	5.93E-04	0.015853	Decreased
gene-PERM1	PERM1	-3.44	5.93E-04	0.015853	Decreased
gene-ASB5	ASB5	-5.20	5.98E-04	0.015941	Decreased
gene-LOC106732	LOC106732681	1.86	5.98E-04	0.015941	Increased
gene-SPI1	SPI1	-2.09	6.00E-04	0.015941	Decreased
gene-RIMBP2	RIMBP2	-2.05	6.01E-04	0.015941	Decreased
gene-TBX18	TBX18	-7.42	6.01E-04	0.015941	Decreased
gene-NT5DC2	NT5DC2	-3.13	6.01E-04	0.015941	Decreased
gene-GPD2	GPD2	-2.72	6.02E-04	0.015941	Decreased
gene-LOC102453	LOC102453597	3.92	6.06E-04	0.016026	Increased
gene-LOC102463	LOC102463832	-2.24	6.13E-04	0.016183	Decreased
gene-LOC102456	LOC102456421	-11.07	6.14E-04	0.016183	Decreased
gene-LAMC1	LAMC1	-2.30	6.15E-04	0.016187	Decreased
gene-LOC102454	LOC102454339	-4.17	6.28E-04	0.016525	Decreased
gene-TANC2	TANC2	-2.76	6.31E-04	0.016563	Decreased
gene-LOC102450	LOC102450135	-11.02	6.35E-04	0.016666	Decreased
gene-COL6A3	COL6A3	-1.93	6.40E-04	0.016758	Decreased
gene-SESN3	SESN3	-2.83	6.45E-04	0.01686	Decreased
gene-SMTNL1	SMTNL1	-7.21	6.45E-04	0.01686	Decreased
gene-LOC102463	LOC102463379	-10.99	6.47E-04	0.016883	Decreased
gene-ITGA4	ITGA4	-3.22	6.48E-04	0.016884	Decreased
gene-LOC102451	LOC102451362	-3.30	6.52E-04	0.016955	Decreased
gene-LRRC14B	LRRC14B	-10.98	6.54E-04	0.017007	Decreased
gene-LOC102447	LOC102447683	1.52	6.55E-04	0.017007	Increased
gene-LOC102445	LOC102445141	-11.65	6.67E-04	0.017276	Decreased
gene-PODXL2	PODXL2	-5.70	6.67E-04	0.017276	Decreased
gene-XIRP1	XIRP1	-7.56	6.69E-04	0.017306	Decreased

gene-KCNT1	KCNT1	-10.94	6.75E-04	0.017425	Decreased
gene-NT5E	NT5E	-2.79	6.76E-04	0.017425	Decreased
gene-TMEM132E	TMEM132E	-2.21	6.77E-04	0.01743	Decreased
gene-SMTNL2	SMTNL2	-3.27	6.78E-04	0.017435	Decreased
gene-CAST	CAST	-2.20	6.79E-04	0.017439	Decreased
gene-TYROBP	TYROBP	-1.67	6.81E-04	0.017457	Decreased
gene-MYH7B	MYH7B	-11.62	6.84E-04	0.017519	Decreased
gene-LOC102456	LOC102456995	1.93	6.90E-04	0.017629	Increased
gene-ATP8A2	ATP8A2	-6.59	6.90E-04	0.017629	Decreased
gene-ALPK2	ALPK2	-10.20	6.93E-04	0.017675	Decreased
gene-LOC106732	LOC106732890	-4.96	6.98E-04	0.017788	Decreased
gene-LOC106732	LOC106732354	-7.18	6.99E-04	0.017788	Decreased
gene-ISCA2	ISCA2	1.32	7.00E-04	0.017791	Increased
gene-LOC102461	LOC102461273	-1.82	7.02E-04	0.017815	Decreased
gene-LOC102446	LOC102446308	-1.62	7.04E-04	0.017822	Decreased
gene-TNFRSF19	TNFRSF19	-4.54	7.04E-04	0.017822	Decreased
gene-PRKAR1B	PRKAR1B	-3.39	7.11E-04	0.017982	Decreased
gene-CADM3	CADM3	-1.48	7.15E-04	0.018059	Decreased
gene-LOC102450	LOC102450495	-1.80	7.18E-04	0.01811	Decreased
gene-LOC102460	LOC102460367	-6.15	7.19E-04	0.01811	Decreased
gene-PDE4B	PDE4B	-3.24	7.20E-04	0.018118	Decreased
gene-ACBD4	ACBD4	1.48	7.23E-04	0.018158	Increased
gene-GLRX	GLRX	-2.69	7.25E-04	0.0182	Decreased
gene-KLHL14	KLHL14	-5.23	7.26E-04	0.018202	Decreased
gene-LOC106731	LOC106731839	1.49	7.28E-04	0.01822	Increased
gene-KDM6B	KDM6B	1.64	7.35E-04	0.018361	Increased
gene-CSGALNACT	CSGALNACT1	-2.83	7.37E-04	0.018394	Decreased
gene-LOC102447	LOC102447912	-4.00	7.38E-04	0.018404	Decreased
gene-LOC102459	LOC102459400	-3.53	7.40E-04	0.018419	Decreased
gene-KCNMA1	KCNMA1	-6.89	7.45E-04	0.018525	Decreased
gene-REEP1	REEP1	-5.26	7.46E-04	0.018525	Decreased
gene-NFIX	NFIX	-3.08	7.50E-04	0.018588	Decreased
gene-CRELD2	CRELD2	1.98	7.51E-04	0.01859	Increased
gene-WDR4	WDR4	1.43	7.52E-04	0.018593	Increased
gene-LOC102457	LOC102457416	-10.82	7.54E-04	0.018639	Decreased
gene-RYR3	RYR3	-11.23	7.57E-04	0.018689	Decreased
gene-LOC102450	LOC102450141	-6.02	7.59E-04	0.018695	Decreased
gene-HTRA1	HTRA1	-2.32	7.66E-04	0.018823	Decreased
gene-TMOD4	TMOD4	-4.50	7.66E-04	0.018823	Decreased
gene-PSPC1	PSPC1	1.28	7.73E-04	0.018972	Increased
gene-TLR4	TLR4	-3.47	7.74E-04	0.018988	Decreased
gene-KBTBD13	KBTBD13	-10.78	7.81E-04	0.019121	Decreased
gene-SRPX	SRPX	-2.05	7.82E-04	0.019128	Decreased
gene-LOC102463	LOC102463494	-3.96	7.85E-04	0.019154	Decreased
gene-KAZALD1	KAZALD1	-7.15	7.85E-04	0.019154	Decreased
gene-ATP2A3	ATP2A3	-1.48	7.88E-04	0.019204	Decreased
gene-CHAD	CHAD	-10.77	7.89E-04	0.019204	Decreased
gene-LOC102456	LOC102456961	-1.77	7.91E-04	0.019224	Decreased
gene-ARHGEF10L	ARHGEF10L	-1.98	7.98E-04	0.019336	Decreased
gene-LOC102452	LOC102452702	-3.15	7.98E-04	0.019336	Decreased
gene-ARG2	ARG2	-9.50	7.99E-04	0.019336	Decreased
gene-TMEM159	TMEM159	-1.80	8.13E-04	0.019656	Decreased
gene-PI15	PI15	-5.48	8.14E-04	0.019667	Decreased
gene-FAM151A	FAM151A	-10.74	8.17E-04	0.019701	Decreased

gene-LOC102453	LOC102453393	-8.53	8.18E-04	0.019701	Decreased
gene-ROR1	ROR1	-2.52	8.20E-04	0.019701	Decreased
gene-IVNS1ABP	IVNS1ABP	-2.29	8.20E-04	0.019701	Decreased
gene-OSBPL6	OSBPL6	-5.09	8.24E-04	0.019779	Decreased
gene-BICD2	BICD2	-2.48	8.27E-04	0.019825	Decreased
gene-LOC102461	LOC102461035	-2.44	8.29E-04	0.019826	Decreased
gene-LRRC75A	LRRC75A	-2.52	8.29E-04	0.019826	Decreased
gene-LOC102449	LOC102449223	-9.71	8.31E-04	0.019855	Decreased
gene-COL26A1	COL26A1	-2.28	8.33E-04	0.019875	Decreased
gene-CIRBP	CIRBP	1.59	8.41E-04	0.020048	Increased
gene-LAMP3	LAMP3	-6.00	0.000846	0.020123	Decreased
gene-ADAMTS20	ADAMTS20	-8.50	8.47E-04	0.020123	Decreased
gene-DPYSL2	DPYSL2	-1.59	8.58E-04	0.020363	Decreased
gene-LOC112545	LOC112545411	9.93	8.61E-04	0.0204	Increased
gene-RGCC	RGCC	-10.41	8.61E-04	0.0204	Decreased
gene-WRB	WRB	-1.40	8.65E-04	0.020472	Decreased
gene-LOC102448	LOC102448373	2.83	8.68E-04	0.020518	Increased
gene-ACOD1	ACOD1	-3.94	8.70E-04	0.02054	Decreased
gene-CCNF	CCNF	-1.72	8.78E-04	0.020689	Decreased
gene-CFDP1	CFDP1	1.26	8.80E-04	0.020708	Increased
gene-DOK2	DOK2	-2.68	8.84E-04	0.02078	Decreased
gene-LRRC17	LRRC17	-2.71	8.85E-04	0.02078	Decreased
gene-SLC33A1	SLC33A1	1.58	8.88E-04	0.020835	Increased
gene-PLN	PLN	-1.96	8.93E-04	0.020901	Decreased
gene-LOC106732	LOC106732802	-2.67	8.93E-04	0.020901	Decreased
gene-KCNQ4	KCNQ4	-8.42	8.94E-04	0.020905	Decreased
gene-RGMB	RGMB	-4.30	8.97E-04	0.020927	Decreased
gene-VCAN	VCAN	-4.02	8.98E-04	0.020927	Decreased
gene-PLK1	PLK1	-1.98	8.99E-04	0.020927	Decreased
gene-CRISPLD1	CRISPLD1	-4.03	9.00E-04	0.020927	Decreased
gene-LEMD1	LEMD1	-2.59	9.04E-04	0.020984	Decreased
gene-LOC106731	LOC106731829	-5.87	9.04E-04	0.020984	Decreased
gene-CLDN19	CLDN19	-2.29	9.07E-04	0.021007	Decreased
gene-XKRX	XKRX	-3.38	9.07E-04	0.021007	Decreased
gene-LOC102449	LOC102449377	-8.25	9.11E-04	0.021071	Decreased
gene-VRK2	VRK2	-2.83	9.14E-04	0.021108	Decreased
gene-CREB3L1	CREB3L1	-2.81	9.20E-04	0.021212	Decreased
gene-OSR2	OSR2	-4.28	9.23E-04	0.021267	Decreased
gene-PMP22	PMP22	-2.15	9.25E-04	0.021295	Decreased
gene-SEPT5	5-9月	-1.70	9.27E-04	0.021305	Decreased
gene-LOC102464	LOC102464093	-3.78	9.45E-04	0.021699	Decreased
gene-LOC102450	LOC102450275	-2.72	9.50E-04	0.02179	Decreased
gene-DGCR2	DGCR2	-1.91	9.52E-04	0.021808	Decreased
gene-LOC102456	LOC102456181	-8.68	9.57E-04	0.021884	Decreased
gene-LOC102458	LOC102458090	-2.10	9.62E-04	0.021981	Decreased
gene-THSD7A	THSD7A	-2.69	9.63E-04	0.021982	Decreased
gene-LOC102448	LOC102448731	-2.63	9.69E-04	0.022081	Decreased
gene-JAG2	JAG2	-2.43	9.75E-04	0.0222	Decreased
gene-AARS2	AARS2	1.32	9.77E-04	0.02222	Increased
gene-F2R	F2R	-1.99	9.83E-04	0.022331	Decreased
gene-NRXN2	NRXN2	-8.67	9.84E-04	0.022331	Decreased
gene-GPHB5	GPHB5	-9.38	9.87E-04	0.022369	Decreased
gene-DUSP26	DUSP26	-8.72	9.95E-04	0.022533	Decreased
gene-LOC102451	LOC102451539	6.82	0.001002	0.022658	Increased

gene-LOC112544	LOC112544260	-4.99	0.001011	0.022829	Decreased
gene-LOC102452	LOC102452031	-4.90	0.001016	0.022896	Decreased
gene-SREBF1	SREBF1	-1.73	0.001016	0.022896	Decreased
gene-RUNX1	RUNX1	-2.60	0.001018	0.022896	Decreased
gene-GLI2	GLI2	-3.13	0.001018	0.022896	Decreased
gene-IGF1R	IGF1R	-1.88	0.001025	0.023009	Decreased
gene-CACNB1	CACNB1	-4.09	0.001026	0.023012	Decreased
gene-LOC102461	LOC102461508	-2.37	0.001032	0.023108	Decreased
gene-MYMK	MYMK	-10.49	0.001034	0.023108	Decreased
gene-TMC5	TMC5	-3.15	0.001034	0.023108	Decreased
gene-EBF3	EBF3	-9.52	0.001037	0.023147	Decreased
gene-COL22A1	COL22A1	-4.55	0.00104	0.023197	Decreased
gene-TMEM40	TMEM40	-2.29	0.001046	0.023276	Decreased
gene-TAGLN	TAGLN	-1.46	0.001046	0.023276	Decreased
gene-SPOCK3	SPOCK3	-2.36	0.001048	0.023289	Decreased
gene-LOC102461	LOC102461052	-9.47	0.001062	0.023579	Decreased
gene-ST6GAL1	ST6GAL1	-2.34	0.001068	0.023675	Decreased
gene-ASB4	ASB4	-10.45	0.001069	0.023678	Decreased
gene-LOC102463	LOC102463734	-1.46	0.001071	0.023702	Decreased
gene-EHD4	EHD4	-2.24	0.001073	0.023718	Decreased
gene-MCUB	MCUB	-2.02	0.001075	0.02372	Decreased
gene-MAPKAPK3	MAPKAPK3	-2.28	0.001076	0.023725	Decreased
gene-MMP16	MMP16	-2.74	0.001085	0.023892	Decreased
gene-CAPN2	CAPN2	-2.72	0.001087	0.023906	Decreased
gene-FOS	FOS	-1.89	0.001088	0.023906	Decreased
gene-JCHAIN	JCHAIN	-2.47	0.00109	0.023921	Decreased
gene-LOC102449	LOC102449434	-2.12	0.001094	0.023987	Decreased
gene-MYO18B	MYO18B	-7.86	0.001098	0.024052	Decreased
gene-ISM2	ISM2	-5.21	0.0011	0.024075	Decreased
gene-CHST9	CHST9	-4.13	0.001122	0.024528	Decreased
gene-SLC16A3	SLC16A3	-6.51	0.001124	0.024536	Decreased
gene-HOXD1	HOXD1	-7.35	0.001127	0.024547	Decreased
gene-FIBIN	FIBIN	-3.53	0.001127	0.024547	Decreased
gene-SOX13	SOX13	-1.82	0.001133	0.02464	Decreased
gene-LOC102462	LOC102462321	-7.16	0.001138	0.024724	Decreased
gene-CUNH11orf	CUNH11orf96	-3.11	0.001139	0.024724	Decreased
gene-LOC102453	LOC102453941	-7.70	0.001141	0.024748	Decreased
gene-LRRC32	LRRC32	-2.26	0.001149	0.024859	Decreased
gene-LOC102444	LOC102444628	-7.97	0.001149	0.024859	Decreased
gene-DBX1	DBX1	-10.38	0.001151	0.02488	Decreased
gene-LOC102449	LOC102449766	6.68	0.001156	0.024935	Increased
gene-RALGDS	RALGDS	-1.45	0.001157	0.024935	Decreased
gene-LOC102454	LOC102454920	-10.38	0.001158	0.024935	Decreased
gene-ABLIM3	ABLIM3	-2.68	0.001161	0.024989	Decreased
gene-RASGEF1C	RASGEF1C	-6.28	0.001174	0.025189	Decreased
gene-MLF1	MLF1	-3.45	0.001175	0.025189	Decreased
gene-LAMB1	LAMB1	-1.71	0.001177	0.025189	Decreased
gene-TNNT2	TNNT2	-10.36	0.001179	0.025189	Decreased
gene-CDC20	CDC20	-1.60	0.001179	0.025189	Decreased
gene-LOC102461	LOC102461217	1.79	0.001179	0.025189	Increased
gene-LOC106731	LOC106731492	1.90	0.00118	0.025189	Increased
gene-LAMA5	LAMA5	-1.53	0.001181	0.025189	Decreased
gene-GPATCH4	GPATCH4	1.29	0.001183	0.025194	Increased
gene-LOC112546	LOC112546102	-2.99	0.001185	0.025214	Decreased

gene-COL12A1	COL12A1	-1.67	0.001193	0.025349	Decreased
gene-LOC102459	LOC102459066	1.54	0.001194	0.025349	Increased
gene-LOC102454	LOC102454459	-1.77	0.001204	0.025541	Decreased
gene-PLCB1	PLCB1	-1.55	0.001211	0.025656	Decreased
gene-SPNS2	SPNS2	-6.69	0.001213	0.025658	Decreased
gene-TGFB2	TGFB2	-2.74	0.001214	0.025658	Decreased
gene-RAB15	RAB15	-3.26	0.001216	0.025661	Decreased
gene-LOC102446	LOC102446710	2.28	0.001216	0.025661	Increased
gene-FEV	FEV	-6.71	0.001226	0.025802	Decreased
gene-TGFBI	TGFBI	-2.19	0.001226	0.025802	Decreased
gene-LOC112547	LOC112547508	1.44	0.001227	0.025802	Increased
gene-ARNTL	ARNTL	1.35	0.001229	0.025808	Increased
gene-GNB5	GNB5	-3.44	0.00123	0.025808	Decreased
gene-TBKBP1	TBKBP1	-2.37	0.001235	0.02588	Decreased
gene-LOC102447	LOC102447224	-9.40	0.001236	0.025884	Decreased
gene-LOC102457	LOC102457584	-4.20	0.001239	0.025903	Decreased
gene-PRSS23	PRSS23	-1.52	0.001244	0.025971	Decreased
gene-HEY1	HEY1	-2.26	0.001244	0.025971	Decreased
gene-ARHGAP45	ARHGAP45	-1.71	0.001249	0.026028	Decreased
gene-LOC102451	LOC102451040	-3.02	0.001254	0.026119	Decreased
gene-MGAT3	MGAT3	-1.78	0.001262	0.026256	Decreased
gene-JAK2	JAK2	-1.46	0.001264	0.026276	Decreased
gene-RAB44	RAB44	-2.78	0.001284	0.02665	Decreased
gene-NES	NES	-2.76	0.001294	0.026798	Decreased
gene-PGM2L1	PGM2L1	-3.07	0.001294	0.026798	Decreased
gene-LOC106731	LOC106731632	-2.66	0.001295	0.026798	Decreased
gene-CDKN2C	CDKN2C	-1.66	0.001298	0.026808	Decreased
gene-LOC102459	LOC102459436	5.67	0.001298	0.026808	Increased
gene-DAPK2	DAPK2	-3.52	0.001303	0.026869	Decreased
gene-P4HA3	P4HA3	-4.08	0.001311	0.026992	Decreased
gene-LOC102453	LOC102453376	-10.25	0.001312	0.026992	Decreased
gene-NFATC1	NFATC1	-3.79	0.001313	0.026992	Decreased
gene-B3GNT7	B3GNT7	-4.68	0.001316	0.027031	Decreased
gene-GTPBP8	GTPBP8	1.40	0.001317	0.027031	Increased
gene-LOC102463	LOC102463611	-2.34	0.001326	0.027173	Decreased
gene-GDF1	GDF1	-7.65	0.001327	0.027179	Decreased
gene-SHOX	SHOX	-10.24	0.001334	0.027278	Decreased
gene-RNF24	RNF24	-2.44	0.001341	0.027394	Decreased
gene-TMCC2	TMCC2	-3.33	0.00135	0.027523	Decreased
gene-LOC102445	LOC102445784	-10.17	0.00135	0.027523	Decreased
gene-RAI2	RAI2	-7.00	0.001353	0.027551	Decreased
gene-CD74	CD74	-1.72	0.001357	0.027593	Decreased
gene-LOC102443	LOC102443591	-5.54	0.001359	0.027593	Decreased
gene-LOC106731	LOC106731320	-5.81	0.001359	0.027593	Decreased
gene-CLIP2	CLIP2	-1.69	0.001363	0.027621	Decreased
gene-NCF2	NCF2	-2.45	0.001363	0.027621	Decreased
gene-TMEM52	TMEM52	-2.40	0.001366	0.027641	Decreased
gene-RGS18	RGS18	-1.95	0.001374	0.027791	Decreased
gene-CX3CR1	CX3CR1	-2.57	0.001392	0.028118	Decreased
gene-DUSP10	DUSP10	-2.13	0.001399	0.02823	Decreased
gene-LBH	LBH	-1.41	0.001406	0.028339	Decreased
gene-LOC106731	LOC106731825	-4.02	0.001412	0.028435	Decreased
gene-LOC106732	LOC106732359	-5.01	0.001428	0.028734	Decreased
gene-SETD7	SETD7	-2.87	0.001437	0.028885	Decreased

gene-LOC106732	LOC106732939	-2.30	0.001444	0.028992	Decreased
gene-ASB16	ASB16	-4.43	0.001448	0.029044	Decreased
gene-PKD2	PKD2	-1.37	0.001454	0.029121	Decreased
gene-LOC102455	LOC102455685	-5.66	0.001457	0.029152	Decreased
gene-LOC102447	LOC102447294	-2.90	0.001462	0.02919	Decreased
gene-PLEKHG1	PLEKHG1	-2.67	0.001464	0.02919	Decreased
gene-LOC112544	LOC112544046	2.48	0.001465	0.02919	Increased
gene-HOXC5	HOXC5	-6.34	0.001466	0.02919	Decreased
gene-LRRC2	LRRC2	-4.49	0.001466	0.02919	Decreased
gene-MYPN	MYPN	-3.77	0.001468	0.02919	Decreased
gene-LOC102452	LOC102452743	-4.04	0.001473	0.029259	Decreased
gene-HIC1	HIC1	-1.84	0.00148	0.029375	Decreased
gene-PLEK	PLEK	-2.01	0.001482	0.029387	Decreased
gene-STK17A	STK17A	-2.37	0.001487	0.029464	Decreased
gene-VWA1	VWA1	-1.56	0.00149	0.029468	Decreased
gene-ABCD2	ABCD2	-3.41	0.001491	0.029468	Decreased
gene-LOC106732	LOC106732757	-2.81	0.001499	0.029594	Decreased
gene-LOC102447	LOC102447311	-3.41	0.0015	0.029594	Decreased
gene-LPCAT1	LPCAT1	-2.37	0.001502	0.029594	Decreased
gene-CCDC9B	CCDC9B	-8.14	0.001503	0.029601	Decreased
gene-CASZ1	CASZ1	-3.28	0.001508	0.029666	Decreased
gene-RGL1	RGL1	-1.40	0.001521	0.029882	Decreased
gene-SHCBP1	SHCBP1	-1.80	0.001524	0.029917	Decreased
gene-NSG1	NSG1	-4.11	0.001527	0.029934	Decreased
gene-MYCT1	MYCT1	-2.17	0.00153	0.029978	Decreased
gene-CCDC86	CCDC86	1.42	0.001533	0.030009	Increased
gene-TNFAIP2	TNFAIP2	-5.80	0.001552	0.030339	Decreased
gene-SPACA4	SPACA4	-2.63	0.001554	0.03036	Decreased
gene-LOC106731	LOC106731923	-6.67	0.001564	0.03052	Decreased
gene-LOC102446	LOC102446475	-1.51	0.00158	0.030776	Decreased
gene-LOC112546	LOC112546949	9.66	0.001582	0.030776	Increased
gene-AEBP2	AEBP2	1.44	0.001582	0.030776	Increased
gene-LOC102445	LOC102445332	-8.43	0.001587	0.030847	Decreased
gene-LOC102452	LOC102452806	-1.64	0.001606	0.031173	Decreased
gene-CALCRL	CALCRL	-1.40	0.001608	0.031177	Decreased
gene-KBTBD12	KBTBD12	-7.23	0.001609	0.031177	Decreased
gene-KIF1C	KIF1C	-2.66	0.001614	0.031247	Decreased
gene-IGFBP3	IGFBP3	-1.33	0.001616	0.031257	Decreased
gene-FGF4	FGF4	-10.04	0.001644	0.031753	Decreased
gene-COL4A5	COL4A5	-1.22	0.001648	0.031799	Decreased
gene-LOC102462	LOC102462782	-1.54	0.001665	0.032109	Decreased
gene-AK5	AK5	-2.49	0.001668	0.032121	Decreased
gene-LOC102449	LOC102449842	-2.03	0.001676	0.032258	Decreased
gene-GABRR2	GABRR2	-4.83	0.001684	0.032379	Decreased
gene-UCHL1	UCHL1	-5.36	0.001694	0.032527	Decreased
gene-LOC102443	LOC102443765	-3.20	0.001698	0.03257	Decreased
gene-ZFYVE19	ZFYVE19	1.24	0.001702	0.032615	Increased
gene-SOX7	SOX7	-4.04	0.001703	0.032615	Decreased
gene-LOC102448	LOC102448680	1.58	0.001708	0.032665	Increased
gene-ADGRG1	ADGRG1	-3.04	0.001711	0.032678	Decreased
gene-NEURL1B	NEURL1B	-1.89	0.001712	0.032678	Decreased
gene-LRRC31	LRRC31	-6.43	0.001719	0.032793	Decreased
gene-DUSP6	DUSP6	-1.48	0.001724	0.032851	Decreased
gene-PLAC9	PLAC9	-2.69	0.001728	0.032877	Decreased

gene-LOC102459	LOC102459910	-6.72	0.001729	0.032877	Decreased
gene-LGALS1	LGALS1	-1.76	0.001733	0.032928	Decreased
gene-CARNS1	CARNS1	-4.80	0.001742	0.03306	Decreased
gene-TTC39C	TTC39C	-1.54	0.001758	0.033337	Decreased
gene-LOC102456	LOC102456325	-3.19	0.001762	0.033378	Decreased
gene-PLPP7	PLPP7	-6.35	0.001765	0.03341	Decreased
gene-SYNGAP1	SYNGAP1	-3.46	0.001769	0.033441	Decreased
gene-LOC106732	LOC106732166	-9.96	0.001773	0.033451	Decreased
gene-PHACTR2	PHACTR2	-2.16	0.001774	0.033451	Decreased
gene-LYN	LYN	-1.71	0.001775	0.033451	Decreased
gene-LOC102462	LOC102462624	-2.38	0.001779	0.033496	Decreased
gene-ARPP21	ARPP21	-6.30	0.001781	0.033504	Decreased
gene-INPP4A	INPP4A	-1.39	0.001785	0.033519	Decreased
gene-LOC102459	LOC102459430	-3.73	0.001786	0.033519	Decreased
gene-LOC102463	LOC102463364	-1.53	0.001787	0.033519	Decreased
gene-LOC102449	LOC102449267	-4.60	0.001807	0.033853	Decreased
gene-HPSE	HPSE	-1.78	0.001808	0.033853	Decreased
gene-KLHL24	KLHL24	-2.04	0.001818	0.034003	Decreased
gene-TLCD1	TLCD1	1.33	0.00183	0.034178	Increased
gene-MLIP	MLIP	-10.21	0.001831	0.034178	Decreased
gene-LOC102457	LOC102457168	-1.56	0.001842	0.034364	Decreased
gene-MYOZ3	MYOZ3	-2.60	0.001849	0.034446	Decreased
gene-TMTC2	TMTC2	-2.30	0.00185	0.034446	Decreased
gene-ADAM8	ADAM8	-4.72	0.001855	0.03447	Decreased
gene-ELOVL6	ELOVL6	-2.03	0.001855	0.03447	Decreased
gene-PPM1E	PPM1E	-2.56	0.001861	0.034529	Decreased
gene-LOC102451	LOC102451500	-2.30	0.001862	0.034529	Decreased
gene-LDH-A	LDH-A	-2.89	0.001864	0.034529	Decreased
gene-HVCN1	HVCN1	-2.25	0.001885	0.034892	Decreased
gene-LOC102456	LOC102456975	1.30	0.001888	0.034903	Increased
gene-TMEM65	TMEM65	-2.47	0.001889	0.034903	Decreased
gene-MTFR2	MTFR2	-1.95	0.001891	0.034904	Decreased
gene-CRTAP	CRTAP	-2.24	0.001901	0.035028	Decreased
gene-SLC22A16	SLC22A16	-7.93	0.001901	0.035028	Decreased
gene-LOC106731	LOC106731529	1.54	0.001911	0.035121	Increased
gene-TTK	TTK	-1.94	0.001912	0.035121	Decreased
gene-LOC112544	LOC112544112	1.48	0.001913	0.035121	Increased
gene-BAAT	BAAT	1.74	0.001914	0.035121	Increased
gene-CUNH1orf1	CUNH1orf167	-3.84	0.001919	0.035194	Decreased
gene-CPLX4	CPLX4	2.80	0.001922	0.035204	Increased
gene-GLIPR2	GLIPR2	-2.05	0.001943	0.035514	Decreased
gene-CA4	CA4	-1.92	0.001945	0.035514	Decreased
gene-LOC102462	LOC102462444	-4.61	0.001946	0.035514	Decreased
gene-EPHA3	EPHA3	-3.54	0.001946	0.035514	Decreased
gene-TOR4A	TOR4A	-1.95	0.001949	0.035531	Decreased
gene-TTC27	TTC27	1.32	0.001952	0.035566	Increased
gene-SEC31A	SEC31A	1.57	0.001954	0.03557	Increased
gene-LOC106731	LOC106731296	-1.64	0.001963	0.035668	Decreased
gene-TTI2	TTI2	1.26	0.001963	0.035668	Increased
gene-LOC102457	LOC102457850	1.82	0.001975	0.035782	Increased
gene-SLA	SLA	-1.60	0.001975	0.035782	Decreased
gene-ANKRD33	ANKRD33	-9.87	0.001977	0.035782	Decreased
gene-LOC102455	LOC102455367	-4.88	0.001977	0.035782	Decreased
gene-QPCT	QPCT	-2.94	0.001986	0.03588	Decreased

gene-NOS1	NOS1	-6.62	0.001986	0.03588	Decreased
gene-ADORA2A	ADORA2A	-1.51	0.001989	0.0359	Decreased
gene-LOC102452	LOC102452824	-4.40	0.001992	0.035913	Decreased
gene-IFRD1	IFRD1	1.42	0.001994	0.035913	Increased
gene-KCNMB1	KCNMB1	-1.82	0.001995	0.035913	Decreased
gene-MYL9	MYL9	-1.37	0.002005	0.036015	Decreased
gene-CILP	CILP	-4.90	0.002005	0.036015	Decreased
gene-NRP1	NRP1	-1.55	0.002014	0.036144	Decreased
gene-TMEM129	TMEM129	1.19	0.002017	0.036172	Increased
gene-UTP20	UTP20	1.28	0.002027	0.036311	Increased
gene-CACNA1B	CACNA1B	-7.93	0.002031	0.036357	Decreased
gene-LRRC39	LRRC39	-6.48	0.002038	0.036444	Decreased
gene-HTRA4	HTRA4	-3.26	0.002051	0.036629	Decreased
gene-P2RX5	P2RX5	-8.95	0.002052	0.036629	Decreased
gene-KIAA0754	KIAA0754	-3.58	0.002055	0.036641	Decreased
gene-SMC4	SMC4	-1.52	0.002057	0.036641	Decreased
gene-PLEKHB2	PLEKHB2	-1.69	0.002064	0.036732	Decreased
gene-GPR182	GPR182	-2.44	0.002069	0.036792	Decreased
gene-MSN	MSN	-1.48	0.002072	0.036821	Decreased
gene-L3MBTL3	L3MBTL3	-2.09	0.002077	0.036855	Decreased
gene-LOC102458	LOC102458332	3.71	0.002078	0.036855	Increased
gene-TNFSF8	TNFSF8	-6.37	0.00208	0.036865	Decreased
gene-ARHGAP11A	ARHGAP11A	-1.48	0.002096	0.037112	Decreased
gene-LOC112546	LOC112546558	-6.30	0.002109	0.037301	Decreased
gene-EFEMP1	EFEMP1	-2.26	0.002111	0.037301	Decreased
gene-LOC102452	LOC102452665	-2.23	0.002113	0.037301	Decreased
gene-DHDDS	DHDDS	1.24	0.002116	0.037325	Increased
gene-LOC102462	LOC102462718	-5.18	0.002124	0.037431	Decreased
gene-LOC102452	LOC102452890	-9.81	0.002146	0.03779	Decreased
gene-ASB18	ASB18	-9.81	0.002149	0.037813	Decreased
gene-IL2RG	IL2RG	-1.75	0.002156	0.037894	Decreased
gene-SRP19	SRP19	1.42	0.00216	0.037923	Increased
gene-TMEM25	TMEM25	1.29	0.002166	0.038001	Increased
gene-LOC102445	LOC102445302	-2.80	0.002172	0.038065	Decreased
gene-IRF7	IRF7	-2.71	0.002174	0.038065	Decreased
gene-MBOAT1	MBOAT1	-2.53	0.002178	0.038109	Decreased
gene-HSPB2	HSPB2	-7.54	0.002182	0.038127	Decreased
gene-RBM12B	RBM12B	1.24	0.002184	0.038127	Increased
gene-CEL	CEL	5.76	0.002185	0.038127	Increased
gene-ERO1A	ERO1A	1.41	0.00219	0.038177	Increased
gene-NABP1	NABP1	2.22	0.002192	0.038177	Increased
gene-PLCH2	PLCH2	-1.91	0.002194	0.038177	Decreased
gene-GAMT	GAMT	-3.02	0.002196	0.038177	Decreased
gene-LOC102452	LOC102452280	-1.95	0.002202	0.038232	Decreased
gene-KIF24	KIF24	-2.53	0.002203	0.038232	Decreased
gene-PPM1F	PPM1F	-2.00	0.002215	0.038408	Decreased
gene-LOC102455	LOC102455962	-3.80	0.002217	0.038408	Decreased
gene-JPH3	JPH3	-3.39	0.00223	0.038607	Decreased
gene-LPAR1	LPAR1	-1.88	0.002236	0.03868	Decreased
gene-CADM2	CADM2	-9.77	0.002241	0.038731	Decreased
gene-LOC102448	LOC102448870	-5.39	0.002253	0.038899	Decreased
gene-DDX3X	DDX3X	1.48	0.002256	0.038918	Increased
gene-PDK4	PDK4	-1.87	0.00226	0.038936	Decreased
gene-PLS1	PLS1	-2.31	0.002261	0.038936	Decreased

gene-TRIP10	TRIP10	-1.73	0.002265	0.038964	Decreased
gene-LOC102448	LOC102448244	-2.53	0.002268	0.038974	Decreased
gene-LOC102464	LOC102464019	6.38	0.00227	0.038974	Increased
gene-LOC102447	LOC102447429	1.78	0.002271	0.038974	Increased
gene-TMEM37	TMEM37	-1.72	0.00228	0.039089	Decreased
gene-LOC102459	LOC102459484	-1.47	0.002293	0.039283	Decreased
gene-LOC106731	LOC106731824	-2.98	0.002307	0.039482	Decreased
gene-CENPE	CENPE	-1.82	0.002317	0.039608	Decreased
gene-FLI1	FLI1	-1.56	0.002324	0.039647	Decreased
gene-LOC102448	LOC102448459	-1.83	0.002325	0.039647	Decreased
gene-ADAM10	ADAM10	-1.17	0.002325	0.039647	Decreased
gene-LOC102461	LOC102461172	1.78	0.002343	0.039918	Increased
gene-MYBPC3	MYBPC3	-6.27	0.002347	0.03996	Decreased
gene-ZBTB44	ZBTB44	-1.67	0.002356	0.040079	Decreased
gene-LOC102445	LOC102445757	-7.64	0.002372	0.040312	Decreased
gene-MYOF	MYOF	-1.61	0.002386	0.040521	Decreased
gene-LOC102451	LOC102451058	-3.90	0.002392	0.04054	Decreased
gene-LOC102444	LOC102444600	-3.63	0.002393	0.04054	Decreased
gene-CDC42EP3	CDC42EP3	-2.40	0.002394	0.04054	Decreased
gene-PDCD4	PDCD4	-1.62	0.002405	0.040643	Decreased
gene-TPM3	TPM3	-2.57	0.002405	0.040643	Decreased
gene-ADAM23	ADAM23	-2.49	0.002406	0.040643	Decreased
gene-LOC102462	LOC102462297	-4.32	0.002411	0.040696	Decreased
gene-METRNL	METRNL	-1.45	0.002427	0.040918	Decreased
gene-MPEG1	MPEG1	-1.74	0.002439	0.041094	Decreased
gene-LOC102462	LOC102462824	5.06	0.002442	0.041109	Increased
gene-PHPT1	PHPT1	1.30	0.002457	0.041313	Increased
gene-LOC106731	LOC106731340	1.70	0.002463	0.041388	Increased
gene-LOC102458	LOC102458579	-7.67	0.002468	0.041431	Decreased
gene-SRGAP3	SRGAP3	-3.37	0.002475	0.041524	Decreased
gene-CDH15	CDH15	-9.69	0.002485	0.041646	Decreased
gene-LOC102455	LOC102455085	-2.64	0.00249	0.041701	Decreased
gene-TAX1BP3	TAX1BP3	-1.38	0.002495	0.041737	Decreased
gene-MYLIP	MYLIP	-1.89	0.002503	0.041836	Decreased
gene-LOC102451	LOC102451319	-1.53	0.002507	0.041872	Decreased
gene-LOC112547	LOC112547061	2.01	0.00251	0.041881	Increased
gene-LOC102453	LOC102453747	6.15	0.002516	0.041936	Increased
gene-AHSA1	AHSA1	1.36	0.002517	0.041936	Increased
gene-PLEKHO1	PLEKHO1	-1.88	0.002524	0.04201	Decreased
gene-VRK1	VRK1	-1.19	0.00253	0.042074	Decreased
gene-LOC102456	LOC102456330	-8.45	0.002539	0.042185	Decreased
rna-NC_006132.	rna-NC_00613[illegible]	1.64	0.002543	0.042226	Increased
gene-MEGF11	MEGF11	-5.86	0.002548	0.042264	Decreased
gene-LOC102447	LOC102447253	1.64	0.00255	0.042266	Increased
gene-KLF7	KLF7	-1.74	0.002554	0.042292	Decreased
gene-NDUFAF2	NDUFAF2	1.61	0.002568	0.042495	Increased
gene-LOC102463	LOC102463598	-1.23	0.002573	0.042544	Decreased
gene-PIN1	PIN1	-1.63	0.002587	0.042728	Decreased
gene-LOC106733	LOC106733067	-6.73	0.002609	0.043037	Decreased
gene-DACT1	DACT1	-2.07	0.00261	0.043037	Decreased
gene-NQO1	NQO1	-1.51	0.002632	0.043368	Decreased
gene-LOC102458	LOC102458647	-8.69	0.002635	0.043383	Decreased
gene-BCAS4	BCAS4	-1.89	0.002639	0.043404	Decreased
gene-MERTK	MERTK	-1.75	0.002652	0.043552	Decreased

gene-PTAFR	PTAFR	-2.63	0.002654	0.043552	Decreased
gene-GPM6A	GPM6A	-5.81	0.002656	0.043552	Decreased
gene-LOC112544	LOC112544823	-8.64	0.002657	0.043552	Decreased
gene-TIMM9	TIMM9	1.25	0.002665	0.043644	Increased
gene-KANK3	KANK3	-1.54	0.002672	0.043702	Decreased
gene-WNT9A	WNT9A	-1.31	0.002673	0.043702	Decreased
gene-CASTOR2	CASTOR2	-2.22	0.002676	0.04372	Decreased
gene-LOC102444	LOC102444635	-7.54	0.002682	0.043764	Decreased
gene-ANKRD9	ANKRD9	-2.14	0.002683	0.043764	Decreased
gene-TSPAN9	TSPAN9	-1.79	0.002696	0.043907	Decreased
gene-WNT5A	WNT5A	-1.67	0.002696	0.043907	Decreased
gene-CRY1	CRY1	-1.20	0.002699	0.043908	Decreased
gene-LOC102461	LOC102461370	-3.47	0.002705	0.043972	Decreased
gene-CLEC17A	CLEC17A	-6.37	0.002726	0.044267	Decreased
gene-KIDINS220	KIDINS220	-1.41	0.002727	0.044267	Decreased
gene-LOC102445	LOC102445365	6.06	0.002732	0.044301	Increased
gene-LOC102447	LOC102447669	2.75	0.002747	0.044517	Increased
gene-ADD3	ADD3	-1.48	0.002754	0.044593	Decreased
gene-FAM98A	FAM98A	1.37	0.002761	0.044655	Increased
gene-LOC112546	LOC112546151	5.46	0.002771	0.044786	Increased
gene-PER2	PER2	-1.55	0.002806	0.045322	Decreased
gene-ADGRL3	ADGRL3	-2.44	0.00282	0.045498	Decreased
gene-FGD3	FGD3	-1.84	0.00283	0.04559	Decreased
gene-WDFY4	WDFY4	-2.22	0.00283	0.04559	Decreased
gene-TMEM223	TMEM223	1.13	0.002835	0.045639	Increased
gene-LOC102456	LOC102456197	-1.53	0.002851	0.045837	Decreased
gene-LOC102459	LOC102459166	2.45	0.002852	0.045837	Increased
gene-PPP2R2C	PPP2R2C	-2.41	0.002864	0.045983	Decreased
gene-MTHFD2	MTHFD2	-2.41	0.002866	0.045984	Decreased
gene-LOC102454	LOC102454860	-8.31	0.002869	0.045986	Decreased
gene-PCK1	PCK1	3.47	0.002873	0.04602	Increased
gene-EHBP1L1	EHBP1L1	-2.57	0.002884	0.046094	Decreased
gene-RACGAP1	RACGAP1	-2.11	0.002885	0.046094	Decreased
gene-CHN1	CHN1	-2.63	0.002885	0.046094	Decreased
gene-LOC102456	LOC102456223	-3.15	0.0029	0.046298	Decreased
gene-PRX	PRX	-1.36	0.002934	0.046796	Decreased
gene-MLLT11	MLLT11	-9.53	0.002936	0.046796	Decreased
gene-FABP3	FABP3	-5.99	0.002944	0.04689	Decreased
gene-LOC102455	LOC102455347	-3.25	0.002947	0.046896	Decreased
gene-MRPL20	MRPL20	1.18	0.002951	0.046917	Increased
gene-GMNC	GMNC	-2.26	0.002964	0.047093	Decreased
gene-CBL	CBL	-1.83	0.002976	0.047234	Decreased
gene-HOXA9	HOXA9	-8.71	0.002978	0.047234	Decreased
gene-SMC2	SMC2	-1.70	0.002983	0.047269	Decreased
gene-PODXL	PODXL	-2.20	0.002987	0.047297	Decreased
gene-NR1H3	NR1H3	-1.39	0.002989	0.047297	Decreased
gene-LOC112547	LOC112547335	1.93	0.002995	0.047335	Increased
gene-LOC102453	LOC102453786	-9.52	0.002997	0.047335	Decreased
gene-LOC102452	LOC102452820	-2.20	0.003001	0.047371	Decreased
gene-LOC102445	LOC102445244	-4.15	0.003005	0.047396	Decreased
gene-IZUMO4	IZUMO4	-1.87	0.00301	0.047434	Decreased
gene-CAP2	CAP2	-3.35	0.003016	0.047484	Decreased
gene-LOC102455	LOC102455936	-6.12	0.00302	0.047499	Decreased
gene-TMEM255A	TMEM255A	-3.02	0.003022	0.047499	Decreased

gene-RARB	RARB	-2.50	0.003029	0.047574	Decreased
gene-LOC102446	LOC102446326	-2.00	0.003034	0.04758	Decreased
gene-NETO2	NETO2	-3.24	0.003035	0.04758	Decreased
gene-RGS3	RGS3	-1.40	0.003037	0.04758	Decreased
gene-NUF2	NUF2	-1.54	0.003042	0.047619	Decreased
gene-CALHM2	CALHM2	-4.15	0.003044	0.047619	Decreased
gene-COQ10B	COQ10B	-1.99	0.003064	0.047899	Decreased
gene-LOC102457	LOC102457886	-2.74	0.003069	0.047931	Decreased
gene-LOC102453	LOC102453262	3.47	0.003101	0.0484	Increased
gene-TSPAN15	TSPAN15	-3.54	0.003107	0.048443	Decreased
gene-LOC112546	LOC112546175	5.78	0.003111	0.048468	Increased
gene-LOC106732	LOC106732236	-9.47	0.003125	0.048655	Decreased
gene-LOC102457	LOC102457786	-3.33	0.003133	0.048739	Decreased
gene-PALD1	PALD1	-2.79	0.00315	0.048957	Decreased
gene-SEMA5B	SEMA5B	-2.17	0.003153	0.04896	Decreased
gene-DOCK4	DOCK4	-1.82	0.003155	0.04896	Decreased
gene-IRS1	IRS1	-2.67	0.003165	0.049081	Decreased
gene-FNIP1	FNIP1	-1.78	0.003169	0.049109	Decreased
gene-SLC12A4	SLC12A4	-1.34	0.003173	0.049112	Decreased
gene-LOC102455	LOC102455980	-10.42	0.003175	0.049112	Decreased
gene-SOX10	SOX10	-9.46	0.003199	0.049443	Decreased
gene-POLR1E	POLR1E	1.14	0.003204	0.049482	Increased
gene-LOC102463	LOC102463999	-1.65	0.003211	0.049559	Decreased
gene-LRRC6	LRRC6	-6.19	0.003215	0.049574	Decreased
gene-LOC102455	LOC102455075	-1.76	0.003222	0.04964	Decreased
gene-CKAP2	CKAP2	-2.01	0.00323	0.049672	Decreased
gene-ACSL1	ACSL1	1.68	0.003231	0.049672	Increased
gene-XYLT1	XYLT1	-3.67	0.003232	0.049672	Decreased
gene-LOC112546	LOC112546124	-7.38	0.003234	0.049672	Decreased
gene-EFNB2	EFNB2	-1.76	0.003237	0.049678	Decreased
gene-LOC102444	LOC102444032	1.20	0.003241	0.049699	Increased

Double HW *vs* CTRL

Gene ID	Gene name	Log_2FC	*P* value	*P* adj	level
gene-LOC102	LOC102447587	-12.01	5.97E-08	0.001169	Decreased
gene-LOC102	LOC102457799	-5.46	2.03E-07	0.001989	Decreased
gene-IGF1	IGF1	-4.27	4.29E-07	0.002799	Decreased
gene-LOC102	LOC102461675	11.60	5.95E-07	0.002915	Increased
gene-MSS51	MSS51	-12.43	1.20E-06	0.003833	Decreased
gene-FDFT1	FDFT1	-2.91	1.37E-06	0.003833	Decreased
gene-LOC112	LOC112546150	-3.57	1.65E-06	0.003833	Decreased
gene-LOC102	LOC102452168	-11.75	1.72E-06	0.003833	Decreased
gene-LOC102	LOC102459228	7.80	1.76E-06	0.003833	Increased
gene-LOC102	LOC102458707	3.61	3.74E-06	0.00732	Increased
gene-FLRT3	FLRT3	-2.06	4.52E-06	0.008052	Decreased
gene-ADIPOQ	ADIPOQ	-7.94	6.19E-06	0.00976	Decreased
gene-LOC102	LOC102456827	3.36	6.48E-06	0.00976	Increased
gene-SYPL2	SYPL2	-8.16	8.35E-06	0.011687	Decreased
gene-LOC102	LOC102455936	-10.28	1.04E-05	0.013566	Decreased
gene-LOC102	LOC102445123	-7.09	1.17E-05	0.014304	Decreased
gene-PLK1	PLK1	-1.92	1.40E-05	0.016113	Decreased
gene-CIART	CIART	-2.08	1.48E-05	0.016113	Decreased
gene-KCNS3	KCNS3	-9.64	1.77E-05	0.016255	Decreased
gene-PLCD4	PLCD4	-7.68	1.79E-05	0.016255	Decreased
gene-GATM	GATM	-8.25	1.82E-05	0.016255	Decreased
gene-SERPINI	SERPINH1	2.37	1.89E-05	0.016255	Increased
gene-LOC102	LOC102463012	-7.88	1.91E-05	0.016255	Decreased
gene-LOC112	LOC112546115	-4.15	2.30E-05	0.017512	Decreased
gene-LOC102	LOC102454405	-10.24	2.37E-05	0.017512	Decreased
gene-LOC102	LOC102453880	-6.79	2.42E-05	0.017512	Decreased
gene-ASB16	ASB16	-5.60	2.56E-05	0.017512	Decreased
gene-NOS1	NOS1	-10.05	2.64E-05	0.017512	Decreased
gene-MYPN	MYPN	-4.46	2.67E-05	0.017512	Decreased
gene-XIRP2	XIRP2	-7.08	2.72E-05	0.017512	Decreased
gene-CUNH4o	CUNH4orf54	-14.31	2.85E-05	0.017512	Decreased
gene-AK1	AK1	-5.79	2.86E-05	0.017512	Decreased
gene-MYLK4	MYLK4	-6.61	3.17E-05	0.018408	Decreased
gene-LOC102	LOC102451909	-5.15	3.28E-05	0.018408	Decreased
gene-RSRP1	RSRP1	2.14	3.48E-05	0.018408	Increased
gene-FHL1	FHL1	-7.39	3.71E-05	0.018408	Decreased
gene-CHRNE	CHRNE	-6.15	3.75E-05	0.018408	Decreased
gene-BATF	BATF	-3.02	3.85E-05	0.018408	Decreased
gene-MYOM3	MYOM3	-4.24	3.86E-05	0.018408	Decreased
gene-CAPN3	CAPN3	-6.77	3.99E-05	0.018408	Decreased
gene-EYA4	EYA4	-13.04	4.06E-05	0.018408	Decreased
gene-AQP4	AQP4	-8.39	4.08E-05	0.018408	Decreased
gene-LOC102	LOC102453787	-9.90	4.11E-05	0.018408	Decreased
gene-PFKM	PFKM	-5.17	4.13E-05	0.018408	Decreased
gene-PYGM	PYGM	-6.25	4.25E-05	0.018521	Decreased
gene-PKIA	PKIA	-3.76	4.37E-05	0.018608	Decreased
gene-NRAP	NRAP	-6.23	4.50E-05	0.01874	Decreased
gene-PER1	PER1	-1.97	4.88E-05	0.018867	Decreased
gene-LOC102	LOC102447514	-12.88	4.93E-05	0.018867	Decreased
gene-LOC102	LOC102446585	-4.22	4.94E-05	0.018867	Decreased
gene-HSPH1	HSPH1	2.24	4.95E-05	0.018867	Increased
gene-KY	KY	-12.36	5.01E-05	0.018867	Decreased

gene-KLHL41	KLHL41	-5.41	5.23E-05	0.018994	Decreased
gene-ASB2	ASB2	-7.25	5.29E-05	0.018994	Decreased
gene-TMOD1	TMOD1	-6.13	5.33E-05	0.018994	Decreased
gene-HACD1	HACD1	-6.40	5.47E-05	0.019143	Decreased
gene-HSPB1	HSPB1	-6.55	5.72E-05	0.019654	Decreased
gene-SLC43A2	SLC43A2	-3.87	5.84E-05	0.019723	Decreased
gene-MB	MB	-9.81	6.24E-05	0.020708	Decreased
gene-SQLE	SQLE	-5.13	6.50E-05	0.021228	Decreased
gene-LOC106'	LOC106732484	-10.41	6.82E-05	0.021863	Decreased
gene-ALPK3	ALPK3	-5.77	6.92E-05	0.021863	Decreased
gene-DNAJA4	DNAJA4	2.31	7.23E-05	0.02249	Increased
gene-MPZ	MPZ	-6.86	7.35E-05	0.022508	Decreased
gene-MYLK2	MYLK2	-5.27	7.50E-05	0.022599	Decreased
gene-FHOD3	FHOD3	-6.70	7.74E-05	0.022968	Decreased
gene-TPMT	TPMT	2.06	8.06E-05	0.023559	Increased
gene-LOC102	LOC102452756	-4.74	8.46E-05	0.023742	Decreased
gene-FILIP1L	FILIP1L	-3.23	8.48E-05	0.023742	Decreased
gene-SLC16A3	SLC16A3	-6.06	8.48E-05	0.023742	Decreased
gene-MBP	MBP	-3.56	9.23E-05	0.02515	Decreased
gene-CCDC68	CCDC68	7.08	9.24E-05	0.02515	Increased
gene-PLP1	PLP1	-5.62	9.44E-05	0.025318	Decreased
gene-PDLIM3	PDLIM3	-5.42	9.72E-05	0.025589	Decreased
gene-LOC102	LOC102459178	-9.36	9.80E-05	0.025589	Decreased
gene-LOC102	LOC102463217	-3.52	1.05E-04	0.026731	Decreased
gene-KIAA11	KIAA1143	-1.56	1.05E-04	0.026731	Decreased
gene-LOC102	LOC102455412	-14.47	1.09E-04	0.027125	Decreased
gene-LIPG	LIPG	-2.58	1.09E-04	0.027125	Decreased
gene-CHRNB1	CHRNB1	-5.45	1.12E-04	0.027226	Decreased
gene-TMEM38A	TMEM38A	-5.67	1.13E-04	0.027226	Decreased
gene-SGCD	SGCD	-5.52	1.17E-04	0.027857	Decreased
gene-SMTNL1	SMTNL1	-7.47	1.22E-04	0.027857	Decreased
gene-MCF2	MCF2	-4.32	1.23E-04	0.027857	Decreased
gene-APOBEC2	APOBEC2	-8.13	1.23E-04	0.027857	Decreased
gene-LOC102	LOC102459215	-3.71	1.24E-04	0.027857	Decreased
gene-ITGB1BI	ITGB1BP2	-4.77	1.24E-04	0.027857	Decreased
gene-TRIM63	TRIM63	-6.79	1.29E-04	0.028761	Decreased
gene-LIMCH1	LIMCH1	-4.00	1.32E-04	0.028998	Decreased
gene-LOC102	LOC102453483	-2.21	1.40E-04	0.030264	Decreased
gene-PRKCQ	PRKCQ	-5.88	1.41E-04	0.030264	Decreased
gene-MFSD2A	MFSD2A	-1.73	1.42E-04	0.030264	Decreased
gene-WIPF3	WIPF3	-6.88	1.46E-04	0.030516	Decreased
gene-TPX2	TPX2	-1.69	1.49E-04	0.030516	Decreased
gene-LRRC20	LRRC20	-3.76	1.50E-04	0.030516	Decreased
gene-NEXN	NEXN	-5.78	1.50E-04	0.030516	Decreased
gene-MYBPC1	MYBPC1	-9.97	1.52E-04	0.030516	Decreased
gene-JPH2	JPH2	-4.85	1.53E-04	0.030516	Decreased
gene-LOC102	LOC102451731	-8.68	1.55E-04	0.030599	Decreased
gene-TPM1	TPM1	-5.13	1.66E-04	0.032512	Decreased
gene-NPAS2	NPAS2	2.48	1.76E-04	0.034147	Increased
gene-VLDLR	VLDLR	-3.73	1.83E-04	0.035116	Decreased
gene-DTNA	DTNA	-5.83	1.87E-04	0.035562	Decreased
gene-MYL4	MYL4	-12.60	1.91E-04	0.035936	Decreased
gene-ERICH3	ERICH3	-5.58	1.99E-04	0.036559	Decreased
gene-LPIN1	LPIN1	-1.76	2.00E-04	0.036559	Decreased

gene-MYOM2	MYOM2	-9.04	2.01E-04	0.036559	Decreased
gene-USP13	USP13	-7.43	2.02E-04	0.036559	Decreased
gene-BIN1	BIN1	-4.89	2.11E-04	0.037429	Decreased
gene-LOC102	LOC102456925	-3.83	2.12E-04	0.037429	Decreased
gene-FHL3	FHL3	-4.86	2.12E-04	0.037429	Decreased
gene-BCAT1	BCAT1	-3.85	2.14E-04	0.037429	Decreased
gene-RNF207	RNF207	-4.96	2.19E-04	0.037943	Decreased
gene-SLC25A	SLC25A12	-5.00	2.24E-04	0.038441	Decreased
gene-MYOM1	MYOM1	-4.80	2.26E-04	0.038441	Decreased
gene-CCNB1	CCNB1	-1.73	2.31E-04	0.038924	Decreased
gene-SLC9A2	SLC9A2	-12.18	2.42E-04	0.039709	Decreased
gene-LDB3	LDB3	-8.88	2.42E-04	0.039709	Decreased
gene-LOC102	LOC102461212	-12.17	2.43E-04	0.039709	Decreased
gene-MEF2C	MEF2C	-3.92	2.44E-04	0.039709	Decreased
gene-LOC102	LOC102444635	-10.30	2.45E-04	0.039709	Decreased
gene-LOC102	LOC102455319	-9.06	2.49E-04	0.039919	Decreased
gene-MYOT	MYOT	-7.32	2.53E-04	0.040228	Decreased
gene-LOC102	LOC102445544	-4.56	2.64E-04	0.041226	Decreased
gene-CUNH10	CUNH10orf71	-7.83	2.65E-04	0.041226	Decreased
gene-CAPN10	CAPN10	-1.64	2.69E-04	0.041226	Decreased
gene-PAG1	PAG1	-2.52	2.69E-04	0.041226	Decreased
gene-LOC106	LOC106731356	3.58	2.70E-04	0.041226	Increased
gene-LOC102	LOC102449400	-4.73	2.71E-04	0.041226	Decreased
gene-PTP4A3	PTP4A3	-4.66	2.74E-04	0.041251	Decreased
gene-CDH13	CDH13	-4.95	2.78E-04	0.041612	Decreased
gene-LOC102	LOC102459272	2.03	2.83E-04	0.041612	Increased
gene-PDZRN3	PDZRN3	-4.32	2.85E-04	0.041612	Decreased
gene-LOC102	LOC102457518	-1.85	2.86E-04	0.041612	Decreased
gene-PRLR	PRLR	-2.98	2.87E-04	0.041612	Decreased
gene-MYL3	MYL3	-7.87	2.90E-04	0.041612	Decreased
gene-TTC39C	TTC39C	-1.82	2.91E-04	0.041612	Decreased
gene-MYL2	MYL2	-9.76	2.94E-04	0.041799	Decreased
gene-LOC102	LOC102462213	-2.49	3.00E-04	0.042288	Decreased
gene-KCNJ2	KCNJ2	-5.80	3.05E-04	0.042632	Decreased
gene-CMYA5	CMYA5	-8.69	3.09E-04	0.042654	Decreased
gene-NR4A1	NR4A1	-3.47	3.09E-04	0.042654	Decreased
gene-LOC102	LOC102461259	5.79	3.24E-04	0.044351	Increased
gene-LOC102	LOC102450815	3.58	3.30E-04	0.044904	Increased
gene-LOC102	LOC102452796	-9.36	3.37E-04	0.045515	Decreased
gene-RBM20	RBM20	-9.32	3.43E-04	0.045729	Decreased
gene-LOC102	LOC102447557	4.37	3.43E-04	0.045729	Increased
gene-COL20A	COL20A1	-7.97	3.49E-04	0.045944	Decreased
gene-SLC2A1	SLC2A12	-10.06	3.51E-04	0.045944	Decreased
gene-A4GALT	A4GALT	-3.97	3.52E-04	0.045944	Decreased
gene-LOC102	LOC102446622	-8.37	3.56E-04	0.046235	Decreased
gene-MYADML	MYADML2	-9.92	3.68E-04	0.047445	Decreased
gene-PERM1	PERM1	-3.46	3.79E-04	0.048528	Decreased
gene-LOC102	LOC102450564	2.65	3.87E-04	0.049125	Increased
gene-GCA	GCA	-1.70	3.93E-04	0.049125	Decreased
gene-LOC112	LOC112545587	4.75	3.93E-04	0.049125	Increased
gene-EPDR1	EPDR1	-3.71	3.94E-04	0.049125	Decreased
gene-SHISA4	SHISA4	-3.77	4.02E-04	0.049813	Decreased
