# Supplementary material for: Liver Antioxidant, Transcriptomic and Metabolomic Responses to Heatwaves in an Aquatic Turtle Species, Pelodiscus sinensis
Source: Animals (Basel). 2026 Jun 17;16(12):1870. doi: 10.3390/ani16121870 (PMC13295678; doi:10.3390/ani16121870)
Supplement: Supplementary file 1 [file animals-16-01870-s001.zip › Supplementary Table S3.pdf]

Single HW *vs* **CTRL**

KEGG ID	Description	GeneRatio	*P* value	*P* adj	Count	Classification	Classification	Gene ID
ko05410	Hypertrophi	45/879	8.18E-23	3.24E-20	45	Human Dis	Cardiovas	gene-ACTA
ko05414	Dilated car	46/879	8.93E-22	1.77E-19	46	Human Dis	Cardiovas	gene-ACTA
ko04260	Cardiac mus	34/879	4.16E-16	5.50E-14	34	Organisma	Circulato	gene-ACTA
ko04510	Focal adhes	54/879	2.29E-13	2.26E-11	54	Cellular	Cellular	gene-ACTA
ko04261	Adrenergic	42/879	5.04E-12	3.93E-10	42	Organisma	Circulato	gene-ACTA
ko04812	Cytoskeleto	78/879	6.36E-12	3.93E-10	78	Brite Hie	Protein f	gene-ACTA
ko04810	Regulation	53/879	6.95E-12	3.93E-10	53	Cellular	Cell moti	gene-ACTA
ko00536	Glycosamino	47/879	1.59E-09	7.86E-08	47	Brite Hie	Protein f	gene-BMP5
ko04512	ECM-recepto	28/879	2.68E-09	1.18E-07	28	Environme	Signaling	gene-CHAD
ko05146	Amoebiasis	36/879	3.03E-09	1.20E-07	36	Human Dis	Infectio	gene-ACTN
ko04147	Exosome	135/879	2.59E-08	8.94E-07	135	Brite Hie	Protein f	gene-ABCC
ko05412	Arrhythmoge	24/879	2.71E-08	8.94E-07	24	Human Dis	Cardiovas	gene-ACTA
ko04270	Vascular sm	31/879	1.38E-07	4.21E-06	31	Organisma	Circulato	gene-ACTA
ko04151	PI3K-Akt si	54/879	1.50E-07	4.23E-06	54	Environme	Signal tr	gene-CHAD
ko00010	Glycolysis /	17/879	2.09E-07	5.52E-06	17	Metabolis	Carbohydr	gene-ALDH
ko05200	Pathways in	73/879	3.95E-07	9.77E-06	73	Human Dis	Cancer: c	gene-CAMK
ko04022	cGMP-PKG si	34/879	4.40E-07	1.03E-05	34	Environme	Signal tr	gene-ATP2
ko04530	Tight junct	38/879	5.42E-07	1.19E-05	38	Cellular	Cellular	gene-ACTA
ko05205	Proteoglyca	44/879	1.24E-06	2.59E-05	44	Human Dis	Cancer: c	gene-ACTA
ko04640	Hematopoiet	22/879	1.56E-06	3.07E-05	22	Organisma	Immune sy	gene-CSF1
ko04933	AGE-RAGE si	26/879	1.63E-06	3.07E-05	26	Human Dis	Endocrine	gene-COL1
ko05418	Fluid shear	30/879	3.15E-06	5.67E-05	30	Human Dis	Cardiovas	gene-ACTA
ko04360	Axon guidan	34/879	7.57E-06	0.00013	34	Organisma	Developme	gene-ABL1
ko04090	CD molecule	71/879	8.57E-06	0.000141	71	Brite Hie	Protein f	gene-ADAM
ko05416	Viral myoca	20/879	1.19E-05	0.000188	20	Human Dis	Cardiovas	gene-ACTA
ko00535	Proteoglyca	17/879	1.57E-05	0.000238	17	Brite Hie	Protein f	gene-CD74
ko04020	Calcium sig	37/879	1.71E-05	0.000251	37	Environme	Signal tr	gene-ADOR
ko04919	Thyroid hor	29/879	1.78E-05	0.000251	29	Organisma	Endocrine	gene-ACTA
ko04974	Protein dig	25/879	1.84E-05	0.000252	25	Organisma	Digestive	gene-COL1
ko04921	Oxytocin si	30/879	2.63E-05	0.000347	30	Organisma	Endocrine	gene-ACTA
ko04670	Leukocyte t	25/879	3.10E-05	0.000396	25	Organisma	Immune sy	gene-ACTA
ko04371	Apelin sign	25/879	5.61E-05	0.000694	25	Environme	Signal tr	gene-ACTA
ko04145	Phagosome	26/879	7.01E-05	0.000841	26	Cellular	Transport	gene-ACTA
ko04930	Type II dia	14/879	7.82E-05	0.000911	14	Human Dis	Endocrine	gene-ABCC
ko04010	MAPK signal	41/879	0.000103	0.001162	41	Environme	Signal tr	gene-CACN
ko04514	Cell adhesi	26/879	0.000129	0.001423	26	Environme	Signaling	gene-CADM
ko04926	Relaxin sig	24/879	0.000137	0.001466	24	Organisma	Endocrine	gene-ACTA
ko03110	Chaperones	30/879	0.000194	0.002017	30	Brite Hie	Protein f	gene-CRTA
ko05202	Transcripti	36/879	0.000202	0.002053	36	Human Dis	Cancer: c	gene-CDKN
ko05140	Leishmanias	16/879	0.00029	0.002872	16	Human Dis	Infectio	gene-EEF1
ko04611	Platelet ac	27/879	0.000356	0.003434	27	Organisma	Immune sy	gene-ACTA
ko04040	Ion channel	35/879	0.000445	0.0042	35	Brite Hie	Protein f	gene-ANO5
ko04380	Osteoclast	20/879	0.000627	0.005777	20	Organisma	Developme	gene-CALC
ko05152	Tuberculosi	27/879	0.000671	0.006042	27	Human Dis	Infectio	gene-CAMK
ko00620	Pyruvate me	10/879	0.00072	0.006339	10	Metabolis	Carbohydr	gene-LDH-
ko05415	Diabetic ca	30/879	0.000785	0.006759	30	Human Dis	Cardiovas	gene-ATP2
ko04515	Cell adhesi	23/879	0.000906	0.007633	23	Brite Hie	Protein f	gene-CADM
ko05130	Pathogenic	32/879	0.001159	0.009564	32	Human Dis	Infectio	gene-ACTA
ko04066	HIF-1 signa	19/879	0.001235	0.009978	19	Environme	Signal tr	gene-CAMK
ko05230	Central car	14/879	0.001549	0.012271	14	Human Dis	Cancer: c	gene-FLT1
ko99992	Structural	9/879	0.001817	0.01407	9	Not Inclu	Unclassif	gene-JPH2
ko04713	Circadian e	17/879	0.001883	0.01407	17	Organisma	Environme	gene-CAMK

ko05222	Small cell	17/879	0.001883	0.01407	17	Human Dis	Cancer: s	gene-CDC2
ko04725	Cholinergic	19/879	0.002154	0.015794	19	Organisma	Nervous s	gene-BCHE
ko04990	Domain-conta	22/879	0.002361	0.017	22	Brite Hie	Protein f	gene-ADIP
ko05100	Bacterial in	18/879	0.002781	0.019667	18	Human Dis	Infectiou	gene-ACTA
ko04015	Rap1 signal	31/879	0.003401	0.023626	31	Environme	Signal tr	gene-ACTA
ko04972	Pancreatic s	19/879	0.004125	0.028167	19	Organisma	Digestive	gene-AMY2
ko00051	Fructose and	7/879	0.004473	0.03002	7	Metabolis	Carbohydr	gene-HK1/
ko00330	Arginine and	9/879	0.004854	0.032034	9	Metabolis	Amino aci	gene-ARG2
ko05150	Staphylococc	12/879	0.006156	0.039625	12	Human Dis	Infectiou	gene-ITGE
ko00521	Streptomycin	4/879	0.006204	0.039625	4	Metabolis	Biosynthe	gene-HK1/
ko04911	Insulin secr	15/879	0.006503	0.040874	15	Organisma	Endocrine	gene-ABCC
ko05165	Human papill	38/879	0.007185	0.044457	38	Human Dis	Infectiou	gene-CHAD
ko05144	Malaria	11/879	0.008732	0.053196	11	Human Dis	Infectiou	gene-GYPC
ko05133	Pertussis	15/879	0.01005	0.060302	15	Human Dis	Infectiou	gene-CFL2
ko04091	Lectins	20/879	0.010354	0.061199	20	Brite Hie	Protein f	gene-CLEC
ko04060	Cytokine-cy	23/879	0.011046	0.064329	23	Environme	Signaling	gene-BMP5
ko05323	Rheumatoid a	12/879	0.011276	0.064473	12	Human Dis	Immune di	gene-CXCL
ko04610	Complement a	18/879	0.011397	0.064473	18	Organisma	Immune sy	gene-F13A
ko05134	Legionellos	13/879	0.011826	0.065377	13	Human Dis	Infectiou	gene-CDC2
ko99995	Signaling pr	14/879	0.01216	0.065377	14	Not Inclu	Unclassif	gene-AKAP
ko04054	Pattern reco	12/879	0.012217	0.065377	12	Brite Hie	Protein f	gene-CLEC
ko04370	VEGF signal	12/879	0.012217	0.065377	12	Environme	Signal tr	gene-CRYA
ko04971	Gastric acid	15/879	0.01316	0.069487	15	Organisma	Digestive	gene-ACTA
ko04052	Cytokines an	18/879	0.013554	0.070626	18	Brite Hie	Protein f	gene-BMP5
ko04131	Membrane tra	127/879	0.014562	0.074285	127	Brite Hie	Protein f	gene-ACTN
ko04728	Dopaminergic	19/879	0.014751	0.074285	19	Organisma	Nervous s	gene-ARNT
ko05417	Lipid and a	29/879	0.014819	0.074285	29	Human Dis	Cardiovas	gene-CAMK
ko05020	Prion diseas	27/879	0.015179	0.0744	27	Human Dis	Neurodege	gene-CACN
ko05131	Shigellosis	32/879	0.015218	0.0744	32	Human Dis	Infectiou	gene-ACTA
ko00680	Methane meta	5/879	0.015687	0.075754	5	Metabolis	Energy me	gene-ENO3
ko04964	Proximal tub	6/879	0.017618	0.084059	6	Organisma	Excretory	gene-AQP1
ko04970	Salivary sec	15/879	0.018046	0.085074	15	Organisma	Digestive	gene-AMY2
ko04925	Aldosterone	14/879	0.019379	0.090285	14	Organisma	Endocrine	gene-CAMK
ko01001	Protein kina	47/879	0.02227	0.102545	47	Brite Hie	Protein f	gene-ALPK
ko04141	Protein proc	20/879	0.023397	0.106497	20	Genetic I	Folding,	gene-CAPN
ko04922	Glucagon sig	14/879	0.024749	0.11137	14	Organisma	Endocrine	gene-CAMK
ko04350	TGF-beta sig	13/879	0.025116	0.111753	13	Environme	Signal tr	gene-BMP5
ko04931	Insulin res	15/879	0.028593	0.125031	15	Human Dis	Endocrine	gene-CREB
ko00030	Pentose phos	5/879	0.028732	0.125031	5	Metabolis	Carbohydr	gene-LOC1
ko04612	Antigen proc	7/879	0.030343	0.130609	7	Organisma	Immune sy	gene-CD74
ko05221	Acute myelo	11/879	0.030799	0.131146	11	Human Dis	Cancer: s	gene-CSF1
ko05135	Yersinia in	21/879	0.03168	0.133462	21	Human Dis	Infectiou	gene-ACTA
ko00524	Neomycin, ka	2/879	0.032563	0.135736	2	Metabolis	Biosynthe	gene-HK1/
ko04929	GnRH secret	9/879	0.034585	0.142663	9	Organisma	Endocrine	gene-KCNJ
ko05215	Prostate can	14/879	0.038685	0.157929	14	Human Dis	Cancer: s	gene-CREB
ko00052	Galactose me	6/879	0.044064	0.178055	6	Metabolis	Carbohydr	gene-HK1/
ko05145	Toxoplasmos	13/879	0.04468	0.178721	13	Human Dis	Infectiou	gene-HSPA
ko01521	EGFR tyrosin	11/879	0.047775	0.189082	11	Human Dis	Drug resi	gene-EGF/
ko04062	Chemokine s	20/879	0.048226	0.189082	20	Organisma	Immune sy	gene-CX3C
ko04711	Circadian rh	3/879	0.049132	0.190749	3	Organisma	Environme	gene-ARNT
ko04658	Th1 and Th2	10/879	0.051993	0.199895	10	Organisma	Immune sy	gene-EGF/
ko00500	Starch and s	6/879	0.053225	0.202665	6	Metabolis	Carbohydr	gene-AMY2
ko00710	Carbon fixa	4/879	0.054382	0.205099	4	Metabolis	Energy me	gene-LOC1
ko04666	Fc gamma R-m	14/879	0.05748	0.214736	14	Organisma	Immune sy	gene-BIN1

ko04910	Insulin sig	16/879	0.058732	0.217363	16	Organisma	Endocrine	gene-CBL/
ko00660	C5-Branched	1/879	0.060717	0.222629	1	Metabolis	Carbohydr	gene-ACOI
ko04659	Th17 cell d	12/879	0.063606	0.231083	12	Organisma	Immune sy	gene-FOS/
ko04550	Signaling p	17/879	0.067689	0.243679	17	Cellular	Cellular	gene-FLT1
ko04726	Serotonergi	16/879	0.072065	0.257097	16	Organisma	Nervous s	gene-CACN
ko04915	Estrogen si	15/879	0.073666	0.260462	15	Organisma	Endocrine	gene-CREE
ko05206	MicroRNAs i	22/879	0.07451	0.261114	22	Human Dis	Cancer: c	gene-EGF/
ko04361	Axon regene	14/879	0.07835	0.272164	14	Organisma	Developme	gene-ADAM
ko04613	Neutrophil	22/879	0.079377	0.273332	22	Organisma	Immune sy	gene-ACTA
ko04928	Parathyroid	14/879	0.085108	0.290543	14	Organisma	Endocrine	gene-CREE
ko04540	Gap junctio	12/879	0.088544	0.299686	12	Cellular	Cellular	gene-EGF/
ko04050	Cytokine re	11/879	0.089767	0.300642	11	Brite Hie	Protein f	gene-CSF3
ko04912	GnRH signal	13/879	0.090695	0.300642	13	Organisma	Endocrine	gene-CAMK
ko04621	NOD-like re	18/879	0.091104	0.300642	18	Organisma	Immune sy	gene-HSP9
ko00410	beta-Alanin	5/879	0.092843	0.303849	5	Metabolis	Metabolis	gene-ALDH
ko04973	Carbohydrat	7/879	0.098134	0.317038	7	Organisma	Digestive	gene-AMY2
ko04520	Adherens ju	13/879	0.098474	0.317038	13	Cellular	Cellular	gene-ACTA
ko04730	Long-term d	10/879	0.099775	0.318162	10	Organisma	Nervous s	gene-CACN
ko03320	PPAR signal	9/879	0.10043	0.318162	9	Organisma	Endocrine	gene-ACSL
ko05032	Morphine ad	10/879	0.109629	0.344549	10	Human Dis	Substance	gene-CACN
ko04630	JAK-STAT si	14/879	0.115741	0.351175	14	Environme	Signal tr	gene-CSF3
ko04920	Adipocytoki	9/879	0.116642	0.351175	9	Organisma	Endocrine	gene-ACSL
ko00966	Glucosinola	1/879	0.117751	0.351175	1	Metabolis	Biosynthe	gene-BCAT
ko02026	Biofilm for	1/879	0.117751	0.351175	1	Cellular	Cellular	gene-PYGM
ko00533	Glycosamino	3/879	0.117945	0.351175	3	Metabolis	Glycan bi	gene-B3GN
ko00604	Glycosphing	3/879	0.117945	0.351175	3	Metabolis	Glycan bi	gene-SLC3
ko05310	Asthma	3/879	0.117945	0.351175	3	Human Dis	Immune di	gene-FCER
ko04924	Renin secre	9/879	0.122347	0.361564	9	Organisma	Endocrine	gene-AQP1
ko05224	Breast canc	15/879	0.124025	0.363807	15	Human Dis	Cancer: s	gene-EGF/
ko00537	Glycosylpho	10/879	0.136578	0.397683	10	Brite Hie	Protein f	gene-ART1
ko04024	cAMP signal	24/879	0.138329	0.399841	24	Environme	Signal tr	gene-ADOR
ko99980	Enzymes wit	22/879	0.139739	0.400989	22	Not Inclu	Unclassif	gene-APOE
ko00910	Nitrogen me	3/879	0.146014	0.415981	3	Metabolis	Energy me	gene-CA4/
ko04625	C-type lect	13/879	0.148221	0.419255	13	Organisma	Immune sy	gene-CLEC
ko04213	Longevity r	7/879	0.153609	0.431411	7	Organisma	Aging	gene-CRYA
ko00240	Pyrimidine	7/879	0.1614	0.450101	7	Metabolis	Nucleotid	gene-AK1/
ko02024	Quorum sens	2/879	0.162561	0.450169	2	Cellular	Cellular	gene-ACSL
ko04976	Bile secret	12/879	0.165217	0.454348	12	Organisma	Digestive	gene-AQP1
ko04727	GABAergic s	9/879	0.173118	0.472791	9	Organisma	Nervous s	gene-CACN
ko04710	Circadian r	4/879	0.184794	0.501222	4	Organisma	Environme	gene-ARNT
ko04152	AMPK signal	12/879	0.188406	0.50533	12	Environme	Signal tr	gene-ADIP
ko05142	Chagas dise	11/879	0.19083	0.50533	11	Human Dis	Infectiou	gene-FOS/
ko04013	MAPK signal	13/879	0.191413	0.50533	13	Environme	Signal tr	gene-DOK2
ko04750	Inflammator	13/879	0.191413	0.50533	13	Organisma	Sensory s	gene-CAMK
ko04745	Phototransd	6/879	0.200975	0.527059	6	Organisma	Sensory s	gene-ACTA
ko04061	Viral prote	7/879	0.20285	0.528477	7	Environme	Signaling	gene-CSF1
ko00340	Histidine m	3/879	0.207472	0.534439	3	Metabolis	Amino aci	gene-ALDH
ko04390	Hippo signa	17/879	0.207838	0.534439	17	Environme	Signal tr	gene-ACTA
ko00260	Glycine, se	4/879	0.210162	0.53693	4	Metabolis	Amino aci	gene-BPGM
ko04014	Ras signali	21/879	0.212399	0.539167	21	Environme	Signal tr	gene-CSF1
ko01007	Amino acid	6/879	0.220456	0.555531	6	Brite Hie	Protein f	gene-AARS
ko99976	Replication	1/879	0.221651	0.555531	1	Not Inclu	Unclassif	gene-NEUR
ko00520	Amino sugar	5/879	0.229039	0.563186	5	Metabolis	Carbohydr	gene-HK1/
ko00730	Thiamine me	2/879	0.230254	0.563186	2	Metabolis	Metabolis	gene-AK1/

ko02020	Two-componen	2/879	0.230254	0.563186	2	Environme	Signal t	gene-ME1/
ko01009	Protein pho	40/879	0.230394	0.563186	40	Brite Hie	Protein f	gene-CENF
ko04392	Hippo signa	4/879	0.236436	0.574408	4	Environme	Signal t	gene-LOC1
ko04657	IL-17 signa	9/879	0.23978	0.578982	9	Organisma	Immune sy	gene-FOS/
ko04668	TNF signali	11/879	0.244439	0.586654	11	Environme	Signal t	gene-CREE
ko05321	Inflammator	5/879	0.251988	0.599075	5	Human Dis	Immune di	gene-IL2F
ko05031	Amphetamine	8/879	0.25264	0.599075	8	Human Dis	Substance	gene-CAMK
ko05226	Gastric can	16/879	0.268586	0.633096	16	Human Dis	Cancer: s	gene-EGF/
ko04966	Collecting	3/879	0.273456	0.640673	3	Organisma	Excretory	gene-CA4/
ko01002	Peptidases	49/879	0.275036	0.640673	49	Brite Hie	Protein f	gene-ADAM
ko04214	Apoptosis -	8/879	0.278879	0.645824	8	Cellular	Cell grow	gene-CDC2
ko04218	Cellular se	15/879	0.288438	0.660124	15	Cellular	Cell grow	gene-CAPN
ko00640	Propanoate	3/879	0.290326	0.660124	3	Metabolis	Carbohydr	gene-LDH-
ko02010	ABC transpo	4/879	0.290953	0.660124	4	Environme	Membrane	gene-ABCC
ko05022	Pathways of	37/879	0.291721	0.660124	37	Human Dis	Neurodege	gene-ASB1
ko00230	Purine meta	12/879	0.293886	0.661244	12	Metabolis	Nucleotic	gene-AK1/
ko99973	Transcripti	2/879	0.299353	0.669664	2	Not Inclu	Unclassif	gene-EFNE
ko04936	Alcoholic l	12/879	0.301011	0.669664	12	Human Dis	Endocrine	gene-ADIF
ko05132	Salmonella	22/879	0.303219	0.670809	22	Human Dis	Infectiou	gene-ACTA
ko00770	Pantothenat	3/879	0.307266	0.675985	3	Metabolis	Metabolis	gene-BCAT
ko04217	Necroptosis	14/879	0.310052	0.676543	14	Cellular	Cell grow	gene-CAMK
ko00290	Valine, leu	1/879	0.313327	0.676543	1	Metabolis	Amino aci	gene-BCAT
ko00627	Aminobenzoa	1/879	0.313327	0.676543	1	Metabolis	Xenobioti	gene-LOC1
ko05217	Basal cell	6/879	0.314353	0.676543	6	Human Dis	Cancer: s	gene-FZD4
ko05017	Spinocerebe	13/879	0.319683	0.684295	13	Human Dis	Neurodege	gene-ATP2
ko00532	Glycosamino	3/879	0.324239	0.690316	3	Metabolis	Glycan bi	gene-CHST
ko05235	PD-L1 expre	9/879	0.330946	0.698465	9	Human Dis	Cancer: c	gene-EGF/
ko05167	Kaposi sarc	17/879	0.331595	0.698465	17	Human Dis	Infectiou	gene-FOS/
ko05231	Choline met	10/879	0.336453	0.70495	10	Human Dis	Cancer: c	gene-EGF/
ko04072	Phospholipa	15/879	0.339448	0.70666	15	Environme	Signal t	gene-EGF/
ko05010	Alzheimer d	28/879	0.344676	0.70666	28	Human Dis	Neurodege	gene-ADAM
ko00670	One carbon	2/879	0.344947	0.70666	2	Metabolis	Metabolis	gene-ALDH
ko02044	Secretion s	2/879	0.344947	0.70666	2	Brite Hie	Protein f	gene-SEC3
ko05143	African try	4/879	0.346912	0.70666	4	Human Dis	Infectiou	gene-LOC1
ko05207	Chemical ca	16/879	0.354383	0.70666	16	Human Dis	Cancer: c	gene-CACN
ko00780	Biotin meta	1/879	0.355037	0.70666	1	Metabolis	Metabolis	gene-SIM2
ko02048	Prokaryotic	1/879	0.355037	0.70666	1	Brite Hie	Protein f	gene-EARS
ko99987	Cofactor me	1/879	0.355037	0.70666	1	Not Inclu	Unclassif	gene-COQ1
ko01004	Lipid biosy	7/879	0.355114	0.70666	7	Brite Hie	Protein f	gene-ACSL
ko04917	Prolactin s	7/879	0.365172	0.717435	7	Organisma	Endocrine	gene-FOS/
ko05218	Melanoma	7/879	0.365172	0.717435	7	Human Dis	Cancer: s	gene-EGF/
ko00220	Arginine bi	2/879	0.367393	0.717435	2	Metabolis	Amino aci	gene-ARG2
ko04310	Wnt signali	13/879	0.369038	0.717435	13	Environme	Signal t	gene-CAMK
ko04144	Endocytosis	21/879	0.371415	0.717435	21	Cellular	Transport	gene-BIN1
ko04064	NF-kappa B	9/879	0.374388	0.717435	9	Environme	Signal t	gene-CXCL
ko00760	Nicotinate	4/879	0.375023	0.717435	4	Metabolis	Metabolis	gene-LOC1
ko00970	Aminoacyl-t	4/879	0.375023	0.717435	4	Genetic I	Translati	gene-AARS
ko05161	Hepatitis B	14/879	0.377685	0.719055	14	Human Dis	Infectiou	gene-CDC2
ko05219	Bladder can	6/879	0.3801	0.720189	6	Human Dis	Cancer: s	gene-DAPK
ko04720	Long-term p	7/879	0.385339	0.723196	7	Organisma	Nervous s	gene-CAMK
ko05163	Human cytom	19/879	0.387704	0.724198	19	Human Dis	Infectiou	gene-CREE
ko03060	Protein exp	2/879	0.389531	0.724198	2	Genetic I	Folding,	gene-HSPA
ko04724	Glutamaterg	10/879	0.394022	0.726092	10	Organisma	Nervous s	gene-CACN
ko00960	Tropane, pi	1/879	0.394217	0.726092	1	Metabolis	Biosynthe	gene-LOC1

ko04623	Cytosolic DI	5/879	0.409299	0.747161	5	Organisma	Immune sy	gene-IRF7
ko00430	Taurine and	2/879	0.411316	0.747161	2	Metabolis	Metabolis	gene-BAAT
ko04626	Plant-patho	2/879	0.411316	0.747161	2	Organisma	Environme	gene-HSP9
ko04962	Vasopressin-	4/879	0.416944	0.753926	4	Organisma	Excretory	gene-AQP1
ko04111	Cell cycle -	6/879	0.424134	0.763442	6	Cellular	Cell grov	gene-CDC2
ko00930	Caprolactam	1/879	0.431018	0.772322	1	Metabolis	Xenobioti	gene-LOC1
ko00564	Glycerophos	9/879	0.435819	0.774256	9	Metabolis	Lipid met	gene-BCHE
ko00062	Fatty acid (	3/879	0.441372	0.774256	3	Metabolis	Lipid met	gene-BAAT
ko01523	Antifolate r	3/879	0.441372	0.774256	3	Human Dis	Drug resi	gene-LOC1
ko00510	N-Glycan bi	4/879	0.444541	0.774256	4	Metabolis	Glycan bi	gene-ALG1
ko00561	Glycerolipi	5/879	0.445736	0.774256	5	Metabolis	Lipid met	gene-CEL/
ko00983	Drug metabo	5/879	0.445736	0.774256	5	Metabolis	Xenobioti	gene-AK1/
ko04662	B cell rece	7/879	0.445784	0.774256	7	Organisma	Immune sy	gene-DAPP
ko05162	Measles	10/879	0.452034	0.778001	10	Human Dis	Infectiou	gene-CDC2
ko00511	Other glyca	2/879	0.453678	0.778001	2	Metabolis	Glycan bi	gene-NEU3
ko04916	Melanogenes	8/879	0.454576	0.778001	8	Organisma	Endocrine	gene-CAMK
ko04918	Thyroid horm	6/879	0.456881	0.778001	6	Organisma	Endocrine	gene-CREB
ko04622	RIG-I-like r	5/879	0.457763	0.778001	5	Organisma	Immune sy	gene-DDX3
ko04723	Retrograde (	10/879	0.460285	0.778943	10	Organisma	Nervous s	gene-CACN
ko04935	Growth horm	10/879	0.468516	0.787886	10	Organisma	Endocrine	gene-CREB
ko00600	Sphingolipi	5/879	0.469716	0.787886	5	Metabolis	Lipid met	gene-CERS
ko00592	alpha-Linol	3/879	0.473528	0.787886	3	Metabolis	Lipid met	gene-LOC1
ko01040	Biosynthesi	3/879	0.473528	0.787886	3	Metabolis	Lipid met	gene-BAAT
ko00565	Ether lipid	5/879	0.481585	0.793916	5	Metabolis	Lipid met	gene-LOC1
ko04391	Hippo signa	8/879	0.482309	0.793916	8	Environme	Signal tr	gene-ACTA
ko05166	Human T-cel	16/879	0.483166	0.793916	16	Human Dis	Infectiou	gene-CCNE
ko04913	Ovarian ste	6/879	0.499789	0.817837	6	Organisma	Endocrine	gene-BAAT
ko04215	Apoptosis -	3/879	0.504808	0.822651	3	Cellular	Cell grov	gene-CDC2
ko00513	Various typ	4/879	0.511519	0.830171	4	Metabolis	Glycan bi	gene-ALG1
ko02000	Transporter	39/879	0.515207	0.832742	39	Brite Hie	Protein f	gene-ABCC
ko04071	Sphingolipi	10/879	0.525319	0.845636	10	Environme	Signal tr	gene-CERS
ko00860	Porphyrin m	2/879	0.532833	0.851001	2	Metabolis	Metabolis	gene-BLVR
ko04950	Maturity on	3/879	0.535099	0.851001	3	Human Dis	Endocrine	gene-BHLH
ko04975	Fat digesti	3/879	0.535099	0.851001	3	Organisma	Digestive	gene-CEL/
ko04664	Fc epsilon I	6/879	0.541458	0.85767	6	Organisma	Immune sy	gene-FCER
ko01522	Endocrine re	8/879	0.545388	0.860452	8	Human Dis	Drug resi	gene-CDKN
ko04960	Aldosterone-	3/879	0.549845	0.862997	3	Organisma	Excretory	gene-IGF1
ko00120	Primary bil	2/879	0.551359	0.862997	2	Metabolis	Lipid met	gene-BAAT
ko00130	Ubiquinone a	1/879	0.557205	0.866952	1	Metabolis	Metabolis	gene-NQO1
ko04150	mTOR signal	11/879	0.558265	0.866952	11	Environme	Signal tr	gene-CLIP
ko04341	Hedgehog si	3/879	0.56431	0.872917	3	Environme	Signal tr	gene-EN2/
ko00534	Glycosamino	2/879	0.569362	0.877305	2	Metabolis	Glycan bi	gene-LOC1
ko05120	Epithelial (	6/879	0.571687	0.877472	6	Human Dis	Infectiou	gene-ADAM
ko04932	Non-alcohol	11/879	0.594726	0.906403	11	Human Dis	Endocrine	gene-ADIP
ko04722	Neurotrophi	10/879	0.595113	0.906403	10	Organisma	Nervous s	gene-ARHG
ko04330	Notch signa	4/879	0.598457	0.908003	4	Environme	Signal tr	gene-EGF/
ko04620	Toll-like re	7/879	0.607327	0.908718	7	Organisma	Immune sy	gene-FOS/
ko04742	Taste trans	7/879	0.607327	0.908718	7	Organisma	Sensory s	gene-CACN
ko04961	Endocrine an	4/879	0.610097	0.908718	4	Organisma	Excretory	gene-LOC1
ko04211	Longevity re	6/879	0.610401	0.908718	6	Organisma	Aging	gene-ADIP
ko05214	Glioma	6/879	0.610401	0.908718	6	Human Dis	Cancer: s	gene-CAMK
ko04210	Apoptosis	14/879	0.613613	0.909607	14	Cellular	Cell grov	gene-ACTA
ko04068	FoxO signal	10/879	0.617295	0.909607	10	Environme	Signal tr	gene-CCNB
ko00100	Steroid bio	2/879	0.620187	0.909607	2	Metabolis	Lipid met	gene-CEL/

ko00360	Phenylalanii	2/879	0.620187	0.909607	2	Metabolis	Amino aci	gene-ALDH
ko05212	Pancreatic (	6/879	0.629014	0.914444	6	Human Dis	Cancer: s	gene-EGF/
ko04114	Oocyte meio	8/879	0.629523	0.914444	8	Cellular	Cell grov	gene-CAMK
ko00350	Tyrosine me	3/879	0.63221	0.914444	3	Metabolis	Amino aci	gene-ALDH
ko00720	Carbon fixa	1/879	0.633131	0.914444	1	Metabolis	Energy me	gene-LOC1
ko04940	Type I diab	5/879	0.63503	0.914444	5	Human Dis	Endocrine	gene-CPE/
ko04115	p53 signali	5/879	0.644873	0.922517	5	Cellular	Cell grov	gene-CCNE
ko04110	Cell cycle	8/879	0.645296	0.922517	8	Cellular	Cell grov	gene-CCNE
ko00603	Glycosphing	2/879	0.651423	0.927926	2	Metabolis	Glycan bi	gene-A4GA
ko01006	Prenyltrans	1/879	0.655431	0.929502	1	Brite Hie	Protein f	gene-DHDD
ko04320	Dorso-ventra	3/879	0.657223	0.929502	3	Organisma	Developme	gene-ARPP
ko00650	Butanoate me	2/879	0.666258	0.938925	2	Metabolis	Carbohydr	gene-GADI
ko03250	Viral life (	3/879	0.680988	0.94838	3	Genetic I	Informati	gene-BICI
ko03310	Nuclear rec	3/879	0.680988	0.94838	3	Brite Hie	Protein f	gene-MAPK
ko05216	Thyroid can	3/879	0.680988	0.94838	3	Human Dis	Cancer: s	gene-LOC1
ko04927	Cortisol syı	5/879	0.682546	0.94838	5	Organisma	Endocrine	gene-CREE
ko05110	Vibrio chol	4/879	0.685541	0.94921	4	Human Dis	Infectiou	gene-ACTA
ko05012	Parkinson d	17/879	0.688483	0.949962	17	Human Dis	Neurodege	gene-ADOF
ko00590	Arachidonic	5/879	0.700343	0.962822	5	Metabolis	Lipid met	gene-LOC1
ko04340	Hedgehog si	3/879	0.703506	0.962822	3	Environme	Signal tr	gene-GLI2
ko04978	Mineral abs	4/879	0.705097	0.962822	4	Organisma	Digestive	gene-LOC1
ko00270	Cysteine and	3/879	0.714301	0.965713	3	Metabolis	Amino aci	gene-BCAT
ko00061	Fatty acid l	1/879	0.71453	0.965713	1	Metabolis	Lipid met	gene-ACSI
ko00450	Selenocompou	1/879	0.71453	0.965713	1	Metabolis	Metabolis	gene-TXNR
ko05210	Colorectal (	6/879	0.72914	0.976678	6	Human Dis	Cancer: s	gene-EGF/
ko00562	Inositol pho	4/879	0.732752	0.976678	4	Metabolis	Carbohydr	gene-INPP
ko04923	Regulation (	4/879	0.732752	0.976678	4	Organisma	Endocrine	gene-IGF1
ko04672	Intestinal	2/879	0.732889	0.976678	2	Organisma	Immune sy	gene-CXCI
ko00591	Linoleic ac	3/879	0.734975	0.976678	3	Metabolis	Lipid met	gene-LOC1
ko05203	Viral carcii	14/879	0.742797	0.978727	14	Human Dis	Cancer: (	gene-ACTN
ko05164	Influenza A	12/879	0.746908	0.978727	12	Human Dis	Infectiou	gene-ACTA
ko04934	Cushing syn	11/879	0.746995	0.978727	11	Human Dis	Endocrine	gene-CAMK
ko00900	Terpenoid ba	1/879	0.74819	0.978727	1	Metabolis	Metabolis	gene-DHDD
ko03000	Transcripti	61/879	0.748875	0.978727	61	Brite Hie	Protein f	gene-AEBP
ko00982	Drug metabo	3/879	0.754449	0.982769	3	Metabolis	Xenobioti	gene-ALDH
ko00040	Pentose and	1/879	0.763502	0.988923	1	Metabolis	Carbohydr	gene-LOC1
ko05160	Hepatitis C	10/879	0.764168	0.988923	10	Human Dis	Infectiou	gene-CDC2
ko01003	Glycosyltrai	14/879	0.775884	0.998084	14	Brite Hie	Protein f	gene-A4GA
ko00531	Glycosamino	1/879	0.777884	0.998084	1	Metabolis	Glycan bi	gene-HPSE
ko04012	ErbB signal	5/879	0.778808	0.998084	5	Environme	Signal tr	gene-CAMK
ko04011	MAPK signal	2/879	0.787859	1	2	Environme	Signal tr	gene-TEAD
ko03051	Proteasome	3/879	0.789927	1	3	Brite Hie	Protein f	gene-HSP9
ko00053	Ascorbate ai	1/879	0.791393	1	1	Metabolis	Carbohydr	gene-LOC1
ko03009	Ribosome bi	13/879	0.798711	1	13	Brite Hie	Protein f	gene-ATP8
ko04614	Renin-angio	4/879	0.80395	1	4	Organisma	Endocrine	gene-LOC1
ko05033	Nicotine ad	2/879	0.806901	1	2	Human Dis	Substance	gene-CACN
ko04660	T cell rece	6/879	0.808602	1	6	Organisma	Immune sy	gene-FOS/
ko05225	Hepatocellu	10/879	0.809416	1	10	Human Dis	Cancer: s	gene-ACTA
ko05170	Human immun	11/879	0.81116	1	11	Human Dis	Infectiou	gene-CCNE
ko00980	Metabolism (	3/879	0.813651	1	3	Metabolis	Xenobioti	gene-ALDH
ko00950	Isoquinolin	1/879	0.815998	1	1	Metabolis	Biosynthe	gene-LOC1
ko04080	Neuroactive	27/879	0.822465	1	27	Environme	Signaling	gene-ADOF
ko00071	Fatty acid (	2/879	0.824407	1	2	Metabolis	Lipid met	gene-ACSI
ko04979	Cholesterol	3/879	0.828175	1	3	Organisma	Digestive	gene-LOC1

ko00514	Other types	2/879	0.832614	1	2	Metabolis	Glycan bi	gene-LFN(
ko04721	Synaptic ve:	4/879	0.842263	1	4	Organisma	Nervous s	gene-CACN
ko00563	Glycosylpho:	1/879	0.847577	1	1	Metabolis	Glycan bi	gene-CLIF
ko00480	Glutathione	2/879	0.847996	1	2	Metabolis	Metabolis	gene-LOC1
ko00310	Lysine degra	3/879	0.848136	1	3	Metabolis	Amino aci	gene-MLLT
ko04146	Peroxisome	4/879	0.853551	1	4	Cellular	Transport	gene-ABCI
ko04624	Toll and Im	2/879	0.855195	1	2	Organisma	Immune sy	gene-LOC1
ko00020	Citrate cyc	1/879	0.856851	1	1	Metabolis	Carbohydr	gene-PCK1
ko05034	Alcoholism	8/879	0.869579	1	8	Human Dis	Substance	gene-ADOF
ko00199	Cytochrome I	3/879	0.871593	1	3	Brite Hie	Protein f	gene-LOC1
ko00630	Glyoxylate a	1/879	0.873742	1	1	Metabolis	Carbohydr	gene-LOC1
ko04216	Ferroptosis	2/879	0.874961	1	2	Cellular	Cell grow	gene-ACSI
ko00280	Valine, leu	2/879	0.886725	1	2	Metabolis	Amino aci	gene-BCAT
ko04914	Progesterone	4/879	0.887798	1	4	Organisma	Endocrine	gene-CCNE
ko05208	Chemical ca	11/879	0.890939	1	11	Human Dis	Cancer: (	gene-EGF/
ko05220	Chronic mye	4/879	0.911367	1	4	Human Dis	Cancer: s	gene-CBL/
ko04137	Mitophagy -	3/879	0.913003	1	3	Cellular	Transport	gene-LOC1
ko04650	Natural kil	7/879	0.91564	1	7	Organisma	Immune sy	gene-FCEF
ko00380	Tryptophan r	2/879	0.916133	1	2	Metabolis	Amino aci	gene-LOC1
ko00790	Folate bios	1/879	0.918653	1	1	Metabolis	Metabolis	gene-LOC1
ko00512	Mucin type (	1/879	0.923606	1	1	Metabolis	Glycan bi	gene-ST3(
ko03020	RNA polymera	3/879	0.927173	1	3	Genetic I	Transcrip	gene-LOC1
ko05340	Primary immu	1/879	0.928257	1	1	Human Dis	Immune di	gene-IL2F
ko04212	Longevity re	3/879	0.936375	1	3	Organisma	Aging	gene-IGF1
ko04031	GTP-binding	8/879	0.939663	1	8	Brite Hie	Protein f	gene-ARL1
ko03022	Basal trans	1/879	0.944201	1	1	Genetic I	Transcrip	gene-FUS
ko05332	Graft-versus	2/879	0.944306	1	2	Human Dis	Immune di	gene-LOC1
ko05171	Coronavirus	13/879	0.946931	1	13	Human Dis	Infectiou	gene-F13/
ko03016	Transfer RN	7/879	0.950926	1	7	Brite Hie	Protein f	gene-AARS
ko00830	Retinol meta	2/879	0.952338	1	2	Metabolis	Metabolis	gene-LOC1
ko00250	Alanine, asp	1/879	0.953789	1	1	Metabolis	Amino aci	gene-GADI
ko04140	Autophagy -	6/879	0.955518	1	6	Cellular	Transport	gene-DAPK
ko03032	DNA replica	4/879	0.956018	1	4	Brite Hie	Protein f	gene-CCNE
ko03420	Nucleotide (	1/879	0.956605	1	1	Genetic I	Replicati	gene-RAL(
ko05213	Endometrial	2/879	0.957064	1	2	Human Dis	Cancer: s	gene-EGF/
ko03018	RNA degrada	2/879	0.959256	1	2	Genetic I	Folding,	gene-ENO3
ko05030	Cocaine add	2/879	0.959256	1	2	Human Dis	Substance	gene-CREE
ko04142	Lysosome	7/879	0.960299	1	7	Cellular	Transport	gene-LAMF
ko04113	Meiosis - y	2/879	0.96134	1	2	Cellular	Cell grow	gene-CDC2
ko05014	Amyotrophic	18/879	0.961383	1	18	Human Dis	Neurodege	gene-ACTA
ko00601	Glycosphing	1/879	0.961732	1	1	Metabolis	Glycan bi	gene-A4GA
ko05169	Epstein-Bar	9/879	0.961857	1	9	Human Dis	Infectiou	gene-CDC2
ko05211	Renal cell (	2/879	0.968698	1	2	Human Dis	Cancer: s	gene-SLC2
ko04070	Phosphatidy	3/879	0.971133	1	3	Environme	Signal tr	gene-INPF
ko03008	Ribosome bi	2/879	0.971851	1	2	Genetic I	Translati	gene-MDN1
ko03029	Mitochondria	16/879	0.977437	1	16	Brite Hie	Protein f	gene-ACTA
ko01524	Platinum dru	2/879	0.980648	1	2	Human Dis	Drug resi	gene-CDC2
ko04714	Thermogenes	8/879	0.982697	1	8	Organisma	Environme	gene-ACSI
ko03012	Translation	2/879	0.983541	1	2	Brite Hie	Protein f	gene-CENF
ko05223	Non-small ce	2/879	0.985232	1	2	Human Dis	Cancer: s	gene-EGF/
ko03015	mRNA survei	2/879	0.986753	1	2	Genetic I	Translati	gene-FUS/
ko00140	Steroid horr	2/879	0.987455	1	2	Metabolis	Lipid met	gene-LOC1
ko03019	Messenger RI	21/879	0.99333	1	21	Brite Hie	Protein f	gene-ACTA
ko05330	Allograft re	1/879	0.993831	1	1	Human Dis	Immune di	gene-LOC1

ko03040	Spliceosome	3/879	0.994334	1	3	Genetic I	Transcrip	gene-CIRI
ko04120	Ubiquitin m	4/879	0.995461	1	4	Genetic I	Folding,	gene-CBL/
ko05322	Systemic lu	4/879	0.995461	1	4	Human Dis	Immune di	gene-ACTN
ko04138	Autophagy -	1/879	0.996274	1	1	Cellular	Transport	gene-LOC1
ko04121	Ubiquitin s	41/879	0.996365	1	41	Brite Hie	Protein f	gene-ASB1
ko03041	Spliceosome	13/879	0.9966	1	13	Brite Hie	Protein f	gene-CELF
ko05320	Autoimmune	1/879	0.997104	1	1	Human Dis	Immune di	gene-LOC1
ko03010	Ribosome	2/879	0.997881	1	2	Genetic I	Translati	gene-MRPI
ko03013	Nucleocytop	1/879	0.998725	1	1	Genetic I	Translati	gene-EEF1
ko05016	Huntington	12/879	0.998973	1	12	Human Dis	Neurodege	gene-ASB1
ko03021	Transcripti	9/879	0.999211	1	9	Brite Hie	Protein f	gene-ACTA
ko00190	Oxidative p	1/879	0.999278	1	1	Metabolis	Energy me	gene-LOC1
ko03037	Cilium and	11/879	0.999463	1	11	Brite Hie	Protein f	gene-ASB1
ko03036	Chromosome	45/879	0.999645	1	45	Brite Hie	Protein f	gene-ACTA
ko03011	Ribosome	2/879	0.99973	1	2	Brite Hie	Protein f	gene-MRPI
ko03400	DNA repair	9/879	0.999749	1	9	Brite Hie	Protein f	gene-LCP1
ko05168	Herpes simp	13/879	0.999818	1	13	Human Dis	Infectio	gene-CD74

Double HW ***vs*** **CTRL**

KEGG ID	Description	GeneRatio	*P* value	*P* adj	Count	Classific	Classific	Gene ID
ko05410	Hypertrophi	10/110	5.28E-08	1.20E-05	10	Human Dis	Cardiovas	gene-IGF1
ko05414	Dilated car	10/110	1.30E-07	1.48E-05	10	Human Dis	Cardiovas	gene-IGF1
ko00909	Sesquiterpe	3/110	4.27E-07	3.23E-05	3	Metabolis	Metabolis	gene-FDFT
ko04260	Cardiac mus	8/110	1.61E-06	9.15E-05	8	Organisma	Circulato	gene-LOC1
ko04371	Apelin sign	9/110	7.31E-06	0.000332	9	Environme	Signal tr	gene-MEF2
ko04812	Cytoskeleto	15/110	2.45E-05	0.000928	15	Brite Hie	Protein f	gene-CUNH
ko04270	Vascular sm	8/110	7.08E-05	0.002295	8	Organisma	Circulato	gene-LOC1
ko04261	Adrenergic	8/110	0.000198	0.005621	8	Organisma	Circulato	gene-LOC1
ko00680	Methane met	3/110	0.000869	0.020092	3	Metabolis	Energy me	gene-LOC1
ko04530	Tight junct	8/110	0.000885	0.020092	8	Cellular	Cellular	gene-HSPH
ko00030	Pentose pho	3/110	0.001351	0.027261	3	Metabolis	Carbohydr	gene-LOC1
ko04022	cGMP-PKG si	7/110	0.001441	0.027261	7	Environme	Signal tr	gene-LOC1
ko00100	Steroid bio	3/110	0.002152	0.037573	3	Metabolis	Lipid met	gene-FDFT
ko00051	Fructose an	3/110	0.002341	0.037951	3	Metabolis	Carbohydr	gene-LOC1
ko04919	Thyroid hor	6/110	0.004742	0.069588	6	Organisma	Endocrine	gene-LOC1
ko04711	Circadian r	2/110	0.004905	0.069588	2	Organisma	Environme	gene-NPAS
ko04921	Oxytocin si	6/110	0.006647	0.088757	6	Organisma	Endocrine	gene-KCNJ
ko01006	Prenyltrans	2/110	0.007222	0.091082	2	Brite Hie	Protein f	gene-FDFT
ko04810	Regulation	7/110	0.009713	0.116044	7	Cellular	Cell moti	gene-LOC1
ko04040	Ion channel	7/110	0.01054	0.11865	7	Brite Hie	Protein f	gene-CHRN
ko03110	Chaperones	6/110	0.010976	0.11865	6	Brite Hie	Protein f	gene-DNAJ
ko00010	Glycolysis /	3/110	0.013255	0.132284	3	Metabolis	Carbohydr	gene-LOC1
ko00710	Carbon fixa	2/110	0.014159	0.132284	2	Metabolis	Energy me	gene-LOC1
ko00966	Glucosinola	1/110	0.015139	0.132284	1	Metabolis	Biosynthe	gene-BCAT
ko02026	Biofilm for	1/110	0.015139	0.132284	1	Cellular	Cellular	gene-PYGM
ko04925	Aldosterone	4/110	0.015151	0.132284	4	Organisma	Endocrine	gene-KCNJ
ko04713	Circadian e	4/110	0.016394	0.136094	4	Organisma	Environme	gene-KCNJ
ko04930	Type II dia	3/110	0.016787	0.136094	3	Human Dis	Endocrine	gene-ADIP
ko04971	Gastric aci	4/110	0.0177	0.138545	4	Organisma	Digestive	gene-KCNJ
ko04141	Protein pro	5/110	0.019685	0.148953	5	Genetic I	Folding,	gene-CAPN

ko02000	Transporter:	10/110	0.023688	0.173458	10	Brite Hie	Protein f	gene-AQP4
ko04066	HIF-1 signa	4/110	0.024638	0.174773	4	Environme	Signal tr	gene-IGF1
ko04152	AMPK signal	4/110	0.02631	0.180981	4	Environme	Signal tr	gene-ADIF
ko04710	Circadian rl	2/110	0.032057	0.214027	2	Organisma	Environme	gene-NPAS
ko05202	Transcripti(	6/110	0.033435	0.216849	6	Human Dis	Cancer: c	gene-EYA4
ko00290	Valine, leu(	1/110	0.04474	0.281138	1	Metabolis	Amino aci	gene-BCAT
ko04914	Progesteron(	3/110	0.045824	0.281138	3	Organisma	Endocrine	gene-CCNE
ko05416	Viral myoca	3/110	0.053664	0.320572	3	Human Dis	Cardiovas	gene-LOC1
ko04990	Domain-conta	4/110	0.055095	0.320681	4	Brite Hie	Protein f	gene-ADIF
ko00330	Arginine an(	2/110	0.063417	0.356021	2	Metabolis	Amino aci	gene-GATM
ko04147	Exosome	16/110	0.064303	0.356021	16	Brite Hie	Protein f	gene-AK1/
ko04020	Calcium sign	5/110	0.07179	0.37914	5	Environme	Signal tr	gene-LOC1
ko04218	Cellular se	4/110	0.075032	0.37914	4	Cellular	Cell grov	gene-CAPN
ko04510	Focal adhes	5/110	0.07512	0.37914	5	Cellular	Cellular	gene-CAPN
ko04922	Glucagon si	3/110	0.07516	0.37914	3	Organisma	Endocrine	gene-LOC1
ko04114	Oocyte meio:	3/110	0.092282	0.455394	3	Cellular	Cell grov	gene-CCNE
ko04976	Bile secret	3/110	0.096796	0.467506	3	Organisma	Digestive	gene-AQP4
ko00561	Glycerolipi(	2/110	0.103885	0.474671	2	Metabolis	Lipid met	gene-LIPC
ko00983	Drug metabo	2/110	0.103885	0.474671	2	Metabolis	Xenobioti	gene-AK1/
ko04213	Longevity r(	2/110	0.106303	0.474671	2	Organisma	Aging	gene-HSPE
ko00730	Thiamine me	1/110	0.108157	0.474671	1	Metabolis	Metabolis	gene-AK1
ko04929	GnRH secret	2/110	0.108735	0.474671	2	Organisma	Endocrine	gene-KCNJ
ko04978	Mineral abs(	2/110	0.11861	0.508009	2	Organisma	Digestive	gene-LOC1
ko04010	MAPK signal	5/110	0.145031	0.577152	5	Environme	Signal tr	gene-HSPE
ko04068	FoxO signal	3/110	0.146429	0.577152	3	Environme	Signal tr	gene-CCNE
ko01001	Protein kina	7/110	0.146744	0.577152	7	Brite Hie	Protein f	gene-ALPK
ko00220	Arginine bi(	1/110	0.148098	0.577152	1	Metabolis	Amino aci	gene-NOS1
ko04115	p53 signalir	2/110	0.149438	0.577152	2	Cellular	Cell grov	gene-CCNE
ko00910	Nitrogen me	1/110	0.15458	0.577152	1	Metabolis	Energy me	gene-LOC1
ko04920	Adipocytokir	2/110	0.160047	0.577152	2	Organisma	Endocrine	gene-ADIF
ko04927	Cortisol syr	2/110	0.160047	0.577152	2	Organisma	Endocrine	gene-LOC1
ko05230	Central carl	2/110	0.160047	0.577152	2	Human Dis	Cancer: c	gene-PFKM
ko04728	Dopaminergi(	3/110	0.160858	0.577152	3	Organisma	Nervous s	gene-KCNJ
ko04924	Renin secre	2/110	0.162721	0.577152	2	Organisma	Endocrine	gene-KCNJ
ko04091	Lectins	3/110	0.171954	0.600518	3	Brite Hie	Protein f	gene-LOC1
ko04727	GABAergic s	2/110	0.184385	0.615349	2	Organisma	Nervous s	gene-KCNJ
ko04211	Longevity r(	2/110	0.187123	0.615349	2	Organisma	Aging	gene-ADIF
ko04730	Long-term d(	2/110	0.187123	0.615349	2	Organisma	Nervous s	gene-IGF1
ko04217	Necroptosis	3/110	0.188994	0.615349	3	Cellular	Cell grov	gene-CAPN
ko05130	Pathogenic l	4/110	0.189755	0.615349	4	Human Dis	Infectio	gene-LOC1
ko05146	Amoebiasis	3/110	0.192839	0.616543	3	Human Dis	Infectio	gene-HSPE
ko05412	Arrhythmoger	2/110	0.200894	0.633374	2	Human Dis	Cardiovas	gene-LOC1
ko00770	Pantothenat(	1/110	0.216775	0.674082	1	Metabolis	Metabolis	gene-BCAT
ko04964	Proximal tul	1/110	0.234533	0.717929	1	Organisma	Excretory	gene-SLC9
ko05235	PD-L1 expre:	2/110	0.239983	0.717929	2	Human Dis	Cancer: c	gene-BATF
ko00603	Glycosphing(	1/110	0.240364	0.717929	1	Metabolis	Glycan bi	gene-A4GA
ko05205	Proteoglycar	4/110	0.253932	0.748605	4	Human Dis	Cancer: c	gene-HSPE
ko00260	Glycine, se	1/110	0.257592	0.749659	1	Metabolis	Amino aci	gene-GATM
ko00062	Fatty acid (	1/110	0.263248	0.753149	1	Metabolis	Lipid met	gene-HACD
ko01040	Biosynthesi:	1/110	0.274433	0.753149	1	Metabolis	Lipid met	gene-HACD
ko04970	Salivary se(	2/110	0.2766	0.753149	2	Organisma	Digestive	gene-NOS1
ko00052	Galactose m(	1/110	0.279962	0.753149	1	Metabolis	Carbohydr	gene-PFKM
ko05131	Shigellosis	4/110	0.280812	0.753149	4	Human Dis	Infectio	gene-CAPN
ko01009	Protein pho:	6/110	0.28269	0.753149	6	Brite Hie	Protein f	gene-EYA4

ko00500	Starch and s	1/110	0.290895	0.753149	1	Metabolis	Carbohydr	gene-PYGM
ko04912	GnRH signal	2/110	0.293493	0.753149	2	Organisma	Endocrine	gene-LOC1
ko04110	Cell cycle	2/110	0.296305	0.753149	2	Cellular	Cell grov	gene-CCNE
ko04931	Insulin res	2/110	0.299115	0.753149	2	Human Dis	Endocrine	gene-PRKC
ko04960	Aldosterone	1/110	0.301664	0.753149	1	Organisma	Excretory	gene-IGF1
ko99992	Structural	1/110	0.301664	0.753149	1	Not Inclu	Unclassif	gene-JPH2
ko04666	Fc gamma R-r	2/110	0.301923	0.753149	2	Organisma	Immune sy	gene-BIN1
ko04612	Antigen proc	1/110	0.317514	0.764122	1	Organisma	Immune sy	gene-HSPE
ko04361	Axon regene	2/110	0.321523	0.764122	2	Organisma	Developme	gene-LOC1
ko04725	Cholinergic	2/110	0.321523	0.764122	2	Organisma	Nervous s	gene-KCNJ
ko04933	AGE-RAGE sig	2/110	0.324314	0.764122	2	Human Dis	Endocrine	gene-PLCD
ko04723	Retrograde e	2/110	0.327101	0.764122	2	Organisma	Nervous s	gene-KCNJ
ko00601	Glycosphingo	1/110	0.327882	0.764122	1	Metabolis	Glycan bi	gene-A4GA
ko04935	Growth horm	2/110	0.329885	0.764122	2	Organisma	Endocrine	gene-IGF1
ko04962	Vasopressin	1/110	0.338094	0.766002	1	Organisma	Excretory	gene-AQP4
ko04630	JAK-STAT sig	2/110	0.349285	0.766002	2	Environme	Signal tr	gene-FHL1
ko04750	Inflammatory	2/110	0.352042	0.766002	2	Organisma	Sensory s	gene-IGF1
ko03310	Nuclear rec	1/110	0.353124	0.766002	1	Brite Hie	Protein f	gene-NR4A
ko05216	Thyroid canc	1/110	0.353124	0.766002	1	Human Dis	Cancer: s	gene-TPM1
ko04915	Estrogen sig	2/110	0.354795	0.766002	2	Organisma	Endocrine	gene-KCNJ
ko04936	Alcoholic l	2/110	0.357544	0.766002	2	Human Dis	Endocrine	gene-ADIF
ko04080	Neuroactive	5/110	0.358313	0.766002	5	Environme	Signaling	gene-CHRN
ko99980	Enzymes with	3/110	0.364462	0.766002	3	Not Inclu	Unclassif	gene-APOE
ko00270	Cysteine and	1/110	0.367816	0.766002	1	Metabolis	Amino aci	gene-BCAT
ko00280	Valine, leuc	1/110	0.367816	0.766002	1	Metabolis	Amino aci	gene-BCAT
ko04910	Insulin sign	2/110	0.376667	0.777304	2	Organisma	Endocrine	gene-LOC1
ko04726	Serotonergic	2/110	0.390186	0.789226	2	Organisma	Nervous s	gene-KCNJ
ko04973	Carbohydrate	1/110	0.391572	0.789226	1	Organisma	Digestive	gene-LOC1
ko05322	Systemic lup	2/110	0.392874	0.789226	2	Human Dis	Immune di	gene-LOC1
ko01007	Amino acid r	1/110	0.400824	0.798132	1	Brite Hie	Protein f	gene-BCAT
ko04150	mTOR signal	2/110	0.406237	0.801877	2	Environme	Signal tr	gene-IGF1
ko04515	Cell adhesio	2/110	0.414189	0.810524	2	Brite Hie	Protein f	gene-CDH1
ko04979	Cholesterol	1/110	0.427754	0.824303	1	Organisma	Digestive	gene-LIPC
ko00240	Pyrimidine r	1/110	0.432124	0.824303	1	Metabolis	Nucleotic	gene-AK1
ko05332	Graft-versus	1/110	0.432124	0.824303	1	Human Dis	Immune di	gene-LOC1
ko03018	RNA degrada	1/110	0.457658	0.843307	1	Genetic I	Folding,	gene-PFKM
ko00562	Inositol pho	1/110	0.461802	0.843307	1	Metabolis	Carbohydr	gene-PLCD
ko05330	Allograft re	1/110	0.461802	0.843307	1	Human Dis	Immune di	gene-LOC1
ko04621	NOD-like rec	2/110	0.478272	0.843307	2	Organisma	Immune sy	gene-LOC1
ko04934	Cushing synd	2/110	0.480748	0.843307	2	Human Dis	Endocrine	gene-LOC1
ko00535	Proteoglycar	1/110	0.486014	0.843307	1	Brite Hie	Protein f	gene-LOC1
ko05150	Staphylococc	1/110	0.486014	0.843307	1	Human Dis	Infectiou	gene-LOC1
ko01002	Peptidases a	6/110	0.492476	0.843307	6	Brite Hie	Protein f	gene-CAPN
ko04940	Type I diabe	1/110	0.493842	0.843307	1	Human Dis	Endocrine	gene-LOC1
ko04614	Renin-angio	1/110	0.497712	0.843307	1	Organisma	Endocrine	gene-LOC1
ko04611	Platelet ac	2/110	0.497879	0.843307	2	Organisma	Immune sy	gene-MYLK
ko03320	PPAR signal	1/110	0.501552	0.843307	1	Organisma	Endocrine	gene-ADIF
ko04658	Th1 and Th2	1/110	0.505364	0.843307	1	Organisma	Immune sy	gene-PRKC
ko04913	Ovarian ster	1/110	0.509146	0.843307	1	Organisma	Endocrine	gene-IGF1
ko05320	Autoimmune	1/110	0.509146	0.843307	1	Human Dis	Immune di	gene-LOC1
ko01004	Lipid biosyn	1/110	0.516625	0.843307	1	Brite Hie	Protein f	gene-HACD
ko04370	VEGF signal	1/110	0.516625	0.843307	1	Environme	Signal tr	gene-HSPE
ko04917	Prolactin s	1/110	0.520322	0.843307	1	Organisma	Endocrine	gene-PRLF
ko05218	Melanoma	1/110	0.520322	0.843307	1	Human Dis	Cancer: s	gene-IGF1

ko05221	Acute myelo:	1/110	0.520322	0.843307	1	Human Dis	Cancer: s	gene-PER1
ko04146	Peroxisome	1/110	0.527632	0.843307	1	Cellular	Transport	gene-NOS1
ko04720	Long-term po	1/110	0.527632	0.843307	1	Organisma	Nervous s	gene-LOC1
ko05031	Amphetamine	1/110	0.531246	0.843307	1	Human Dis	Substance	gene-LOC1
ko05140	Leishmanias:	1/110	0.531246	0.843307	1	Human Dis	Infectiou	gene-NOS1
ko01521	EGFR tyrosin	1/110	0.545429	0.845797	1	Human Dis	Drug resi	gene-IGF1
ko05214	Glioma	1/110	0.548907	0.845797	1	Human Dis	Cancer: s	gene-IGF1
ko04210	Apoptosis	2/110	0.551594	0.845797	2	Cellular	Cell grow	gene-CAPN
ko05032	Morphine add	1/110	0.555786	0.845797	1	Human Dis	Substance	gene-KCNJ
ko05134	Legionellos:	1/110	0.555786	0.845797	1	Human Dis	Infectiou	gene-LOC1
ko04131	Membrane tra	13/110	0.570864	0.845797	13	Brite Hie	Protein f	gene-BIN1
ko00537	Glycosylpho:	1/110	0.572531	0.845797	1	Brite Hie	Protein f	gene-LOC1
ko04070	Phosphatidy:	1/110	0.579052	0.845797	1	Environme	Signal tr	gene-PLCD
ko04050	Cytokine rec	1/110	0.585475	0.845797	1	Brite Hie	Protein f	gene-PRLF
ko05206	MicroRNAs in	2/110	0.592868	0.845797	2	Human Dis	Cancer: c	gene-PRKC
ko04613	Neutrophil (	2/110	0.597046	0.845797	2	Organisma	Immune sy	gene-LOC1
ko05415	Diabetic car	2/110	0.603254	0.845797	2	Human Dis	Cardiovas	gene-LOC1
ko04659	Th17 cell d:	1/110	0.604166	0.845797	1	Organisma	Immune sy	gene-PRKC
ko04742	Taste transc	1/110	0.604166	0.845797	1	Organisma	Sensory s	gene-LOC1
ko04911	Insulin sec	1/110	0.604166	0.845797	1	Organisma	Endocrine	gene-LOC1
ko05022	Pathways of	4/110	0.610112	0.845797	4	Human Dis	Neurodege	gene-CAPN
ko04121	Ubiquitin sy	7/110	0.612405	0.845797	7	Brite Hie	Protein f	gene-ASB1
ko04391	Hippo signa:	1/110	0.613196	0.845797	1	Environme	Signal tr	gene-LOC1
ko05133	Pertussis	1/110	0.622022	0.845797	1	Human Dis	Infectiou	gene-NOS1
ko05145	Toxoplasmos:	1/110	0.622022	0.845797	1	Human Dis	Infectiou	gene-NOS1
ko03032	DNA replica:	1/110	0.62492	0.845797	1	Brite Hie	Protein f	gene-CCNF
ko04064	NF-kappa B s	1/110	0.62492	0.845797	1	Environme	Signal tr	gene-PRKC
ko05222	Small cell l	1/110	0.62492	0.845797	1	Human Dis	Cancer: s	gene-NOS1
ko04151	PI3K-Akt sig	3/110	0.626109	0.845797	3	Environme	Signal tr	gene-IGF1
ko05010	Alzheimer d:	3/110	0.627714	0.845797	3	Human Dis	Neurodege	gene-CAPN
ko04660	T cell recep	1/110	0.630649	0.845797	1	Organisma	Immune sy	gene-PRKC
ko01522	Endocrine re	1/110	0.63348	0.845797	1	Human Dis	Drug resi	gene-IGF1
ko05142	Chagas disea	1/110	0.641848	0.845797	1	Human Dis	Infectiou	gene-NOS1
ko05020	Prion diseas	2/110	0.642816	0.845797	2	Human Dis	Neurodege	gene-LOC1
ko00564	Glycerophosp	1/110	0.644594	0.845797	1	Metabolis	Lipid met	gene-LPIN
ko05215	Prostate can	1/110	0.650025	0.848022	1	Human Dis	Cancer: s	gene-IGF1
ko00536	Glycosaminog	2/110	0.666844	0.862077	2	Brite Hie	Protein f	gene-LOC1
ko04520	Adherens jun	1/110	0.668394	0.862077	1	Cellular	Cellular	gene-LIMC
ko04724	Glutamaterg:	1/110	0.673465	0.863709	1	Organisma	Nervous s	gene-LOC1
ko04024	cAMP signal:	2/110	0.689568	0.866701	2	Environme	Signal tr	gene-LOC1
ko05417	Lipid and a:	2/110	0.689568	0.866701	2	Human Dis	Cardiovas	gene-HSPH
ko04928	Parathyroid	1/110	0.69299	0.866701	1	Organisma	Endocrine	gene-MEF2
ko04625	C-type lect:	1/110	0.695348	0.866701	1	Organisma	Immune sy	gene-PRKC
ko04144	Endocytosis	2/110	0.697966	0.866701	2	Cellular	Transport	gene-BIN1
ko04972	Pancreatic s	1/110	0.709123	0.866701	1	Organisma	Digestive	gene-SLC9
ko04071	Sphingolipid	1/110	0.711358	0.866701	1	Environme	Signal tr	gene-PRKC
ko04013	MAPK signal:	1/110	0.713576	0.866701	1	Environme	Signal tr	gene-MEF2
ko00230	Purine metal	1/110	0.715777	0.866701	1	Metabolis	Nucleotic	gene-AK1
ko04974	Protein dige	1/110	0.715777	0.866701	1	Organisma	Digestive	gene-SLC9
ko04610	Complement a	1/110	0.717961	0.866701	1	Organisma	Immune sy	gene-LOC1
ko04052	Cytokines an	1/110	0.724415	0.866701	1	Brite Hie	Protein f	gene-IGF1
ko04670	Leukocyte tr	1/110	0.726533	0.866701	1	Organisma	Immune sy	gene-MYL2
ko04722	Neurotrophin	1/110	0.730722	0.866701	1	Organisma	Nervous s	gene-PRKC
ko04140	Autophagy -	1/110	0.734847	0.866701	1	Cellular	Transport	gene-PRKC

ko04926	Relaxin sigı	1/110	0.736887	0.866701	1	Organisma	Endocrine	gene-NOS1
ko04650	Natural kil	1/110	0.740918	0.866751	1	Organisma	Immune sy	gene-LOC1
ko05224	Breast canc	1/110	0.744889	0.866751	1	Human Dis	Cancer: s	gene-IGF1
ko05034	Alcoholism	1/110	0.752649	0.866751	1	Human Dis	Substance	gene-PKIA
ko05017	Spinocerebe	1/110	0.754553	0.866751	1	Human Dis	Neurodege	gene-VLDL
ko04145	Phagosome	1/110	0.763854	0.866751	1	Cellular	Transport	gene-NOS1
ko04932	Non-alcohol	1/110	0.763854	0.866751	1	Human Dis	Endocrine	gene-ADIP
ko04550	Signaling pa	1/110	0.765672	0.866751	1	Cellular	Cellular	gene-IGF1
ko04310	Wnt signali	1/110	0.767476	0.866751	1	Environme	Signal tı	gene-LOC1
ko04514	Cell adhesio	1/110	0.776292	0.869523	1	Environme	Signaling	gene-MPZ
ko04142	Lysosome	1/110	0.781421	0.869523	1	Cellular	Transport	gene-LOC1
ko05418	Fluid shear	1/110	0.781421	0.869523	1	Human Dis	Cardiovas	gene-MEF2
ko03019	Messenger RN	3/110	0.792666	0.877733	3	Brite Hie	Protein f	gene-LOC1
ko03029	Mitochondria	2/110	0.816289	0.88941	2	Brite Hie	Protein f	gene-DNAJ
ko04062	Chemokine s	1/110	0.817064	0.88941	1	Organisma	Immune sy	gene-PRKC
ko05135	Yersinia in	1/110	0.821266	0.88941	1	Human Dis	Infectiou	gene-WIPF
ko05170	Human immunc	1/110	0.822645	0.88941	1	Human Dis	Infectiou	gene-CCNE
ko04060	Cytokine-cy	1/110	0.825373	0.88941	1	Environme	Signaling	gene-PRLR
ko05152	Tuberculosis	1/110	0.826721	0.88941	1	Human Dis	Infectiou	gene-NOS1
ko05207	Chemical ca	1/110	0.835872	0.891902	1	Human Dis	Cancer: c	gene-LOC1
ko05014	Amyotrophic	2/110	0.836895	0.891902	2	Human Dis	Neurodege	gene-LOC1
ko05208	Chemical ca	1/110	0.85046	0.902123	1	Human Dis	Cancer: c	gene-PRKC
ko05166	Human T-cel	1/110	0.859463	0.907433	1	Human Dis	Infectiou	gene-CCNE
ko03009	Ribosome bic	1/110	0.863761	0.907749	1	Brite Hie	Protein f	gene-LOC1
ko03036	Chromosome a	6/110	0.874582	0.913051	6	Brite Hie	Protein f	gene-CCNE
ko01003	Glycosyltrai	1/110	0.876851	0.913051	1	Brite Hie	Protein f	gene-A4GA
ko05200	Pathways in	3/110	0.883327	0.915595	3	Human Dis	Cancer: c	gene-IGF1
ko04014	Ras signali	1/110	0.889556	0.91786	1	Environme	Signal tı	gene-IGF1
ko04015	Rap1 signal	1/110	0.904744	0.929307	1	Environme	Signal tı	gene-IGF1
ko05171	Coronavirus	1/110	0.910502	0.931009	1	Human Dis	Infectiou	gene-LOC1
ko05132	Salmonella	1/110	0.915257	0.931674	1	Human Dis	Infectiou	gene-MYL2
ko03000	Transcripti	5/110	0.920923	0.933257	5	Brite Hie	Protein f	gene-BATF
ko03400	DNA repair a	1/110	0.943555	0.951942	1	Brite Hie	Protein f	gene-PER1
ko03041	Spliceosome	1/110	0.954676	0.9589	1	Brite Hie	Protein f	gene-HSPE
ko04030	G protein-cc	1/110	0.999999	0.999999	1	Brite Hie	Protein f	gene-LOC1

Gene name
ACTA1/ATP2A3/CACNA2D1/CACNB1/CACNG1/CACNG5/CCDC141/ITGA4/ITGA7/ITGA8/ITGAV/ITGB2/LAMA2/LO
ACTA1/ATP2A3/CACNA2D1/CACNB1/CACNG1/CACNG5/CCDC141/ITGA4/ITGA7/ITGA8/ITGAV/ITGB2/LAMA2/LO
ACTA1/ATP2A3/CACNA2D1/CACNB1/CACNG1/CACNG5/LOC102445983/LOC102446383/LOC102446622/LOC1024
ACTA1/ACTN2/ACTN3/CAPN2/CAPN3/CAV1/CAV2/CAV3/CHAD/COL11A1/COL1A1/COL1A2/COL4A1/COL4A2/COL
ACTA1/ATP2A3/CACNA2D1/CACNB1/CACNG1/CACNG5/CAMK2A/CREB3L1/LOC102445983/LOC102446383/LOC10
ACTA1/ACTN2/ACTN3/ADD3/AIF1L/ASB14/CCDC141/CFL2/CLIP2/CLIP4/CUNH10orf71/DST/EHBP1L1/EML1/
ACTA1/ACTN2/ACTN3/BAIAP2/CFL2/CXCL12/DOCK2/DOCK4/EGF/F2R/FGD3/FGF16/FGF4/FGF6/FLT1/GSN/IG
BMP5/COL11A1/COL1A1/COL1A2/COL22A1/COL4A1/COL4A2/COL4A5/COL5A1/COL5A2/COL6A1/COL6A3/CXCL1
CHAD/COL11A1/COL1A1/COL1A2/COL4A1/COL4A2/COL4A5/COL5A1/COL5A2/COL6A1/COL6A3/ITGA4/ITGA7/I
ACTN2/ACTN3/ARG2/COL11A1/COL1A1/COL1A2/COL4A1/COL4A2/COL4A5/COL5A1/COL5A2/CRYAB/HSPB1/HSP
ABCC9/ACSL1/ACTA1/ACTN2/ACTN3/AK1/AK5/ANXA1/ANXA2/AQP1/ARHGDIB/BASP1/CA4/CAP2/CCDC141/CEL
ACTA1/ACTN2/ACTN3/ATP2A3/CACNA2D1/CACNB1/CACNG1/CACNG5/ITGA4/ITGA7/ITGA8/ITGAV/ITGB2/LAMA
ACTA1/ADORA2A/CALCRL/EDNRA/KCNMA1/KCNMB1/LOC102445983/LOC102446383/LOC102446622/LOC10244
CHAD/COL11A1/COL1A1/COL1A2/COL4A1/COL4A2/COL4A5/COL5A1/COL5A2/COL6A1/COL6A3/CREB3L1/CSF1R
ALDH1A3/BPGM/ENO3/HK1/LDH-A/LOC102445123/LOC102447192/LOC102447223/LOC102447912/LOC102449
CAMK2A/CBL/CDC20/COL4A1/COL4A2/COL4A5/CSF1R/CSF3R/CXCL12/DAPK2/EDNRA/EGF/F2R/FGF16/FGF4/F
ATP2A3/CREB3L1/EDNRA/IGF1R/IRS1/KCNMA1/KCNMB1/LOC102445983/LOC102446383/LOC102446622/LOC1
ACTA1/ACTN2/ACTN3/CLDN19/HCLS1/HSPA4L/HSPH1/ITGB2/JAM3/LOC102443591/LOC102445983/LOC10244
ACTA1/CAMK2A/CAV1/CAV2/CAV3/CBL/COL11A1/COL1A1/COL1A2/COL5A1/COL5A2/CRYAB/FLT1/FZD4/HCLS1
CSF1R/CSF3R/IL5RA/ITGA4/ITGA7/ITGA8/ITGAV/LOC102444204/LOC102444471/LOC102447615/LOC10244
COL11A1/COL1A1/COL1A2/COL4A1/COL4A2/COL4A5/COL5A1/COL5A2/JAK2/LOC102447978/LOC102448244/L
ACTA1/CAV1/CAV2/CAV3/CDH5/FLT1/FOS/HSP90AA1/HSP90B1/ITGA4/ITGA8/ITGAV/KDR/LOC102448244/LO
ABLIM3/BMP5/CAMK2A/CFL2/CXCL12/DPYSL2/DPYSL4/EFNB2/EPHA3/ITGB2/LOC102453431/LOC102455777/
ADAM10/ADAM8/ART1/ART5/CD109/CD74/CD99/CDH5/CLEC17A/CSF1R/CSF3R/ENG/ENTPD2/FLT1/FZD4/GYPC
ACTA1/CAV1/CAV3/ITGB2/LAMA2/LOC102445983/LOC102446383/LOC102446622/LOC102450688/LOC102454
CD74/CHAD/COL12A1/COL14A1/CRTAP/CSPG4/ECM2/LOC102447587/LOC102449267/LOC102449499/LOC1024
ADORA2A/ATP2A3/CACNA1B/CAMK2A/CAMK4/EDNRA/EGF/F2R/FGF16/FGF4/FGF6/FLT1/HTR2A/KDR/LOC10245
ACTA1/ATP2A3/EGF/ITGA4/ITGA8/ITGAV/LOC102445983/LOC102446383/LOC102446622/LOC102450688/LO
COL11A1/COL12A1/COL14A1/COL1A1/COL1A2/COL22A1/COL4A1/COL4A2/COL4A5/COL5A1/COL5A2/COL6A1/C
ACTA1/CACNA2D1/CACNB1/CACNG1/CACNG5/CAMK2A/CAMK4/FOS/KCNJ2/KCNJ3/LOC102446945/LOC10245378
ACTA1/ACTN2/ACTN3/CD99/CDH5/CLDN19/CXCL12/ITGA4/ITGB2/JAM3/LOC102444471/LOC102454753/LOC1
ACTA1/CAMK4/GNB5/GNG7/HDAC9/JAG2/LOC102450075/LOC102454459/LOC102456421/LOC102456632/MEF2
ACTA1/CLEC17A/COLEC12/ITGA4/ITGA8/ITGAV/ITGB2/LOC102443591/LOC102444471/LOC102454753/LOC1
ABCC9/ADIPOQ/CACNA1B/HK1/IGF1R/IRS1/KCNJ11/LOC102447192/LOC102453880/PKM/PRKCD/PRKCQ/SLC2
CACNA1B/CACNA2D1/CACNB1/CACNG1/CACNG5/CRYAB/CSF1R/DUSP10/DUSP26/DUSP6/EGF/FGF16/FGF4/FGF6
CADM3/CD99/CDH15/CDH5/CEL/CLDN19/ITGA4/ITGA7/ITGA8/ITGAV/ITGB2/JAM3/LOC102444471/LOC10245
ACTA1/COL11A1/COL1A1/COL1A2/COL4A1/COL4A2/COL4A5/COL5A1/COL5A2/CREB3L1/FOS/GNB5/GNG7/LOC1
CRTAP/CRYAB/DNAJA4/DNAJB9/DNAJC10/GLRX/HSP90AA1/HSP90B1/HSPA2/HSPA4L/HSPA5/HSPB1/HSPB2/HS
CDKN2C/CSF1R/DUSP6/EYA4/FEV/FLI1/FLT1/FUS/FUT8/HOXA9/ID2/IGF1R/IGFBP3/KDM6B/KDR/LOC102444
EEF1A2/FOS/ITGA4/ITGB2/JAK2/LOC102444471/LOC102448244/LOC102451319/LOC102454753/LOC102457
ACTA1/COL11A1/COL1A1/COL1A2/COL5A1/COL5A2/F2R/FCER1G/ITGB2/LOC102446945/LOC102447615/LOC1
ANO5/ANO6/CACNA1B/CACNA2D1/CACNB1/CACNG1/CACNG5/CHRNA1/CHRNB1/CHRND/CHRNE/GABRR2/KCNJ11/K
CALCRL/CAMK4/CSF1R/FOS/JAK2/LOC102444600/LOC102448244/LOC102454753/LOC102458809/MAP2K6/NC
CAMK2A/CD74/CDC20/CLEC17A/FCER1G/ITGB2/JAK2/LOC102444471/LOC102448244/LOC102448341/LOC102
LDH-A/LOC102447223/LOC102447912/LOC102460367/LOC102462297/LOC102463832/ME1/ME3/PCK1/PKM
ATP2A3/CAMK2A/COL11A1/COL1A1/COL1A2/COL5A1/COL5A2/IGF1R/IRS1/LOC102447978/LOC102454753/LO
CADM2/CADM3/CDH11/CDH13/CDH15/CDH23/CDH5/ITGA4/ITGA7/ITGA8/ITGAV/ITGB2/LOC102444471/LOC10
ACTA1/BAIAP2/CLDN19/F2R/FOS/HCLS1/ITGB2/LOC102443591/LOC102445983/LOC102446383/LOC102446
CAMK2A/EGF/ENO3/FLT1/HK1/IGF1R/KDR/LDH-A/LOC102447192/LOC102449400/LOC102454753/LOC102455
FLT1/HK1/KDR/LDH-A/LOC102447192/LOC102461694/LOC102461925/LOC102462297/PDGFRA/PDK4/PFKM/P
JPH2/JPH3/LOC102456325/PGM5/PMP22/REEP1/SSPN/TSPAN2/TSPAN9
CAMK2A/FOS/GNB5/GNG7/KCNJ2/KCNJ3/LOC102453786/LOC102453880/LOC102454459/LOC102455777/LOC1

CDC20/COL4A1/COL4A2/COL4A5/ITGA4/ITGA7/ITGA8/ITGAV/ITGB2/LAMA2/LAMA5/LAMB1/LAMC1/LOC10245
BCHE/CACNA1B/CAMK2A/CAMK4/CREB3L1/FOS/GNB5/GNG7/JAK2/KCNJ2/KCNJ3/KCNQ4/LOC102453489/LOC10
ADIPOQ/DOK2/EFEMP1/EFEMP2/EGF/FBN1/FBN2/FGD3/FHL1/FLRT2/FLRT3/HPCA/LDB3/LOC102456421/LOC1
ACTA1/CAV1/CAV2/CAV3/CBL/DOCK2/DOCK4/HCLS1/ITGA4/ITGA8/ITGAV/ITGB2/LOC102452779/LOC10245
ACTA1/ADORA2A/CSF1R/DOCK2/DOCK4/EGF/F2R/FGF16/FGF4/FGF6/FLT1/IGF1R/ITGB2/KDR/LOC102444471
AMY2A/ATP2A3/CA4/CEL/KCNMA1/LOC102445365/LOC102449893/LOC102450141/LOC102454459/LOC102455
HK1/LOC102445123/LOC102447192/LOC102447223/LOC102449400/LOC102460847/PFKM
ARG2/CARNS1/CKMT2/GAMT/GATM/LOC102464074/NOS1/OAT/P4HA3
ITGB2/LOC102444471/LOC102446475/LOC102455367/LOC102457786/LOC102462153/LOC102462321/LOC10
HK1/LOC102447192/PGM2L1/PGM5
ABCC9/CAMK2A/CREB3L1/KCNJ11/KCNMA1/KCNMB1/LOC102444244/LOC102453880/LOC102454459/LOC10245
CHAD/COL11A1/COL1A1/COL1A2/COL4A1/COL4A2/COL4A5/COL5A1/COL5A2/COL6A1/COL6A3/CREB3L1/EGF/F
GYPC/ITGB2/LOC102448244/LOC102451319/LOC102454894/LOC102462153/PECAM1/TGFB2/THBS2/TLR4/TS
CFL2/FOS/ITGA4/ITGA8/ITGAV/ITGB2/LOC102444471/LOC102446475/LOC102448244/LOC102454894/LOC1
CLEC17A/CLEC3B/COLEC12/LGALS1/LOC102445784/LOC102448870/LOC102449267/LOC102449499/LOC1024
BMP5/CSF1R/CSF3R/CX3CR1/CXCL12/GDF1/IL17D/IL2RG/IL5RA/LIFR/LOC102443642/LOC102448244/LOC1
CXCL12/FLT1/FOS/ITGB2/KDR/LOC102448244/LOC102454894/LOC102456381/LOC102461925/TGFB2/TLR4/
F13A1/F2R/ITGB2/LOC102444471/LOC102445365/LOC102446475/LOC102447429/LOC102451319/LOC10245
CDC20/EEF1A2/HSPA2/ITGB2/LOC102444471/LOC102448244/LOC102451319/LOC102454894/LOC102457786
AKAP6/CLMP/CPZ/DACT1/EMCN/GLDN/LOC102443906/MGP/OLFML2B/PODXL2/RGS16/RGS18/RGS3/RTN4RL1
CLEC17A/LOC102445784/LOC102448870/LOC102449267/LOC102449499/LOC102451408/LOC102451500/LOC
CRYAB/FLT1/HSPB1/HSPB2/HSPB6/KDR/LOC102446945/LOC102458809/LOC102461925/LOC106732455/MAPK
ACTA1/CA4/CAMK2A/KCNJ2/LOC102449893/LOC102453786/LOC102454459/LOC102455777/LOC102456632/L
BMP5/CXCL12/EFNB2/EGF/FGF16/FGF4/FGF6/GAS6/GDF1/IL17D/LOC102443642/LOC102448244/LOC10245
ACTN2/ACTN3/ADGRL3/AMPD1/ANKRD1/ANKRD23/ANXA1/ANXA2/ARHGAP11A/ARHGAP18/ARHGAP31/ARHGAP45/
ARNTL/CACNA1B/CAMK2A/CREB3L1/FOS/GNB5/GNG7/KCNJ2/KCNJ3/LOC102452890/LOC102453786/LOC10245
CAMK2A/CDC20/ERO1A/FOS/HSP90AA1/HSP90B1/HSPA2/HSPA4L/HSPA5/HSPH1/IRF7/JAK2/LOC102448244/L
CACNA1B/CAV1/CAV2/CAV3/CDC20/CREB3L1/EGF/HSPA2/HSPA5/LAMC1/LOC102443591/LOC102446475/LOC1
ACTA1/ACTN2/ACTN3/CAPN2/CAPN3/CAST/DOCK2/DOCK4/HCLS1/HK1/ITGA4/ITGA8/ITGAV/ITGB2/LOC10244
ENO3/LOC102445123/LOC102447912/LOC102449400/PFKM
AQP1/CA4/LOC102449893/LOC102462326/PCK1/SLC9A2
AMY2A/KCNMA1/LOC102448373/LOC102454459/LOC102456421/LOC102461370/LOC102462321/LOC10246232
CAMK2A/CAMK4/CREB3L1/KCNJ2/LOC102453786/LOC102453880/LOC102454459/LOC102455777/LOC1024623
ALPK2/ALPK3/BMP5/CAMK2A/CAMK4/CCDC141/CIT/CSF1R/DAPK2/EPHA3/FLT1/HCK/HSPB8/IGF1R/JAK2/KDR
CAPN2/CAPN3/CRYAB/DNAJA4/DNAJC10/ERO1A/HSP90AA1/HSP90B1/HSPA2/HSPA4L/HSPA5/HSPB1/HSPB2/HS
CAMK2A/CREB3L1/LDH-A/LOC102445123/LOC102454459/LOC102455777/LOC102462297/PCK1/PFKM/PKM/PL
BMP5/FBN1/FBN2/GDF1/ID2/MATN2/PITX1/PITX3/RGMA/RGMB/TGFB2/TGFBR2/THBS2
CREB3L1/IGF1R/IRS1/NR1H3/PCK1/PPP1R3A/PRKCD/PRKCQ/PTPRS/PYGM/SLC2A4/SOCS3/SREBF1/TBC1D1/T
LOC102445123/LOC102449400/PFKM/PGM2L1/PGM5
CD74/HSP90AA1/HSPA2/HSPA4L/HSPA5/HSPH1/LOC102458936
CSF1R/DUSP6/LOC102444471/LOC102457786/LOC102462624/LOC102463089/PER1/PER2/RARB/RUNX1/SPI1
ACTA1/BAIAP2/DOCK2/DOCK4/FOS/ITGA4/ITGA8/ITGAV/ITGB2/LOC102448244/LOC102452779/LOC1024544
HK1/LOC102447192
KCNJ11/KCNJ2/KCNJ3/LOC102444244/LOC102453786/LOC102453880/LOC102454459/LOC102455075/PLCB1
CREB3L1/EGF/FEV/FLI1/FLT1/HSP90AA1/HSP90B1/IGF1R/KDR/LOC102454339/LOC102461925/PDGFD/PDGF
HK1/LOC102447192/LOC102447223/PFKM/PGM2L1/PGM5
HSPA2/ITGA7/ITGB2/JAK2/LAMA2/LAMA5/LAMB1/LAMC1/LOC102460859/MAP2K6/NOS1/TGFB2/TLR4
EGF/FLT1/GAS6/IGF1R/JAK2/KDR/LOC102458809/LOC102461925/MERTK/PDGFD/PDGFRA
CX3CR1/CXCL12/DOCK2/DOCK4/GNB5/GNG7/HCK/JAK2/LOC102443642/LOC102454459/LOC102454894/LOC10
ARNTL/PER1/PER2
EGF/FOS/IL2RG/JAG2/JAK2/NFATC1/PPP3CA/PRKCD/PRKCQ/RUNX1
AMY2A/HK1/LOC102447192/PGM2L1/PGM5/PYGM
LOC102445123/LOC102449400/ME1/ME3
BIN1/CFL2/DOCK2/DOCK4/GSN/HCK/LOC102446945/LOC102452779/LOC106732455/LYN/MARCKS/NCF1/PRKC

CBL/HK1/IGF1R/IRS1/LOC102445123/LOC102447192/PCK1/PKM/PPP1R3A/PRKAR1B/PTPRS/PYGM/SLC2A4/S
ACOD1
FOS/HSP90AA1/IL2RG/JAK2/LOC102448244/NFATC1/PPP3CA/PRKCD/PRKCQ/RARB/RUNX1/TGFBR2
FLT1/FZD4/HOXD1/ID2/IGF1R/JAK2/JARID2/KDR/LIFR/LOC102444628/LOC102461925/MLLT11/MYF5/MYF6
CACNA1B/GNB5/GNG7/HTR2A/KCNJ2/KCNJ3/LOC102444244/LOC102446945/LOC102453786/LOC102453880/L
CREB3L1/FOS/HSP90AA1/HSP90B1/HSPA2/KCNJ2/KCNJ3/LOC102453786/LOC102454459/LOC102458809/MMP
EGF/HDAC9/IRS1/ITGA4/ITGA8/ITGAV/LOC102448341/LOC102459430/LOC102464093/MARCKS/MMP16/MSN/
ADAM10/DUSP26/DUSP6/EGF/EPHA3/IGF1R/KIF24/LOC102453880/LOC102454459/MAPKAPK3/PLCB1/PRKCD/
ACTA1/HDAC9/ITGB2/LOC102444471/LOC102447615/LOC102451319/LOC102453941/LOC102454753/LOC102
CREB3L1/FOS/LOC102448341/LOC102448373/LOC102448412/LOC102454459/LOC102455075/MAFB/MEF2A/M
EGF/HTR2A/LOC102443591/LOC102454459/LOC102458809/LOC102463494/LPAR1/PDGFD/PDGFRA/PLCB1/TU
CSF3R/CX3CR1/IL2RG/IL5RA/LIFR/LOC102457584/LOC102458090/LOC102460859/PRLR/RELT/TNFRSF19
CAMK2A/LOC102446945/LOC102453880/LOC102454459/LOC102455777/LOC102458809/LOC106732455/MAP2
HSP90AA1/IRF7/JAK2/LOC102448244/LOC102454753/LOC102454894/LOC102455367/LOC102455936/LOC10
ALDH1A3/CARNS1/DPYSL2/GADL1/LOC102464074
AMY2A/HK1/LOC102447192/LOC102453880/LOC102462326/PLCB1/SLC2A4
ACTA1/ACTN2/ACTN3/BAIAP2/IGF1R/LIMCH1/LOC102455684/LOC102456632/LOC102458809/PTPRB/PTPRM/
CACNA1B/IGF1R/LOC102446945/LOC102454459/LOC102456421/LOC102463494/LOC106732455/LYN/NOS1/P
ACSL1/ADIPOQ/FABP3/LOC102447294/LOC102448341/ME1/ME3/NR1H3/PCK1
CACNA1B/GABRR2/GNB5/GNG7/KCNJ2/KCNJ3/LOC102453786/LOC102455075/PDE2A/PDE4B
CSF3R/EGF/FHL1/IL17D/IL2RG/IL5RA/JAK2/LIFR/LOC102457584/LOC102458090/PDGFRA/PRLR/PTPRS/SO
ACSL1/ADIPOQ/IRS1/JAK2/PCK1/PRKCD/PRKCQ/SLC2A4/SOCS3
BCAT1
PYGM
B3GNT7/FUT8/ST3GAL1
SLC33A1/ST3GAL1/ST8SIA5
FCER1G/LOC102443642/LOC102463089
AQP1/EDNRA/KCNJ2/KCNMA1/LOC102453786/LOC102453880/LOC102454459/PLCB1/PPP3CA
EGF/FGF16/FGF4/FGF6/FLT1/FOS/FZD4/HEY1/IGF1R/JAG2/KDR/LOC102461925/TNFSF11/WNT5A/WNT9A
ART1/ART5/BCHE/CA4/LOC102447587/LOC102451362/LOC102453489/LOC102463142/NT5E/SPACA4
ADORA2A/ATP2A3/CAMK2A/CAMK4/CREB3L1/EDNRA/F2R/FOS/GLI2/GLI3/LOC102443591/LOC102445828/LOC
APOBEC2/ART1/ART5/BCHE/CCNB1/DHRS7C/F13A1/FLT1/KDR/LOC102443765/LOC102445544/LOC102446471
CA4/LOC102449893/LOC102453787
CLEC17A/FCER1G/IL17D/LOC102448244/LOC102449267/LOC102449499/LOC102458809/LOC102459066/MAP
CRYAB/HSPA2/HSPB1/HSPB2/HSPB6/IGF1R/IRS1
AK1/AK5/DPYSL2/ENTPD2/LOC102446326/NT5C1A/NT5E
ACSL1/GADL1
AQP1/AQP4/BAAT/CA4/LOC102443591/LOC102444244/LOC102449893/LOC102452280/LOC102459228/LOC10
CACNA1B/GABRR2/GADL1/GNB5/GNG7/KCNJ2/LOC102453786/LOC102453880/LOC102458809
ARNTL/CRY1/PER1/PER2
ADIPOQ/CREB3L1/IGF1R/IRS1/LOC102445123/PCK1/PFKM/PPP2R2C/SLC2A4/SREBF1/TBC1D1/TBC1D4
FOS/LOC102446475/LOC102448244/LOC102454459/LOC102454894/NOS1/PLCB1/PPP2R2C/TGFB2/TGFBR2/T
DOK2/DUSP10/DUSP26/DUSP6/LOC102458809/LOC112546124/LYN/MAP2K6/MAP3K15/MEF2A/MEF2C/MYF6/RU
CAMK2A/HTR2A/LOC102446945/LOC102447429/LOC102448244/LOC102454459/LOC102455777/LOC10245880
ACTA1/CAMK2A/LOC102454459/LOC102455777/LOC102456632/PLCB1
CSF1R/CX3CR1/CXCL12/IL2RG/LOC102443642/LOC102454894/LOC102460859
ALDH1A3/CARNS1/LOC102464074
ACTA1/BMP5/FZD4/GDF1/GLI2/GLI3/ID2/ITGB2/LOC102455684/LOC102456632/PPP2R2C/TEAD3/TGFB2/TG
BPGM/GAMT/GATM/LOC102464074
CSF1R/EGF/FGF16/FGF4/FGF6/FLI1/FLT1/GNB5/GNG7/IGF1R/KDR/LOC102446945/LOC102461925/LOC1067
AARS2/ALG12/BCAT1/EARS2/LOC102447253/OAT
NEURL1B
HK1/LOC102447192/LOC102460847/PGM2L1/PGM5
AK1/AK5

ME1/ME3
CENPE/DUSP10/DUSP26/DUSP27/DUSP6/EYA4/HSP90AA1/HSPA2/JAK2/LOC102448731/LOC102451438/LOC10
LOC102462444/RASSF2/TEAD3/TSPAN9
FOS/HSP90AA1/HSP90B1/IL17D/LOC102443642/LOC102448244/LOC102448412/LOC102454894/LOC102455
CREB3L1/FOS/JAG2/LOC102448244/LOC102462153/MAP2K6/MAP3K15/MMP16/SOCS3/SYNGAP1/VEGFD
IL2RG/LOC102448244/NFATC1/TGFB2/TLR4
CAMK2A/CAMK4/CREB3L1/FOS/LOC102453880/LOC102455777/PPP3CA/TTI2
EGF/FGF16/FGF4/FGF6/FLT1/FZD4/KDR/LOC102449267/LOC102449499/LOC102455684/LOC102461925/RAR
CA4/LOC102449893/SLC12A4
ADAM10/ADAM23/ADAM8/ADAMTS15/ADAMTS19/ADAMTS20/ADGRG1/ADGRL3/ADGRL4/CAPN2/CAPN3/CAST/CD10
CDC20/DUSP10/DUSP26/DUSP6/HTRA1/LOC112546124/MAP3K15/NR1H3
CAPN2/CAPN3/CCNB1/IGFBP3/LOC102453880/LOC102454894/LOC102455936/MAP2K6/MAPKAPK3/MCU/MCUB/
LDH-A/LOC102447912/LOC102462297
ABCC9/ABCD2/LOC102443591/LOC102452280
ASB14/ATP2A3/CACNA1B/CAMK2A/CAPN2/CAPN3/CDC20/FUS/FZD4/HSPA5/HTRA1/LOC102443591/LOC102448
AK1/AK5/AMPD1/ENTPD2/LOC102443591/LOC102446326/NT5C1A/NT5E/PDE2A/PDE4B/PGM2L1/PGM5
EFNB2/ZMIZ1
ADIPOQ/LOC102446475/LOC102448244/LOC102454753/LOC102454894/LOC102463832/LOC106732070/LPIN
ACTA1/ANXA2/FOS/HSP90AA1/HSP90B1/LOC102443591/LOC102448244/LOC102452779/LOC102454894/LOC1
BCAT1/DPYSL2/GADL1
CAMK2A/CAPN2/CAPN3/HSP90AA1/JAK2/LOC102446945/LOC102448244/LOC102454753/LOC102455777/LOC1
BCAT1
LOC102460367
FZD4/GDF1/GLI2/GLI3/WNT5A/WNT9A
ATP2A3/CACNA1B/LOC102454459/LOC102456421/LOC102458926/LOC102460301/MAP3K15/MCU/MCUB/MYF6/
CHST9/CSGALNACT1/XYLT1
EGF/FOS/JAK2/MAP2K6/NFATC1/PPP3CA/PRKCD/PRKCQ/TLR4
FOS/GNB5/GNG7/HCK/IRF7/JAK2/LOC102451500/LOC102454894/LOC102456961/LOC102458809/LOC102460
EGF/FOS/LOC102446945/LOC102452806/LOC106732455/PDGFD/PDGFRA/RALGDS/RGL1/SLC22A16
EGF/F2R/FCER1G/IGF1R/LOC102446945/LOC102447429/LOC102454894/LOC102458809/LOC106732455/LPA
ADAM10/ATP2A3/CAPN2/CAPN3/CDC20/FZD4/IGF1R/IRS1/LOC102443591/LOC102448244/LOC102453880/LO
ALDH1L2/MTHFD2
SEC31A/SRP19
LOC102448244/LOC102454459/LOC102462153/PLCB1
CACNA1B/CREB3L1/EGF/FGF16/FGF4/FGF6/FOS/HSP90AA1/HSP90B1/JAG2/JAK2/KLF5/LOC102453880/LOC1
SIM2
EARS2
COQ10B
ACSL1/BAAT/ELOVL6/HACD1/LOC102447912/LPCAT1/MBOAT1
FOS/JAK2/LOC102458809/PRLR/SLC2A4/SOCS3/TNFSF11
EGF/FGF16/FGF4/FGF6/IGF1R/PDGFD/PDGFRA
ARG2/NOS1
CAMK2A/FRZB/FZD4/LOC102447587/LOC102455777/NFATC1/PLCB1/PPP3CA/PRICKLE1/ROR1/SOX7/WNT5A/W
BIN1/CAV1/CAV2/CAV3/CBL/EHD4/FLT1/HSPA2/IGF1R/IL2RG/KDR/LOC102449842/LOC102452779/LOC1024
CXCL12/LOC102443642/LOC102448244/LOC102454894/LYN/PRKCD/PRKCQ/TLR4/TNFSF11
LOC102446326/NMRK2/NT5C1A/NT5E
AARS2/ALG12/EARS2/LOC102447253
CDC20/CREB3L1/DDX3X/FOS/IRF7/JAK2/LOC102454894/LOC102456381/LOC102458809/MAP2K6/NFATC1/TG
DAPK2/EGF/LOC102454894/LOC102458809/MMP2/THBS2
CAMK2A/CAMK4/LOC102453880/LOC102454459/LOC102455777/PLCB1/PPP3CA
CREB3L1/CXCL12/GNB5/GNG7/ITGA4/ITGA8/ITGAV/JAK2/LOC102443642/LOC102448244/LOC102454459/LO
HSPA5/SRP19
CACNA1B/GNB5/GNG7/KCNJ3/LOC102446945/LOC102453880/LOC102454459/LOC106732455/PLCB1/PPP3CA
LOC102464074

IRF7/LOC102443642/LOC102448244/LOC102459066/POLR2H
BAAT/GADL1
HSP90AA1/HSP90B1
AQP1/AQP4/ARHGDIB/CREB3L1
CDC20/CDC45/PPP2R2C/SMC2/SMC4/TTK
LOC102447223
BCHE/GPD2/LOC102446945/LOC102453489/LOC106732455/LPCAT1/LPIN1/MBOAT1/PLA2G5
BAAT/ELOVL6/HACD1
LOC102443591/LOC102448244/LOC102452280
ALG12/FUT8/MGAT3/ST6GAL1
CEL/LOC102447223/LPIN1/MBOAT1/MOGAT1
AK1/AK5/DPYSL2/LOC102463089/TPMT
DAPP1/FOS/LOC102455168/LYN/NFATC1/PPP3CA/TSPAN2
CDC20/CLEC17A/FOS/HSPA2/IL2RG/IRF7/JAK2/LOC102448244/MSN/TLR4
NEU3/TEAD3
CAMK2A/CREB3L1/FZD4/LOC102454459/LOC102455777/PLCB1/WNT5A/WNT9A
CREB3L1/HSP90B1/HSPA5/LOC102454459/LOC102462326/PLCB1
DDX3X/IRF7/LOC102454894/PIN1/TBKBP1
CACNA1B/GABRR2/GNB5/GNG7/KCNJ2/KCNJ3/LOC102453786/LOC102453880/LOC102454459/PLCB1
CREB3L1/FOS/IGFBP3/IRS1/JAK2/LOC102453880/LOC102454459/MAP2K6/PLCB1/SOCS3
CERS4/GDF1/LOC102453468/NEU3/TEAD3
LOC102446945/LOC106732455/PLA2G5
BAAT/ELOVL6/HACD1
LOC102446945/LOC102450275/LOC106732455/LPCAT1/PLA2G5
ACTA1/LOC102447587/LOC102456632/LOC102462444/PPP2R2C/RASSF2/TEAD3/TSPAN9
CCNB1/CDC20/CDKN2C/CREB3L1/FLI1/FOS/IL2RG/ITGB2/JAK2/NFATC1/NRP1/PPP3CA/SLC2A4/SPI1/TGFB2[illegible]
BAAT/BMP5/IGF1R/LOC102446945/LOC106732455/STAR
CDC20/HTRA1/SEPT5
ALG12/B4GALNT4/CHST9/FUT8
ABCC9/ABCD2/AQP1/AQP4/CALHM2/LOC102443591/LOC102445332/LOC102446585/LOC102450447/LOC10245[illegible]
CERS4/FCER1G/GDF1/LOC102454459/LOC102458809/MAP3K15/PLCB1/PPP2R2C/PRKCD/PRKCQ
BLVRA/EARS2
BHLHA15/PKM/SLC2A4
CEL/MOGAT1/PLA2G5
FCER1G/LOC102446945/LOC102458809/LOC106732455/LYN/MAP2K6
CDKN2C/EGF/FOS/IGF1R/JAG2/LOC102458809/LOC102462423/MMP2
IGF1R/IRS1/LOC102462326
BAAT/LOC102448341
NQO1
CLIP2/FNIP1/FZD4/IGF1R/IRS1/LOC102461694/LPIN1/RNF152/SESN3/WNT5A/WNT9A
EN2/GLI2/GLI3
LOC102459910/XYLT1
ADAM10/JAM3/LOC102454894/LOC102456381/LOC102458809/LYN
ADIPOQ/FOS/IGF1R/IRS1/LOC102448244/LOC102454894/MAP3K15/NR1H3/PKM/SOCS3/SREBF1
ARHGDIB/CAMK2A/CAMK4/IRS1/KIDINS220/LOC102455777/MAP3K15/MAPKAPK3/PRKCD/PRKCQ
EGF/HEY1/JAG2/LFNG
FOS/IRF7/LOC102443642/LOC102448244/LOC102454894/MAP2K6/TLR4
CACNA1B/ENTPD2/LOC102443591/LOC102452890/LOC102453880/PKD2/PLCB1
LOC102448373/LOC102454459/LOC102462326/PLCB1
ADIPOQ/CAMK4/CREB3L1/IGF1R/IRS1/SESN3
CAMK2A/CAMK4/EGF/IGF1R/LOC102455777/PDGFRA
ACTA1/CAPN2/CAPN3/CDC20/FOS/HTRA1/LOC102443591/LOC102456632/LOC102458936/LOC102459436/MAP[illegible]
CCNB1/EGF/IGF1R/IRS1/PCK1/PLK1/SETD7/SLC2A4/TGFB2/TGFBR2
CEL/LOC102448341

ALDH1A3/LOC102464074
EGF/JAK2/RALGDS/RGL1/TGFB2/TGFBR2
CAMK2A/CCNB1/CDC20/IGF1R/LOC102455777/PLK1/PPP3CA/SGO1
ALDH1A3/LOC102463832/LOC102464074
LOC102447912
CPE/CPZ/GADL1/LOC102448244/LOC102459436
CCNB1/CDC20/IGFBP3/SESN3/THBS2
CCNB1/CDC20/CDC45/CDKN2C/PLK1/TGFB2/TTK/ZBTB44
A4GALT/ST3GAL1
DHDDS
ARPP21/CELF2/EGF
GADL1/OXCT1
BICD2/LOC102460859/PIN1
MAPKAPK3/NR1H3/RARB
LOC102464093/TPM1/TPM3
CREB3L1/LOC102453880/LOC102454459/PLCB1/STAR
ACTA1/ERO1A/LOC102456381/LOC102456632
ADORA2A/CAMK2A/CDC20/HSPA5/HTRA1/LOC102443591/LOC102455777/LOC102456421/MAP3K15/MCU/MCUB/[illegible]
LOC102446945/LOC102457340/LOC106732455/PLA2G5/PTGIS
GLI2/GLI3/LOC102455075
LOC102448373/LOC102455936/LOC102462326/SLC9A2
BCAT1/LDH-A/LOC102462297
ACSL1
TXNRD1
EGF/FOS/RALGDS/RGL1/TGFB2/TGFBR2
INPP4A/PLCB1/PLCD4/PLCH2
IGF1R/IRS1/LOC102447294/LOC102463494
CXCL12/ITGA4
LOC102446945/LOC106732455/PLA2G5
ACTN2/ACTN3/CDC20/CREB3L1/DDX3X/GSN/HDAC9/IRF7/JAK2/LOC102458809/LOC102460859/LYN/MAPKAPK[illegible]
ACTA1/CDC20/IRF7/JAK2/LOC102445365/LOC102448244/LOC102450141/LOC102454894/LOC102456632/LO[illegible]
CAMK2A/CDKN2C/CREB3L1/FZD4/LOC102453880/LOC102454459/LOC102455777/PLCB1/STAR/WNT5A/WNT9A
DHDDS
AEBP2/ARNTL/ARX/BARX2/BHLHA15/CREB3L1/CSRP3/EBF3/EMX2/EN2/FEV/FLI1/FOS/GLI2/GLI3/HEY1/HIV[illegible]
ALDH1A3/LOC102462423/LOC102463832
LOC102447223
CDC20/CLDN19/EGF/IRF7/JAK2/LOC102455962/NR1H3/PPP2R2C/SOCS3/TSPAN2
A4GALT/ALG12/B3GNT7/B4GALNT4/CHST9/CSGALNACT1/FUT8/LFNG/LOC102459910/MGAT3/ST3GAL1/ST6GAL[illegible]
HPSE
CAMK2A/CBL/EGF/LOC102455777/LOC102458809
TEAD3/TSPAN9
HSP90AA1/HSPA2/RALGDS
LOC102447223
ATP8A2/CCDC86/DUSP6/FTSJ3/HSPA2/LOC102462213/LOC106731839/MAK16/MDN1/NHP2/NLE1/SOX7/UTP20
LOC102444204/LOC102451133/LOC102455777/MME
CACNA1B/GABRR2
FOS/LOC102458809/NFATC1/PPP3CA/PRKCD/PRKCQ
ACTA1/FZD4/IGF1R/LOC102456632/NQO1/TGFB2/TGFBR2/TXNRD1/WNT5A/WNT9A
CCNB1/CFL2/FOS/GNB5/GNG7/LOC102454459/LOC102460859/MAP2K6/NFATC1/PPP3CA/TLR4
ALDH1A3/LOC102462423/LOC102463832
LOC102464074
ADORA2A/CALCRL/CHRNA1/CHRNB1/CHRND/CHRNE/CNR2/EDNRA/F2R/GABRR2/HTR2A/LOC102444635/LOC1024[illegible]
ACSL1/LOC102463832
LOC102448341/MYLIP/STAR

LFNG/ST6GAL1
CACNA1B/CPLX4/SLC6A4/TTI2
CLIP4
LOC102444204/LOC102451133
MLLT11/SETD7/SMYD1
ABCD2/ACSL1/BAAT/NOS1
LOC102450275/MAP2K6
PCK1
ADORA2A/CAMK4/CREB3L1/GNB5/GNG7/HDAC9/PKIA/TTI2
LOC102448341/LOC102462423/PTGIS
LOC102447912
ACSL1/LOC102454753
BCAT1/OXCT1
CCNB1/HSP90AA1/IGF1R/PLK1
EGF/FOS/LOC102447223/LOC102454753/LOC102458809/MAP3K15/NCF1/NCF2/NQO1/PRKCD/PRKCQ
CBL/RUNX1/TGFB2/TGFBR2
LOC102458809/RAB7B/USP43
FCER1G/ITGB2/LOC102458809/LOC102459436/NFATC1/PPP3CA/TYROBP
LOC102457574/LOC102464074
LOC102447223
ST3GAL1
LOC102453747/POLR1E/POLR2H
IL2RG
IGF1R/IRS1/MAP2K6
ARL15/GNB5/GNG7/LOC102454459/RAB15/RAB44/RAB7B/RHOJ
FUS
LOC102448244/LOC102459436
F13A1/FOS/JAK2/LOC102446475/LOC102448244/LOC102454753/LOC102454894/LOC102462153/LOC106731
AARS2/ALG12/EARS2/EEF1A2/FAM98A/LOC102447253/WDR4
LOC102448341/LOC102463832
GADL1
DAPK2/IGF1R/IRS1/PRKCD/PRKCQ/RAB7B
CCNB1/CDC45/NHP2/SOX7
RALGDS
EGF/LOC102455684
ENO3/PFKM
CREB3L1/TTI2
LAMP3/LAPTM4B/LAPTM5/LOC102453468/LOC102456381/LOC102458936/TEAD3
CDC20/CDC45
ACTA1/ASB14/CDC20/FUS/HSPA5/LOC102443591/LOC102456632/LOC102462213/MAP2K6/MAP3K15/MCU/MCU
A4GALT
CDC20/ENTPD2/IRF7/JAK2/LOC102456961/LYN/MAP2K6/RUNX1/VIM
SLC2A4/TGFB2
INPP4A/PLCB1/PLCD4
MDN1/NHP2
ACTA1/DNAJA4/HSP90AA1/HSPA2/ISCA2/LOC102451040/LOC102456421/LOC102456632/MCU/MCUB/MSS51/N
CDC20/MAP3K15
ACSL1/ACTA1/CREB3L1/LOC102456632/LOC102463494/MAP2K6/MAP3K15/NDUFAF2
CENPE/EEF1A2
EGF/RARB
FUS/PPP2R2C
LOC102453468/LOC102462423
ACTA1/BICD2/CELF2/CENPE/DDX3X/EEF1A2/ENO3/FUS/GDF1/LOC102443591/LOC102451058/LOC102453747
LOC102459436

CIRBP/FUS/HSPA2
CBL/CDC20/KLHL31/SOCS3
ACTN2/ACTN3/LOC102446475/LOC102453747
LOC102452779
ASB14/ASB16/ASB18/ASB2/ASB4/ASB5/BTBD6/CBL/CCNF/CDC20/FBXL22/FBXO40/IVNS1ABP/KBTBD11/KBTB[illegible]
CELF2/CIRBP/CRYAB/DDX3X/EEF1A2/FUS/HSPA2/HSPB1/HSPB2/HSPB6/MANF/MBNL2/PSPC1
LOC102459436
MRPL20/RPL3L
EEF1A2
ASB14/CACNA1B/CDC20/CREB3L1/LOC102443591/LOC102453747/LOC102454459/MAP3K15/PLCB1/POLR2H/T[illegible]
ACTA1/CAVIN1/FUS/LOC102453747/LOC102456632/PIN1/POLR1E/POLR2H/PSPC1
LOC102456381
ASB14/CDH23/CLDN19/DOCK2/DOCK4/LOC102446703/LRRC6/PKD2/SEPT5/SPACA4/TMC5
ACTA1/AEBP2/ARHGEF10L/CCNB1/CCNF/CDC20/CDH13/CENPE/CEP85L/CKAP2/CLIP2/DDX3X/HDAC9/JARID2/[illegible]
MRPL20/RPL3L
LCP1/LOC102450275/LOC102453747/NAV3/PER1/PER2/PLS1/POLR2H/RALGDS
CD74/CDC20/IRF7/ITGA4/ITGA8/ITGAV/JAK2/LOC102448244/LOC102457850/LOC102458809/LOC10246279[illegible]

Gene name
IGF1/LOC102446622/LOC102451731/LOC102453880/LOC102454405/MYL2/MYL3/MYL4/SGCD/TPM1
IGF1/LOC102446622/LOC102451731/LOC102453880/LOC102454405/MYL2/MYL3/MYL4/SGCD/TPM1
FDFT1/LOC102453483/SQLE
LOC102446622/LOC102453880/LOC102454405/MYL2/MYL3/MYL4/SLC9A2/TPM1
MEF2C/MYL2/MYL3/MYL4/MYLK2/MYLK4/NOS1/PRKCQ/SLC9A2
CUNH10orf71/LOC102446622/LOC102451731/LOC102454405/LOC102455319/LOC102455412/LOC112546115[illegible]
LOC102446622/LOC102453880/LOC102454405/LOC102455412/LOC112546115/MYLK2/MYLK4/PRKCQ
LOC102446622/LOC102453880/LOC102454405/MYL2/MYL3/MYL4/SLC9A2/TPM1
LOC102445123/LOC102449400/PFKM
HSPH1/LOC102446622/LOC102453880/LOC102454405/LOC102455412/LOC112546115/MYL2/PRKCQ
LOC102445123/LOC102449400/PFKM
LOC102446622/LOC102453880/LOC102454405/MEF2C/MYLK2/MYLK4/PRKCQ
FDFT1/LOC102453483/SQLE
LOC102445123/LOC102449400/PFKM
LOC102446622/LOC102454405/LOC102459228/PFKM/PLCD4/SLC9A2
NPAS2/PER1
KCNJ2/LOC102453880/LOC102455412/MEF2C/MYLK2/MYLK4
FDFT1/LOC102453483
LOC102446622/LOC102454405/LOC112546115/MYL2/MYLK2/MYLK4/SLC9A2
CHRNB1/CHRNE/KCNJ2/KCNS3/LOC102453880/LOC102455936/LOC102461212
DNAJA4/HSPB1/HSPH1/ITGB1BP2/LOC112545587/SERPINH1
LOC102445123/LOC102449400/PFKM
LOC102445123/LOC102449400
BCAT1
PYGM
KCNJ2/LOC102453880/NR4A1/PRKCQ
KCNJ2/LOC102453880/NOS1/PER1
ADIPOQ/LOC102453880/PRKCQ
KCNJ2/MYLK2/MYLK4/SLC9A2
CAPN3/DNAJA4/HSPB1/HSPH1/LOC102462213

AQP4/LOC102446585/LOC102459228/LOC102461259/MB/SLC16A3/SLC25A12/SLC2A12/SLC43A2/SLC9A2
IGF1/LOC102449400/NOS1/PFKM
ADIPOQ/IGF1/LOC102445123/PFKM
NPAS2/PER1
EYA4/IGF1/LOC102450564/LOC112545587/MEF2C/PER1
BCAT1
CCNB1/IGF1/PLK1
LOC102446622/LOC102454405/SGCD
ADIPOQ/FHL1/FLRT3/LDB3
GATM/NOS1
AK1/HSPB1/LOC102445123/LOC102446622/LOC102449400/LOC102451731/LOC102454405/LOC102455412/L
LOC102453880/MYLK2/MYLK4/NOS1/PLCD4
CAPN3/CCNB1/LOC102453880/LOC102455936
CAPN3/IGF1/MYL2/MYLK2/MYLK4
LOC102445123/PFKM/PYGM
CCNB1/IGF1/PLK1
AQP4/LOC102459228/SLC9A2
LIPG/LPIN1
AK1/TPMT
HSPB1/IGF1
AK1
KCNJ2/LOC102453880
LOC102455936/SLC9A2
HSPB1/IGF1/LOC102453880/MEF2C/NR4A1
CCNB1/IGF1/PLK1
ALPK3/LOC102451731/LOC102455936/MYLK2/MYLK4/PLK1/PRKCQ
NOS1
CCNB1/IGF1
LOC102453787
ADIPOQ/PRKCQ
LOC102453880/NR4A1
PFKM/SLC16A3
KCNJ2/LOC102453880/NPAS2
KCNJ2/LOC102453880
LOC102447557/LOC102452168/LOC102456827
KCNJ2/LOC102453880
ADIPOQ/IGF1
IGF1/NOS1
CAPN3/LOC102455936/PYGM
LOC102446622/LOC102454405/LOC112546115/WIPF3
HSPB1/LOC102450564/NOS1
LOC102453880/SGCD
BCAT1
SLC9A2
BATF/PRKCQ
A4GALT
HSPB1/IGF1/LOC102447587/SLC9A2
GATM
HACD1
HACD1
NOS1/SLC9A2
PFKM
CAPN3/MYL2/PLCD4/PRKCQ
EYA4/LOC102451909/LPIN1/PFKM/PTP4A3/SLC9A2

PYGM
LOC102453880/PRKCQ
CCNB1/PLK1
PRKCQ/PYGM
IGF1
JPH2
BIN1/PRKCQ
HSPH1
LOC102453880/PRKCQ
KCNJ2/LOC102453880
PLCD4/PRKCQ
KCNJ2/LOC102453880
A4GALT
IGF1/LOC102453880
AQP4
FHL1/PRLR
IGF1/PRKCQ
NR4A1
TPM1
KCNJ2/PRKCQ
ADIPOQ/LPIN1
CHRNB1/CHRNE/LOC102444635/LOC102450564/PRLR
APOBEC2/CCNB1/LOC102445544
BCAT1
BCAT1
LOC102445123/PYGM
KCNJ2/LOC102453880
LOC102453880
LOC102450564/LOC112545587
BCAT1
IGF1/LPIN1
CDH13/MPZ
LIPG
AK1
LOC102450564
PFKM
PLCD4
LOC102450564
LOC102455936/PRKCQ
LOC102453880/NR4A1
LOC102447587
LOC112545587
CAPN10/CAPN3/LOC102450564/LOC112545587/SERPINH1/USP13
LOC102450564
LOC102450564
MYLK2/MYLK4
ADIPOQ
PRKCQ
IGF1
LOC102450564
HACD1
HSPB1
PRLR
IGF1

PER1
NOS1
LOC102453880
LOC102453880
NOS1
IGF1
IGF1
CAPN3/LOC102450564
KCNJ2
LOC102462213
BIN1/DNAJA4/LOC102449400/LOC102451731/LOC102452796/LOC102455319/LOC102462213/MCF2/MYOT/PL[illegible]
LOC102447587
PLCD4
PRLR
PRKCQ/TPM1
LOC102450564/LOC112545587
LOC102450564/PRKCQ
PRKCQ
LOC102453880
LOC102453880
CAPN3/LOC102453880/LOC102462213/NOS1
ASB16/ASB2/KLHL41/LIMCH1/PDZRN3/TRIM63/USP13
LOC102447587
NOS1
NOS1
CCNB1
PRKCQ
NOS1
IGF1/NR4A1/PRLR
CAPN3/LOC102453880/NOS1
PRKCQ
IGF1
NOS1
LOC102453880/PRKCQ
LPIN1
IGF1
LOC102450564/LOC112545587
LIMCH1
LOC102453880
LOC102453880/SLC9A2
HSPH1/VLDLR
MEF2C
PRKCQ
BIN1/WIPF3
SLC9A2
PRKCQ
MEF2C
AK1
SLC9A2
LOC112545587
IGF1
MYL2
PRKCQ
PRKCQ

NOS1
LOC102450564
IGF1
PKIA
VLDLR
NOS1
ADIPOQ
IGF1
LOC102447587
MPZ
LOC102450564
MEF2C
LOC102462213/MBP/PFKM
DNAJA4/MSS51
PRKCQ
WIPF3
CCNB1
PRLR
NOS1
LOC102453880
LOC102462213/NOS1
PRKCQ
CCNB1
LOC102462213
CCNB1/CDH13/LOC102449400/NPAS2/PLK1/TPX2
A4GALT
IGF1/NOS1/TPM1
IGF1
IGF1
LOC112545587
MYL2
BATF/CIART/MEF2C/NPAS2/NR4A1
PER1
HSPB1
LOC102444635

C102445983/LOC102446383/LOC102446622/LOC102450688/LOC102451731/LOC102453880/LOC102454405/L
C102445983/LOC102446383/LOC102446622/LOC102450688/LOC102451731/LOC102453880/LOC102454405/L
50688/LOC102453880/LOC102454405/LOC102455980/LOC102456421/LOC102456632/LOC102458660/LOC102
4A5/COL5A1/COL5A2/COL6A1/COL6A3/DOCK2/DOCK4/EGF/FLT1/IGF1R/ITGA4/ITGA7/ITGA8/ITGAV/ITGB2/K
2446622/LOC102450688/LOC102452890/LOC102453880/LOC102454405/LOC102454459/LOC102455777/LOC1
FSCN1/GSN/HCLS1/KIF1C/KIF24/LCP1/LMOD2/LOC102443591/LOC102445983/LOC102446383/LOC102446622
F1R/ITGA4/ITGA7/ITGA8/ITGAV/ITGB2/KDR/LOC102444471/LOC102445983/LOC102446383/LOC102446622/
2/FBN1/FBN2/FGF16/FGF4/FGF6/FLT1/GDF1/HPSE/KDR/LOC102444471/LOC102447224/LOC102447978/LOC1
TGA8/ITGAV/ITGB2/LAMA2/LAMA5/LAMB1/LAMC1/LOC102447224/LOC102447615/LOC102447978/LOC102456
B2/HSPB6/ITGB2/LAMA2/LAMA5/LAMB1/LAMC1/LOC102444471/LOC102447978/LOC102448244/LOC10244965
/CENPE/CFL2/CKMT2/CLDN19/COL4A1/COL4A2/COL4A5/COL6A1/COL6A3/CRYAB/CSTA/DPYSL2/ECE1/EEF1A2/
2/LOC102453880/LOC102455684/LOC102456421/LOC102456632/LOC102458926/LOC102460301/RYR3/SGCA/
945/LOC102450688/LOC102453880/LOC102454405/LOC102454459/LOC102455412/LOC102455980/LOC10245
/CSF3R/EGF/F2R/FGF16/FGF4/FGF6/FLT1/GNB5/GNG7/HSP90AA1/HSP90B1/IGF1R/IL2RG/IRS1/ITGA4/ITGA
400/LOC102462297/LOC102463832/PCK1/PFKM/PGM2L1/PGM5/PKM
GF6/FLT1/FOS/FZD4/GDF1/GLI2/GLI3/GNB5/GNG7/HEY1/HSP90AA1/HSP90B1/IGF1R/IL2RG/IL5RA/ITGA4/I
02450688/LOC102453880/LOC102454405/LOC102454459/LOC102455980/LOC102458660/LOC102458926/LOC
6383/LOC102446622/LOC102450688/LOC102452779/LOC102453393/LOC102453880/LOC102454405/LOC1024
/HPSE/HSPB1/HSPB2/HSPB6/IGF1R/ITGA4/ITGA8/ITGAV/ITGB2/KDR/LOC102447587/LOC102447978/LOC102
8244/LOC102451133/LOC102451319/LOC102451362/LOC102453941/LOC102456961/LOC102457584/LOC1024
OC102454753/LOC102454894/LOC102456223/LOC102459430/LOC102461635/LOC102462153/MMP2/NFATC1/F
C102454753/LOC102456632/LOC102458809/LOC102461635/LOC102461925/LOC102462153/MAP2K6/MAP3K15
LOC102458809/LOC102460983/LOC106732359/LOC106732423/MYL10/MYL9/NRP1/NTNG1/NTNG2/PLXNA1/PLX
/IGF1R/IGSF3/IL2RG/IL5RA/ITGA4/ITGA7/ITGA8/ITGAV/ITGB2/JAG2/KDR/LAMP3/LIFR/LOC102443928/LO
405/LOC102455980/LOC102456632/LOC102458660/LOC102458809/LOC102459436/LOC102459610/MYH7B/SO
61635/LUM/OGN/PRELP/SPOCK3/TGFBR3/VCAN
3880/LOC102454459/LOC102455777/LOC102456421/LOC102458926/LOC102460301/LOC102461925/MCU/MCU
C102454405/LOC102455980/LOC102456632/LOC102458660/LOC102458809/LOC102458926/LOC102459228/L
OL6A3/LOC102445365/LOC102447224/LOC102447978/LOC102450141/LOC102455777/LOC102456223/LOC102
6/LOC102453880/LOC102454459/LOC102455412/LOC102455777/LOC102456421/LOC102456632/LOC102458
02455684/LOC102456632/LOC102457786/LOC102457840/LOC102462624/MMP2/MSN/MYL10/MYL2/MYL9/NCF1
A/MEF2C/MYL10/MYL2/MYL3/MYL4/MYLK2/MYLK4/NOS1/PLAT/PLCB1/PRKCD/PRKCQ/RYR3/SLC9A2
02456197/LOC102456381/LOC102456632/LOC102457786/LOC102458936/LOC102462624/LOC102463089/MRC
A4/SOCS3
/FLT1/FOS/HSPA2/HSPB1/HSPB2/HSPB6/IGF1R/KDR/LOC102446945/LOC102448244/LOC102453880/LOC1024
3941/LOC102457786/LOC102462153/LOC102462624/MPZ/NRXN2/NTNG1/NTNG2/PECAM1/PTPRM/PTPRS/SELPL
02447978/LOC102448412/LOC102455075/LOC102456223/LOC102456632/LOC102458809/MMP2/NOS1/PLCB1/
PB3/HSPB6/HSPB7/HSPB8/HSPH1/HTRA1/HTRA3/HTRA4/HYOU1/ITGB1BP2/LOC102449766/LOC102455777/LOC
471/LOC102454339/LOC102454894/LOC102457786/LOC102461925/LOC102462624/LOC102463089/MEF2A/ME
786/LOC102462624/NCF1/NCF2/NOS1/TGFB2/TLR4
02447978/LOC102454459/LOC102456223/LOC102456632/LOC102458809/LOC102463494/LOC106732455/LYN
CNJ2/KCNJ3/KCNMA1/KCNMB1/KCNQ4/KCNS3/KCNT1/LOC102444244/LOC102448373/LOC102452890/LOC10245
F1/NCF2/NFATC1/PPP3CA/SOCS3/SPI1/TGFB2/TGFBR2/TNFSF11/TYROBP
449267/LOC102449499/LOC102451319/LOC102455777/LOC102456197/LOC102456381/LOC102457786/LOC10

C102455777/LOC102456223/LOC102456421/LOC102458926/LOC102460301/MMP2/NCF1/NCF2/PDK4/PLCB1/P
2453941/LOC102457786/LOC102462153/LOC102462444/LOC102462624/LOC102463734/LOC106731301/LOC1
22/LOC102447429/LOC102448244/LOC102448731/LOC102450688/LOC102452779/LOC102454405/LOC102454
777/LOC102461925/LOC102462297/NOS1/PDK4/PFKM/SLC2A4/TLR4
KM/SLC16A3/SLC2A4

02456421/LOC102463494/NOS1/PER1/PER2/PLCB1/RYR3

9430/NOS1/RARB/ZBTB44
2453786/LOC102453880/LOC102454459/LOC102455777/LOC102458809/PLCB1
02459430/LRFN4/MATN2/NID2/POSTN/RAB44/RYR3/TGFBI
684/LOC102456632/LOC102458809/LOC102459430/SEPT5
/LOC102447429/LOC102454459/LOC102456632/LOC102457786/LOC102458809/LOC102461925/LOC10246262
777/LOC102456421/LOC102458926/LOC102460301/LOC102462326/LOC102464019/PLA2G5/PLCB1/RYR3/SLC

2462624/LOC102462847/LOC106732070/PTAFR/SELPLG

5777/LOC102456421/LOC102462326/PLCB1/RYR3/SLC2A4
ZD4/HEY1/ITGA4/ITGA7/ITGA8/ITGAV/ITGB2/JAG2/JAK2/LAMA2/LAMA5/LAMB1/LAMC1/LFNG/LOC10244722
PAN2
02457786/LOC102459066/LOC102462624/NOS1/TLR4
51408/LOC102451500/LOC102452168/LOC102453941/LOC102456197/LOC102456961/LOC102460285/LOC102
02454894/LOC102457584/LOC102458090/LOC102460859/PRLR/RELT/TGFB2/TGFBR2/TNFRSF19/TNFSF11/TN
TNFSF11
1362/LOC102455777/LOC102457786/LOC102461035/LOC102461273/LOC102461635/LOC102462624/LOC1067
/LOC102459066/LOC102462213/LOC102462624/TLR4

102456197/LOC102456961/LOC102460285/MRC2/TLR4
APK3/PPP3CA
OC102462326/MSN/MYLK2/MYLK4/PLCB1/SLC9A2
894/PDGFD/TGFB2/TNFSF11/TNFSF8/VEGFD
ARHGEF10L/BAIAP2/BCAS4/BICD2/BIN1/CAP2/CAV1/CAV2/CAV3/CBL/CCDC141/CEL/CFL2/CHN1/CLEC17A/CC
3880/LOC102454459/LOC102455075/LOC102455777/PLCB1/PPP2R2C/PPP3CA/TTI2
OC102454753/LOC102454894/LOC102455777/LOC102458809/LOC102459066/LOC102462153/LYN/MAP2K6/MA
02448244/LOC102453880/LOC102454753/LOC102456421/LOC102458809/MCU/MCUB/NCF1/NCF2/PPP3CA/PRK
7192/LOC102448244/LOC102450275/LOC102452779/LOC102454894/LOC102456632/LOC102457840/LOC1024

6/LOC102462847/LOC102463153/LOC102463494/NOS1/PLCB1/RYR3/SLC9A2
26/PDE2A/PLCB1/PRKCD/PRKCQ/STAR
/LOC102447848/LOC102451731/LOC102455777/LOC102455936/LOC102458809/LOC102460847/LOC10246192
PB6/HSPH1/HYOU1/LOC102462213/MAP3K15/RALGDS/SEC31A
CB1/PPP3CA/PYGM/SLC2A4

BC1D4

59/LOC102454894/LOC102456632/LOC102458809/LOC102459066/LOC102459430/MAP2K6/NFATC1/TLR4/WIP

RA/PLAT

2455075/LOC102458809/LOC102460859/LYN/NCF1/PLCB1/PREX1/PRKCD/PRKCQ

D/PRKCQ

OCS3/SREBF1/TRIP10

/MYOD1/WNT5A/WNT9A
OC102454459/LOC102462423/LOC106732455/PLCB1/SLC6A4/TTI2
2/PLCB1/PRKCD/PRKCQ/RARB
PDCD4/PDGFRA/PRKCD/PRKCQ/TGFB2/THBS2/TIMP3/TPM1/TPM3/VIM
PRKCQ/SLIT3
456632/LOC102457786/LOC102458809/LOC102462153/LOC102462321/LOC102462624/LOC102462847/LOC1(
EF2C/MMP16/PDE4B/PLCB1/TNFSF11
BA8/TUBB6

K6/MMP16/MMP2/PLCB1/PRKCD/PRKCQ
2459066/LOC102462321/LOC102462847/MCU/MCUB/PLCB1/PRKCD/PRKCQ/TLR4/ZYX

PTPRS/TGFBR2
LCB1

CS3

102453880/LOC102455777/LOC102456421/LOC102458926/LOC102460301/LOC102462326/MYL9/NFATC1/PDI
/LOC102447528/LOC102456975/LOC102458579/LOC102461925/LOC106731529/LOXL2/MSRB3/PHPT1/PXDN/I

KAPK3/NFATC1/PPP3CA/PRKCD/PRKCQ

2462326/SLC2A4/SLC9A2

LR4
NX1
9/LOC106732455/MAP2K6/PLCB1/PRKCD/PRKCQ

FBR2/TSPAN9/WNT5A/WNT9A

32455/PDGFD/PDGFRA/PLA2G5/RALGDS/RGL1/SYNGAP1/VEGFD

2452319/LOC102456181/LOC102456421/LOC102462153/LOC102463734/LOC106731301/LOC106731824/LOC1

67

B/TGFB2/TGFBR2/WNT5A/WNT9A

9/CPE/CPZ/CSTA/ECE1/HTRA1/HTRA3/HTRA4/LOC102443928/LOC102444204/LOC102445365/LOC102445828/

NFATC1/PPP3CA/TGFB2/TGFBR2

244/LOC102453880/LOC102454459/LOC102454753/LOC102455777/LOC102456421/LOC102458926/LOC1024[illegible]

1/MAP2K6/MAP3K15/SREBF1/TLR4
02456632/LOC102457840/LOC102459066/MAP2K6/MYL10/MYL2/MYL9/PODXL/RAB7B/RHOJ/TLR4/TUBA8/TUB[illegible]

02455936/LOC102459066/LOC106732455/PYGM/TLR4

MYOD1/PLCB1/VLDLR

859/LYN/MAP2K6/MAPKAPK3/NFATC1/PPP3CA/PREX1

R1/PDGFD/PDGFRA/PLCB1/RALGDS/RGL1
C102454459/LOC102454753/LOC102456421/LOC102458926/LOC102460301/MAP3K15/MCU/MCUB/MME/NOS1/[illegible]

02455075/LOC102458809/TNFSF11

NT9A
55075/LOC102458809/LOC102460859/LOC102461925/PDGFRA/RAB11FIP5/TGFBR2/WIPF3

FB2/TGFBR2/TLR4

C102454894/LOC102458809/LOC102460859/MAP2K6/NFATC1/PDGFRA/PLCB1/PPP3CA

/TGFBR2

2280/LOC102452806/LOC102455168/LOC102455367/LOC102459166/LOC102459228/LOC102461694/LOC1067

3K15/SEPT5/SYNGAP1/TUBA8

RYR3/SEPT5/TTI2/TUBA8/TUBB6/UCHL1

3/PKM
C102459066/SOCS3/TLR4

EP1/HOXA7/HOXA9/HOXC5/HOXD1/ID2/IRF7/KLF5/KLF7/LOC102444628/LOC102454339/LOC102457850/LOC1

1/ST8SIA5/XYLT1

45365/LOC102445828/LOC102447429/LOC102450075/LOC102450141/LOC102453747/LOC102458647/LOC10

529/LOC106732070/NRP1/RPL3L/TLR4

B/NOS1/PPP3CA/PRPH/TUBA8/TUBB6/VIM

DUFAF2/RYR3/SMTNL2/TIMM9/ZNF106

/LOC102456632/LOC102462213/MANF/MBP/PFKM/PPP2R2C/TACC3/TUBA8/VIM

D12/KBTBD13/KLHL14/KLHL24/KLHL30/KLHL31/KLHL40/KLHL41/LIMCH1/LOC102443392/LOC102453747/LO[illegible]

UBA8/TUBB6

KDM6B/KIF24/LOC102443591/LOC102448731/LOC102449400/LOC102451058/LOC102456632/LOC102463734/

1/LOC112547508/SOCS3

/MYBPC1/MYL2/MYL3/MYL4/MYOT/MYPN/TMOD1/TPM1

OC102461675/LOC102462213/LOC112545587/LOC112546115/MBP/PLP1/TPM1/USP13

CD4/SERPINH1/VLDLR/WIPF3

.OC102455980/LOC102456421/LOC102456632/LOC102458660/LOC102458926/LOC102459610/LOC102460301
.OC102455980/LOC102456421/LOC102456632/LOC102458660/LOC102458926/LOC102459610/LOC102460301
2458926/LOC102459610/LOC102460301/LOC102462326/LOC102464093/MYH7B/MYL10/MYL2/MYL3/MYL4/RYR
KDR/LAMA2/LAMA5/LAMB1/LAMC1/LOC102447224/LOC102447978/LOC102456223/LOC102456632/LOC1024578
102455980/LOC102456421/LOC102456632/LOC102458660/LOC102458926/LOC102459610/LOC102460301/LO
2/LOC102448731/LOC102449842/LOC102450688/LOC102451731/LOC102452779/LOC102454405/LOC1024553
/LOC102447429/LOC102450688/LOC102452779/LOC102454405/LOC102455980/LOC102456632/LOC10245778
102448244/LOC102449267/LOC102449499/LOC102454894/LOC102456223/LOC102457786/LOC102459430/LO
223/LOC102459430/LOC102462782/SEPT5/THBS2
5/LOC102450310/LOC102454459/LOC102454894/LOC102456223/LOC102457786/LOC102459430/LOC1024626
/EFEMP1/EFEMP2/EFNB2/EHD4/ENO3/FABP3/FBN1/FBN2/FCER1G/GNB5/GNG7/GPRC5B/GSN/HSP90AA1/HSPA2/
/SGCD/SGCG
56632/LOC102458660/LOC102459610/LOC102463494/LOC106732455/LOC112546115/MYH7B/MYL1/MYL9/MYL
A7/ITGA8/ITGAV/ITGB2/JAK2/KDR/LAMA2/LAMA5/LAMB1/LAMC1/LOC102447224/LOC102447429/LOC1024479

ITGA7/ITGA8/ITGAV/ITGB2/JAG2/JAK2/KDR/LAMA2/LAMA5/LAMB1/LAMC1/LOC102447429/LOC102454459/LO
C102459610/LOC102460301/LOC102462326/LOC102463494/MEF2A/MEF2C/MYH7B/MYL9/MYLK2/MYLK4/NFATC
455412/LOC102455980/LOC102456632/LOC102458660/LOC102458809/LOC102459610/LOC112546115/MAP3K
2455777/LOC102456223/LOC102456632/LOC102458809/LOC102459430/LOC102461925/LUM/MMP2/MSN/PDCD
457786/LOC102458090/LOC102462624/MME/SEPT5
PLCB1/PLCD4/PRKCD/PRKCQ/TGFB2/TGFBR2/VEGFD
5/MEF2A/MEF2C/MMP2/NCF1/NCF2/NQO1/PECAM1/PLAT
KNA2/PPP3CA/RGS3/SEMA3A/SEMA3C/SEMA5A/SEMA5B/SEMA6C/SEMA7A/SLIT3/SRGAP3/WNT5A
OC102444204/LOC102444471/LOC102445784/LOC102447615/LOC102449267/LOC102449499/LOC102451133/
GCA/SGCD/SGCG

JB/MYLK2/MYLK4/NOS1/P2RX5/PDGFD/PDGFRA/PLCB1/PLCD4/PLN/PPP3CA/PTAFR/RYR3/TNNC1/VEGFD
.OC102459610/LOC102460301/LOC102462326/MYH7B/PFKM/PLCB1/PLCD4/PLN/SLC2A4/SLC9A2/TBC1D1/TBC
2461694/LOC102462326/LOC102462782/LOC102464019/MME/SLC9A2
309/LOC106732455/MEF2A/MEF2C/MYL1/MYL9/MYLK2/MYLK4/NFATC1/PLCB1/PPP3CA/RYR3
l/NCF2/PECAM1

C2/NCF1/NCF2/NOS1/RAB7B/THBS2/TLR4/TUBA8/TUBB6

455075/LOC102461925/LOC106732455/LOC112546124/MAP2K6/MAP3K15/MAPKAPK3/MEF2A/MEF2C/NFATC1/P
.G/VCAN
/TGFBR2/VEGFD
C102457168/LOC102458936/PIN1/SERPINH1/UNC45B
EF2C/MLF1/PER1/PER2/PLAT/RARB/RUNX1/SIX1/SIX2/SPI1/TGFBR2/TSPAN7/ZBTB44

N/MYL10/MYL9/MYLK2/MYLK4/P2RX5/P2RY12/PLCB1/SEPT5
53786/LOC102453880/LOC102455936/LOC102456421/LOC102461212/LOC102462718/P2RX5/PKD2/RYR3/SCN

02458809/LOC102458936/LOC102462321/LOC102462624/LOC102462847/MRC2/NOS1/PPP3CA/TGFB2/TLR4

PLN/PRKCD/PRKCQ/RYR3/SLC2A4/TBC1D1/TBC1D4/TGFB2/TGFBR2
106731824/LOC106731825/MPZ
4894/LOC102455980/LOC102456632/LOC102458660/LOC102458809/LOC102459066/LOC102459610/LOC1125

24/LPAR1/MAP2K6/PDGFD/PDGFRA/PLCB1/RALGDS/RGL1/THBS2/VEGFD
C9A2

4/LOC102447978/LOC102456223/LOC102456381/LOC102459430/LOC102462782/PKM/PPP2R2C/THBS2/WNT5A

2461635/LOC102462153/MRC2/PECAM1/VCAN
NFSF8

731529/LOC106732070/PLAT

OLEC12/CPLX4/DNAJA4/DNAJC10/DOCK2/DOCK4/EEF1A2/EGF/EHD4/FOS/HCK/HCLS1/HK1/HSP90AA1/HSPA2/H

AP3K15/NCF1/NCF2/NFATC1/PLCB1/PPP3CA/TLR4/VLDLR
KCD/PRKCQ/RYR3/TUBA8/TUBB6
458809/LOC102459066/MYL10/MYL2/MYL9/PLCB1/PLCD4/PRKCD/PRKCQ/SEPT5/TLR4

25/LOC102463494/LOC106732354/LYN/MAP2K6/MAP3K15/MAPKAPK3/MERTK/MYLK2/MYLK4/OBSCN/PDGFRA/PD

PF3

)2463089/LOC106732070/NCF1/NCF2/PLCB1/SELPLG/TLR4

E4B/PLN/RYR3/SLC9A2
ROR1

106731825/LOC112543494/LOC112544823/LOC112546124/LPIN1/PFKM/PPM1E/PPM1F/PPP1R26/PPP1R3A/PP

/LOC102448412/LOC102449267/LOC102449499/LOC102449655/LOC102450141/LOC102450310/LOC10245113

50301/LOC102462213/MAP2K6/MAP3K15/MCU/MCUB/NOS1/PLCB1/PPP3CA/PRPH/RYR3/SEPT5/TUBA8/TUBB6/U

36

PLCB1/PPP3CA/RYR3/TUBA8/TUBB6/WNT5A/WNT9A

732518/MB/MCU/MCUB/PENK/PLN/SLC12A4/SLC16A3/SLC16A7/SLC22A16/SLC25A12/SLC2A4/SLC33A1/SLC43

102462791/LOC112547508/MAFB/MAPKAPK3/MEF2A/MEF2C/MYF5/MYF6/MYOD1/NFATC1/NFIX/NOL3/NR1H3/OS

6732070/LPAR1/P2RX5/P2RY12/PENK/PRLR/PTAFR/TAAR1

C102459781/MYLIP/NEURL1B/PDZRN3/RNF152/RNF24/SOCS3/TMEM129/TRIM36/TRIM55/TRIM63/TSPAN5/UCH

/LOC106731301/LOC106731442/LOC106731824/LOC106731825/MLLT11/NHP2/NUF2/PDPN/PLA2G5/PLK1/PPP

/LOC102464093/MYBPC3/MYH7B/MYL10/MYL2/MYL3/MYL4/RYR3/SGCA/SGCD/SGCG/TGFB2/TNNC1/TNNT2/TNN
/LOC102464093/MYBPC3/MYH7B/MYL10/MYL2/MYL3/MYL4/PLN/RYR3/SGCA/SGCD/SGCG/TGFB2/TNNC1/TNNT2/
3/SLC9A2/SLC9A9/TNNC1/TNNT2/TNNT3/TPM1/TPM3
40/LOC102458809/LOC102459430/LOC102461925/LOC102462782/MYL10/MYL2/MYL9/MYLK2/MYLK4/PARVB/
C102462326/LOC102464093/MYH7B/MYL10/MYL2/MYL3/MYL4/PLCB1/PLN/PPP2R2C/RYR3/SCN1B/SLC9A2/TN
19/LOC102455412/LOC102455684/LOC102455980/LOC102456632/LOC102457840/LOC102458660/LOC10245
6/LOC102457840/LOC102458660/LOC102458809/LOC102459430/LOC102459610/LOC102461925/LOC10246
C102461925/LOC102462153/LOC102462624/LOC102462782/MATN2/PECAM1/PLAT/SLIT3/TGFB2/THBS2/TLR

24/NOS1/PLCB1/RAB7B/TGFB2/TLR4
HSPA5/HSPB1/HSPB2/HSPB6/IGFBP3/IGSF3/ITGA4/ITGA7/ITGA8/ITGAV/ITGB2/LCP1/LDH-A/LGALS1/LOC1

K2/MYLK4/PLA2G5/PLCB1/PRKCD/PRKCQ
78/LOC102456223/LOC102459430/LOC102461925/LOC102462782/LPAR1/PCK1/PDGFD/PDGFRA/PPP2R2C/PR

C102454894/LOC102455684/LOC102455777/LOC102457584/LOC102458090/LOC102459430/LOC102461925/
1/PDE2A/PLCB1/PLN/PPP3CA/PRKCD/PRKCQ
15/MSN/MYH7B/MYL1/MYL10/MYL2/MYL9/PPP2R2C/PRKCD/PRKCQ/RUNX1/SYNPO/TUBA8
4/SLC9A2/TGFB2/THBS2/TIMP3/TLR4/WNT5A/WNT9A

LOC102451319/LOC102451362/LOC102452280/LOC102452824/LOC102453747/LOC102453941/LOC10245516

1D4

DGFD/PDGFRA/PPP3CA/TGFB2/TGFBR2/VEGFD

1B/SCN2B/SCN3B

46115/LPAR1/MSN/MYH7B/TLR4/TUBA8/TUBB6/WIPF3

/WNT9A

SPA5/HSPB8/IGFBP3/ITGA4/ITGA8/ITGAV/ITGB2/LOC102444204/LOC102444471/LOC102446475/LOC10244[illegible]

K4/PLK1/PRKCD/PRKCQ/ROR1/STK17A/TGFBR2/TMCC2/TTK/TTN/VRK1/VRK2/WNK2

P2R2C/PPP3CA/PREX1/PSPC1/PTP4A3/PTPRB/PTPRM/PTPRS/RIMBP2/SLC9A2/TMEM132E/WNK2

3/LOC102455777/LOC102456107/LOC102458936/LOC102461260/LOC102464019/LOC106733009/MME/MMP16/

CHL1/VIM/WNT5A/WNT9A

A2/SLC43A3/SLC6A4/SLC9A2/SLC9A9/TIMM9/TMC5/TTI2/WRB/XKRX

R2/PITX1/PITX3/RARB/RUNX1/SHOX/SIM2/SIX1/SIX2/SOX10/SOX13/SOX7/SPI1/SREBF1/TBX15/TBX18/TCI

L1/USP13/USP43/ZBTB44

2R2C/RACGAP1/SETD7/SGO1/SMC2/SMC4/SMYD1/TACC3/TBCCD1/TPX2/TTK/TUBA8/TUBB6

T3/TPM1/TPM3/TTN
/TNNT3/TPM1/TPM3/TTN

PDGFD/PDGFRA/THBS2/VEGFD/ZYX
NC1/TNNT2/TNNT3/TPM1/TPM3
9610/LOC102460124/LOC102464093/LOC112546115/LRRC6/MSN/MYBPC1/MYH7B/MYL1/MYL10/MYL2/MYL3/MY
524/LOC112546115/LPAR1/MSN/MYH7B/MYL10/MYL2/MYL9/MYLK2/MYLK4/PDGFD/PDGFRA/SLC9A2
4/TNFAIP6/VCAN/WNT5A/WNT9A

02443591/LOC102443928/LOC102444204/LOC102444471/LOC102445123/LOC102445365/LOC102445983/LOC

LR/THBS2/TLR4/VEGFD

LOC102464093/LPAR1/MMP2/NOS1/NQO1/PDGFRA/PLCB1/RALGDS/RARB/RGL1/RUNX1/SLC2A4/SPI1/TGFB2/TG

3/LOC102456197/LOC102456325/LOC102456961/LOC102457584/LOC102457786/LOC102458090/LOC1024608

6703/LOC102447192/LOC102447311/LOC102447848/LOC102448731/LOC102449400/LOC102449766/LOC1024

/MMP2/MMP23B/PLAT/PRSS23/QPCT/SERPINH1/TIMP3/UCHL1/USP13/USP43

[illegible]4/TEAD3/TSPAN9/ZBTB44

[illegible]L4/MYL9/MYO18B/MYOT/MYPN/NES/PARVB/PLS1/PRPH/RCSD1/SEPT5/SVIL/SYNC/SYNM/TCAP/TMOD1/TMOD4/[illegible]

[illegible]102446383/LOC102446622/LOC102447223/LOC102447224/LOC102447294/LOC102448731/LOC102449400/L[illegible]

[illegible]FBR2/TPM1/TPM3/TXNRD1/VEGFD/WNT5A/WNT9A/ZBTB44

[illegible]59/LOC102460983/LOC102461635/LOC102461925/LOC102462153/LOC102462624/LOC106732070/MME/MRC2[illegible]

[illegible]49842/LOC102450275/LOC102450447/LOC102451133/LOC102451319/LOC102451731/LOC102452665/LOC10[illegible]

TNNC1/TNNI1/TNNI2/TNNT2/TNNT3/TPM1/TPM3/TTN/TUBA8/TUBB6/VIM

OC102449842/LOC102449893/LOC102450141/LOC102450688/LOC102451133/LOC102451362/LOC102451731/

/NRP1/NT5E/PDGFRA/PECAM1/SELPLG/SEMA7A/SEPT5/TLR4/TNFSF11/TNFSF8/TSPAN2/TSPAN7

2452779/LOC102452796/LOC102452820/LOC102453747/LOC102454753/LOC102455075/LOC102455319/LOC1

/LOC102452280/LOC102452806/LOC102452824/LOC102453747/LOC102454405/LOC102454459/LOC10245516

102456197/LOC102457786/LOC102458809/LOC102459093/LOC102459430/LOC102459532/LOC102459781/LO

8/LOC102455412/LOC102455980/LOC102456223/LOC102456421/LOC102456632/LOC102457786/LOC1024578

C102462085/LOC102462213/LOC102462624/LOC106732354/LOC112544823/LYN/MRC2/MYLIP/MYO18B/MYOF/

340/LOC102458660/LOC102459430/LOC102459610/LOC102462213/LOC102462297/LOC102462326/LOC10246

/MYOT/NCF1/NCF2/NRXN2/OBSCN/OSBPL6/PI15/PKM/PLCB1/PLCD4/PLCH2/PLEK/RAB11FIP5/RAB15/RAB7B/R

62624/LOC102462782/LOC102463089/LOC102463153/LOC102464093/LOC112546115/MATN2/MBP/MME/MSN/M

RACGAP1/RHOJ/RIMBP2/SEC31A/SEPT5/SERPINH1/SREBF1/SRGAP3/TAGLN/TBC1D1/TBC1D4/TCAP/THBS2/TLR

YH7B/MYL1/MYL10/MYL9/MYOF/NEB/PARVB/PKM/P

4/TNFAIP2/TRIM36/TRIP10/TTN/USP43/VLDLR/W
