# Supplementary material for: Liver Antioxidant, Transcriptomic and Metabolomic Responses to Heatwaves in an Aquatic Turtle Species, Pelodiscus sinensis
Source: Animals (Basel). 2026 Jun 17;16(12):1870. doi: 10.3390/ani16121870 (PMC13295678; doi:10.3390/ani16121870)
Supplement: Supplementary file 1 [file animals-16-01870-s001.zip › Supplementary Table S4.pdf]

Single-HW vs CTRL

**Metabolite name**	**Fold change**	***P* value**	**FDR**
S-(-)-ureidoglycolate	2.83	< 0.0001	0.0092
2-(2-Furyl)-3-(5-nitro-2-furyl)acrylamide	0.14	< 0.0001	0.0092
Dihydroisopentenyldehydrorhodopin	14.91	< 0.0001	0.0092
Cytosine	1.75	< 0.0001	0.0092
3-(4-methylthio)butylmalate	4.20	< 0.0001	0.0092
3-acetyloctanal	10.97	< 0.0001	0.0093
xi-2,3-Dihydro-2-oxo-1H-indole-3-acetic acid	13.43	< 0.0001	0.0108
R.g.-Keto I	7.93	< 0.001	0.0145
Mycocyclosin	0.26	< 0.001	0.0145
Dhurrin	0.28	< 0.001	0.0145
N-Acetyl-glucosamine 1-phosphate	7.05	< 0.001	0.0145
26-hydroxybrassinolide	4.84	< 0.001	0.0145
5-Amino-6-ribitylamino uracil	0.44	< 0.001	0.0145
2-Octaprenyl-3-methyl-5-hydroxy-6-methoxy-1,4-benzoqu	7.36	< 0.001	0.0145
Cyclooctat-9-ene-5,7-diol	17.57	< 0.001	0.0150
Allopurinol riboside	9.32	< 0.001	0.0202
Maltotetraose	6.19	< 0.001	0.0216
Coprocholic acid	5.66	< 0.001	0.0233
20-Hydroxyeicosatetraenoic acid	8.32	< 0.001	0.0249
2-((2E)-3,7-Dimethyl-2,6-octadienyl)-5,6-dimethoxy-3-me	2.88	< 0.001	0.0249
N-Didesmethyl-tamoxifen	0.23	< 0.001	0.0263
4-Anilino-4-oxobutanoic acid	0.24	< 0.001	0.0274
2-Methoxyestrone 3-glucuronide	9.79	< 0.001	0.0279
Nicardipine	10.30	< 0.001	0.0279
Indoleacetaldehyde	2.31	< 0.001	0.0294
3-Deoxo-4b-deoxypaxilline	16.04	< 0.001	0.0295
12,13-EpOME	3.32	< 0.001	0.0295
eugeniin	53.82	< 0.001	0.0315
Psoralen	0.24	< 0.001	0.0315
N-Acetylneuraminic acid	0.20	< 0.001	0.0374
Xanthosine	3.74	< 0.001	0.0374
5,10-dihydrophenazine	67.61	< 0.001	0.0374
8-Hydroxyguanine	3.05	< 0.001	0.0418
PC(20_4(8Z,11Z,14Z,17Z)_18_4(6Z,9Z,12Z,15Z))	8.84	< 0.001	0.0418
(3S,5S)-Carbapenam-3-carboxylate	0.31	< 0.001	0.0418
N,N-Dimethylaniline	1.32	< 0.001	0.0418
erythro-6,8-Triacontanediol	10.82	< 0.01	0.0425
Quinaldic acid	2.76	< 0.01	0.0425
Moupinamide	0.36	< 0.01	0.0425
Isobutyrylglycine	1.91	< 0.01	0.0430
Ethionamide sulphoxide	3.88	< 0.01	0.0430
Astaxanthin	8.41	< 0.01	0.0503
Cyclopentolate	3.39	< 0.01	0.0503
Dehydrogenated ticlopidine	5.39	< 0.01	0.0503
Didehydroagroclavine	3.00	< 0.01	0.0503

fecosterol	98.67	< 0.01	0.0503
L-Histidine	0.34	< 0.01	0.0503
2-cis,4-trans-xanthoxin	1.54	< 0.01	0.0503
typhasterol	1.49	< 0.01	0.0503
Neuro_000161	8.19	< 0.01	0.0503
Taurine	6.32	< 0.01	0.0503
3-Epiecdysone	61.72	< 0.01	0.0509
N6-cis-p-Coumaroylserotonin	0.26	< 0.01	0.0512
Coumarin	115.07	< 0.01	0.0512
Hydroxymethyl indol-3-yl ketone	1.20	< 0.01	0.0515
Alkannin	3.70	< 0.01	0.0515
Glycerophosphorylcholine	0.04	< 0.01	0.0516
(Z)-S-1-Propenyl methanesulfinothioate	0.15	< 0.01	0.0521
4-Hydroxydebrisoquine	0.07	< 0.01	0.0534
L-Kynurenine	4.20	< 0.01	0.0537
5-KETE	10.54	< 0.01	0.0537
3-Dehydrosphinganine	9.96	< 0.01	0.0549
L-tetrahomomethionine	3.51	< 0.01	0.0549
Ginsenoside F2	7.54	< 0.01	0.0559
Carbamazepine	0.27	< 0.01	0.0563
Sventenic acid	1.97	< 0.01	0.0563
5-(Hydroxymethyl)-2-methylpyrimidin-4-OL	3.27	< 0.01	0.0585
L-Tyrosine	1.41	< 0.01	0.0606
S-Ribosylhomocysteine	3.10	< 0.01	0.0629
Usnic acid	13.31	< 0.01	0.0629
Inulobiose	0.29	< 0.01	0.0634
8,10-Octacosanedione	0.23	< 0.01	0.0634
Anandamide	27.91	< 0.01	0.0642
3-Amino-2-azepanone	0.18	< 0.01	0.0642
L-Fuculose	3.74	< 0.01	0.0642
Oleamide	5.39	< 0.01	0.0642
Tetramethylpyrazine	0.02	< 0.01	0.0642
Nnal-N-oxide	0.24	< 0.01	0.0691
4α-hydroxymethyl-4β-methyl-5α-cholesta-8,24-dien-3β-ol	7.97	< 0.01	0.0691
Dipentyl phthalate	0.68	< 0.01	0.0691
3,5-Dibromo-4-hydroxybenzoate	6.43	< 0.01	0.0691
trihomomethionine	4.60	< 0.01	0.0691
Methacrylyl-CoA	5.83	< 0.01	0.0698
CoA 8_0;O	7.99	< 0.01	0.0698
Canthaxanthin	15.76	< 0.01	0.0703
N-Phenylacetylaspartic acid	0.38	< 0.01	0.0723
Fructose 1,6-bisphosphate	0.63	< 0.01	0.0723
13-Deoxycarminomycin	1.45	< 0.01	0.0723
PC(P-16_0_18_2)	15.31	< 0.01	0.0734
S-(2-Methylpropionyl)-dihydrolipoamide-E	11.66	< 0.01	0.0734
Flunixin	17.34	< 0.01	0.0740
cis-p-Coumaric acid 4-[apiosyl-(1->2)-glucoside]	157.01	< 0.01	0.0740
SCHEMBL16620138	1.29	< 0.01	0.0749

Kanzonol K	58.40	< 0.01	0.0764
Chlorobactene	3.31	< 0.01	0.0764
Ribonic acid	5.88	< 0.01	0.0780
3-Hexen-1-ol	0.48	< 0.01	0.0780
L-Glutamic acid	0.24	< 0.01	0.0785
8-Demethyl-8-alpha-L-rhamnosyltetracenomycin C	17.55	< 0.01	0.0823
13-HOTE	4.04	< 0.01	0.0823
DL-Malic acid	0.50	< 0.01	0.0826
Megalomicin C1	37.41	< 0.01	0.0844
2,6-Diethylaniline	3.69	< 0.01	0.0863
cis,cis-3,6-Dodecadienoyl-CoA	17.22	< 0.01	0.0865
Androstanediol-17g	0.20	< 0.01	0.0865
L-Ornithine	0.21	< 0.01	0.0868
Avermectin B1a aglycone	1.81	< 0.01	0.0868
PC(18_2_18_2)	8.67	< 0.01	0.0872
Sophoraflavanone G	4.36	< 0.01	0.0896
D-Fructuronic acid	2.55	< 0.01	0.0913
2-Hydroxyhexadecanoic acid	1.86	< 0.01	0.0913
ST 19_0;O2	0.26	< 0.01	0.0913
Galactosamine	9.95	< 0.01	0.0913
L-Glutamine	0.25	< 0.01	0.0923
D-Erythrose 4-phosphate	6.98	< 0.01	0.0923
Allantoic acid	3.42	< 0.01	0.0923
Linoleamide	5.52	< 0.01	0.0923
Benzyl benzoate	0.09	< 0.01	0.0923
(Z)-4',6-Dihydroxyaurone	3.35	< 0.01	0.0923
Aloin	25.18	< 0.01	0.0923
Paxilline	36.62	< 0.01	0.0937
gamma-Glutamyl-gamma-aminobutyraldehyde	21.43	< 0.01	0.0946
gamma-Glutamylcysteine	5.37	< 0.01	0.0974
N(6)-Methyllysine	1.05	< 0.01	0.0977
Styrene Oxide	0.62	< 0.01	0.0989
Limonene-1,2-diol	0.56	< 0.01	0.1012
Isobutylpropylamine	1.53	< 0.01	0.1012
Heptadecanoic acid	2.30	< 0.01	0.1012
Quercetin 3-galactoside 7-rhamnoside	61.31	< 0.01	0.1021
4,4-Diaponeurosporene	50.04	< 0.01	0.1027
alpha,alpha-Trehalose 6-mycolate	6.49	< 0.01	0.1030
Tetrahydrospirilloxanthin	3.01	< 0.01	0.1036
delta-Valerolactone	1.71	< 0.01	0.1036
(2Z,6E)-3,7,11,15,19-Pentamethyl-2,6-eicosadien-1-ol	4.62	< 0.01	0.1047
Aminopropylcadaverine	3.20	< 0.01	0.1047
Equilin	2.75	< 0.01	0.1064
3-(2-hydroxyphenyl)propionate	1.83	< 0.01	0.1064
Piperine	0.48	< 0.01	0.1064
PE-NMe2(18_1(9Z)_18_3(9Z,12Z,15Z))	9.63	< 0.01	0.1073
gamma-Tocotrienol	0.18	< 0.01	0.1073
Maritinamine	0.58	< 0.01	0.1073

Trigonelline (N'-methylnicotinate)	0.01	< 0.01	0.1073
Citicoline	10.68	< 0.01	0.1078
Carisoprodol	47.65	< 0.01	0.1078
Glycogen	0.59	< 0.01	0.1078
Artemetin	3.01	< 0.01	0.1078
1H-Imidazole-1-acetic acid	25.60	< 0.01	0.1078
N-(3-Methylbut-2-EN-1-YL)-9H-purin-6-amine	4.57	< 0.01	0.1078
Amino aspartic acid	1.62	< 0.01	0.1078
Xanthylic acid	0.61	< 0.01	0.1078
Lecithin	4.62	< 0.01	0.1078
Iminodiacetate (IDA)	3.20	< 0.01	0.1078
Ubiquinol 8	4.80	< 0.01	0.1078
1-Methyladenosine	1.94	< 0.01	0.1078
beta-Geraniol	0.16	< 0.01	0.1078
Tryprostatin B	0.31	0.0101	0.1090
N-Fructosyl isoleucine	5.31	0.0103	0.1101
Cysteineglutathione disulfide	0.32	0.0105	0.1123
α-Methyltryptophan	1.60	0.0107	0.1129
Dihydromacarpine	0.34	0.0107	0.1129
Acetyl-N-formyl-5-methoxykynurenamine	30.56	0.0112	0.1162
Alanylglutamic acid	0.41	0.0112	0.1162
Glyceric acid 1,3-biphosphate	11.66	0.0118	0.1221
(S)-scoulerine	0.37	0.0119	0.1221
Goltix	0.38	0.0120	0.1224
Leukoefdin	0.26	0.0122	0.1229
10S-HpOME	43.54	0.0123	0.1229
Naringenin	0.23	0.0124	0.1229
Gibberellin A3	0.14	0.0124	0.1229
CDP	8.30	0.0125	0.1229
(-)-Thebaine	0.31	0.0125	0.1229
trans-Methylbixin	9.41	0.0126	0.1229
L-Isoleucine	0.32	0.0127	0.1240
2-((N-(1-(1H-Imidazol-4-yl)-2-propyl)imino)phenylmethyl	0.19	0.0133	0.1278
4-Hydroxyphenylpyruvic acid	0.90	0.0134	0.1278
Harmolol	1.31	0.0135	0.1278
PC(20_3(8Z,11Z,14Z)_20_5(5Z,8Z,11Z,14Z,17Z))	13.02	0.0135	0.1278
Trioxilin A3	11.72	0.0136	0.1278
8-Hydroxy-2-methoxy-6-methyl-1,4-naphthoquinone	41.09	0.0136	0.1278
Caldariellaquinone	3.34	0.0137	0.1279
Indoleacetylaspartate	0.37	0.0140	0.1299
LysoSM(d18_1)	3.79	0.0140	0.1299
pyrethrin I	39.49	0.0142	0.1309
Prostaglandin-c2	0.83	0.0146	0.1338
Nudifloramide	1.48	0.0151	0.1375
Torulene	0.25	0.0152	0.1375
Guanosine	0.84	0.0154	0.1376
Pivmecillinam	0.05	0.0154	0.1376
Estriol	25.73	0.0155	0.1384

Cyclic ADP-ribose	3.09	0.0158	0.1400
Pseudoionone	4.65	0.0161	0.1414
4-Aminobutanoyl-CoA	3.87	0.0161	0.1414
3-Hydroxy-2H-pyran-2-one	1.56	0.0163	0.1417
Germacrene A acid	11.99	0.0166	0.1442
3-Methyl-3H-imidazo[4,5-f]quinoxalin-2-amine	1.19	0.0168	0.1454
MFCD00049038	3.30	0.0174	0.1498
Glycohyodeoxycholic acid	3.91	0.0176	0.1501
2-Deamino-2-hydroxy-6-dehydroparomamine	0.06	0.0178	0.1507
5-methylthioadenosine (MTA)	0.18	0.0178	0.1507
Dihydromethanophenazine	41.83	0.0181	0.1525
Levobunolol	6.30	0.0182	0.1525
PC-M6	25.05	0.0185	0.1535
Pyridoxamine	3.49	0.0185	0.1535
Phenylalanylleucine	1.04	0.0187	0.1541
5-O-beta-D-Mycaminosyltylactone	13.39	0.0188	0.1543
Quetiapine	2.73	0.0192	0.1572
3-deoxy-D-manno-octulosonate	1.56	0.0196	0.1593
Hypoxanthine	0.69	0.0199	0.1604
Rotenone	0.02	0.0202	0.1604
Methyl indole-3-propanoate	0.74	0.0202	0.1604
5-Carboxy-2-pentenoyl-CoA	143.35	0.0203	0.1604
Coclaurine	18.16	0.0203	0.1604
Terpendole E	1.57	0.0204	0.1604
Citraconic acid	2.75	0.0205	0.1604
Oxanthrene	2.54	0.0206	0.1604
Glucohesperalin	9.72	0.0206	0.1604
Benzoic acid	2.20	0.0209	0.1610
3-Ethyl-2-hydroxy-4-methyl-2-cyclopenten-1-one	1.46	0.0209	0.1610
Dihydrotestosterone	1.36	0.0209	0.1610
PC(P-16_0_22_0)	8.65	0.0212	0.1615
corynantheal	2.52	0.0213	0.1615
L-Olivosyl-oleandolide	0.11	0.0215	0.1615
Naringin	5.85	0.0215	0.1615
(5Z,9E,14Z)-(8xi,11R,12S)-11,12-epoxy-8-hydroxyicosa-5	0.38	0.0215	0.1615
Choline sulfate	1.67	0.0216	0.1616
10-epi-gamma-eudesmol	2.53	0.0220	0.1642
2-(beta-D-Glucosyl)-sn-glycerol 3-phosphate	0.30	0.0223	0.1645
Indolepyruvate	32.41	0.0223	0.1645
Hovenidulcigenin A	2.80	0.0224	0.1645
LysoPE (17_0_0_0)	4.08	0.0225	0.1645
Oryzalexin C	4.54	0.0232	0.1688
Galactosylsphingosine	16.07	0.0235	0.1690
beta-L-Arabinose 1-phosphate	0.33	0.0236	0.1690
3-Aminobutanoic acid	0.24	0.0237	0.1690
(2R,4S)-Pyrrolidine-2,4-dicarboxylic acid	2.12	0.0237	0.1690
Tuftsin	0.20	0.0238	0.1690
Sucrose 6-phosphate	3.05	0.0241	0.1690

Crocetindial	4.81	0.0242	0.1690
CAR 3_0	10.20	0.0242	0.1690
Dyspropterin	2.43	0.0242	0.1690
PC(18_4(6Z,9Z,12Z,15Z)_22_5(7Z,10Z,13Z,16Z,19Z))	5.33	0.0242	0.1690
3-Hydroxy-11Z-octadecenoylcarnitine	12.38	0.0243	0.1690
6-methoxy-3-methyl-2-all-trans-hexaprenyl-1,4-benzoquin	3.58	0.0247	0.1708
N-Benzoyl-4-methoxyanthranilate	4.89	0.0248	0.1711
D-Arabitol	2.63	0.0254	0.1730
Cholic acid	2.81	0.0254	0.1730
3-hydroxymyristate	0.33	0.0255	0.1730
5-hydroxyindole thiazolidine carboxylate	0.26	0.0257	0.1730
7-hydroxy-4-isopropenyl-7-methyloxepan-2-one	0.38	0.0257	0.1730
Indoline	0.64	0.0257	0.1730
L-Tryptophan	41.32	0.0262	0.1757
6'-O-p-Coumaroyltrifolin	27.30	0.0263	0.1757
6-Chloro-N-(1-methylethyl)-1,3,5-triazine-2,4-diamine	0.27	0.0266	0.1767
Strigol	0.53	0.0267	0.1767
1,2-di-O-sinapoyl-beta-D-glucose	52.86	0.0269	0.1772
FA 18_1	2.11	0.0270	0.1772
dihydro-3-hydroxy-4,4-dimethyl- 2(3H)-Furanone	0.58	0.0271	0.1772
Manzamine A	9.49	0.0275	0.1781
N-Acetyl-b-glucosaminylamine	0.52	0.0276	0.1781
dolichyl beta-D-glucosyl phosphate	9.98	0.0276	0.1781
LysoPE (16_0_0_0)	1.57	0.0276	0.1781
Tembetarine	6.99	0.0278	0.1788
Cefdinir	11.19	0.0281	0.1797
carlactone	0.25	0.0283	0.1803
Gentamicinc1A	0.33	0.0286	0.1811
6alpha,8beta-Dihydroxygermacra-1(10),4,11(13)-trien-12-	1.29	0.0286	0.1811
Phosphoribosyl formamidocarboxamide	84.15	0.0290	0.1818
7-Ketodeoxycholic acid	46.23	0.0291	0.1818
L-Arabinonate	0.58	0.0292	0.1818
Dibenzothiophene 5-oxide	2.15	0.0292	0.1818
L-Arabinose	7.85	0.0294	0.1822
Calcipotriol	5.11	0.0295	0.1822
Eriodictyol	0.57	0.0298	0.1837
2-Deoxycastasterone	5.59	0.0300	0.1837
Dicrocin	0.47	0.0301	0.1837
6-Deoxy-6-sulfoglucono-1,5-lactone	0.36	0.0303	0.1837
Camalexin	1.12	0.0304	0.1837
Betaine	1.43	0.0305	0.1837
10-Formyltetrahydrofolate	13.12	0.0306	0.1837
Phycocyanobilin	3.17	0.0306	0.1837
Octanal	0.58	0.0307	0.1837
SCHEMBL21051772	1.35	0.0308	0.1838
Benzo[a]pyrene-4,5-oxide	8.22	0.0311	0.1851
1,3-alpha-D-Mannosyl-1,2-alpha-D-mannosyl-1,2-alpha-D	8.41	0.0318	0.1875
1-Acetoxy-2-hydroxy-16-heptadecen-4-one	0.55	0.0319	0.1875

Prostaglandin E1	2.28	0.0320	0.1875
2-Oxazolidinone	1.18	0.0323	0.1875
FO 2546M	0.26	0.0324	0.1875
13(S)-Hydroperoxylinolenic acid	1.46	0.0325	0.1875
Cinnatriacetin A	5.69	0.0325	0.1875
PC(20_4(8Z,11Z,14Z,17Z)_22_6(4Z,7Z,10Z,13Z,16Z,19Z	86.50	0.0325	0.1875
5-Hydroxymethyl-2-furancarboxaldehyde	0.38	0.0326	0.1875
6,8a-Seco-6,8a-deoxy-5-oxoavermectin 2a aglycone	1.58	0.0327	0.1875
(+)-Gallocatechin	0.52	0.0328	0.1875
2-Hydroxyoctadecanoic acid	1.80	0.0332	0.1888
Isopropanolamine	0.45	0.0332	0.1888
Riboflavin cyclic-4',5'-phosphate	2.08	0.0336	0.1892
PC(15_0_20_5(5Z,8Z,11Z,14Z,17Z))	2.78	0.0337	0.1892
DG(10_0_0_0_8_0)	2.26	0.0337	0.1892
2-Hydroxybenzaldehyde	1.36	0.0337	0.1892
25-Hydroxyvitamin D3-26,23-lactol	18.21	0.0343	0.1918
FA 7_3;O4	1.45	0.0344	0.1918
N-Retinylidene-N-retinylethanolamine	11.24	0.0346	0.1921
6-Hydroxy-8-pentacosanone	0.30	0.0348	0.1921
beta-Peltatin A methyl ether	3.30	0.0349	0.1921
Furcatin	3.32	0.0349	0.1921
2-Isopropyl citrate	0.16	0.0354	0.1939
Prostaglandin F1a	1.58	0.0357	0.1950
3-Methylcrotonyl-CoA	4.91	0.0358	0.1953
L-Hexahydro-3-imino-1,2,4-oxadiazepine-3-carboxylic aci	1.32	0.0362	0.1958
Bisnorbiotin	0.15	0.0362	0.1958
9-methylthiononanaldoxime	0.44	0.0363	0.1958
aphidicolin	11.54	0.0365	0.1961
JI-20B	7.25	0.0368	0.1974
Aminoadipic acid	3.00	0.0371	0.1975
Dopamine	2.63	0.0372	0.1975
LysoPC(15_0_0_0)	27.02	0.0373	0.1975
1,5-Anhydrosorbitol	2.14	0.0374	0.1975
S-(Indolylmethylthiohydroximoyl)-L-cysteine	4.06	0.0374	0.1975
Annuolide G	2.05	0.0380	0.1996
Dantrolene	0.12	0.0381	0.1997
Asymmetric dimethylarginine	0.49	0.0382	0.1997
3-Hydroxy-3-methylglutaryl-CoA	29.97	0.0383	0.1997
1,3,7-trimethyl-5-hydroxyisourate	1.81	0.0386	0.2005
2,5-Bis(hydroxymethyl)furan	1.45	0.0388	0.2011
Dihydrosterigmatocystin	12.99	0.0389	0.2011
10-Apo-beta-carotenal	18.22	0.0393	0.2018
3-O-Methylkaempferol	36.30	0.0393	0.2018
Dihydroclavaminate	1.27	0.0395	0.2018
Oxaloacetate	2.49	0.0396	0.2018
S-adenosylhomocysteine (SAH)	12.17	0.0400	0.2029
Quinone	0.23	0.0402	0.2029
PC(20_5(5Z,8Z,11Z,14Z,17Z)_18_1(11Z))	2.47	0.0402	0.2029

14alpha-Hydroxymethylsteroid	0.90	0.0403	0.2029
PC(22_6(4Z,7Z,10Z,13Z,16Z,19Z)_18_4(6Z,9Z,12Z,15Z))	16.14	0.0404	0.2029
Formononetin	70.75	0.0407	0.2038
dUDP	0.89	0.0410	0.2042
Dicyclohexylamine	3.29	0.0410	0.2042
N-acetylglutamate	1.20	0.0414	0.2054
Paraoxon	16.39	0.0415	0.2054
Cyclic cmp	3.65	0.0418	0.2063
Aflatoxin M1	27.57	0.0420	0.2065
N-Methyl-4-dimethylallyltryptophan	46.43	0.0423	0.2071
3-Phenylcatechol	1.87	0.0423	0.2071
Pyrrolidine-2-carboxamide	2.28	0.0427	0.2082
2-Acetylpyridine	0.14	0.0435	0.2116
gamma-Glutamyltyramine	0.39	0.0438	0.2122
Campesterol	2.53	0.0444	0.2135
LysoPC(18_3(6Z,9Z,12Z)_0_0)	0.30	0.0444	0.2135
sn-glycero-3-Phosphoethanolamine	0.17	0.0446	0.2135
Autumnaline	0.20	0.0448	0.2135
2-Oxokanamycin	30.78	0.0449	0.2135
2-[Bis-(2-hydroxy-ethyl)-amino]-2-hydroxymethyl-propan	0.68	0.0450	0.2135
Roquefortine F	0.36	0.0451	0.2135
8-Amino-7-oxononanoic acid	4.14	0.0452	0.2135
Hexazinone	17.99	0.0453	0.2135
Margaroylglycine	8.03	0.0454	0.2135
LysoPC(20_3(5Z,8Z,11Z)_0_0)	0.64	0.0454	0.2135
Homo-L-arginine	0.52	0.0458	0.2148
Multifidol	0.61	0.0459	0.2148
PC(24_0_22_4(7Z,10Z,13Z,16Z))	8.66	0.0463	0.2158
1,3-dihydroxy-N-methylacridone	0.16	0.0468	0.2164
Guanine	1.60	0.0469	0.2164
28-Homobrassinolide	18.31	0.0469	0.2164
(ent-6alpha,7alpha)-6,7-Dihydroxy-16-kauren-19-oic acid	0.32	0.0469	0.2164
Dubinidine	0.32	0.0471	0.2164
Prostaglandin F-main urinary metabolite	0.36	0.0475	0.2179
7Z-tetradecenoic acid	17.58	0.0481	0.2195
Dehydrozingerone	0.46	0.0483	0.2195
Isonicotinamide	1.39	0.0484	0.2195
Isoformononetin	0.61	0.0487	0.2195
LysoPC(22_6_0_0)	11.16	0.0488	0.2195
2-Methylacetoacetyl-CoA	11.15	0.0488	0.2195
O-Demethylencainide	2.02	0.0489	0.2195
Deoxyuridine	2.30	0.0490	0.2195
Albendazole-2-aminosulfone	1.91	0.0490	0.2195
Resveratrol	5.65	0.0493	0.2201

Ion mode	Exact mas	Precursor type	Class	Subclass	KEGG Pathway	CAS
pos	133.0249	[M-NH3+H]+				
pos	248.0433	[M-OH+H]+				18819-45-9
neg	622.5113	[M-NH3-H]-				
pos	111.0433	[M+H]+	Diazines	Pyrimidine	Global and overview	71-30-7
neg	236.0718	[M-NH3-H]-			Biosynthesis of other secondary	
pos	170.1307	[M+H]+			Biosynthesis of other secondary	
neg	191.0582	[M-H]-	Indoles and de	Indolyl car	Amino acid metabol	2971-31-5
pos	582.4437	[M+NH4]+			Metabolism of terpe	38748-34-4
pos	324.111	[M-OH+H]+				
pos	311.1005	[M+H]+	Organooxyger	Carbohydrates and carbohydrate		21401-21-8
pos	301.0563	[M-H2O+H]+	Organooxyger	Carbohydrates and carbohydrate		901851-43
neg	496.34	[M-NH3-H]-			Metabolism of terpenoids and po	
pos	276.107	[M+NH4]+	Organooxyger	Carbohydr	Energy metabolism;	17014-74-1
neg	712.543	[M+HCO3]-			Metabolism of cofac	35278-60-5
pos	306.2559	[M+Na]+			Biosynthesis of other secondary	
pos	268.0808	[M+H]+	Pyrazolo[3,4-d]pyrimidine glycosides			16220-07-8
pos	666.2219	[M+Na]+	Organooxyger	Carbohydrates and carbohydrate		34612-38-9
neg	450.3345	[M-H]-	Steroids and s	Bile acids,	Lipid metabolism; G	547-98-8
pos	320.2351	[M+H]+	Fatty Acyls	Fatty acids	Lipid metabolism; G	79551-86-1
pos	320.1988	[M-CO2+H]+				5677-55-4
pos	343.1936	[M+NH4]+	Stilbenes		Xenobiotics biodegr	80234-20-4
pos	193.0739	[M+H]+	Benzene and s	Anilides		
pos	476.2046	[M-OH+H]+	Steroids and s	Steroidal g	Lipid metabolism	25577-70-1
pos	479.2056	[M+K]+	Pyridines and	Hydropyridines		106664-28
neg	159.0684	[M+HCO3]-	Indoles and de	Indoles	Amino acid metabol	2591-98-2
neg	405.2668	[M+HCOO]-			Biosynthesis of other secondary	
pos	296.2351	[M+H]+	Fatty Acyls	Fatty acids and conjugates		32381-42-1
pos	938.1025	[M-CO2+H]+	Tannins	Hydrolyzable tannins		58970-75-5
neg	186.0317	[M-H]-	Coumarins an	Furanocou	Chemical structure t	66-97-7
pos	309.106	[M+H]+				114942-08
neg	284.0757	[M-NH3-H]-	Purine nucleosides		Membrane transport	146-80-5
pos	182.0844	[M+K]+			Biosynthesis of othe	613-32-1
pos	167.0443	[M+H]+	Imidazopyrim	Purines and purine derivatives		5614-64-2
pos	801.5308	[M-CO2+H]+	Glycerophosp	Glycerophosphocholines		
neg	155.0582	[M-H]-			Biosynthesis of othe	123409-86-
pos	121.0891	[M+NH4]+	Organonitroge	Amines		101357-19
pos	454.475	[M+NH4]+	Fatty Acyls	Fatty alcohols		155800-87
neg	173.0477	[M-H]-	Quinolines an	Quinoline carboxylic acids		1199266-7
pos	313.1314	[M-H2O+H]+	Phenols	Methoxyphenols		640235-90
pos	145.0739	[M+H]+	Carboxylic ac	Amino acids, peptides, and anal		1219795-0
pos	182.0514	[M-OH+H]+	Pyridines and derivatives			536-28-7
pos	596.3865	[M+Na]+	Prenol lipids	Tetraterpe	Global and overview	472-61-7
pos	291.1834	[M+H]+	Benzene and substituted derivatives			512-15-2
pos	261.0379	[M+H]+	Thienopyridines			
pos	237.1392	[M-NH3+H]+			Biosynthesis of other secondary	

pos	398.3549	[M+H]+	Steroids and s	Ergostane	Lipid metabolism; G	516-86-9
neg	155.0695	[M-H]-	Carboxylic ac	Amino aci	Cancer: overview; M	71-00-1
neg	250.1569	[M-H]-	Prenol lipids	Sesquiterp	Chemical structure t	8066-07-7
pos	448.3552	[M+H]+	Steroids and s	Bile acids,	Global and overview	87734-68-
neg	399.1682	[M+HCO3]-			Chemical structure t	14686-61-
neg	125.0147	[M-H]-	Organic sulfo	Organosul	Membrane transport	107-35-7
pos	464.3138	[M-CO2+H]+			Metabolism of terpenoids and p	
pos	322.1317	[M+K]+	Indoles and d	Tryptamines and derivatives		201301-83
pos	146.0368	[M-NH3+H]+	Coumarins and derivative		Biosynthesis of othe	91-64-5
pos	175.0633	[M-OH+H]+	Indoles and d	Indoles		2400-51-3
neg	288.0998	[M-H]-				23444-65-
pos	258.1106	[M+H]+			Lipid metabolism; C	28319-77-
pos	136.0017	[M+NH4]+	Thiosulfinic acid esters			119052-99
pos	191.1059	[M+NH4]+	Tetrahydroisoquinolines			59333-79-
pos	208.0848	[M+H]+	Organooxyge	Carbonyl c	Amino acid metabol	2922-83-0
pos	318.2195	[M+H]+	Fatty Acyls	Fatty acids	Lipid metabolism	106154-18
pos	299.2824	[M+H]+	Organooxyge	Carbonyl c	Lipid metabolism; G	16105-69-
pos	205.1136	[M+K]+			Biosynthesis of other secondary	
pos	784.4973	[M+NH4]+				62025-49-
pos	236.095	[M+NH4]+	Benzazepines	Dibenzaze	Xenobiotics biodegr	298-46-4
neg	318.2195	[M-H]-			Global and overview	21435-01-
pos	140.0586	[M-H2O+H]+				698-30-6
pos	181.0739	[M+H]+	Carboxylic ac	Amino aci	Cancer: overview; M	60-18-4
pos	267.0777	[M-H2O+H]+			Amino acid metabol	15912-98-
pos	344.0896	[M+H]+				7562-61-0
pos	342.1162	[M+NH4]+	Organooxyge	Carbohydrates and carbohydrate		470-58-6
pos	422.4124	[M-OH+H]+	Organooxyge	Carbonyl compounds		
pos	347.2824	[M-CO2+H]+	Organonitrog	Amines	Nervous system; Se	924894-98
pos	128.095	[M+H]+				21568-87-
neg	164.0685	[M+HCOO]-	Fatty Acyls	Fatty alcoh	Carbohydrate metab	13074-08-
pos	281.2719	[M+H]+	Fatty Acyls	Fatty amides		301-02-0
pos	136.1	[M+H]+	Diazines	Pyrazines		1124-11-4
pos	225.1113	[M+H]+	Pyridines and	Pyridinium	Xenobiotics biodegr	85352-99-
neg	428.3654	[M-NH3-H]-	Prenol lipids	Triterpenoids		616897-75
pos	306.1831	[M+NH4]+	Benzene and	Benzoic acids and derivatives		131-18-0
pos	293.8527	[M+H]+			Xenobiotics biodegr	3337-62-0
neg	191.098	[M+HCOO]-			Biosynthesis of other secondary	
pos	835.1414	[M+NH4]+			Amino acid metabol	6008-91-9
pos	909.2146	[M+NH4]+			Lipid metabolism; G	79171-48-
pos	564.3967	[M-NH3+H]+	Prenol lipids	Tetraterpe	Global and overview	514-78-3
neg	251.0794	[M+HCO3]-	Carboxylic ac	Amino acids, peptides, and anal		2752-32-1
pos	339.9961	[M+Na]+			Cancer: overview; S	34693-23-
pos	499.1842	[M+Na]+			Global and overview	76034-18-
pos	741.5672	[M+K]+	Glycerophosp	Glycerophosphocholines		
pos	277.117	[M+H]+	Fatty Acyls	Fatty amides		958734-24
neg	296.0773	[M-H]-	Benzene and	Trifluoromethylbenzenes		38677-85-
pos	458.1424	[M+Na]+	Organooxyge	Carbohydrates and carbohydrate		95-01-2
pos	187.0481	[M+NH4]+			Amino acid metabol	185103-33

pos	436.1886	[M+H]+	Isoflavonoids	Isoflavans		156281-30
pos	532.4069	[M+NH4]+			Metabolism of terpe	2932-09-4
neg	166.0477	[M-H]-	Organooxyge	Carbohydrates and carbohydrat		17812-24-
pos	100.0888	[M+Na]+	Fatty Acyls	Fatty alcoh	Lipid metabolism; G	928-96-1
pos	147.0532	[M+H]+	Carboxylic ac	Amino aci	Neurodegenerative d	56-86-0
pos	604.1428	[M+K]+			Global and overview maps; Met	
neg	294.2195	[M-H]-	Fatty Acyls	Lineolic ac	Lipid metabolism	87984-82-
neg	134.0215	[M-H]-	Hydroxy acids	Beta hydro	Cancer: overview; C	97-67-6
pos	960.577	[M-OH+H]+			Global and overview	49669-76-
pos	149.1204	[M+NH4]+	Benzene and s	Aniline and substituted anilines		579-66-8
pos	945.251	[M+NH4]+	Fatty Acyls	Fatty acyl	Lipid metabolism	
neg	468.2723	[M-H]-	Steroids and s	Steroidal glycosides		
pos	132.0899	[M+H]+	Carboxylic ac	Amino aci	Membrane transport	3184-13-2
pos	584.3349	[M-OH+H]+			Global and overview maps; Met	
pos	781.5621	[M+H]+	Glycerophosp	Glycerophosphocholines		998-06-1
neg	424.1886	[M-NH3-H]-				97938-30-
neg	194.0427	[2M-H]-			Carbohydrate metab	13425-76-
neg	272.2351	[M-H]-	Fatty Acyls	Fatty acids and conjugates		16452-52-
neg	292.2402	[M+HCOO]-				571-20-0
pos	179.0794	[M+H]+			Carbohydrate metab	7535-00-4
pos	146.0691	[M+H]+	Carboxylic ac	Amino aci	Cancer: overview; M	56-85-9
pos	200.0086	[M+NH4]+	Organooxyge	Carbohydr	Metabolism of cofac	585-18-2
pos	176.0546	[M-NH3+H]+	Carboxylic ac	Amino aci	Global and overview	99-16-1
pos	279.2562	[M+H]+	Fatty Acyls	Fatty amides		3072-13-7
pos	212.0837	[M+K]+	Benzene and s	Benzoic acids and derivatives		347840-01
neg	254.0579	[2M-H]-	Aurone flavonoids			5786-54-9
pos	418.1264	[M+NH4]+				452311-56
neg	435.241	[M-H]-	Naphthopyrans		Biosynthesis of othe	1233509-8
pos	216.111	[M+NH4]+			Amino acid metabolism; Global	
neg	250.0623	[M-NH3-H]-	Carboxylic ac	Amino aci	Cell growth and dea	636-58-8
pos	160.1212	[M+H]+	Carboxylic ac	Amino acids, peptides, and anal		1188-07-4
pos	120.0575	[M-H2O+H]+	Benzene and substituted derivatives			192506-02
neg	170.1307	[M+HCOO]-			Metabolism of terpe	38630-75-
pos	115.1361	[M+H]+	Organonitrog	Amines		39190-66-
pos	270.2559	[M+NH4]+	Fatty Acyls	Fatty acids and conjugates		506-12-7
pos	610.1534	[M+NH4]+	Flavonoids	Flavonoid glycosides		38784-81-
neg	402.3286	[M+HCO3]-			Metabolism of terpe	53872-50-
pos	818.5755	[M-NH3+H]+				
neg	600.4906	[M-H2O-H]-			Global and overview	13833-01-
pos	100.0524	[M+H]+	Lactones	Delta valerolactones		42932-61-
pos	364.3705	[M-NH3+H]+	Prenol lipids	Sesterterpenoids		26549-03-
pos	159.1735	[M+H]+	Organonitrog	Amines	Metabolism of other	56-19-9
neg	268.1463	[M-H]-				474-86-2
neg	166.063	[M-H]-	Phenylpropanoic acids		Amino acid metabol	495-78-3
neg	285.1365	[M+HCOO]-			Chemical structure t	94-62-2
pos	767.5465	[M+H]+	Glycerophosp	Glycerophosphoethanolamines		
neg	410.3185	[M-H]-			Metabolism of cofac	14101-61-
neg	275.1521	[M-H]-			Biosynthesis of other secondary	

pos	137.0477	[M+NH4]+			Metabolism of cofac	535-83-1
neg	488.1073	[M+HCO3]-			Lipid metabolism; C	987-78-0
pos	260.1736	[M+K]+	Carboxylic ac	Amino acids, peptides, and anal		78-44-4
neg	666.2219	[M+HCOO]-	Organooxyge	Carbohydr	Endocrine system	149820-99
pos	388.1158	[M+H]+	Flavonoids	O-methylated flavonoids		479-90-3
pos	126.0429	[M+H]+	Carboxylic ac	Amino acids, peptides, and anal		22884-10-
neg	203.1171	[M-H]-	Imidazopyrim	Purines an	Signal transduction;	2365-40-4
pos	148.0484	[M-H2O+H]+	Carboxylic ac	Amino acids, peptides, and anal		92751-07-
neg	364.042	[M+HCOO]-	Purine nucleo	Purine ribo	Chemical structure t	523-98-8
pos	757.5621	[M+H]+				8002-43-5
neg	133.0375	[M-H]-	Carboxylic ac	Amino acids, peptides, and anal		142-73-4
neg	728.5743	[M+HCOO]-	Prenol lipids	Quinone and hydroquinone lipi		74075-00-
neg	281.1124	[M-H]-	Purine nucleosides			15763-06-
neg	154.1358	[M+HCOO]-	Prenol lipids	Monoterpe	Chemical structure t	106-24-1
pos	351.1947	[M-CO2+H]+			Biosynthesis of othe	179936-52
neg	293.1474	[M-H]-	Organooxyge	Alcohols and polyols		87304-79-
neg	426.0879	[M-H]-	Carboxylic ac	Amino aci	Amino acid metabol	13081-14-
pos	218.1055	[M-OH+H]+	Carboxylic ac	Amino acids, peptides, and anal		153-91-3
pos	393.1212	[2M+H]+			Biosynthesis of othe	77785-12-
pos	264.111	[M+NH4]+	Organooxyge	Carbonyl c	Amino acid metabol	52450-38-
pos	218.0903	[M+H]+	Carboxylic ac	Amino acids, peptides, and anal		16809-27-
neg	265.9593	[2M-H]-	Organic phos	Phosphate	Carbohydrate metab	38168-82-
pos	327.1471	[M+H]+	Protoberberine alkaloids		Chemical structure t	6451-72-5
pos	202.0855	[M+H]+	Triazines	1,2,4-triazines		41394-05-
neg	322.0689	[M-H2O-H]-			Biosynthesis of othe	98919-67-
pos	314.2457	[M-OH+H]+				
pos	272.0685	[M-H2O+H]+			Chemical structure t	67604-48-
neg	346.1416	[M-H]-	Prenol lipids	Diterpenoi	Global and overview	77-06-5
neg	403.0182	[M-H]-	Pyrimidine nu	Pyrimidine	Global and overview	102601-46
pos	311.1521	[M-OH+H]+	Morphinans		Chemical structure t	115-37-7
neg	408.23	[M+HCO3]-	Prenol lipids	Diterpenoids		62697-46-
neg	131.0946	[M-H]-	Carboxylic ac	Amino aci	Cancer: overview; M	73-32-5
neg	305.1528	[M-H]-	Benzene and	Diphenylmethanes		139191-80
pos	180.0423	[M-H2O+H]+	Benzene and	Phenylpyr	Metabolism of cofac	156-39-8
pos	200.095	[M+NH4]+	Harmala alkaloids			525-57-5
pos	829.5621	[M-CO2+H]+	Glycerophosp	Glycerophosphocholines		
neg	354.2406	[M+HCO3]-			Lipid metabolism	68860-46-
pos	218.0579	[M+H]+	Naphthalenes	Naphthoquinones		1589-92-0
pos	630.4504	[M+NH4]+				63693-26-
pos	290.0903	[M+NH4]+				2456-73-7
pos	465.3457	[M+NH4]+	Sphingolipids	Phosphosp	Lipid metabolism	
pos	328.2038	[M+K]+				121-21-1
pos	334.2144	[M-H2O+H]+	Fatty Acyls	Eicosanoid	Lipid metabolism; N	49825-91-
pos	152.0586	[M+NH4]+	Pyridines and	Pyridineca	Metabolism of cofac	701-44-0
neg	534.4225	[M+HCOO]-			Global and overview	547-23-9
pos	283.0917	[M+H]+	Purine nucleosides		Membrane transport	118-00-3
pos	439.2141	[M+H]+	Carboxylic ac	Amino acids, peptides, and anal		32886-97-
pos	288.1725	[M+H]+	Steroids and s	Estrane ste	Lipid metabolism; T	221093-41

pos	541.0611	[M+NH4]+			Signal transduction;	119340-53-
neg	192.1514	[M+HCOO]-	Prenol lipids	Monoterpenoids		3548-78-5
pos	852.168	[M-CO2+H]+				
pos	112.016	[M+H]+	Pyrans	Pyranones and derivatives		496-64-0
neg	234.162	[M-H]-			Global and overview	347377-92-
pos	199.0858	[M+H]+	Diazanaphtha	Benzodiazines		108354-47
neg	285.0629	[2M-H]-				892-48-8
neg	449.3141	[M-H]-	Steroids and s	Bile acids, alcohols and derivati		13042-33-(
pos	322.1376	[M+NH4]+			Biosynthesis of other secondary	
pos	297.0896	[M+H]+	5'-deoxyribon	5'-deoxy-5	Chemical structure t	2457-80-9
pos	540.4079	[M-NH3+H]+				
pos	291.1834	[M+NH4]+	Tetralins			47141-42-4
neg	421.2617	[M-H]-	Naphthopyrans		Biosynthesis of othe	133613-76
neg	168.0899	[M+HCO3]-	Pyridines and	Pyridoxam	Metabolism of cofac	85-87-0
pos	278.163	[M+H]+	Carboxylic ac	Amino acids, peptides, and analogues		
neg	567.3771	[M+HCOO]-			Global and overview	81661-90-7
pos	383.1667	[M-NH3+H]+	Benzothiazepi	Dibenzothiazepines		111974-69
neg	238.0689	[M-NH3-H]-	Organooxyge	Carbohydr	Glycan biosynthesis	10149-14-
pos	136.0385	[M+H]+	Imidazopyrim	Purines and	Global and overview	68-94-0
neg	394.1416	[M-NH3-H]-	Isoflavonoids	Rotenoids	Chemical structure t	83-79-4
pos	203.0946	[M+H]+	Indoles and de	Indolyl carboxylic acids and der		5548-09-4
pos	893.1469	[M+H]+	Prenol lipids	Monoterpe	Amino acid metabol	138149-18
pos	285.1365	[M+K]+			Chemical structure t	486-39-5
neg	437.293	[M-NH3-H]-			Biosynthesis of othe	167427-23-
neg	130.0266	[M-H]-	Fatty Acyls	Fatty acids	Carbohydrate metab	498-23-7
neg	184.0524	[M+HCO3]-			Xenobiotics biodegr	262-12-4
pos	465.0797	[M+H]+	Organooxyge	Carbohydrates and carbohydrate		33049-17-
neg	122.0368	[M-H]-	Benzene and s	Benzoic acids and derivatives		65-85-0
pos	140.0837	[M-OH+H]+	Organooxyge	Carbonyl compounds		42348-12-9
pos	290.2246	[M-NH3+H]+	Steroids and s	Androstane	Lipid metabolism; C	521-18-6
pos	801.6611	[M-CO2+H]+	Glycerophosp	Glycerophosphocholines		
pos	294.1732	[2M+H]+			Chemical structure transformati	
pos	516.2934	[M-NH3+H]+			Global and overview maps; Met	
pos	580.1792	[M+K]+	Flavonoids	Flavonoid	Biosynthesis of othe	10236-47-2
pos	336.2301	[M+H]+	Fatty Acyls	Eicosanoids		
pos	183.0565	[M+H]+	Organic sulfu	Sulfuric ac	Membrane transport	4858-96-2
pos	222.1984	[M+NH4]+	Prenol lipids	Sesquiterpenoids		15051-81-7
neg	334.0665	[M-H]-				
neg	203.0582	[M-H]-	Indoles and de	Indolyl car	Amino acid metabol	392-12-1
pos	544.34	[M+H]+	Prenol lipids	Terpene lactones		171499-81
pos	467.3012	[M+H]+	Glycerophosp	Glycerophosphoethanolamines		
pos	300.2089	[M-OH+H]+	Prenol lipids	Diterpenoids		90044-20-3
neg	461.3352	[M+HCO3]-	Sphingolipids	Glycosphi	Lipid metabolism	2238-90-6
pos	230.0192	[M+H]+	Organic phosp	Phosphate esters		
pos	103.0633	[M+H]+	Carboxylic ac	Amino acids, peptides, and anal		167222-96
pos	159.0532	[M+Na]+	Carboxylic ac	Amino acids, peptides, and anal		99319-03-(
neg	500.3071	[M-H]-	Carboxylic ac	Amino acids, peptides, and anal		112592-90
pos	422.0825	[M+H]+			Carbohydrate metab	4549-10-4

pos	296.1776	[M-CO2+H]+			Biosynthesis of othe	502-70-5
pos	217.1314	[M+H]+				17298-37-2
pos	237.0862	[M-OH+H]+	Pteridines and	Pterins and	Metabolism of cofac	89687-39-8
pos	827.5465	[M-CO2+H]+	Glycerophosp	Glycerophosphocholines		
pos	441.3454	[M-H2O+H]+	Fatty Acyls	Fatty acid esters		
pos	562.4386	[M-OH+H]+	Prenol lipids	Polyprenylphenols		
pos	271.0845	[M+NH4]+				109437-82-
pos	152.0685	[M+H]+	Organooxyger	Carbohydr	Carbohydrate metab	488-82-4
neg	408.2876	[M+HCOO]-	Steroids and s	Bile acids,	Lipid metabolism; G	81-25-4
pos	244.2038	[M+K]+	Fatty Acyls	Fatty acids and conjugates		3422-31-9
neg	278.0725	[M+HCO3]-	Carboxylic ac	Amino acids, peptides, and anal		121514-30
neg	184.1099	[M-H]-			Metabolism of terpenoids and p	
pos	119.0735	[M+H]+	Indoles and de	Indolines		496-15-1
neg	204.0899	[M-H]-	Indoles and de	Indolyl car	Cancer: overview; N	73-22-3
pos	594.1373	[M+K]+	Flavonoids	Flavonoid glycosides		2173206-3
neg	187.0625	[M+HCOO]-	Triazines	Aminotria	Xenobiotics biodegr	6190-65-4
neg	346.1416	[M-H2O-H]-	Prenol lipids	Terpene la	Chemical structure t	11017-56-4
pos	592.1792	[M+H]+				91095-79-3
neg	282.2559	[2M-H]-				693-72-1
pos	130.063	[M+NH4]+	Lactones	Gamma butyrolactones		599-04-2
neg	548.3515	[M-H]-	Harmala alkaloids			
neg	220.1059	[2M-H]-	Organooxyger	Carbohydrates and carbohydrate		112339-01
neg	602.3584	[M-NH3-H]-				
pos	453.2855	[M+H]+	Glycerophosp	Glycerophosphoethanolamines		53862-35-4
neg	344.1862	[M-H]-				18446-73-6
pos	395.0358	[M+NH4]+	Lactams	Beta lactams		91832-40-5
neg	302.1882	[M-H]-			Global and overview	1372138-8
pos	449.2849	[M-H2O+H]+			Biosynthesis of othe	26098-04-4
neg	266.1518	[M-H]-				
pos	366.0577	[M+K]+	Imidazole ribo	1-ribosyl-i	Chemical structure t	13018-54-7
pos	406.2719	[M-H2O+H]+	Steroids and s	Bile acids, alcohols and derivati		911-40-0
neg	166.0477	[M-H2O-H]-			Carbohydrate metab	608-53-7
neg	200.0296	[M+HCOO]-				1013-23-6
neg	150.0528	[2M-H]-				7296-56-2
pos	412.2977	[M-H2O+H]+	Steroids and s	Vitamin D and derivatives		112828-00
neg	288.0634	[M+HCOO]-	Flavonoids	Flavans	Biosynthesis of othe	4049-38-1
pos	448.3552	[M-H2O+H]+	Steroids and s	Bile acids,	Global and overview	92751-21-8
neg	652.2731	[M-NH3-H]-	Prenol lipids	Diterpenoi	Biosynthesis of othe	57710-64-2
pos	242.0096	[M+Na]+				
pos	200.0408	[M+H]+	Indoles and de	Indoles		135531-86
pos	117.079	[M+H]+	Carboxylic ac	Amino acids, peptides, and anal		107-43-7
pos	473.1659	[M+H]+	Pteridines and	Pterins and	Metabolism of cofac	2800-34-2
pos	586.2791	[M-OH+H]+			Metabolism of cofac	20298-86-6
neg	128.1201	[M+HCOO]-	Organooxyger	Carbonyl compounds		27457-18-7
pos	162.0528	[M-OH+H]+			Carbohydrate metabolism; Glob	
pos	268.0888	[2M+H]+	Phenanthrene	Chrysenes	Xenobiotics biodegr	72010-13-0
pos	666.2219	[M+H]+				
pos	326.2457	[M+H]+	Fatty Acyls	Fatty alcohols		25346-18-3

pos	354.2406	[M-OH+H]+	Fatty Acyls	Eicosanoids		745-65-3
pos	87.03203	[M+Na]+	Azolines	Oxazolines		51667-26-
pos	451.2722	[M+H]+			Biosynthesis of other secondary	
neg	310.2144	[M-H]-	Fatty Acyls	Lineolic ac	Lipid metabolism; G	67597-26-
pos	376.1311	[M+K]+	Cinnamic aci	Hydroxycinnamic acids and derivatives		
pos	853.5621	[M+NH4]+	Glycerophosp	Glycerophosphocholines		
pos	126.0317	[M+H]+	Organooxyge	Carbonyl c	Xenobiotics biodegr	67-47-0
pos	586.3506	[M-OH+H]+			Metabolism of terpenoids and p	
pos	306.074	[M+H]+			Biosynthesis of othe	1617-55-6
neg	300.2664	[M-H]-				1330-70-7
pos	75.06841	[2M+H]+			Amino acid metabol	2799-16-8
pos	438.0941	[M+H]+	Pteridines and	Alloxazines and isoalloxazines		
pos	765.5308	[M+NH4]+	Glycerophosp	Glycerophosphocholines		
pos	372.2876	[M+NH4]+	Glycerolipids	Diradylglycerols		
pos	122.0368	[M+NH4]+	Organooxyge	Carbonyl c	Chemical structure t	90-02-8
pos	430.3083	[M+H]+	Steroids and s	Vitamin D and derivatives		
neg	188.0321	[M+HCOO]-				13366-20-
neg	592.4518	[M-H2O-H]-	Prenol lipids	Sesquiterpenoids		173449-96
pos	382.3811	[M-OH+H]+	Fatty Acyls	Fatty alcohols		
neg	428.1471	[M-H]-				23978-65-
neg	428.1682	[M-H]-				499-33-2
neg	234.074	[M-H2O-H]-	Carboxylic ac	Tricarboxylic acids and derivati		83966-24-
neg	356.2563	[M-H]-	Fatty Acyls	Eicosanoids		745-62-0
pos	849.1571	[M+Na]+			Amino acid metabol	6712-03-4
pos	159.0644	[M+H]+	Carboxylic ac	Amino acids, peptides, and anal		21539-44-
neg	216.0569	[M-H]-	Carboxylic ac	Amino aci	Metabolism of cofac	16968-98-
pos	203.1344	[M+NH4]+			Biosynthesis of other secondary	
pos	338.2457	[M-CO2+H]+			Global and overview	38966-21-1
pos	495.2904	[M-H2O+H]+			Biosynthesis of othe	51846-98-1
pos	161.0688	[M-H2O+H]+	Carboxylic ac	Amino aci	Amino acid metabol	1118-90-7
pos	153.079	[M-H2O+H]+	Phenols	Benzenedi	Nervous system; Sig	62-31-7
pos	481.3168	[M+NH4]+	Glycerophosp	Glycerophosphocholines		
neg	164.0685	[M+HCOO]-				40026-07-1
neg	293.0834	[M+HCO3]-			Amino acid metabolism; Biosyn	
pos	346.178	[M+K]+	Prenol lipids	Terpene lactones		
neg	314.0651	[M-H]-	Azolidines	Imidazolidines		1185234-9
neg	202.143	[M-H]-	Carboxylic ac	Amino acids, peptides, and anal		30315-93-
pos	911.1575	[M-OH+H]+	Fatty Acyls	Fatty acyl	Cell growth and dea	26926-09-
neg	226.0702	[M-NH3-H]-			Biosynthesis of other secondary	
pos	128.0473	[M-H2O+H]+			Xenobiotics biodegr	1883-75-6
neg	326.079	[M-NH3-H]-	Sterigmatocystins		Biosynthesis of othe	6795-16-0
pos	376.2766	[M+H]+			Global and overview maps; Me	
pos	300.0634	[M+NH4]+	Flavonoids	O-methylated flavonoids		1592-70-7
neg	200.0797	[M-H2O-H]-			Biosynthesis of other secondary	
pos	132.0059	[2M+H]+	Keto acids an	Short-chai	Cancer: overview; C	328-42-7
neg	384.1216	[M+HCOO]-	Lactones	Gamma bu	Amino acid metabol	979-92-0
neg	108.0211	[2M-H]-	Organooxyge	Carbonyl c	Xenobiotics biodegr	3225-29-4
pos	805.5621	[M+H]+	Glycerophosp	Glycerophosphocholines		

pos	290.261	[M-H2O+H]+				
pos	825.5308	[M-CO2+H]+	Glycerophosp	Glycerophosphocholines		
pos	268.0736	[2M+H]+	Isoflavonoids	O-methyla	Chemical structure t	485-72-3
neg	388.0073	[M-H]-	Pyrimidine nu	Pyrimidine	Global and overview	4208-67-7
pos	181.183	[M+H]+	Organonitroge	Cyclohexylamines		101-83-7
neg	189.0637	[M-H]-	Carboxylic ac	Amino aci	Amino acid metabol	1188-37-0
neg	275.0559	[2M-H]-	Benzene and s	Nitrobenze	Xenobiotics biodegr	311-45-5
neg	305.0413	[M+HCOO]-				3616-08-8
neg	328.0583	[M-NH3-H]-	Coumarins an	Furanocou	Biosynthesis of othe	6795-23-9
pos	286.1681	[M-CO2+H]+			Biosynthesis of othe	75917-16-7
neg	186.0681	[2M-H]-			Xenobiotics biodegr	1133-63-7
pos	114.0793	[M+H]+	Carboxylic ac	Amino acids, peptides, and anal		2812-47-7
pos	121.0528	[M+Na]+	Organooxyger	Carbonyl compounds		30440-88-1
pos	266.1267	[M-OH+H]+			Energy metabolism; Global and	
pos	400.3705	[M+H]+	Steroids and s	Ergostane	Lipid metabolism; C	474-62-4
pos	517.3168	[M+Na]+	Glycerophosp	Glycerophosphocholines		1199257-4
neg	215.0559	[M-H]-	Glycerophosp	Glyceroph	Lipid metabolism	1190-00-7
neg	373.1889	[M-NH3-H]-			Chemical structure t	23068-65-7
neg	482.2224	[M-H]-			Biosynthesis of other secondary	
pos	209.1263	[M+H]+	Organonitroge	Amines		
neg	419.1957	[M-NH3-H]-			Biosynthesis of other secondary	
pos	187.1208	[M-H2O+H]+	Fatty Acyls	Fatty acids	Metabolism of cofac	4707-58-8
pos	252.1586	[M+H]+	Organonitroge	Amines		51235-04-2
pos	327.2773	[M+K]+	Carboxylic ac	Amino acids, peptides, and analogues		
pos	545.3481	[M+Na]+	Glycerophosp	Glycerophosphocholines		1199257-4
pos	188.1273	[M+H]+	Carboxylic ac	Amino acids, peptides, and anal		156-86-5
pos	210.0892	[M+NH4]+				125074-06-
pos	921.7186	[M+Na]+	Glycerophosp	Glycerophosphocholines		
neg	241.0739	[M+HCO3]-	Quinolines an	Benzoquin	Chemical structure t	28333-02-0
pos	151.0494	[M+H]+	Imidazopyrim	Purines an	Global and overview	66224-64-4
neg	494.3607	[M-H]-	Steroids and s	Steroid lactones		80483-89-2
neg	334.2144	[M-H2O-H]-	Prenol lipids	Diterpenoi	Global and overview	26109-32-0
pos	275.1158	[M+H]+				22964-77-8
neg	342.2042	[M-H2O-H]-	Fatty Acyls	Eicosanoids		
pos	226.1933	[M-OH+H]+	Fatty Acyls	Fatty acids and conjugates		2430-95-7
pos	192.0786	[M+H]+	Cinnamic acic	Hydroxycinnamic acids and der		22214-42-2
pos	122.048	[M+H]+	Pyridines and	Pyridinecarboxylic acids and de		1453-82-3
pos	268.0736	[M+K]+			Chemical structure t	486-63-5
pos	567.3325	[M+Na]+	Glycerophosp	Glycerophosphocholines		162440-05
pos	865.152	[M-NH3+H]+	Fatty Acyls	Fatty acyl	Amino acid metabol	6712-01-2
neg	338.1994	[M-H]-	Benzene and s	Anilides		81329-70-0
neg	228.0746	[M+HCO3]-	Pyrimidine nu	Pyrimidine	Membrane transport	951-78-0
neg	239.0728	[M-H]-	Benzimidazoles			80983-34-2
neg	228.0786	[2M-H]-	Stilbenes		Chemical structure t	31100-06-8

HMDB	Metabolite name	Fold change
	**Double-HW vs CTRL**	
HMDB0304474	Tetramethylpyrazine	0.01
)	Indolepyruvate	17.03
	Quercetin 3-galactoside 7-rhamnoside	52.24
HMDB0000630	CoA 8_0;O	13.35
metabolites; Global and overviev	7Z-tetradecenoic acid	20.83
metabolites; Global and overviev	8-Demethyl-8-alpha-L-rhamnosyltetracenomycin C	25.80
HMDB0035514	PC(20_4(8Z,11Z,14Z,17Z)_22_6(4Z,7Z,10Z,13Z,16Z,19Z))	35.46
4	trans-Methylbixin	8.67
	D-2-Amino-hexano-6-lactam	22.67
HMDB0030704	Phosphoribosyl formamidocarboxamide	88.56
HMDB0001367	6'-O-p-Coumaroyltrifolin	13.50
olyketides	2-Methylacetoacetyl-CoA	12.09
HMDB0011106	Rotenone	0.03
5	Estriol	13.50
metabolites; Global and overviev	Glyceryl lactooleate	59.94
HMDB0000481	Carisoprodol	43.00
HMDB0001296	Ribonic acid	5.27
HMDB0000601	3-Epiecdysone	82.72
HMDB0005998	1,2,3,4,6-Pentagalloyl_glucose	23.63
	PC-M6	18.05
HMDB0061086	Neoporrigenin B	14.77
HMDB0060758	pyrethrin I	59.28
HMDB0004482	Diosgenin	21.61
HMDB0014760	L-Ornithine	0.23
HMDB0001190	(Z)-S-1-Propenyl methanesulfinothioate	0.17
metabolites; Global and overviev	Kanzonol K	89.84
HMDB0004702	ST 21_2;O2	7.95
HMDB0039265	5-Carboxy-2-pentenoyl-CoA	99.54
HMDB0034272	Rishitin	242.16
HMDB0000773	Gibberellin A3	0.14
HMDB0000299	25-Hydroxyvitamin D3-26,23-lactol	16.86
	Tylactone	22.27
HMDB0002032	Undecaprenyl diphosphate	182.78
HMDB0008470	LysoPC(15_0_0_0)	43.89
-9	Citicoline	11.43
HMDB0001020	cis-p-Coumaric acid 4-[apiosyl-(1->2)-glucoside]	160.39
HMDB0041069	Cosmosiin	6.41
HMDB0000842	Levobunolol	4.83
HMDB0029365	Cytosine	1.50
HMDB0000730	S-(-)-ureidoglycolate	2.93
HMDB0060624	L-Histidine	0.29
HMDB0002204	PC(MonoMe(11,5)_MonoMe(13,5))	514.56
HMDB0015114	Hexazinone	16.15
HMDB0013926	Tetrahydropalmatine	139.04
metabolites; Global and overviev	5,10-dihydrophenazine	48.51

HMDB0304351	Naringenin	0.17
HMDB0000177	20-Hydroxyeicosatetraenoic acid	9.10
HMDB0304066	10-Apo-beta-carotenal	16.88
HMDB0304522	Formononetin	48.48
4	4-Anilino-4-oxobutanoic acid	0.21
HMDB0000251	N-Methyl-4-dimethylallyltryptophan	53.18
olyketides	PC(P-16_0_22_0)	9.04
HMDB0038340	PE-NMe2(18_1(9Z)_18_3(9Z,12Z,15Z))	6.24
HMDB0001218	CAR 3_0	4.91
HMDB0038628	DIMBOA-Glc	30.15
7	Astaxanthin	9.65
HMDB00086	L-Glutamic acid	0.29
HMDB0032747	L-Glutamine	0.31
HMDB0006468	Coumarin	157.09
HMDB0000684	Paxilline	22.30
HMDB0010217	Megalomicin C1	39.08
HMDB0001480	2-((N-(1-(1H-Imidazol-4-yl)-2-propyl)imino)phenylmethyl)	0.19
metabolites; Global and overvie	Dihydromethanophenazine	52.59
HMDB0039545	3-Methylcrotonyl-CoA	5.50
HMDB0014704	gamma-Glutamylcysteine	3.76
3	beta-Geraniol	0.19
	6-Hydroxyprotopine	25.69
HMDB0000158	Taurine	6.98
3	Styrene Oxide	0.61
	3,5-Dibromo-4-hydroxybenzoate	4.64
HMDB0029898	Diadinoxanthin	41.01
HMDB0035541	Protoporphyrinogen IX	5.43
HMDB0004080	Allantoic acid	4.57
5	miconazole	24.70
HMDB0060267	Trigonelline (N'-methylnicotinate)	0.03
HMDB0002117	Methoxamine	1.57
HMDB0036584	Nicardipine	9.62
HMDB0041953	Argininosuccinic acid disodium	18.13
HMDB0062390	PC(P-16_0_18_2)	12.97
HMDB0251441	delta3,5-Deoxytigogenin	9.62
	L-Olivosyl-oleandolide	0.11
metabolites; Global and overvie	L,L-Cyclo(leucylprolyl)	78.09
HMDB0001011	S-(2-Methylpropionyl)-dihydrolipoamide-E	10.09
5	N-Fructosyl isoleucine	3.69
HMDB0003154	Linoleamide	4.03
HMDB0029355	Neuro_000161	5.59
HMDB0001058	Tembetarine	5.22
9	Camalexin	0.85
HMDB0011211	Glucohesperalin	6.28
HMDB0006868	Coprocholic acid	4.09
HMDB0252340	6-Deoxocastasterone	87.58
HMDB0037088	Prostaglandin C1	0.09
-7	xi-2,3-Dihydro-2-oxo-1H-indole-3-acetic acid	8.59

HMDB0041212	Androstanediol-17g	0.19
	Dibenzothiophene	6.41
HMDB0000867	L-Isoleucine	0.21
HMDB0030003	Tryprostatin B	0.20
HMDB0000148	Cymarin	24.96
abolism of terpenoids and polyke	Acetyl-N-formyl-5-methoxykynurenamine	28.75
HMDB0010203	PC(14_1(9Z)_P-18_1(11Z))	587.21
HMDB0000156	5,15-DiHETE	3.14
3	Methacrylyl-CoA	4.08
HMDB0032782	3-Hydroxy-3-methylglutaryl-CoA	40.25
HMDB0003952	aphidicolin	7.06
HMDB0248414	PC(24_0_22_4(7Z,10Z,13Z,16Z))	9.29
HMDB0000214	2-Hexaprenyl-6-methoxy-1,4-benzoquinone	11.81
abolism of terpenoids and polyke	N1-Amidinostreptamine 6-phosphate	5.22
HMDB0008138	Coclaurine	16.89
2	28-Homobrassinolide	41.65
3	8-Hydroxy-2-methoxy-6-methyl-1,4-naphthoquinone	31.57
HMDB0031057	Scymnol	6.09
HMDB0000493	Chlorobactene	5.94
	LysoPE(18_1(9Z)_0_0)	54.15
HMDB0000641	7-Ketodeoxycholic acid	29.77
HMDB0001321	PC(20_4(8Z,11Z,14Z,17Z)_18_4(6Z,9Z,12Z,15Z))	11.39
HMDB0001209	3-Aminobutanoic acid	0.26
HMDB0062656	SCHEMBL16620138	0.86
HMDB0014814	dolichyl beta-D-glucosyl phosphate	7.52
HMDB0033153	PC(18_2(9Z,12Z)_16_0)	4.03
-7	dTDP-4-oxo-2-deoxy-beta-L-xylose	28.41
HMDB0030323	1-Archaetidyl-1D-myo-inositol 3-phosphate; 1-Saturated arc	13.99
and overview maps	Trioxilin A3	7.60
HMDB0001049	3-Hydroxy-2H-pyran-2-one	1.79
HMDB0002038	2'-Deoxyuridine 5'-monophosphate disodium salt	8.23
HMDB0062765	7-alpha-hydroxy-3-oxo-4-cholestenoate (7-HOCA)	14.68
)	10-Hydroxydihydrosanguinarine	20.55
HMDB0031244	Canthaxanthin	16.15
HMDB0002259	N-Nonanoylglycine	21.92
HMDB0037538	1-(11Z-eicosenoyl)-glycero-3-phosphate	32.80
7	5-KETE	8.99
	Dihydroisopentenyldehydrorhodopin	15.90
7	PC(20_3(8Z,11Z,14Z)_20_5(5Z,8Z,11Z,14Z,17Z))	7.30
HMDB0250981	Dehydrogenated ticlopidine	3.54
HMDB0035154	Usnic acid	7.91
HMDB0012189	3-acetyloctanal	6.95
	Glyceric acid 1,3-biphosphate	25.40
HMDB0033752	1-pentadecanoyl-glycero-3-phosphate	51.50
HMDB0029377	Germacrene A acid	10.65
HMDB0114046	fecosterol	112.60
HMDB0012958	xi-5-Dodecanolide	0.23
metabolites	3-(4-methylthio)butylmalate	3.28

HMDB0000875	Cyclopentolate	3.17
HMDB0001413	Phycocyanobilin	8.97
HMDB0014539	Asymmetric dimethylarginine	0.35
HMDB0000757	Flunixin	7.14
HMDB0030095	Taurocholate	10.12
HMDB0029736	Orientin	29.80
HMDB0245646	SAICAR	4.13
HMDB0248306	LysoPC(18_3(9Z,12Z,15Z)_0_0)	8.30
HMDB0001554	3-(all-trans-octaprenyl)benzene-1,2-diol	16.10
HMDB0007973	gamma-Tocotrienol	0.29
HMDB0011753	D-Erythrose 4-phosphate	6.54
HMDB0001060	Xanthosine	2.16
HMDB0003331	LPC 18_2	51.53
HMDB0035155	gamma-Glutamyl-gamma-aminobutyraldehyde	14.45
-8	L-Thyronine	0.27
HMDB0039780	3-Ketosucrose	12.56
HMDB0000656	Allopurinol riboside	9.69
HMDB0248231	Goltix	0.34
7	1,5-Anhydrosorbitol	2.28
HMDB0004259	PC(22_6(4Z,7Z,10Z,13Z,16Z,19Z)_18_4(6Z,9Z,12Z,15Z))	14.24
HMDB0028686	roquefortine D	17.28
HMDB0001270	Manzamine A	13.15
HMDB0304001	3-Amino-2-azepanone	0.26
HMDB0254501	Inulobiose	0.21
HMDB0136090	Ubiquinol-10	35.56
	Galactinol	32.62
2	Ginsenoside F2	4.21
HMDB0003559	14alpha-Hydroxymethylsteroid	0.94
HMDB0001546	N-(3-Methylbut-2-EN-1-YL)-9H-purin-6-amine	3.34
HMDB0029378	Bilastine	5.56
HMDB0039078	Phenol sulphate	1.08
HMDB0000172	Calcipotriol	5.20
HMDB0249356	Deoxymyxol	31.43
HMDB0000707	10-Formyltetrahydrofolate	18.96
HMDB0029838	trihomomethionine	3.17
HMDB0008412	3-Hexen-1-ol	0.38
HMDB0001977	N-Desmethylvenlafaxine	5.55
HMDB0030769	N-Benzoyl-4-methoxyanthranilate	4.32
5	4-Hydroxysphinganine	0.28
	Oryzalexin C	3.73
HMDB0006482	Pyroglutamic acid	6.03
	Xenognosin B	6.25
HMDB0060095	FA 18_4;O	9.15
HMDB0004193	Ciceritol	38.18
	Dantrolene	0.12
HMDB0000133	Aflatoxin M1	10.51
HMDB0015543	Galactosylsphingosine	11.31
HMDB0000153	Thellungianin G	1.86

-3		3-Ethyl-2-hydroxy-4-methyl-2-cyclopenten-1-one	1.63
HMDB0032498		Aminopropylcadaverine	3.79
		delta-Valerolactone	1.91
HMDB0032994		Quinaldic acid	1.97
-8		Aloin	29.61
HMDB0040443		omega-Hydroxyphylloquinone	15.06
		Ciliatocholic acid	12.39
HMDB0304944		2-Isopropyl citrate	0.18
metabolites; Global and overview		Leprotin	20.78
HMDB0001173		dTDP-4-oxo-6-deoxy-D-glucose	89.24
		2-((2E)-3,7-Dimethyl-2,6-octadienyl)-5,6-dimethoxy-3-meth	2.47
HMDB0015341		4-[(2,4-Dihydroxy-3,3-dimethylbutanoyl)amino]butanoic aci	5.96
HMDB0038568		Cyclooctat-9-ene-5,7-diol	23.53
HMDB0001431		1-Carboxyethylleucine	2.58
HMDB0253025		Dicyclohexylamine	2.74
7		6-Chloro-N-(1-methylethyl)-1,3,5-triazine-2,4-diamine	0.28
HMDB0005021		L-Arabinonate	0.58
HMDB0304125		Tuftsin	0.25
HMDB0000157		6,7-Dimethyl-8-(1-D-ribityl)lumazine	8.14
HMDB0034436		4,4-Diaponeurosporene	26.78
HMDB0240623		UDP-N-acetylmuramoyl-L-alanyl-D-glutamate	4.03
HMDB0060392		24,25-Diacetylvulgaroside	3.93
		Lecithin	4.13
-8		Alkannin	2.50
HMDB0000634		Prephenate	5.78
		ST 28_0;O3	8.61
HMDB0038410		Bisnorbiotin	0.19
HMDB0001870		Cefdinir	10.01
HMDB0036176		Sinapyl alcohol	27.79
HMDB0002961		CMP-8-amino-3,8-dideoxy-beta-D-manno-octulosonate	6.79
HMDB0011223		6-Thioxanthine 5'-monophosphate	6.99
on maps		L-tetrahomomethionine	2.50
abolism of terpenoids and polyke		2-Octaprenyl-6-methoxyphenol	10.05
HMDB0002927		Phylloquinol	0.34
HMDB0062619		(ent-6alpha,7alpha)-6,7-Dihydroxy-16-kauren-19-oic acid	0.11
HMDB0250194		alpha-Curcumene	2.77
HMDB0303908		12,13-EpOME	2.85
		2-Hydroxybenzaldehyde	1.45
HMDB0060484		alpha,alpha-Trehalose 6-mycolate	6.06
HMDB0041027		Crocetindial	4.94
HMDB0061691		Trichocarposide	3.82
HMDB0302101		N-Acetyl-glucosamine 1-phosphate	4.31
HMDB0000648		Dibenzothiophene sulfone	7.56
HMDB0012195		2-Nonenal	0.86
HMDB0031654		Fe2+	2.47
HMDB0242583		alpha-Hydroxy-N-desmethyltamoxifen	10.97
HMDB0005770		Pseudoionone	3.80
		L-Fuculose	2.94

	Estrone glucuronide	28.96
2	Beta-Citryl-L-glutamic acid	0.45
HMDB0001195	7,8-Dihydro-7-hydroxy-8-S-glutathionyl-benzo[a]pyrene	11.39
HMDB0008254	Fexofenadine	0.28
HMDB0013339	8-Hydroxyguanine	3.17
HMDB0304230	N-Formyl-L-glutamic acid	1.77
-3	3-Deoxo-4b-deoxypaxilline	9.86
HMDB0000568	5-(Hydroxymethyl)-2-methylpyrimidin-4-OL	3.15
HMDB0000619	Oleamide	3.49
HMDB0061656	Paraoxon	15.09
HMDB0304217	20-Hydroxy-leukotriene B4	2.94
olyketides	PC(MonoMe(9,5)_MonoMe(9,5))	1.64
HMDB0253472	Flavonol base + 4O, O-Hex-dHex-Pen	3.94
HMDB0000929	D-Fructuronic acid	2.01
HMDB0040689	cis,cis-3,6-Dodecadienoyl-CoA	15.54
HMDB0033249	(+)-Dihydrocarveol	3.07
HMDB0036677	Homo-L-arginine	0.50
3	4-Hydroxydebrisoquine	0.08
HMDB0240219	2-Oxokanamycin	39.13
HMDB0303902	5-pentyl-2-furannonanoic acid	0.40
HMDB0242555	PC(18_2_18_2)	7.53
HMDB0001104	3-O-Methylkaempferol	37.83
	beta-Citraurin	1.31
HMDB0011503	17alpha,21-Dihydroxypregnenolone	0.51
5	corynantheal	2.33
HMDB0014675	Tauro-α-muricholic acid	3.90
6-7	eugeniin	24.63
4	Citraconic acid	3.04
	2-(2,4-Dichloro-phenoxy)-N-(2-mercapto-ethyl)-acetamide	1.76
HMDB0001439	Benzo[a]pyrene-4,5-oxide	5.46
HMDB0000391	Vitamin A2	4.33
	4α-hydroxymethyl-4β-methyl-5α-cholesta-8,24-dien-3β-ol	4.17
	2-Hydroxy-2-[2-oxo-2-[[4-[(3-aminopropyl)amino]butyl]am	10.29
HMDB0000646	Avermectin B2b aglycone	6.10
HMDB0015567	(S)-2-Methylbutanal	0.52
HMDB0005810	Maritinamine	0.50
HMDB0034423	2-(2-Furyl)-3-(5-nitro-2-furyl)acrylamide	0.11
HMDB0002353	L-Allothreonine	0.60
	Fumiquinazoline C	6.80
HMDB0038631	sn-glycero-3-Phosphoethanolamine	0.27
HMDB0000043	2-Deoxycastasterone	3.98
HMDB0000972	1,3,7-trimethyl-5-hydroxyisourate	2.09
5	Perfluorooctanesulfonate (PFOS)	20.78
HMDB0001140	L-Arabinose	8.48
al and overview maps	L-Kynurenine	3.79
HMDB0060091	PC(16_0_18_1(9Z)-O(12,13))	11.24
	Nnal-N-oxide	0.30
HMDB0031006	3-Sulfinylpyruvic acid	0.73

HMDB0001442		THC 4-glucoside	2.93
HMDB0245282		3-hydroxybenzyl alcohol	0.54
metabolites; Global and overviev		Isobutyrylglycine	2.27
HMDB0301803		Fructose 1,6-bisphosphate	0.83
HMDB0033932		Deoxylimonate	0.40
HMDB0008485		Uridine 5'-monophosphate	0.72
HMDB0034355		Vincristine	248.40
olyketides		(S)-scoulerine	0.50
HMDB0038365		pentahomomethionine	2.61
		Tetradecanoylcarnitine	5.53
		Cinnatriacetin A	5.93
HMDB0059614		2-methyl-6-geranylgeranyl-1,4-benzoquinol	2.76
HMDB0007951		dTDP-4-amino-2,3,4,6-tetradeoxy-D-glucose	13.95
HMDB0092960		4-Aminobutanoyl-CoA	3.69
HMDB0034170		Cinncassiol D1 glucoside	11.15
HMDB0060127		Hapten	17.82
5		Glucoiberverin	0.46
HMDB0060196		Naringin	10.90
HMDB0035629		3-Hydroxy-11Z-octadecenoylcarnitine	8.68
5		Stachydrine	0.72
		S-adenosylhomocysteine (SAH)	6.11
HMDB0038083		LysoPC(20_1(11Z)_0_0)	5.41
HMDB0002685		Pantothenic acid	1.67
HMDB0001493		5-Ethoxysorgoleone 358	5.94
HMDB0030402		Hyperforin	4.81
HMDB0004821		dihydro-3-hydroxy-4,4-dimethyl- 2(3H)-Furanone	0.63
metabolites; Global and overviev		6-methoxy-3-methyl-2-all-trans-hexaprenyl-1,4-benzoquinol	3.79
l		8,8a-Deoxyoleandolide	4.62
l		Isopropanolamine	0.48
HMDB0000510		SCHEMBL21051772	1.89
HMDB0000073		Ethyl hydrogen sulfate	1.48
HMDB0010381		Nocardicin E	0.14
l		2-(beta-D-Glucosyl)-sn-glycerol 3-phosphate	0.38
ıthesis of other secondary metabol		(1S,5R)-5-Hydroxyaverantin	3.74
HMDB0034605		(E)-4-Isothiocyanato-1-(methylthio)-1-butene	0.16
HMDB0015350		Anandamide	9.57
HMDB0001539		Urdamycinone B	9.04
HMDB0001375		4-O-Methylnorbelladine	19.85
metabolites; Global and overviev		Dihydrosterigmatocystin	6.23
		Ubiquinone-1	0.50
HMDB0030590		erythromycin E	4.44
HMDB0036887		Dolichol phosphate	0.48
HMDB0302564		Verapamil	11.97
metabolites; Global and overviev		(3S,5S)-Carbapenam-3-carboxylate	0.24
HMDB0000223		beta-Glycerophosphoric acid	2.05
HMDB0000939		Spirotaccagenin	14.81
HMDB0003364		Sanguinarine	0.59
HMDB0008498		SS-secoisolariciresinol	0.28

		N-Retinylidene-N-retinylethanolamine	17.24
HMDB0008733		Prostaglandin E1	2.22
HMDB0005808		ent-Gallocatechin 3-gallate	3.10
HMDB0001000		Testololactone	1.53
HMDB0251214		Prostaglandin-c2	0.83
HMDB0001138		Dihydrosanguinarine	2.56
HMDB0013035		1,3-alpha-D-Mannosyl-1,2-alpha-D-mannosyl-1,2-alpha-D-r	8.32
		L-Homoserine	2.12
HMDB0030479		Levan	3.99
7		Guanine	1.89
		Myxoxanthophyll	2.00
HMDB0253910		7,8-Dihydro-beta-carotene	4.65
HMDB0035281		Avermectin A1b aglycone	0.34
overview maps		Methyl indole-3-acetate	0.35
HMDB0002869		epi-aristolochene	0.34
HMDB0010387		2-Methoxyestrone 3-glucuronide	6.79
HMDB0059660		Isohyodeoxycholic acid	2.07
7		(S)-Methylmalonic acid semialdehyde	1.42
metabolites; Global and overview		PE 40_4	18.80
HMDB0249241		N-Ribosylhistidine	1.19
metabolites; Global and overview		Glycogen	0.69
HMDB0240687		APC	2.79
HMDB0253153		2,5-Dichlorophenol	1.61
HMDB0013246		Dehydroxypaxilline	35.58
HMDB0010393		3-Dehydrosphinganine	6.86
HMDB0000670		1H-Imidazole-1-acetic acid	20.99
-8		Erythritol	1.49
HMDB0008778		Macarpine	0.37
HMDB0304008		4a-Hydroxytetrahydrobiopterin	0.45
HMDB0000132		(+)-O-methylkolavelool	0.60
HMDB0030423		OA-6129 B2	0.33
HMDB0036763		N6,N6,N6-Trimethyl-L-lysine	1.42
3		beta-Apo-4-carotenal	4.19
HMDB0256371		Nuatigenin	8.84
HMDB0062243		R.g.-Keto I	9.34
HMDB0032591		26-hydroxybrassinolide	4.42
HMDB0253663		6-Hydroxyparmomomycin	3.34
HMDB0033994		PC(18_4(6Z,9Z,12Z,15Z)_22_5(7Z,10Z,13Z,16Z,19Z))	3.04
HMDB0010404		(5Z,9E,14Z)-(8xi,11R,12S)-11,12-epoxy-8-hydroxyicosa-5,9	0.48
HMDB0001157		Neurosporaxanthin	27.67
HMDB0255848		3-(2-hydroxyphenyl)propionate	1.60
HMDB0000012		5-hydroxyindole thiazolidine carboxylate	0.47
HMDB0247482		Roquefortine F	0.40
HMDB0003747		Pivmecillinam	0.23
		13-L-Hydroperoxylinoleic acid	0.34
		Indole-3-carboxylic acid	0.18
		Multifidol	0.62
		D-4-Hydroxy-2-oxoglutarate	1.44

	Butirosin B	12.50
	threo-(Homo)2-isocitrate	3.02
	TR 1 toxin	22.04
	D-Apiose	1.99
	LysoPC(P-18_0_0_0)	2.23
	Phosphodimethylethanolamine	0.67
	LysoPE(0_0_20_4(8Z,11Z,14Z,17Z))	21.64
	Didehydroagroclavine	3.14
	ST 19_1;O2	0.23
	LysoPC(22_6_0_0)	7.02
	Deoxyuridine triphosphate	16.29
	Isonicotinamide	1.42
	PC(18_1(9Z)_18_0)	0.73
	gamma-Glutamylarginine	0.24
	6-Hydroxyflavanone	2.58
	beta-Damascenone	0.22
	10S-HpOME	16.92
	Mycocyclosin	0.68
	Adenine	0.42
	L-Aspartic acid	0.61
	N-Acetyl-b-glucosaminylamine	0.59
	Glutaminylcysteine	0.32
	6-Hydroxy-8-pentacosanone	0.42
	2'-Deoxysepiapterin	0.90
	L-Norleucine	0.62
	Legumelin	7.50
	erythro-6,8-Triacontanediol	5.83
	Glutathione	0.57
	2-Acetylpyridine	0.36
	13-HOTE	1.88
	Sinapine	4.37
	Dimethyldisulfide	2.00
	Glutamylhistidine	14.36
	Riboflavin cyclic-4',5'-phosphate	1.49
	D-Ribose 5-phosphate	8.36
	beta-Peltatin A methyl ether	3.41
	Tiagabine	27.93
	Deoxyuridine	2.45
	Hexylamine	1.40
	5-epi-Lividomycin B	12.93
	Floramultine	3.42
	alpha-Ketoisovaleric acid	4.68
	Caldariellaquinone	4.04
	LysoPC(14_0_0_0)	29.96
	Curcumanolide A	0.62
	Castasterone	6.16
	Anthranilyl-CoA	1.52
	Indoline	0.50

	Glutathionylspermine	13.25
	Thialysine ketimine	2.87
	Dihydrobisanhydrobacterioruberin	2.99
	Demethylphylloquinone	0.45
	Melezitose	2.12
	8-demethyl-8-(2,3,4-O-trimethyl-alpha-L-rhamnosyl)tetracer	2.62
	Benzimidazole	6.41
	2,4-DICHLOROTOLUENE	1.29
	10-epi-gamma-eudesmol	3.07
	6-Chlorohydroxyquinol	4.47
	Avermectin A2a aglycone	2.12
	8-Amino-7-oxononanoic acid	6.47
	Harmol	2.03
	Phosphoenol-4-deoxy-3-tetrulosonate	0.47
	UDP-N-acetylglucosamine enolpyruvate	7.61
	PC(16_0_16_0)	5.61

P value	FDR	Ion mode	Exact mass	Precursor type	Class	Subclass	KEGG Pathway
< 0.0001	0.0002	pos	136.10004	[M+H]+	Diazines	Pyrazines	
< 0.0001	0.0026	neg	203.05824	[M-H]-	Indoles an	Indolyl car	Amino acid metab
< 0.0001	0.0006	pos	610.15338	[M+NH4]+	Flavonoids	Flavonoid glycosides	
< 0.0001	0.0008	pos	909.21458	[M+NH4]+			Lipid metabolism;
< 0.0001	0.0009	pos	226.19327	[M-OH+H]+	Fatty Acyl	Fatty acids and conjugates	
< 0.0001	0.0009	pos	604.14281	[M+K]+			Global and overvie
< 0.0001	0.0010	pos	853.56213	[M+NH4]+	Glyceroph	Glycerophosphocholines	
< 0.0001	0.0051	neg	408.23005	[M+HCO3]-	Prenol lipi	Diterpenoids	
< 0.0001	0.0011	pos	128.09496	[M-NH3+H]+			
< 0.0001	0.0011	pos	366.05766	[M+K]+	Imidazole	1-ribosyl-i	Chemical structure
< 0.0001	0.0012	pos	594.13733	[M+K]+	Flavonoids	Flavonoid glycosides	
< 0.0001	0.0013	pos	865.15198	[M-NH3+H]+	Fatty Acyl	Fatty acyl	Amino acid metab
< 0.0001	0.0068	neg	394.14163	[M-NH3-H]-	Isoflavono	Rotenoids	Chemical structure
< 0.0001	0.0016	pos	288.17254	[M+H]+	Steroids ar	Estrane ste	Lipid metabolism;
< 0.0001	0.0016	pos	428.31377	[M+H]+	Fatty Acyl	Fatty acid esters	
< 0.0001	0.0016	pos	260.1736	[M+K]+	Carboxylic	Amino acids, peptides, and ar	
< 0.0001	0.0074	neg	166.04774	[M-H]-	Organooxy	Carbohydrates and carbohydr	
< 0.0001	0.0017	pos	464.31377	[M-CO2+H]+			Metabolism of terp
< 0.0001	0.0017	pos	940.11818	[M+Na]+			Chemical structure
< 0.0001	0.0074	neg	421.26168	[M-H]-	Naphthopyrans		Biosynthesis of ot
< 0.0001	0.0017	pos	446.30321	[M+NH4]+	Prenol lipi	Triterpenoids	
< 0.0001	0.0018	pos	328.20383	[M+K]+			
< 0.001	0.0019	pos	414.31338	[M-OH+H]+			Chemical structure
< 0.001	0.0020	pos	132.08987	[M+H]+	Carboxylic	Amino aci	Membrane transpo
< 0.001	0.0023	pos	136.00166	[M+NH4]+	Thiosulfinic acid esters		
< 0.001	0.0023	pos	436.18858	[M+H]+	Isoflavono	Isoflavans	
< 0.001	0.0023	pos	316.24022	[M+H]+			Lipid metabolism
< 0.001	0.0023	pos	893.1469	[M+H]+	Prenol lipi	Monoterpe	Amino acid metab
< 0.001	0.0097	neg	222.16197	[M+HCO3]-	Organooxy	Alcohols and polyols	
< 0.001	0.0105	neg	346.14163	[M-H]-	Prenol lipi	Diterpenoi	Global and overvie
< 0.001	0.0028	pos	430.30829	[M+H]+	Steroids ar	Vitamin D and derivatives	
< 0.001	0.0030	pos	394.27191	[M+H]+			Global and overvie
< 0.001	0.0030	pos	926.63179	[M+NH4]+	Prenol lipi	Polyprenol	Chemical structure
< 0.001	0.0031	pos	481.31682	[M+NH4]+	Glyceroph	Glycerophosphocholines	
< 0.001	0.0125	neg	488.10733	[M+HCO3]-			Lipid metabolism;
< 0.001	0.0033	pos	458.14242	[M+Na]+	Organooxy	Carbohydrates and carbohydr	
< 0.001	0.0033	pos	432.10564	[M+K]+	Flavonoids	Flavonoid	Biosynthesis of ot
< 0.001	0.0034	pos	291.18343	[M+NH4]+	Tetralins		
< 0.001	0.0034	pos	111.04326	[M+H]+	Diazines	Pyrimidine	Global and overvie
< 0.001	0.0034	pos	133.02493	[M-NH3+H]+			
< 0.001	0.0133	neg	155.06947	[M-H]-	Carboxylic	Amino aci	Cancer: overview;
< 0.001	0.0034	pos	922.65367	[M+Na]+	Glyceroph	Glycerophosphocholines	
< 0.001	0.0034	pos	252.15862	[M+H]+	Organonitr	Amines	
< 0.001	0.0036	pos	355.17835	[M+NH4]+			Biosynthesis of ot
< 0.001	0.0036	pos	182.08439	[M+K]+			Biosynthesis of ot

< 0.001	0.0037	pos	272.06847	[M-H2O+H]+			Chemical structure
< 0.001	0.0037	pos	320.23513	[M+H]+	Fatty Acyl	Fatty acids	Lipid metabolism;
< 0.001	0.0038	pos	376.2766	[M+H]+			Global and overvie
< 0.001	0.0040	pos	268.07356	[2M+H]+	Isoflavono	O-methyla	Chemical structure
< 0.001	0.0042	pos	193.07389	[M+H]+	Benzene a	Anilides	
< 0.001	0.0042	pos	286.16812	[M-CO2+H]+			Biosynthesis of otl
< 0.001	0.0042	pos	801.66111	[M-CO2+H]+	Glyceroph	Glycerophosphocholines	
< 0.001	0.0042	pos	767.54648	[M+H]+	Glyceroph	Glycerophosphoethanolamine	
< 0.001	0.0043	pos	217.1314	[M+H]+			
< 0.001	0.0043	pos	373.10089	[M+K]+	Organooxy	Carbohydr	Biosynthesis of otl
< 0.001	0.0043	pos	596.38654	[M+Na]+	Prenol lipi	Tetraterpe	Global and overvie
< 0.001	0.0045	pos	147.05316	[M+H]+	Carboxylic	Amino aci	Neurodegenerative
< 0.001	0.0045	pos	146.06914	[M+H]+	Carboxylic	Amino aci	Cancer: overview;
< 0.001	0.0047	pos	146.03678	[M-NH3+H]+	Coumarins and deriva		Biosynthesis of otl
< 0.001	0.0170	neg	435.24095	[M-H]-	Naphthopyrans		Biosynthesis of otl
< 0.001	0.0051	pos	960.57697	[M-OH+H]+			Global and overvie
< 0.001	0.0170	neg	305.1528	[M-H]-	Benzene a	Diphenylmethanes	
< 0.001	0.0053	pos	540.40794	[M-NH3+H]+			
< 0.001	0.0061	pos	849.15707	[M+Na]+			Amino acid metab
< 0.001	0.0191	neg	250.06234	[M-NH3-H]-	Carboxylic	Amino aci	Cell growth and de
< 0.001	0.0192	neg	154.13576	[M+HCOO]-	Prenol lipi	Monoterpe	Chemical structure
< 0.001	0.0194	neg	369.12123	[M+HCOO]-			Biosynthesis of otl
< 0.001	0.0196	neg	125.01466	[M-H]-	Organic su	Organosul	Membrane transpo
< 0.001	0.0067	pos	120.05751	[M-H2O+H]+	Benzene and substituted derivatives		
< 0.001	0.0069	pos	293.85272	[M+H]+			Xenobiotics biode
< 0.001	0.0077	pos	582.40727	[M-NH3+H]+			
< 0.001	0.0077	pos	568.30494	[M-OH+H]+	Tetrapyrro	Porphyrins	Metabolism of cof
< 0.001	0.0079	pos	176.05455	[M-NH3+H]+	Carboxylic	Amino aci	Global and overvie
< 0.001	0.0081	pos	413.98602	[M+NH4]+			
< 0.001	0.0082	pos	137.04768	[M+NH4]+			Metabolism of cof
< 0.001	0.0083	pos	211.12084	[M-OH+H]+	Benzene a	Methoxybenzenes	
< 0.001	0.0084	pos	479.20563	[M+K]+	Pyridines a	Hydropyridines	
< 0.001	0.0085	pos	290.12263	[M+NH4]+	Carboxylic	Amino aci	Amino acid metab
< 0.001	0.0087	pos	741.56721	[M+K]+	Glyceroph	Glycerophosphocholines	
< 0.01	0.0091	pos	396.30281	[M+H]+	Prenol lipi	Triterpenoids	
< 0.01	0.0091	pos	516.29343	[M-NH3+H]+			Global and overvie
< 0.01	0.0092	pos	210.13682	[M+H]+	Carboxylic	Amino acids, peptides, and ar	
< 0.01	0.0092	pos	277.11701	[M+H]+	Fatty Acyl	Fatty amides	
< 0.01	0.0248	neg	293.14744	[M-H]-	Organooxy	Alcohols and polyols	
< 0.01	0.0095	pos	279.2562	[M+H]+	Fatty Acyl	Fatty amides	
< 0.01	0.0260	neg	399.16818	[M+HCO3]-			Chemical structure
< 0.01	0.0261	neg	344.18617	[M-H]-			
< 0.01	0.0099	pos	200.04082	[M+H]+	Indoles an	Indoles	
< 0.01	0.0100	pos	465.0797	[M+H]+	Organooxy	Carbohydrates and carbohydr	
< 0.01	0.0266	neg	450.33451	[M-H]-	Steroids ar	Bile acids,	Lipid metabolism;
< 0.01	0.0101	pos	450.37089	[2M+H]+	Steroids ar	Bile acids,	Global and overvie
< 0.01	0.0269	neg	336.23005	[M+HCOO]-	Fatty Acyl	Eicosanoids	
< 0.01	0.0275	neg	191.05824	[M-H]-	Indoles an	Indolyl car	Amino acid metab

< 0.01	0.0278	neg	468.2723	[M-H]-	Steroids ar	Steroidal glycosides	
< 0.01	0.0278	neg	184.03467	[M-NH3-H]-	Benzothio|	Dibenzothiophenes	
< 0.01	0.0280	neg	131.09462	[M-H]-	Carboxylic	Amino aci	Cancer: overview;
< 0.01	0.0111	pos	351.19467	[M-CO2+H]+			Biosynthesis of otl
< 0.01	0.0112	pos	548.29852	[M-H2O+H]+			
< 0.01	0.0115	pos	264.111	[M+NH4]+	Organooxy	Carbonyl c	Amino acid metab
< 0.01	0.0115	pos	713.53591	[M+Na]+	Glyceroph	Glycerophosphocholines	
< 0.01	0.0116	pos	336.23005	[M-H2O+H]+	Fatty Acyl	Eicosanoids	
< 0.01	0.0119	pos	835.14142	[M+NH4]+			Amino acid metab
< 0.01	0.0120	pos	911.15746	[M-OH+H]+	Fatty Acyl	Fatty acyl	Cell growth and de
< 0.01	0.0120	pos	338.2457	[M-CO2+H]+			Global and overvie
< 0.01	0.0120	pos	921.71862	[M+Na]+	Glyceroph	Glycerophosphocholines	
< 0.01	0.0120	pos	546.40727	[M-OH+H]+	Prenol lipi	Quinone a	Metabolism of cof
< 0.01	0.0121	pos	300.08348	[M-OH+H]+			Biosynthesis of otl
< 0.01	0.0121	pos	285.13649	[M+K]+			Chemical structure
< 0.01	0.0310	neg	494.36072	[M-H]-	Steroids ar	Steroid lactones	
< 0.01	0.0124	pos	218.05791	[M+H]+	Naphthale	Naphthoquinones	
< 0.01	0.0125	pos	468.34507	[M-NH3+H]+			
< 0.01	0.0128	pos	532.40688	[M+NH4]+			Metabolism of ter
< 0.01	0.0128	pos	479.30117	[M+H]+	Glyceroph	Glycerophosphoethanolamine	
< 0.01	0.0129	pos	406.27191	[M-H2O+H]+	Steroids ar	Bile acids, alcohols and deriv	
< 0.01	0.0132	pos	801.53083	[M-CO2+H]+	Glyceroph	Glycerophosphocholines	
< 0.01	0.0133	pos	103.06333	[M+H]+	Carboxylic	Amino acids, peptides, and ar	
< 0.01	0.0133	pos	187.04807	[M+NH4]+			Amino acid metab
< 0.01	0.0333	neg	602.35835	[M-NH3-H]-			
< 0.01	0.0138	pos	757.56213	[M+H]+	Glyceroph	Glycerophosphocholines	
< 0.01	0.0341	neg	516.05463	[M-H]-			Global and overvie
< 0.01	0.0142	pos	974.6588	[M-CO2+H]+			
< 0.01	0.0349	neg	354.24061	[M+HCO3]-			Lipid metabolism
< 0.01	0.0145	pos	112.01604	[M+H]+	Pyrans	Pyranones and derivatives	
< 0.01	0.0352	neg	308.04095	[M-H]-	Pyrimidine	Pyrimidine	Drug resistance: a
< 0.01	0.0145	pos	430.30829	[M+H]+	Steroids ar	Bile acids,	Lipid metabolism
< 0.01	0.0354	neg	349.09502	[M+HCO3]-			Biosynthesis of otl
< 0.01	0.0149	pos	564.39671	[M-NH3+H]+	Prenol lipi	Tetraterpe	Global and overvie
< 0.01	0.0149	pos	215.15214	[2M+H]+	Carboxylic	Amino acids, peptides, and ar	
< 0.01	0.0149	pos	464.29028	[M-OH+H]+	Glyceroph	Glycerophosphates	
< 0.01	0.0155	pos	318.21948	[M+H]+	Fatty Acyl	Fatty acids	Lipid metabolism
< 0.01	0.0372	neg	622.51134	[M-NH3-H]-			
< 0.01	0.0156	pos	829.56213	[M-CO2+H]+	Glyceroph	Glycerophosphocholines	
< 0.01	0.0156	pos	261.03789	[M+H]+	Thienopyridines		
< 0.01	0.0158	pos	344.0896	[M+H]+			
< 0.01	0.0160	pos	170.13067	[M+H]+			Biosynthesis of otl
< 0.01	0.0378	neg	265.95927	[2M-H]-	Organic pl	Phosphate	Carbohydrate meta
< 0.01	0.0162	pos	396.22768	[M+H]+	Glyceroph	Glycerophosphates	
< 0.01	0.0399	neg	234.16197	[M-H]-			Global and overvie
< 0.01	0.0175	pos	398.35485	[M+H]+	Steroids ar	Ergostane	Lipid metabolism;
< 0.01	0.0179	pos	198.16197	[M+NH4]+	Lactones	Delta valerolactones	
< 0.01	0.0419	neg	236.07184	[M-NH3-H]-			Biosynthesis of otl

< 0.01	0.0187	pos	291.18343	[M+H]+	Benzene and substituted derivatives		
< 0.01	0.0188	pos	586.27912	[M-OH+H]+			Metabolism of cof
< 0.01	0.0426	neg	202.14297	[M-H]-	Carboxylic	Amino acids, peptides, and ar	
< 0.01	0.0426	neg	296.07726	[M-H]-	Benzene ar	Trifluoromethylbenzenes	
< 0.01	0.0192	pos	515.29166	[M-H2O+H]+	Steroids ar	Bile acids,	Lipid metabolism;
< 0.01	0.0439	neg	448.10056	[M-H]-	Flavonoids	Flavonoid glycosides	
< 0.01	0.0200	pos	454.07371	[2M+H]+	Imidazole	1-ribosyl-i	Global and overvie
< 0.01	0.0200	pos	517.31682	[M-H2O+H]+	Glyceroph	Glycerophosphocholines	
< 0.01	0.0443	neg	654.53755	[M-H2O-H]-	Prenol lipi	Polyprenyl	Metabolism of cofa
< 0.01	0.0445	neg	410.31846	[M-H]-			Metabolism of cof
< 0.01	0.0206	pos	200.00859	[M+NH4]+	Organooxy	Carbohydr	Metabolism of cof
< 0.01	0.0455	neg	284.07568	[M-NH3-H]-	Purine nucleosides		Membrane transpo
< 0.01	0.0209	pos	519.33247	[M+H]+			
< 0.01	0.0211	pos	216.111	[M+NH4]+			Amino acid metabo
< 0.01	0.0465	neg	273.1001	[M+HCO3]-	Carboxylic	Amino acids, peptides, and ar	
< 0.01	0.0465	neg	340.10056	[M-NH3-H]-			Carbohydrate meta
< 0.01	0.0219	pos	268.08077	[M+H]+	Pyrazolo[3,4-d]pyrimidine glycosides		
< 0.01	0.0221	pos	202.08546	[M+H]+	Triazines	1,2,4-triazines	
< 0.01	0.0475	neg	164.06847	[M+HCOO]-			
< 0.01	0.0227	pos	825.53083	[M-CO2+H]+	Glyceroph	Glycerophosphocholines	
< 0.01	0.0227	pos	391.20082	[M+K]+			Biosynthesis of oth
< 0.01	0.0477	neg	548.35149	[M-H]-	Harmala alkaloids		
< 0.01	0.0232	pos	128.09496	[M+H]+			
< 0.01	0.0235	pos	342.11621	[M+NH4]+	Organooxy	Carbohydrates and carbohydra	
< 0.01	0.0237	pos	864.69952	[M-OH+H]+	Prenol lipi	Quinone and hydroquinone lip	
< 0.01	0.0237	pos	342.11621	[M+Na]+	Organooxy	Carbohydr	Carbohydrate meta
< 0.01	0.0239	pos	784.49727	[M+NH4]+			
< 0.01	0.0244	pos	290.26095	[M-H2O+H]+			
< 0.01	0.0502	neg	203.11709	[M-H]-	Imidazopy	Purines an	Signal transductio
< 0.01	0.0245	pos	463.28348	[M+H]+	Benzimidazoles		
< 0.01	0.0507	neg	173.99868	[M-H]-	Organic su	Arylsulfates	
< 0.01	0.0247	pos	412.29773	[M-H2O+H]+	Steroids ar	Vitamin D and derivatives	
< 0.01	0.0249	pos	568.42801	[M-CO2+H]+			
< 0.01	0.0250	pos	473.16589	[M+H]+	Pteridines	Pterins anc	Metabolism of cof
< 0.01	0.0521	neg	191.09799	[M+HCOO]-			Biosynthesis of oth
< 0.01	0.0253	pos	100.08881	[M+Na]+	Fatty Acyl	Fatty alcoh	Lipid metabolism;
< 0.01	0.0253	pos	263.18852	[M+H]+	Phenol eth	Anisoles	
< 0.01	0.0253	pos	271.08445	[M+NH4]+			
< 0.01	0.0254	pos	317.29298	[M+H]+	Organonitr	Amines	Lipid metabolism;
< 0.01	0.0260	pos	300.20892	[M-OH+H]+	Prenol lipi	Diterpenoids	
< 0.01	0.0547	neg	129.04259	[M-H]-	Carboxylic	Amino aci	Metabolism of oth
< 0.01	0.0265	pos	284.06847	[M+H]+	Isoflavono	O-methyla	Biosynthesis of oth
< 0.01	0.0551	neg	292.20383	[M-H]-			Lipid metabolism
< 0.01	0.0274	pos	518.18468	[M+NH4]+	Organooxy	Carbohydrates and carbohydra	
< 0.01	0.0569	neg	314.06512	[M-H]-	Azolidines	Imidazolidines	
< 0.01	0.0570	neg	328.0583	[M-NH3-H]-	Coumarins	Furanocou	Biosynthesis of oth
< 0.01	0.0571	neg	461.33524	[M+HCO3]-	Sphingolip	Glycosphin	Lipid metabolism
< 0.01	0.0574	neg	264.13615	[M-H]-	Phenol esters		

< 0.01	0.0282	pos	140.08373	[M-OH+H]+	Organooxy	Carbonyl compounds	
< 0.01	0.0282	pos	159.17354	[M+H]+	Organonitr	Amines	Metabolism of oth
< 0.01	0.0284	pos	100.05243	[M+H]+	Lactones	Delta valerolactones	
< 0.01	0.0583	neg	173.04768	[M-H]-	Quinolines	Quinoline carboxylic acids	
< 0.01	0.0298	pos	418.12638	[M+NH4]+			
< 0.01	0.0299	pos	466.34468	[2M+H]+			
< 0.01	0.0300	pos	515.30117	[M-OH+H]+			Metabolism of oth
< 0.01	0.0611	neg	234.07395	[M-H2O-H]-	Carboxylic	Tricarboxylic acids and deriva	
< 0.01	0.0305	pos	528.37558	[M-OH+H]+			Metabolism of terp
< 0.01	0.0311	pos	546.06519	[M+NH4]+	Pyrimidine	Pyrimidine	Biosynthesis of otl
< 0.01	0.0312	pos	320.19875	[M-CO2+H]+			
< 0.01	0.0312	pos	233.12632	[M+H]+	Carboxylic	Amino acids, peptides, and ar	
< 0.01	0.0315	pos	306.25587	[M+Na]+			Biosynthesis of otl
< 0.01	0.0317	pos	203.11575	[M+H]+	Carboxylic	Amino acids, peptides, and ar	
< 0.01	0.0317	pos	181.18304	[M+H]+	Organonitr	Cyclohexylamines	
< 0.01	0.0641	neg	187.06247	[M+HCOO]-	Triazines	Aminotria	Xenobiotics biode
< 0.01	0.0642	neg	166.04774	[M-H2O-H]-			Carbohydrate meta
< 0.01	0.0644	neg	500.30707	[M-H]-	Carboxylic	Amino acids, peptides, and ar	
< 0.01	0.0644	neg	326.12263	[M-NH3-H]-	Pteridines and derivat		Metabolism of cof
< 0.01	0.0644	neg	402.32863	[M+HCO3]-			Metabolism of terp
< 0.01	0.0323	pos	879.1824	[M+K]+			Glycan biosynthes
< 0.01	0.0324	pos	520.3036	[M-OH+H]+	Prenol lipi	Sesterterpenoids	
< 0.01	0.0330	pos	757.56213	[M+H]+			
< 0.01	0.0656	neg	288.09977	[M-H]-			
< 0.01	0.0331	pos	226.04774	[M-H2O+H]+	Keto acids	Gamma-ke	Chemical structure
< 0.01	0.0334	pos	434.37598	[M-NH3+H]+			Global and overvie
< 0.01	0.0665	neg	216.05686	[M-H]-	Carboxylic	Amino aci	Metabolism of cof
< 0.01	0.0350	pos	395.03581	[M+NH4]+	Lactams	Beta lactams	
< 0.01	0.0354	pos	210.0892	[M+Na]+	Phenols	Methoxypl	Chemical structure
< 0.01	0.0356	pos	542.12613	[M+H]+			
< 0.01	0.0704	neg	380.01917	[M-H]-	Diazines	Pyrimidine	Xenobiotics biode
< 0.01	0.0361	pos	205.11364	[M+K]+			Biosynthesis of otl
< 0.01	0.0709	neg	668.5532	[M+HCO3]-			Metabolism of cof
< 0.01	0.0367	pos	452.36541	[M-NH3+H]+	Prenol lipi	Diterpenoi	Metabolism of cof
< 0.01	0.0724	neg	334.2144	[M-H2O-H]-	Prenol lipi	Diterpenoi	Global and overvie
< 0.01	0.0736	neg	202.17214	[M+HCOO]-	Prenol lipi	Sesquiterpenoids	
< 0.01	0.0384	pos	296.23513	[M+H]+	Fatty Acyl	Fatty acids and conjugates	
< 0.01	0.0384	pos	122.03678	[M+NH4]+	Organooxy	Carbonyl c	Chemical structure
< 0.01	0.0388	pos	818.57551	[M-NH3+H]+			
< 0.01	0.0388	pos	296.17762	[M-CO2+H]+			Biosynthesis of otl
< 0.01	0.0389	pos	432.14203	[M+H]+	Organooxy	Carbohydrates and carbohydr	
< 0.01	0.0390	pos	301.05627	[M-H2O+H]+	Organooxy	Carbohydrates and carbohydr	
< 0.01	0.0756	neg	216.0245	[2M-H]-			
< 0.01	0.0398	pos	140.12011	[M+NH4]+	Organooxy	Carbonyl compounds	
< 0.01	0.0756	neg	55.934939	[M+HCOO]-	Homogeneous transiti		Metabolism of cof
< 0.01	0.0399	pos	373.20417	[M-OH+H]+	Stilbenes		Xenobiotics biode
< 0.01	0.0756	neg	192.15141	[M+HCOO]-	Prenol lipi	Monoterpenoids	
< 0.01	0.0759	neg	164.06847	[M+HCOO]-	Fatty Acyl	Fatty alcoh	Carbohydrate meta

< 0.01	0.0403	pos	446.19406	[M+H]+	Steroids ar	Steroidal g	Lipid metabolism
< 0.01	0.0405	pos	321.06959	[M+H]+	Carboxylic	Amino aci	Amino acid metab
< 0.01	0.0406	pos	575.17261	[M-NH3+H]+	Steroids ar	Pregnane s	Xenobiotics biode
< 0.01	0.0767	neg	501.28789	[M-H]-	Benzene a	Diphenylm	Digestive system
0.0100	0.0408	pos	167.04432	[M+H]+	Imidazopy	Purines and purine derivatives	
0.0102	0.0778	neg	175.04807	[M-H]-	Carboxylic	Amino aci	Amino acid metab
0.0102	0.0778	neg	405.26677	[M+HCOO]-			Biosynthesis of ot
0.0102	0.0415	pos	140.05857	[M-H2O+H]+			
0.0103	0.0415	pos	281.27185	[M+H]+	Fatty Acyl	Fatty amides	
0.0104	0.0787	neg	275.05587	[2M-H]-	Benzene a	Nitrobenze	Xenobiotics biode
0.0105	0.0793	neg	352.22496	[M+HCOO]-	Fatty Acyl	Eicosanoic	Lipid metabolism
0.0107	0.0427	pos	838.55978	[M-NH3+H]+	Glyceroph	Glycerophosphocholines	
0.0107	0.0428	pos	742.19563	[M+K]+			Biosynthesis of ot
0.0108	0.0802	neg	194.04265	[2M-H]-			Carbohydrate meta
0.0108	0.0432	pos	945.25096	[M+NH4]+	Fatty Acyl	Fatty acyl	Lipid metabolism
0.0109	0.0806	neg	154.13576	[2M-H]-	Prenol lipi	Monoterpe	Metabolism of ter
0.0109	0.0435	pos	188.12732	[M+H]+	Carboxylic	Amino acids, peptides, and ar	
0.0110	0.0435	pos	191.10586	[M+NH4]+	Tetrahydroisoquinolines		
0.0111	0.0809	neg	482.2224	[M-H]-			Biosynthesis of ot
0.0112	0.0441	pos	294.21948	[M+H]+	Fatty Acyl	Fatty acids and conjugates	
0.0113	0.0443	pos	781.56213	[M+H]+	Glyceroph	Glycerophosphocholines	
0.0113	0.0443	pos	300.06339	[M+NH4]+	Flavonoids	O-methylated flavonoids	
0.0114	0.0445	pos	432.30281	[M-NH3+H]+	Prenol lipi	Triterpeno	Biosynthesis of ot
0.0115	0.0821	neg	348.23005	[M+HCOO]-	Steroids ar	Hydroxyst	Lipid metabolism
0.0119	0.0461	pos	294.1732	[2M+H]+			Chemical structure
0.0120	0.0462	pos	515.29166	[M+H]+	Steroids ar	Bile acids, alcohols and deriv	
0.0121	0.0465	pos	938.10253	[M-CO2+H]+	Tannins	Hydrolyzable tannins	
0.0122	0.0852	neg	130.02661	[M-H]-	Fatty Acyl	Fatty acids	Carbohydrate meta
0.0124	0.0475	pos	278.98875	[M+H]+	Benzene a	Halobenzenes	
0.0126	0.0481	pos	268.08881	[2M+H]+	Phenanthre	Chrysenes	Xenobiotics biode
0.0127	0.0483	pos	284.214	[M-H2O+H]+	Prenol lipi	Retinoids	Metabolism of cof
0.0131	0.0889	neg	428.36541	[M-NH3-H]-	Prenol lipi	Triterpenoids	
0.0131	0.0494	pos	582.3377	[M-OH+H]+			Metabolism of ter
0.0132	0.0495	pos	588.32982	[M-NH3+H]+			Metabolism of ter
0.0132	0.0496	pos	86.073161	[M+NH4]+	Organooxy	Carbonyl compounds	
0.0135	0.0909	neg	275.15214	[M-H]-			Biosynthesis of ot
0.0137	0.0510	pos	248.04332	[M-OH+H]+			
0.0141	0.0923	neg	119.05824	[M-H]-	Carboxylic	Amino aci	Amino acid metab
0.0144	0.0524	pos	443.15935	[M-H2O+H]+			Biosynthesis of ot
0.0145	0.0939	neg	215.05587	[M-H]-	Glyceroph	Glyceroph	Lipid metabolism
0.0145	0.0528	pos	448.35524	[M-H2O+H]+	Steroids ar	Bile acids,	Global and overvie
0.0145	0.0941	neg	226.0702	[M-NH3-H]-			Biosynthesis of ot
0.0145	0.0941	neg	499.9375	[M-H]-			
0.0146	0.0941	neg	150.05282	[2M-H]-			
0.0146	0.0530	pos	208.08479	[M+H]+	Organooxy	Carbonyl c	Amino acid metab
0.0147	0.0531	pos	773.55704	[M+Na]+	Glyceroph	Glycerophosphocholines	
0.0147	0.0531	pos	225.11134	[M+H]+	Pyridines a	Pyridinium	Xenobiotics biode
0.0148	0.0954	neg	151.97795	[M+HCOO]-	Keto acids	Alpha-keto	Amino acid metab

0.0150	0.0536	pos	434.12129	[2M+H]+			Biosynthesis of otl
0.0152	0.0544	pos	124.05243	[M-H2O+H]+			Xenobiotics biode
0.0157	0.0554	pos	145.07389	[M+H]+	Carboxylic	Amino acids, peptides, and ar	
0.0158	0.0558	pos	339.99605	[M+Na]+			Cancer: overview;
0.0164	0.1004	neg	472.20971	[M-H2O-H]-			
0.0164	0.0571	pos	324.03587	[M+H]+	Pyrimidine	Pyrimidine	Global and overvie
0.0165	0.0574	pos	824.39962	[M+K]+	Carboxylic	Amino aci	Biosynthesis of otl
0.0166	0.0575	pos	327.14705	[M+H]+	Protoberberine alkaloi		Chemical structure
0.0166	0.1010	neg	219.12929	[2M-H]-			Biosynthesis of otl
0.0169	0.0583	pos	371.30354	[M-NH3+H]+	Fatty Acyl	Fatty acid esters	
0.0170	0.0584	pos	376.13107	[M+K]+	Cinnamic a	Hydroxycinnamic acids and d	
0.0171	0.1030	neg	396.30281	[M+HCO3]-			Metabolism of cof
0.0172	0.0590	pos	515.10699	[M+NH4]+			Global and overvie
0.0172	0.0590	pos	852.16797	[M-CO2+H]+			
0.0173	0.0591	pos	514.27778	[M+NH4]+	Prenol lipi	Terpene glycosides	
0.0181	0.0611	pos	523.14448	[M-H2O+H]+	Phenylpropanoic acids		
0.0182	0.1065	neg	406.03002	[M-H2O-H]-	Organooxy	Carbohydrates and carbohydra	
0.0182	0.0614	pos	580.1792	[M+K]+	Flavonoids	Flavonoid	Biosynthesis of otl
0.0183	0.0615	pos	441.34541	[M-H2O+H]+	Fatty Acyl	Fatty acid esters	
0.0184	0.0619	pos	143.09462	[M+H]+	Carboxylic	Amino acids, peptides, and ar	
0.0187	0.1082	neg	384.12158	[M+HCOO]-	Lactones	Gamma bu	Amino acid metab
0.0190	0.0631	pos	549.37942	[M+Na]+	Glyceroph	Glycerophosphocholines	
0.0191	0.1094	neg	219.11067	[M-H]-	Organooxy	Alcohols a	Metabolism of cof
0.0193	0.0639	pos	372.23005	[M+NH4]+	Organooxy	Carbonyl compounds	
0.0196	0.1105	neg	536.38654	[M+HCOO]-	Prenol lipi	Monoterpenoids	
0.0196	0.0646	pos	130.06299	[M+NH4]+	Lactones	Gamma butyrolactones	
0.0202	0.0661	pos	562.43857	[M-OH+H]+	Prenol lipi	Polyprenylphenols	
0.0203	0.0662	pos	372.25118	[M-CO2+H]+			Global and overvie
0.0203	0.0662	pos	75.06841	[2M+H]+			Amino acid metab
0.0207	0.0669	pos	162.05282	[M-OH+H]+			Carbohydrate meta
0.0207	0.0669	pos	125.99868	[M+NH4]+	Organic su	Sulfuric acid esters	
0.0207	0.1142	neg	399.10665	[M-H2O-H]-			
0.0208	0.1145	neg	334.0665	[M-H]-			
0.0208	0.1145	neg	388.11581	[M-H]-			Biosynthesis of otl
0.0210	0.0676	pos	159.01764	[M+H]+	Isothiocyanates		
0.0211	0.0677	pos	347.28241	[M-CO2+H]+	Organonitr	Amines	Nervous system; S
0.0211	0.1152	neg	452.14711	[M+HCO3]-			Global and overvie
0.0211	0.1152	neg	273.13649	[2M-H]-			Chemical structure
0.0216	0.1167	neg	326.07903	[M-NH3-H]-	Sterigmatocystins		Biosynthesis of otl
0.0218	0.0695	pos	250.1205	[M+K]+	Prenol lipi	Quinone and hydroquinone li	
0.0220	0.0698	pos	747.44048	[M+K]+			Global and overvie
0.0220	0.0699	pos	440.30553	[M+H]+			
0.0223	0.0706	pos	454.28314	[M+H]+	Benzene ar	Phenylbutylamines	
0.0224	0.1187	neg	155.05824	[M-H]-			Biosynthesis of otl
0.0232	0.0727	pos	172.01367	[M+H]+	Glyceroph	Glycerophosphates	
0.0233	0.0727	pos	446.30321	[M+H]+	Prenol lipi	Triterpenoids	
0.0233	0.1210	neg	332.09228	[M-H2O-H]-	Benzopher	Quaternary	Biosynthesis of otl
0.0235	0.1218	neg	362.17293	[M-NH3-H]-			Biosynthesis of otl

0.0236	0.1219	neg	592.45182	[M-H2O-H]-	Prenol lipi	Sesquiterpenoids	
0.0236	0.0735	pos	354.24061	[M-OH+H]+	Fatty Acyl	Eicosanoids	
0.0240	0.0744	pos	458.08491	[2M+H]+	Flavonoids	Flavonoid glycosides	
0.0242	0.1237	neg	302.18818	[M+HCOO]-			Lipid metabolism
0.0243	0.0751	pos	334.2144	[M-H2O+H]+	Fatty Acyl	Eicosanoic	Lipid metabolism;
0.0244	0.1240	neg	333.1001	[2M-H]-			Biosynthesis of otl
0.0248	0.0763	pos	666.22185	[M+H]+			
0.0251	0.0769	pos	119.05824	[M-H2O+H]+	Carboxylic	Amino aci	Energy metabolisn
0.0253	0.1261	neg	504.16903	[M+HCOO]-	Organooxy	Carbohydrates and carbohydr	
0.0255	0.0778	pos	151.04941	[M+H]+	Imidazopy	Purines an	Global and overvi
0.0257	0.1270	neg	730.48083	[M+HCO3]-			Metabolism of terp
0.0261	0.0793	pos	538.45383	[M-NH3+H]+			Metabolism of terp
0.0267	0.0807	pos	584.3349	[M-NH3+H]+			Global and overvie
0.0270	0.1310	neg	189.07897	[2M-H]-	Indoles an	Indolyl carboxylic acids and c	
0.0271	0.1313	neg	204.18779	[M+HCO3]-			Metabolism of terp
0.0273	0.0822	pos	476.20462	[M-OH+H]+	Steroids ar	Steroidal g	Lipid metabolism
0.0274	0.1321	neg	392.29264	[M+HCOO]-	Steroids ar	Bile acids,	Lipid metabolism
0.0279	0.1331	neg	102.03169	[M-H]-	Organooxy	Carbonyl c	Carbohydrate meta
0.0281	0.1335	neg	795.57777	[M-H]-			Cell growth and de
0.0286	0.0848	pos	287.11173	[M+H]+	Carboxylic	Amino acids, peptides, and ar	
0.0287	0.1352	neg	666.22185	[M+HCOO]-	Organooxy	Carbohydr	Endocrine system
0.0291	0.1356	neg	618.26895	[M-H]-	Organooxy	Carbonyl c	Xenobiotics biode
0.0292	0.1360	neg	161.96392	[M-H]-	Benzene ai	Halobenze	Xenobiotics biode
0.0295	0.1365	neg	419.24603	[M-NH3-H]-	Naphthopyrans		Biosynthesis of otl
0.0296	0.0870	pos	299.28241	[M+H]+	Organooxy	Carbonyl c	Lipid metabolism;
0.0297	0.0870	pos	126.04293	[M+H]+	Carboxylic	Amino acids, peptides, and ar	
0.0297	0.0871	pos	122.05791	[M+Na]+	Organooxy	Carbohydr	Membrane transpo
0.0297	0.1371	neg	392.11341	[M+HCOO]-			Chemical structure
0.0299	0.0876	pos	257.1124	[M-OH+H]+	Pteridines	Pterins anc	Metabolism of cof
0.0300	0.0876	pos	304.2766	[M+NH4]+			
0.0308	0.1402	neg	473.18318	[M-H]-			Biosynthesis of otl
0.0310	0.0894	pos	188.15247	[M-NH3+H]+	Carboxylic	Amino aci	Amino acid metab
0.0312	0.0897	pos	482.35485	[M-NH3+H]+			Global and overvi
0.0315	0.0905	pos	430.30829	[M+H]+			
0.0318	0.0911	pos	582.44366	[M+NH4]+			Metabolism of terp
0.0319	0.1436	neg	496.33999	[M-NH3-H]-			Metabolism of terp
0.0320	0.1440	neg	616.2803	[M+HCOO]-			Biosynthesis of otl
0.0321	0.0919	pos	827.54648	[M-CO2+H]+	Glyceroph	Glycerophosphocholines	
0.0325	0.0926	pos	336.23005	[M+H]+	Fatty Acyl	Eicosanoids	
0.0328	0.0931	pos	498.34976	[M-NH3+H]+			Global and overvi
0.0331	0.1466	neg	166.06299	[M-H]-	Phenylpropanoic acid		Amino acid metab
0.0332	0.1468	neg	278.07251	[M+HCO3]-	Carboxylic	Amino acids, peptides, and ar	
0.0338	0.1486	neg	419.19573	[M-NH3-H]-			Biosynthesis of otl
0.0338	0.0953	pos	439.21408	[M+H]+	Carboxylic	Amino acids, peptides, and ar	
0.0338	0.0953	pos	312.23005	[M-H2O+H]+	Fatty Acyl	Lineolic ac	Lipid metabolism;
0.0339	0.0955	pos	161.04768	[M+H]+	Indoles an	Indolecarboxylic acids and de	
0.0339	0.0955	pos	210.0892	[M+NH4]+			
0.0340	0.0955	pos	162.01644	[M-H2O+H]+	Keto acids	Gamma-ke	Amino acid metab

0.0345	0.0966	pos	555.27516	[M+NH4]+			Biosynthesis of otl
0.0349	0.0972	pos	220.0583	[M-H2O+H]+			Energy metabolisn
0.0349	0.1519	neg	511.23184	[M-H]-			Biosynthesis of otl
0.0349	0.1519	neg	150.05282	[M-H]-	Organooxy	Carbohydrates and carbohydr	
0.0351	0.0976	pos	507.36886	[M+H]+	Glyceroph	Glycerophosphocholines	
0.0354	0.1528	neg	169.05039	[M-H]-	Organic pl	Phosphate	Lipid metabolism
0.0356	0.0985	pos	501.28552	[M+H]+	Glyceroph	Glycerophosphoethanolamine	
0.0358	0.0989	pos	237.13917	[M-NH3+H]+			Biosynthesis of otl
0.0359	0.0990	pos	290.22457	[M+H]+			
0.0363	0.0999	pos	567.33247	[M+Na]+	Glyceroph	Glycerophosphocholines	
0.0365	0.1559	neg	467.97362	[M+HCOO]-	Pyrimidine	Pyrimidine	Global and overvie
0.0368	0.1008	pos	122.04801	[M+H]+	Pyridines a	Pyridinecarboxylic acids and	
0.0369	0.1011	pos	787.60907	[M+H]+	Glyceroph	Glycerophosphocholines	
0.0377	0.1025	pos	303.15426	[M+H]+	Carboxylic	Amino acids, peptides, and ar	
0.0377	0.1590	neg	240.07864	[M-H]-			
0.0378	0.1026	pos	190.13576	[M+NH4]+	Organooxy	Carbonyl compounds	
0.0384	0.1036	pos	314.2457	[M-OH+H]+			
0.0386	0.1039	pos	324.111	[M-OH+H]+			
0.0387	0.1620	neg	135.05449	[M-H]-	Imidazopy	Purines an	Metabolism of terp
0.0388	0.1042	pos	133.03751	[M+H]+	Carboxylic	Amino aci	Metabolism of cof
0.0397	0.1647	neg	220.10592	[2M-H]-	Organooxy	Carbohydrates and carbohydr	
0.0402	0.1068	pos	249.07832	[M-CO2+H]+	Carboxylic	Amino acids, peptides, and ar	
0.0404	0.1072	pos	382.38106	[M-OH+H]+	Fatty Acyl	Fatty alcohols	
0.0406	0.1076	pos	221.09127	[M+H]+	Pteridines	Pterins and derivatives	
0.0416	0.1093	pos	131.09462	[M+H]+	Carboxylic	Amino acids, peptides, and ar	
0.0417	0.1096	pos	394.14163	[M+K]+	Isoflavono	Rotenoids	
0.0419	0.1098	pos	454.47496	[M+NH4]+	Fatty Acyl	Fatty alcohols	
0.0421	0.1709	neg	307.0838	[M-NH3-H]-	Carboxylic	Amino aci	Membrane transpo
0.0421	0.1102	pos	121.05276	[M+Na]+	Organooxy	Carbonyl compounds	
0.0426	0.1721	neg	294.21948	[M-H]-	Fatty Acyl	Lineolic ac	Lipid metabolism
0.0428	0.1724	neg	310.16544	[2M-H]-	Cinnamic a	Hydroxyci	Biosynthesis of otl
0.0428	0.1118	pos	93.991092	[M+NH4]+	Organic di	Dialkyldisulfides	
0.0431	0.1123	pos	284.11206	[M+Na]+	Carboxylic	Amino acids, peptides, and ar	
0.0434	0.1128	pos	438.09405	[M+H]+	Pteridines	Alloxazines and isoalloxazine	
0.0436	0.1742	neg	230.01915	[M-H]-	Organooxy	Carbohydr	Metabolism of cof
0.0436	0.1742	neg	428.14711	[M-H]-			
0.0440	0.1139	pos	375.13266	[M+NH4]+	Piperidines	Piperidinecarboxylic acids an	
0.0441	0.1749	neg	228.07462	[M+HCO3]-	Pyrimidine	Pyrimidine	Membrane transpo
0.0441	0.1141	pos	101.12044	[M+H]+	Organonitr	Amines	
0.0445	0.1147	pos	599.30137	[M-CO2+H]+			Biosynthesis of otl
0.0446	0.1149	pos	371.17326	[M+H]+			Chemical structure
0.0449	0.1770	neg	116.04734	[M-H]-	Keto acids	Short-chai	Amino acid metab
0.0451	0.1158	pos	630.4504	[M+NH4]+			
0.0454	0.1164	pos	467.30117	[M+H]+	Glyceroph	Glycerophosphocholines	
0.0454	0.1164	pos	234.16197	[M+H]+	Prenol lipi	Terpene lactones	
0.0457	0.1168	pos	464.35016	[2M+H]+			Chemical structure
0.0458	0.1169	pos	886.15232	[M+Na]+			Biosynthesis of otl
0.0464	0.1180	pos	119.0735	[M+H]+	Indoles an	Indolines	

0.0465	0.1182	pos	491.28897	[M+Na]+			Metabolism of oth
0.0466	0.1183	pos	145.01975	[M+H]+			
0.0470	0.1188	pos	706.56885	[M+K]+			
0.0472	0.1834	neg	436.33411	[M+HCO3]-	Prenol lipi	Quinone a	Metabolism of cof
0.0474	0.1196	pos	504.16903	[M-H2O+H]+	Organooxy	Carbohydrates and carbohydr	
0.0477	0.1844	neg	646.18976	[M-NH3-H]-			Global and overvie
0.0481	0.1850	neg	118.0531	[M+HCO3]-	Benzimidazoles		
0.0489	0.1873	neg	159.98465	[M-H]-			Xenobiotics biode
0.0490	0.1223	pos	222.19835	[M+NH4]+	Prenol lipi	Sesquiterpenoids	
0.0492	0.1879	neg	159.99272	[M+HCO3]-			Xenobiotics biode
0.0493	0.1228	pos	616.36111	[M-CO2+H]+			Metabolism of terp
0.0495	0.1231	pos	187.12084	[M-H2O+H]+	Fatty Acyl	Fatty acids	Metabolism of cof
0.0495	0.1231	pos	198.07931	[M+H]+			
0.0496	0.1231	pos	197.99294	[M-H2O+H]+			
0.0496	0.1886	neg	677.08705	[M-NH3-H]-			Carbohydrate meta
0.0499	0.1237	pos	733.56213	[M+K]+	Glyceroph	Glycerophosphocholines	

CAS	HMDB
1124-11-4	HMDB0036584
392-12-1	HMDB0060484
38784-81-:	HMDB0037538
79171-48-5	
2430-95-7	HMDB0062243
:w maps; Metabolism of terpenoids and polyketides	
	HMDB0008485
62697-46-:	HMDB0039078
28957-33-7	
13018-54-'	HMDB0001439
2173206-3	HMDB0040689
6712-01-2	HMDB0001157
83-79-4	HMDB0034436
221093-41	HMDB0000153
30283-16-(	HMDB0032298
78-44-4	HMDB0014539
17812-24-'	HMDB0000867
)enoids and polyketides	
131647-34-2	
133613-76	HMDB0038568
196607-74	HMDB0032680
121-21-1	
512-04-9	
3184-13-2	HMDB0000214
119052-99	HMDB0032747
156281-30	HMDB0041212
566-65-4	HMDB0003759
138149-18	HMDB0060392
18178-54-(	HMDB0035593
77-06-5	HMDB0003559
	HMDB0060127
74758-60-4	
31867-59-	HMDB0001469
	HMDB0010381
987-78-0	HMDB0001413
95-01-2	HMDB0037088
578-74-5	HMDB0037340
47141-42-·	HMDB0015341
71-30-7	HMDB0000630
	HMDB0304474
71-00-1	HMDB0000177
	HMDB0061451
51235-04-:	HMDB0253153
10097-84-4	
613-32-1	

67604-48-2	
79551-86-:	HMDB0005998
:w maps; M	HMDB0036887
485-72-3	HMDB0005808
	HMDB0060758
75917-16-7	
	HMDB0011223
s	HMDB0114046
17298-37-2	
113565-32	HMDB0029710
472-61-7	HMDB0002204
56-86-0	HMDB0000148
56-85-9	HMDB0000641
91-64-5	HMDB0001218
1233509-8	HMDB0030323
49669-76-3	
139191-80	HMDB0249356
6712-03-4	HMDB0001493
636-58-8	HMDB0001049
106-24-1	HMDB0035155
ıer secondary metabolites; Global and overview maps	
107-35-7	HMDB0000251
192506-02	HMDB0062765
3337-62-0	
18457-54-0	
7412-77-3	HMDB0001097
99-16-1	HMDB0001209
22916-47-8	
535-83-1	HMDB0000875
390-28-3	HMDB0014861
106664-28	HMDB0014760
2387-71-5	HMDB0000052
	HMDB0011211
1672-65-7	HMDB0034192
:w maps; Metabolism of terpenoids and polyketides	
2873-36-1	HMDB0034276
958734-24	HMDB0006868
87304-79-:	HMDB0039780
3072-13-7	HMDB0062656
14686-61-4	
18446-73-6	
135531-86	HMDB0038631
33049-17-	HMDB0038410
547-98-8	HMDB0000601
87833-54-:	HMDB0033984
35687-86-(	HMDB0060104
2971-31-5	HMDB0035514

	HMDB0248414
132-65-0	HMDB0251165
73-32-5	HMDB0000172
179936-52-8	
508-77-0	
52450-38-	HMDB0004259
	HMDB0007930
82200-87-	HMDB0010216
6008-91-9	HMDB0001011
26926-09-(	HMDB0001375
38966-21-1	
	HMDB0008778
actors and '	HMDB0006818
ıer secondary metabolites; Global and overview maps	
486-39-5	
80483-89-:	HMDB0030423
1589-92-0	HMDB0030769
6785-34-8	
2932-09-4	
89576-29-·	HMDB0011506
911-40-0	HMDB0000391
	HMDB0008470
167222-96	HMDB0031654
185103-33-7	
	HMDB0008133
:w maps; Metabolism of terpenoids and polyketides	
68860-46-:	HMDB0001977
496-64-0	HMDB0032994
964-26-1	HMDB0001409
115538-85	HMDB0012458
131984-76-4	
514-78-3	HMDB0003154
79251-60-:	HMDB0013279
	HMDB0062305
106154-18	HMDB0010217
	HMDB0008412
	HMDB0013926
7562-61-0	
ıer secondary metabolites; Global and overview maps	
38168-82-(	HMDB0001270
	HMDB0062324
347377-92-8	
516-86-9	HMDB0304351
3051-22-7	HMDB0037742
ıer secondary metabolites; Global and overview maps	

512-15-2	HMDB0015114
20298-86-6	
30315-93-	HMDB0001539
38677-85-	HMDB0252340
145-42-6	HMDB0000036
28608-75-	HMDB0030614
3031-95-6	HMDB0000797
62512-91-	HMDB0010388
actors and	HMDB0304113
14101-61-	HMDB0012958
585-18-2	HMDB0001321
146-80-5	HMDB0000299
22252-07-	HMDB0010386
olism; Global and overview maps	
1596-67-4	HMDB0000667
1883-12-1	
16220-07-	HMDB0000481
41394-05-	HMDB0254501
40026-07-1	
	HMDB0008733
ner secondary metabolites; Global and overview maps	
	HMDB0242555
21568-87-6	
470-58-6	HMDB0029898
5677-55-4	HMDB0013111
3687-64-7	HMDB0005826
62025-49-	HMDB0039545
2365-40-4	HMDB0245646
202189-78	HMDB0240232
937-34-8	HMDB0060015
112828-00	HMDB0015567
2800-34-2	HMDB0000972
ner secondary metabolites; Global and overview maps	
928-96-1	HMDB0030003
149289-30	HMDB0013892
109437-82-3	
554-62-1	HMDB0004610
90044-20-	HMDB0302101
98-79-3	HMDB0000267
1890-99-9	HMDB0031720
52591-16-9	
88167-26-	HMDB0032777
1185234-9	HMDB0015350
6795-23-9	HMDB0030479
2238-90-6	HMDB0000648
97180-28-	HMDB0035805

42348-12-	HMDB0036176
56-19-9	HMDB0012189
42932-61-	HMDB0250981
1199266-7	HMDB0000842
452311-56-7	
13222-48-5	
83966-24-	HMDB0038083
524-01-6	
16752-71-	HMDB0001399
5677-55-4	
1977-33-9	HMDB0246316
ıer secondary metabolites; Global and overview maps	
179795-15	HMDB0240626
101-83-7	HMDB0251214
6190-65-4	HMDB0033249
608-53-7	
112592-90	HMDB0005770
25351-45-	HMDB0003826
53872-50-7	
is and metabolism; Global and overview maps	
172616-87	HMDB0041366
8002-43-5	HMDB0007973
23444-65-7	
87664-40-	HMDB0012283
164034-47-3	
16968-98-	HMDB0004821
91832-40-	HMDB0014675
20675-96-	HMDB0013070
3237-49-8	HMDB0060418
ıer secondary metabolites; Global and overview maps	
actors and vitamins; Global and overview maps	
572-96-3	HMDB0004198
26109-32-	HMDB0036763
4176-17-4	HMDB0059878
32381-42-	HMDB0004702
90-02-8	HMDB0034170
502-70-5	
17063-94-	HMDB0031723
901851-43	HMDB0001367
1016-05-3	
30551-15-	HMDB0031269
15438-31-	HMDB0000692
162070-61	HMDB0060668
3548-78-5	HMDB0032498
13074-08-	HMDB0060267

2479-90-5	HMDB0004483
69281-09-(	HMDB0013220
;radation a	HMDB0060423
1319714-8	HMDB0005030
5614-64-2	HMDB0002032
1681-96-5	HMDB0003470
ıer secondary metabolites; Global and overview maps	
698-30-6	
301-02-0	HMDB0002117
311-45-5	HMDB0013035
79516-82-:	HMDB0001509
	HMDB0061470
129235-39-8	
13425-76-8	
	HMDB0003952
38049-26-:	HMDB0303825
156-86-5	HMDB0000670
59333-79-:	HMDB0006468
ıer secondary metabolites; Global and overview maps	
4179-43-5	HMDB0112102
998-06-1	HMDB0008138
1592-70-7	HMDB0302564
650-69-1	HMDB0035091
1167-48-2	HMDB0006762
: transformation maps	
25613-05-:	HMDB0258742
58970-75-:	HMDB0039265
498-23-7	HMDB0000634
	HMDB0247134
72010-13-(	HMDB0060091
79-80-1	HMDB0013117
616897-75	HMDB0062390
›enoids and polyketides	
›enoids and polyketides	
96-17-3	HMDB0031525
ıer secondary metabolites	
18819-45-9	
144-98-9	HMDB0004041
140924-01-2	
1190-00-7	HMDB0059660
92751-21-:	HMDB0034423
ıer secondary metabolites; Global and overview maps	
1763-23-1	HMDB0059586
7296-56-2	HMDB0000646
2922-83-0	HMDB0000684
	HMDB0285953
85352-99-·	HMDB0041953
88947-38-(	HMDB0001405

25218-09-1	
620-24-6	
1219795-0	HMDB0000730
34693-23-	HMDB0001058
35606-75-8	
58-97-9	HMDB0000288
1217704-9	HMDB0014681
6451-72-5	HMDB0304001
ıer secondary metabolites; Global and overview maps	
25597-07-	HMDB0005066
erivatives	HMDB0033932
57576-81-5	
ɛw maps; Metabolism of terpenoids and polyketides	
	HMDB0034677
	HMDB0253049
ate conjuga	HMDB0304681
10236-47-	HMDB0002927
	HMDB0013339
471-87-4	HMDB0004827
979-92-0	HMDB0000939
	HMDB0010391
79-83-4	HMDB0000210
210708-13	HMDB0032784
11079-53-	HMDB0030463
599-04-2	HMDB0303902
	HMDB0304230
53428-54-9	
2799-16-8	
ıbolism; Global and overview maps	
15066-87-	HMDB0031233
63598-46-9	
151120-47-7	
13028-50-	HMDB0031576
924894-98	HMDB0004080
104542-43-0	
4579-60-6	
6795-16-0	HMDB0030590
727-81-1	HMDB0002012
41451-91-6	
12698-55-4	
52-53-9	HMDB0001850
123409-86-9	
17181-54-	HMDB0002520
	HMDB0034424
2447-54-3	HMDB0029367
145265-02-7	

173449-96	HMDB0060196
745-65-3	HMDB0001442
4233-96-9	HMDB0301673
4416-57-3	
49825-91-	HMDB0060095
3606-45-9	
672-15-1	HMDB0000719
9013-95-0	HMDB0003539
66224-64-	HMDB0000132
863126-98-1	
58218-99-8	
w maps; Metabolism of terpenoids and polyketides	
1912-33-0	HMDB0029738
115888-31-8	
25577-70-	HMDB0004482
570-84-3	HMDB0000664
99043-16-	HMDB0002217
131350-53-3	
98379-91-	HMDB0002089
149820-99	HMDB0000757
181467-56	HMDB0060661
583-78-8	HMDB0041800
112900-05	HMDB0037531
16105-69-	HMDB0001480
22884-10-	HMDB0029736
149-32-6	HMDB0002994
23594-80-1	
1379003-9	HMDB0002281
82475-10-3	
55528-53-	HMDB0001325
5056-12-2	
6811-35-4	
38748-34-4	
enoids and polyketides	
er secondary metabolites; Global and overview maps	
	HMDB0008254
	HMDB0062619
2468-88-4	
495-78-3	HMDB0033752
121514-30	HMDB0304217
er secondary metabolites; Global and overview maps	
32886-97-	HMDB0015543
33964-75-	HMDB0003871
771-50-6	HMDB0003320
125074-06-8	
15044-42-	HMDB0060466

34291-03-7	
1; Global and overview maps	
12771-72-1	
41546-50-	HMDB0029941
	HMDB0013122
6909-62-2	HMDB0060244
s	HMDB0011488
ıer secondary metabolites; Global and overview maps	
1156-92-9	
162440-05	HMDB0010404
102814-08	HMDB0001191
1453-82-3	HMDB0253663
7276-38-2	HMDB0008102
31106-03-	HMDB0029143
4250-77-5	
23696-85-	HMDB0013804
73-24-5	HMDB0000034
56-84-8	HMDB0000191
112339-01	HMDB0001104
629653-16	HMDB0028794
	HMDB0035629
1797-87-1	HMDB0000389
327-57-1	HMDB0001645
522-17-8	HMDB0302647
155800-87	HMDB0041069
70-18-8	HMDB0000125
30440-88-	HMDB0035281
87984-82-	HMDB0010203
18696-26-	HMDB0029379
624-92-0	HMDB0005879
21435-29-	HMDB0028821
s	HMDB0059614
4300-28-1	HMDB0001548
23978-65-6	
1006863-6	HMDB0015042
951-78-0	HMDB0000012
111-26-2	HMDB0032323
ıer secondary metabolites; Global and overview maps	
transformation maps; Biosynthesis of other secondary metabolites	
759-05-7	HMDB0000019
63693-26-5	
20559-16-	HMDB0010379
97550-04-	HMDB0033252
80736-41-0	
31143-02-9	
496-15-1	HMDB0253472

er amino acids	
68006-11-1	
34625-86-(	HMDB0004649
597-12-6	HMDB0011730
;w maps; Metabolism of terpenoids and polyketides	
26985-65-!	HMDB0248993
95-73-8	
15051-81-'	HMDB0303908
150097-90-8	
›enoids and polyketides	
4707-58-8	HMDB0240687
487-03-6	HMDB0034217
70222-94-5	
2797-68-4	HMDB0000564
